# Contrasting Seasonal Variation of Photosynthesis in Evergreen and Deciduous Tree Species From a Tropical Forest

**DOI:** 10.1111/ppl.70410

**Published:** 2025-07-14

**Authors:** Rakesh Tiwari, Balachandra Hegde, Shrihari Hegde, Peddiraju Bandaru, M. Ramesh Babu, K. G. Somashekhara Achar, Caroline Greiser, Robert Muscarella, Deepak Barua, David Galbraith, Emanuel Gloor

**Affiliations:** ^1^ School of Geography University of Leeds Leeds West Yorkshire UK; ^2^ Plant Ecology and Evolution, Institute of Ecology and Genetics Uppsala Universitet Uppsala Sweden; ^3^ Sahyadri Ecological Observatory Sirsi Karnataka India; ^4^ Department of Environmental Science Kuvempu University Shivamogga Karnataka India; ^5^ Mangalore University Mangaluru Karnataka India; ^6^ Department of Biology Indian Institute of Science Education and Research Pune Maharashtra India; ^7^ Department of Electronic Media Bangalore University Bengaluru Karnataka India; ^8^ Department of Botany IDSG Government College Chikkamagaluru Karnataka India; ^9^ Panchavati Research Academy for Nature Shivamogga Karnataka India; ^10^ Department of Physical Geography Stockholm University Stockholm Sweden; ^11^ Bolin Centre for Climate Research Stockholm University Stockholm Sweden

**Keywords:** photosynthesis, seasonal plasticity, soil moisture, stomatal conductance, topography, trees, tropical forest, Western Ghats

## Abstract

Microclimate differences in water availability can drive seasonal water use and photosynthetic variation among co‐occurring tropical tree species, especially in forests with strongly seasonal climates. We studied a tropical forest site in the Western Ghats, India, and characterised seasonal differences in photosynthetic CO_2_ assimilation rates (*A*_net_) among nine tree species with contrasting leaf habit and topographic affinities: deciduous species in dry hilltops, dry‐affinity evergreens on slopes and wet‐affinity evergreens in valleys. Surface soil moisture was lowest in hilltops, intermediate on slopes and highest in valleys, with higher levels during the wet period compared to the dry period. As expected, deciduous species on dry hilltops showed higher photosynthetic rates at the thermal optimum (*T*_opt_) during the wet period, while evergreen species showed no overall seasonal differences. Interestingly, evergreen species with a dry affinity on hill slopes showed higher *A*_net_ at the thermal optimum during the dry period compared to the wet period, despite lower soil moisture. This suggests that these species either have sufficient water availability during the dry period or possess a warmer thermal niche preference/adaptation. Across species, stomatal conductance (*g*_s_) at *T*_opt_ was generally higher during the wet period, except for one evergreen species. Our findings illustrate seasonal differences in photosynthesis among tropical tree species across different leaf habits and topographic affinities.

## 1 Introduction

Globally, tropical forests exhibit diverse seasonal climate patterns, ranging from aseasonal to strongly seasonal climates with distinct wet and dry periods (Carvalho et al. 2021). Seasonally dry tropical forests, in particular, experience contrasting conditions of water availability and air temperature across seasons. This seasonality influences physiological processes among the tree species, which exhibit varying leaf habits and water affinities (Rey‐Sánchez et al. 2016). Furthermore, microclimate factors (Zhang et al. 2023), such as topography and distribution of tree species, can modulate seasonal variations in vegetation (Schwartz et al. 2022). A better understanding of the seasonal variation in physiological processes among co‐occurring tree species in these seasonally dry tropical forests could help us understand species' sensitivity to drought and future warming (Köpp Hollunder et al. 2022). For example, the extent of seasonal variation in photosynthesis – the primary carbon uptake process – is poorly understood, especially among adult trees exposed to diverse seasonal and microclimate‐driven differences in water availability.

Seasonal variation in photosynthesis is driven by complex interactions between environmental factors, including air temperature, water availability, light levels and daylength or photoperiod (Yamaguchi et al. 2016). Biochemical mechanisms also play a role, involving adjustments in enzyme and photosystem proteins, photosynthetic electron transport (Wada et al. 2023) and mitochondrial respiration (Way and Yamori 2014). These factors are often reflected in leaf nitrogen content, leaf pigments and leaf age/phenology (Yasumura et al. 2006; Muller et al. 2011). For instance, leaf nitrogen content typically peaks during early growth stages before declining with senescence (Joshi et al. 2024). Similarly, leaf pigment content, particularly chlorophyll, peaks alongside leaf nitrogen, while carotenoids, which play a key role in photoprotection, show smaller seasonal changes (Shi et al. 2014; Peng et al. 2021; Wada et al. 2023). As leaves age beyond their peak growth period, photosynthetic capacity declines due to reductions in leaf pigment and nitrogen content (Yasumura et al. 2006). Among co‐occurring tree species, especially in forests with high biodiversity, diverse phenology (Corredor‐Londoño et al. 2020; Devi et al. 2023; Wada et al. 2023) can lead to diverse seasonal patterns in photosynthesis.

Seasonal differences in photosynthesis parameters also indicate acclimation responses to seasonal environmental changes (Wittemann et al. 2022). Acclimation involves physiological, structural or biochemical adjustments that cause changes in the thermal optimum of photosynthesis (*T*_opt_), the photosynthetic rate at thermal optimum (*A*_opt_) and maximum rates of RuBP carboxylation, among other factors. Shifts in the thermal optima are associated with changes in rubisco carboxylation activation energy (Hikosaka et al. 2005; Borjigidai et al. 2006) and other temperature‐dependent processes across different photosynthetic pathways (Yamori et al. 2014). Generally, tropical tree species acclimate their photosynthesis to moderate warming, although to a lesser extent than temperate or boreal species (Slot and Winter 2017a; Wittemann et al. 2022; Liu et al. 2024). The extent of acclimation varies among species; for example, low‐elevation species acclimate more strongly to temperature increases than montane species (Wittemann et al. 2022). In general, the thermal optimum of photosynthesis increases with higher growth temperatures (Yamasaki et al. 2002; Hikosaka et al. 2005; Choury et al. 2022). While seasonal acclimation in *T*_opt_ indicates species' strategy to maximise or maintain photosynthetic efficiency throughout the year (Kattge and Knorr 2007), most knowledge is derived from studies on crop plants or juvenile trees (Gjindali et al. 2021; Gjindali and Johnson 2023). Therefore, a significant gap exists in research on adult trees in tropical forests with seasonal environmental variations.

Leaf‐level studies characterising seasonal patterns commonly report a decline in photosynthetic rates during the seasonally dry period, particularly for deciduous species (Eamus et al. 1999; Zhang et al. 2007). Leaf phenology and water availability are often identified as key factors explaining these seasonal variations (Eamus et al. 1999; Zhang et al. 2007). For instance, studies in Panama found reduced photosynthetic rates during the dry season in tree seedlings (Craven et al. 2011). Similar dry period declines were measured in Australian *Acacia* sp. juveniles (Montagu and Woo 1999). Leaf‐level measurements of adult trees conducted in situ at a tropical site in Thailand revealed consistent photosynthetic rates throughout the year for one evergreen species, while two other evergreen species exhibited declines during the dry season (Ishida et al. 2006). Similarly, a study in an Amazonian forest revealed a decline in photosynthetic rates among canopy trees during the dry season, while photosynthetic rates of understory species were only slightly reduced during the same period (Santos et al. 2018). Thus, a dry period reduction in photosynthetic rate is commonly reported. In contrast, some studies have reported higher photosynthetic rates during summer periods in deciduous tree species (Naidu and Swamy 1995) and seedlings of brevideciduous, deciduous and evergreen tree species in India (Abhilash and Devakumar 2023). These diverse responses suggest other regional factors, such as microclimate, could influence the seasonal differences in photosynthesis, particularly in seasonally dry tropical forests.

In addition to seasonal differences in photosynthetic rates, other aspects of gas exchange such as stomatal conductance to water vapour (*g*_s_) can exhibit seasonal variation. Under most common ambient conditions, stomatal conductance is closely linked with photosynthetic rates (Slot and Winter 2017b). However, at higher temperatures, *g*_s_ responses are often decoupled from photosynthesis (Asargew et al. 2024). The shape of the temperature response of *g*_s_ also varies. While a linear decrease in *g*_s_ with increasing temperatures is observed (Urban et al. 2017; Eze et al. 2024), some reports indicate increased *g*_s_ at higher temperatures and, in some cases, a peaking response (Yamori et al. 2006; Hernández et al. 2020). Generally, *g*_s_ tends to be higher during the wet period among deciduous trees (Grace et al. 1982), while some studies show that *g*_s_ of evergreen species do not vary significantly (Andriyas et al. 2021). However, trees in seasonal tropical forests, particularly adult trees, could potentially differ in their stomatal conductance strategies, especially during drier conditions. This seasonal variation may be due to the differences in air temperature, vapour pressure and water availability during dry and wet periods (Comita and Engelbrecht 2009; Schwartz et al. 2022), varied rooting depths (Stahl et al. 2013) and soil water availability (Vourlitis et al. 2005; Schmitt et al. 2022). Thus, while the extant literature provides some insights on the seasonal differences in photosynthesis, there is limited understanding of the seasonal differences in temperature response of photosynthesis among adult trees in a seasonally dry tropical forest.

Addressing this gap, we studied nine tree species in India's Central Western Ghats forest, a global biodiversity hotspot that experiences distinct dry, warm summer periods followed by four‐month‐long monsoons. This region has not been extensively studied for its photosynthetic thermal sensitivity or seasonal patterns. Topography, water availability and microclimate differences across the Western Ghats landscape (Das et al. 2015) create distinct microhabitats. Wet‐affinity, generally evergreen species occur in low‐lying valleys; deciduous trees are found on higher elevation hilltops with shallow soils; and relatively dry‐affinity evergreen species inhabit hill slopes with intermediate water availability (Pascal 1988; Krishnadas et al. 2016). The topographic differences further diversify the seasonal water availability as well as the environmental temperature experienced by species depending on their position along the hill slopes. This leads to the question: How do microhabitat and thermal niche differences relate to variation in photosynthetic thermal sensitivity among co‐occurring tree species?

To characterise seasonal differences in photosynthesis and stomatal conductance thermal sensitivity, we measured instantaneous CO_2_ assimilation rate and stomatal conductance temperature response curves for a set of nine tree species during two seasons. At the thermal optimum, photosynthetic rates are at their maximum potential (*A*_opt_) under saturating irradiance, ambient CO_2_ concentration and relative humidity (*RH*). Hence, *A*_opt_ during the dry and wet periods can provide a measure of seasonal variation in photosynthesis. As an indicator of stomatal conductance variation, we measured the *g*_s_ rate at *T*_opt_, representing the optimal *g*_s_ under the most likely leaf environment and the highest photosynthetic rate conditions during the two study periods. Our study addresses the following questions:

- In a seasonal tropical forest, to what extent do photosynthetic rates and stomatal conductance temperature responses differ among co‐occurring tree species between dry and wet seasons?
- Are seasonal differences in photosynthetic rates and stomatal conductance related to different topographic positions of species?

We hypothesise that photosynthetic rates for all species will be higher during the wet period. We further hypothesise that photosynthetic rate differences between wet and dry periods will be largest in hilltop deciduous species, intermediate in hill slope species and lowest in valley species. We expect *T*_opt_ to increase during the dry period, as previously observed in seedlings (Kositsup et al. 2008; Slot and Winter 2017a) and in response to experimental warming (Crous et al. 2022). Additionally, we expect stomatal conductance at *T*_opt_ to be generally higher during the wet period, but the magnitude of seasonal differences may vary among species across different water access strategies and topographic affinities.

## 2 Materials and Methods

### 2.1 Study Site and Tree Species

**FIGURE 1:**
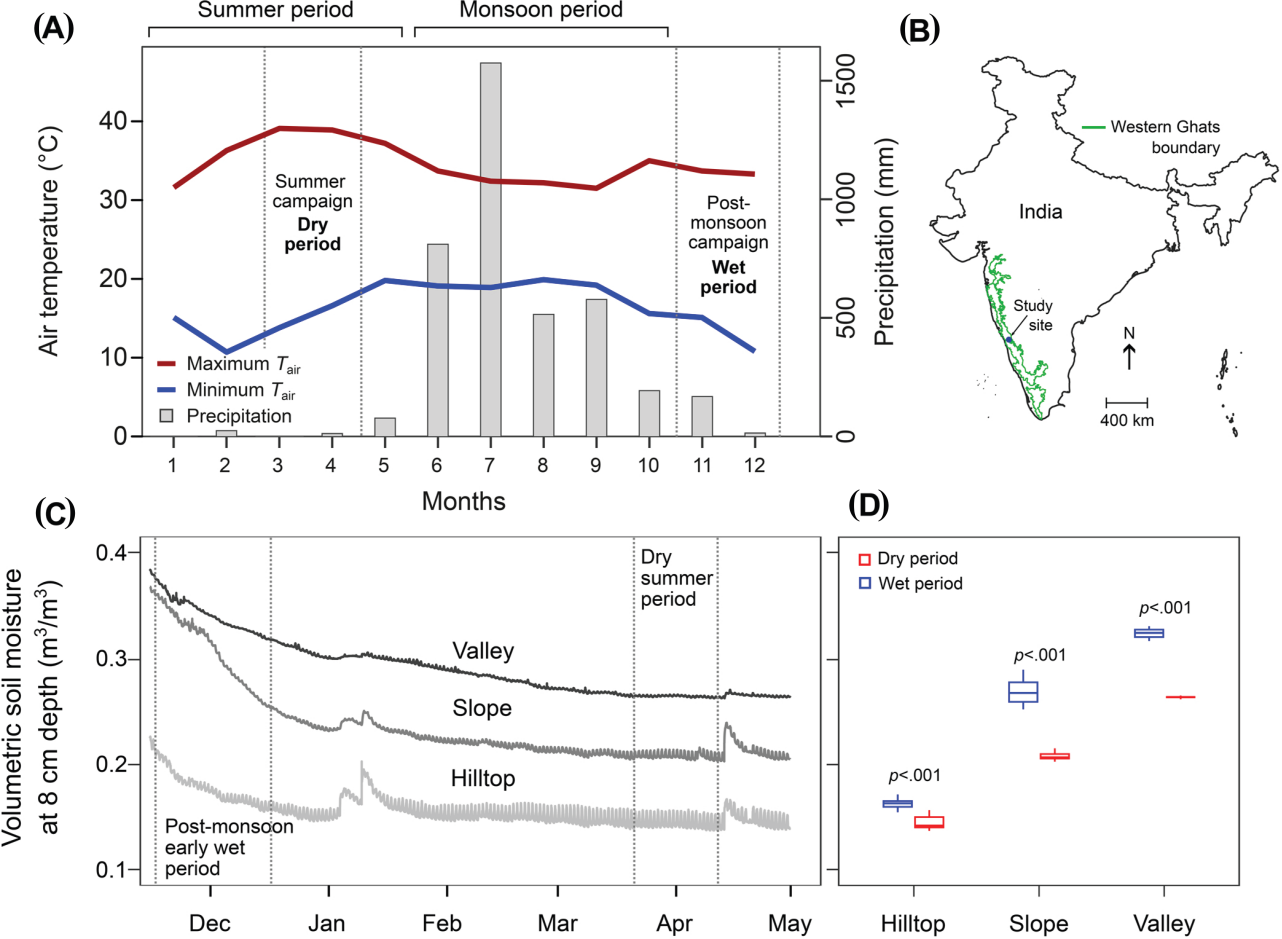
(A) Monthly air temperature (primary ordinate) and precipitation (secondary ordinate) of the study site in the Western Ghats, India. Data are monthly averages calculated using the data from a WatchDog 2000 weather station collected during 2020–2021. The map (B) shows the location of the study site in India and the Western Ghats boundary. Panel C shows surface soil moisture for three points corresponding to the three topographic groups measured in the subsequent year. Panel D shows mean surface soil moisture for the three strata derived from data in Panel C.

We conducted in situ measurements in a forest site located in a typical Central Western Ghats landscape of Uttara Kannada district, Karnataka, India (14.479157° N, 74.758304° E, 523 m asl; Figure 1). The study site features undulating terrain, with elevations ranging from 500 to 550 m in valleys to about 550–600 m on the ridges. We selected a set of three species in each of the three topographic positions. On hilltops that are characterised by sparsely distributed deciduous tree species, we chose *Careya arborea* Roxb. (Lecythidaceae), *Terminalia chebula* Retz. (Combretaceae) and *T. paniculata* B.Heyne ex Roth (Combretaceae) – all deciduous. Along the hill slopes with drier soils, we selected three evergreen species: *Memecylon umbellatum* Burm.f. (Melastomataceae), *Psydrax dicoccos* (Gaertn.) Merr. Rubiaceae and *Tetrapilus dioicus* Roxb. (Oleaceae). Last, in the wetter valleys, we selected three evergreen species: *Hopea ponga* Wall. (Dipterocarpaceae), *Knema attenuata* (Hook.f. & Th.) Warb. (Myristicaceae) and *Garcinia cambogioides* var. *cambogioides* (Clusiaceae). Hilltop trees such as *C. arborea* and *T. paniculata* are associated with wildfire‐prone areas, while *T. chebula* occurs in mixed deciduous forests, with Central Western Ghats being its most suitable habitat (Kailash et al. 2022). On hill slopes, *M. umbellatum* characterises mid‐elevation forests in Northern Western Ghats, associating with *Syzygium* spp. and *Actinodaphne* spp., occurring up to 1000 m in wet and dry forests (Shigwan et al. 2024), and often co‐occurring with *T. dioicus* . In valleys, *Hopea* spp. typify undisturbed evergreen forests and can also be observed to create monodominant patches near the study site. *K. attenuata* and *G. cambogioides* var. *cambogioides* characterise wetter valleys.

In our study site, deciduous species predominantly occupy hilltops and open areas, while evergreen species dominate slopes and valleys, revealing a clear topographical segregation. Conversely, it is hard to find deciduous species on hillslopes and valley regions. This segregation limited our ability to make direct comparisons across all zones for deciduous and evergreen types. The nine selected species collectively represent some of the most common trees in this ecosystem (Pascal 1988) and reflect general patterns of water affinity across the Western Ghats landscape (Krishnadas et al. 2021), closely linked to topographic positions. Due to the terrain and characteristic distribution of trees along the slope, branches from about 2–5 m above ground were accessible for measurement. For each individual tree sampled, we selected a branch for measurement, bent it towards the ground without damaging or detaching it from the tree and stabilised it using ropes to permit access for leaf measurements. We measured at least three biological replicates or individual trees per species, sampling the same individuals during the wet and dry period campaigns.

### 2.2 Sampling Periods

We conducted measurements during two periods: the early post‐monsoon period in 2020 and the dry summer period in 2021. The early post‐monsoon campaign – referred to as ‘wet period’ was conducted from November to mid‐December 2020, which is about 1–1.5 months after the 4‐month‐long monsoon rains (Figure 1A). The second campaign lasted from the end of March to mid‐April 2021 and coincided with the early to mid‐summer period (hereafter referred to as summer). The summer period is characterised by < 100 mm rainfall per month for about 4–5 months (from December to mid‐May, until the onset of monsoon). Typically, during the latter part of April and early May, air temperatures reach their highest levels in this area. The mean maximum air temperature of the summer period is 38.4°C which is about 4°C warmer than the post‐monsoon period (34°C). Relative humidity during the summer period was ~70.4% (59.6–82.3) while for the post‐monsoon period, it was 75.6% (65.3–87.0). The total mean annual precipitation for the site is about 4000 mm. Temperature and precipitation data are from a WatchDog 2000 automated weather station (Spectrum Technologies) set up in the study site. Vapour pressure deficit (VPD) remains stable at 2 kPa throughout the day during the wet period but varies from 2 to 3 kPa in the dry period. Supplementary Figure S1 illustrates the diurnal variations in air temperature, *RH* and leaf‐to‐air VPD between dry and wet periods.

### 2.3 CO_2_ Assimilation Rate Temperature Response Measurements

We measured the temperature response of the CO_2_ assimilation rate, *A*_net_, using a portable infrared gas analyser (IRGA) Li‐6400 XT (LiCor) with a fluorescence leaf chamber (Li‐6400‐40). We measured low to mid‐canopy, fully mature and healthy leaves from sun‐exposed branches of adult trees from about 2–5 m above ground. A typical measurement sequence involved gradual heating of the leaves in the leaf chamber to different temperature points. The target leaf temperatures were: 20°C, 24°C, 28°C, 32°C, 36°C, 40°C, 44°C and 48°C. The irradiance level was set to 1000 μ mol m^−2^ s^−1^, CO_2_ concentration to 400 μ mol mol^−1^. External PAR (photosynthetically active radiation) sensor data recorded by the instrument during the temperature response measurement were used to indicate microhabitat light levels.

To prevent moisture condensation in the IRGA, we set the lowest leaf temperature at 5°C above the dewpoint, which limited the lower temperatures that we were able to measure. We allowed leaves to stabilise at the initial chamber condition for at least 10 min. Following stabilisation, data were recorded after at least 6–7 min of stable conditions. After data logging, the chamber air was set to ~4°C higher temperature followed by a similar stabilisation sequence.

While we attempted to reach the nominally highest temperatures the instrument can reach, leaf temperatures often remained lower than the highest possible chamber temperature due to evapotranspirative cooling, limiting the upper end of the temperature range we could measure. The leaf temperature (*T*_leaf_) range for the summer period was 21.4°C–45.0°C and for the post‐monsoon period, it was 23.2°C–41.1°C. *RH* of the chamber air was maintained at around 60% ± 5%. However, at temperatures above 35°C, during both dry and wet period campaigns, *RH* levels dropped to around 40%. To estimate photosynthesis temperature response parameters, we fitted June et al.'s (June et al. 2004) model to the CO_2_ assimilation rate response as follows:

(1) $$A_{\mathrm{net}}\left(T_{\text{leaf}}\right)=A_{\mathrm{opt}}\times e^{-{\left(\frac{T_{\text{leaf}}-T_{\mathrm{opt}}}{\Omega }\right)}^{2}}$$

**FIGURE 2:**
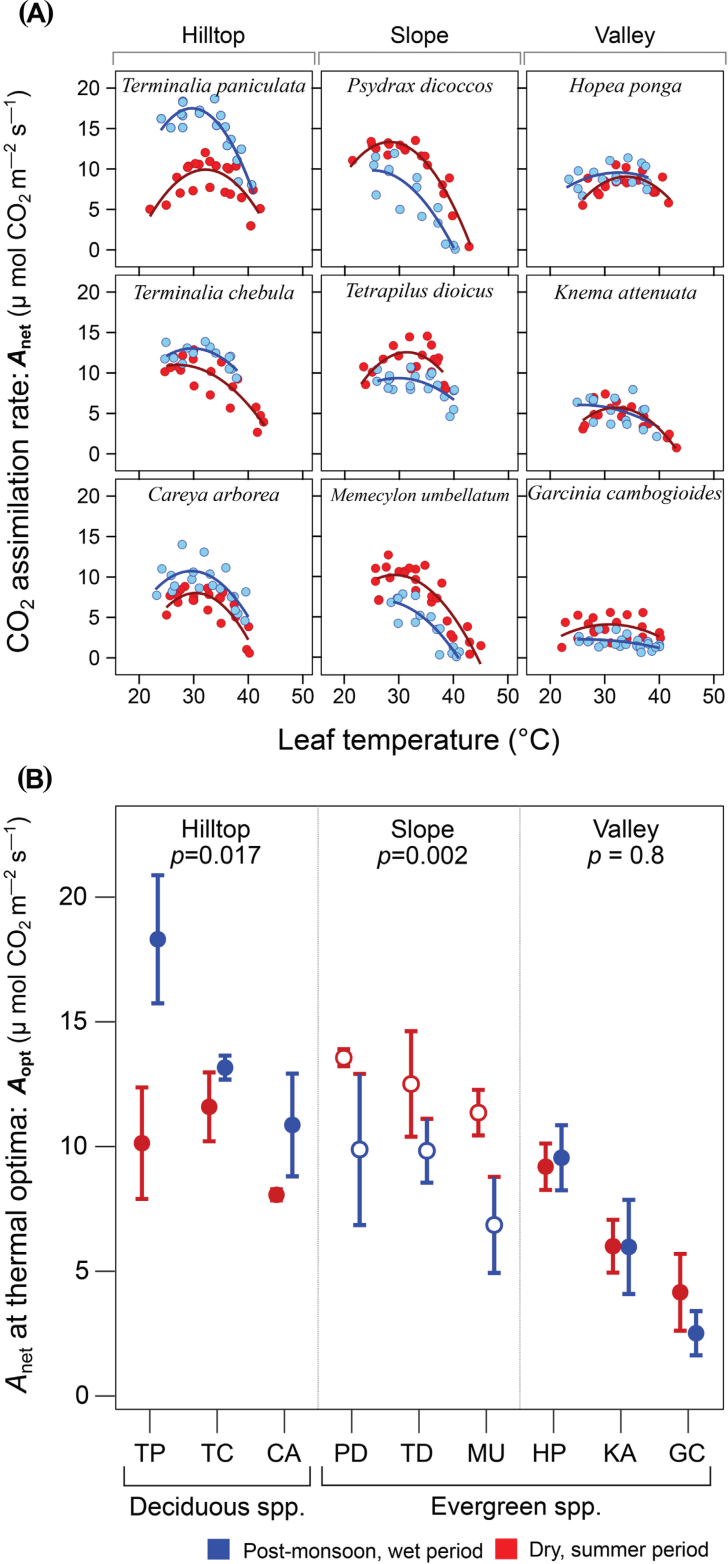
Photosynthetic CO_2_ assimilation rate temperature response curves (panel A, 3 × 3 grid) for 9 tree species in the Western Ghats, India, measured in situ. Blue components represent the wet period, and red components are for the dry period. Panel B shows the species average *A*_opt_, which is the maximum net CO_2_ assimilation rate at the thermal optimum (peaks of curves in Panel A). Measurements were conducted at an irradiance of 1000 μ mol m^−2^ s^−1^, [CO_2_] of 400 μ mol mol^−1^ and *RH* was maintained in the range of 50%–60%. Open circles in Panel B indicate evergreen species with significant seasonal differences in *A*_opt_. Codes for the species shown in Panel B are in parentheses as follows: *Terminalia paniculata* (TP), *Terminalia chebula* (TC), *Careya arborea* (CA), *Psydrax dicoccos* (PD), *Tetrapilus dioicus* (TD), *Memecylon umbellatum* (MU), *Hopea ponga* (HP), *Knema attenuata* (KA) and *Garcinia cambogioides* var. *cambogioides* (GC).

Where *A*_net_ is the photosynthetic CO_2_ assimilation rate at a given leaf temperature, CO_2_ concentration and irradiance level. *T*_opt_ is the thermal optimum of *A*_net_ and *A*_opt_ is the *A*_net_ at the thermal optimum or the maximum CO_2_ assimilation rate. Parameter *Ω*, which represents the width of the curve peak, is the temperature difference between *T*_opt_ and the temperature at which *A*_net_ drops to 37% of its value at *T*_opt_; at both supra and sub optimal temperatures. Compared to the parabolic temperature response curve of Cunningham and Read (2002), the asymmetric peaked function of June et al.'s (2004) provided a better fit to our data, particularly in capturing the peak (*T*_opt_). Temperature response curves were fitted for each species × period, combining replicate individual tree measurements (Figure 2, Panel A). Species mean *T*_opt_ was calculated by fitting curves to individual tree replicate data separately. In contrast, the curves shown in Figure 2, Panel A represents combined fits obtained by pooling all replicate tree data.

**FIGURE 3:**
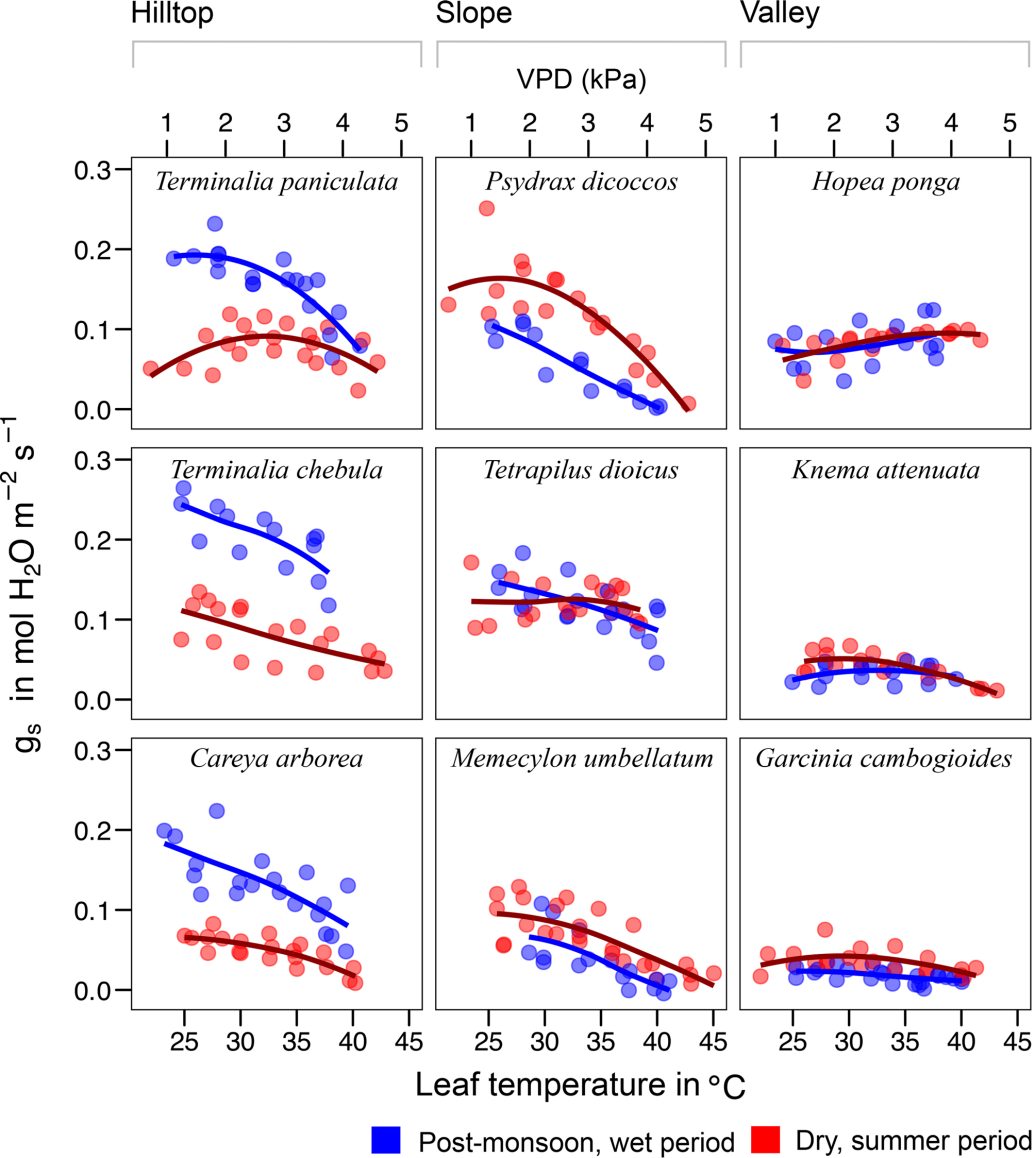
Temperature response of stomatal conductance (*g*_s_) to water for nine tree species in the Western Ghats, India, measured in situ, during post‐monsoon wet and summer dry periods. VPD corresponding to the datapoint is represented in the secondary x‐axis based on a model of *g*_s_, Tleaf and VPD. Curves are linear or quadratic fits (best fit selected based on AIC).

To test the effect of temperature on stomatal conductance (*g*_s_), linear models with and without a quadratic term were compared using Akaike information criterion (Cavanaugh and Neath 2019), and the best fit model was used. We present plots of *g*_s_ against leaf temperature *T*_leaf_ to highlight the diversity of physiological responses observed. Since *g*_s_ is influenced by both leaf temperature dynamics and environmental variables – including water vapour exchange between the leaf and surrounding air, we modelled *g*_s_ using *T*_leaf_ as the primary predictor. The best‐fit linear or quadratic relationships were selected based on the AIC value. To account for corresponding changes in leaf‐to‐air vapour pressure deficit (VPD) across temperature gradients, we fitted a linear regression model between VPD and *T*_leaf_. Using this model, we calculated VPD values for each *T*_leaf_ measurement and plotted them as secondary abscissa. Figure 3 demonstrates the VPD levels achieved through relative humidity *RH* control in the leaf chamber, while Supplementary Figure S1 illustrates diurnal VPD fluctuations recorded at the study site.

Several studies highlight the concerns about errors in leaf temperature measurements using poorly insulated thermocouples commonly used in IRGA Li‐6400 (Mott and Peak 2011; Still et al. 2019; Garen et al. 2022). However, Docherty et al. (2023) tested the differences in temperature response parameters derived from measurements using an instrument with a higher amount of bias (Li‐6400) versus a relatively lower bias (Li‐6800) and found no statistical difference. In our study, since the measurement temperature spanned the achievable range and the *A*_net_ commonly declined beyond the maximum *A*_net_ at *T*_opt_, the absolute values of *A*_opt_ should not be affected by the instrument temperature bias. While we recognise that the actual values of *T*_opt_ could be more directly affected, these effects should be similar in the data collected since the measurement was conducted using the same instrument. Hence, species‐wide differences that we report can nonetheless be interpreted with caution.

### 2.4 Surface Soil Moisture

Three TMT‐4 Standard dataloggers (Wild et al. 2019; TOMST) in the subsequent year (2023–2024) demonstrate marked seasonal differences in surface soil moisture (15‐min frequency at a depth of 8 cm below the soil surface) across the possible topographic strata. Calibration coefficients for the soil bulk density range 1.25–1.28 g/cm^3^ were used to convert the readings into volumetric soil moisture. The loggers were situated strategically, representing hilltop, slope and valley areas, and set to capture data at a 15‐min frequency at a depth of 8 cm below the soil surface.

Surface soil moisture levels were generally lowest in hilltops, followed by slopes and highest in valley regions (Figure 1C,D). For all three strata, soil moisture was seasonally different and was higher during the wet period compared to the dry period. The largest seasonal difference in surface soil moisture was recorded in the valley and slope positions, whereas, for hilltops, in contrast, experienced the lowest seasonal difference. Dry period soil moisture levels in the valley area were comparable with wet period slope levels.

### 2.5 Data Analysis

Data analysis was conducted in R version 4.4.3 (R Core Team 2025). For the temperature response function, we used the ‘nls’ function of the ‘stats’ package to fit *A*_net_ temperature function to Equation (1). *T*_opt_ and *A*_opt_ were calculated from the fitted models. Species‐level seasonal differences in parameter means were tested using paired T‐tests. Two‐way repeated measure linear mixed‐effect analysis of variance (ANOVA) models explaining *A*_opt_, *T*_opt_ and *g*_s_ at *T*_opt_ variation were fitted using the ‘lme’ function in the ‘nlme’ package (Pinheiro et al. 2018). Season, species and leaf habit (deciduous and evergreen) were included as fixed effects and individuals (trees) were included as a random effect in the models. Supplementary Section S2 presents all model fits. All means are presented with ± SEs and significant results are presented for 95% confidence interval (CI = 95%, α = 0.05).

## 3 Results

### 3.1 Thermal Optima of Photosynthetic CO_2_ Assimilation Rate

The average optimal temperature for CO_2_ assimilation rate, combining the values from the nine tree species, showed no seasonal difference between the wet (30.43°C ± 0.39°C) and dry (30.93°C ± 0.31°C) periods (*t*_49_ = 0.99, *p* = 0.325). Species mean *T*_opt_ was close to the mean maximum air temperature during the wet period (Supplementary Figure S3), whereas during the dry period, species mean *T*_opt_ was about 3°C below the mean maximum air temperature. *T*_opt_ differences were significantly related to species (*F*_9_ = 8.21, *p* < 0.001), but not to season (*F*_3_ = 3.2, *p* = 0.07) Additionally, the mixed‐effects model showed no significant interaction between species and season (*F*_9_ = 1.81, *p* = 0.106; Supplementary Section S2 and Figure 3).

### 3.2 CO_2_ Assimilation Rate at Thermal Optimum (*A*_opt_)

Species‐level *A*_net_ temperature response curves are presented in Figure 2A. The mean *A*_opt_ combining all nine species measured was similar during the wet and dry periods at 9.68 ± 0.59 and 9.71 ± 0.83 μ mol CO_2_ m^−2^ s^−1^, respectively, with no seasonal difference. The mixed‐effects model for *A*_opt_ showed a significant species effect (*F*_8_ = 24.8, *p* < 0.001) but no season effects (*F*_1_ = 0.003, *p* = 0.95). However, the interactive effect (*F*_8_ = 8.9, *p* < 0.001) of season and species was significant, indicating that seasonal differences in *A*_opt_ were species dependent. Testing leaf habit as a fixed effect, the *A*_opt_ model showed a significant effect of leaf habit (*F*_1_ = 13.7, *p* < 0.001) and the interaction between leaf habit and season (*F*_1_ = 10.1, *p* = 0.002). Season alone, however, was not a significant predictor (*F*_1_ = 0.003, *p* = 0.95). Results from mixed‐effects models are in Supplementary Section S2.

*A*_opt_ also differed significantly between deciduous and evergreen categories (*F*_1_ = 11.9, *p* = 0.001). Categorising the species by leaf habit (i.e., deciduous and evergreen) revealed distinct patterns. Specifically, deciduous species showed significant seasonal variation (*t*_14_ = 2.85, *p* = 0.012) with higher *A*_opt_ during the wet period (13.78 ± 1.17 μ mol CO_2_ m^−2^ s^−1^) compared to the dry summer period (9.93 ± 0.67 μ mol CO_2_ m^−2^ s^−1^), while evergreens did not change over the two seasons.

Although *A*_opt_ was comparable between evergreen and deciduous species during the dry period, *A*_opt_ of deciduous species (13.78 ± 1.17 μ mol CO_2_ m^−2^ s^−1^) was higher (*t*_16_ = 4.53, *p* < 0.001) than that of evergreen species (7.56 ± 0.72 μ mol CO_2_ m^−2^ s^−1^) during the wet period. Evergreen species further showed two distinct responses that depended on their typical topographic position. For three evergreen species, characteristic of wet valleys, *A*_opt_ did not differ seasonally between dry (6.45 ± 0.81 μ mol CO_2_ m^−2^ s^−1^) and wet periods (6.01 ± 1.1 μ mol CO_2_ m^−2^ s^−1^). In contrast, three slope‐affiliated evergreens differed seasonally significantly with higher *A*_opt_ during the dry period (12.36 ± 0.47 μ mol CO_2_ m^−2^ s^−1^) compared to the wet (8.95 ± 0.74 μ mol CO_2_ m^−2^ s^−1^).

Leaf‐level PAR data didn't explain *A*_opt_ variation (*p* = 0.49). Surface soil moisture significantly influenced *A*_opt_ (*F*_2_ = 11.15, *p* = 0.0046) without seasonal effects, partially explaining *A*_opt_ differences among species. The three evergreen species with seasonal differences are often associated with drier sites and are found on the hill slopes. In contrast, hilltop‐associated deciduous species showed higher *A*_opt_ during the wet period. Overall, deciduous species and three dry‐affinity evergreen species on the slopes showed significant and contrasting seasonal differences in *A*_opt_.

### 3.3 Differences in Stomatal Conductance at Thermal Optimum

The response of stomatal conductance to temperature varied across species (Figure 3). At *T*_opt_, *g*_s_ differed across species (*F*_8_ = 3.9, *p* = 0.027) but not between the dry and wet periods, indicating distinct species responses. Deciduous hilltop species showed lower *g*_s_ during the dry period, indicating that these species regulate stomatal water losses during the dry period. The *g*_s_ response of three valley‐associated evergreen species did not differ seasonally. For slope‐associated evergreen species, the *g*_s_ response was mixed. Specifically, while *Tetrapilus dioicus* showed no seasonal difference, the other species (*Psydrax dicoccos* and *Memecylon umbellatum*) showed higher *g*_s_ during the dry period (particularly clear in *Psydrax dicoccos*).

## 4 Discussion

We conducted in situ measurements of seasonal differences in photosynthetic rates at thermal optimum, *A*_opt_ and *g*_s_ at *T*_opt_ among co‐occurring tropical forest tree species in the Western Ghats, India, to characterise seasonal plasticity in photosynthetic rates. Deciduous species consistently showed higher *A*_opt_ in the wet period driven by higher stomatal conductance rates when water was abundant. In contrast, seasonal changes in *A*_opt_ and *g*_s_ for the evergreen species examined were contingent on topographic position. In contrast to what was seen in the deciduous species, the evergreen species from the hill slopes had higher *A*_opt_ in the dry summer period. The evergreen species from the valley that did not experience water limitations even in the dry season did not show any seasonal changes in *A*_opt_ and *g*_s_. Our results demonstrate complex effects of seasonal climate variability in water availability, microclimate conditions, phenological habits and thermal acclimation of photosynthesis.

### 4.1 Topographic Position Influence on Photosynthetic Seasonality

**FIGURE 4:**
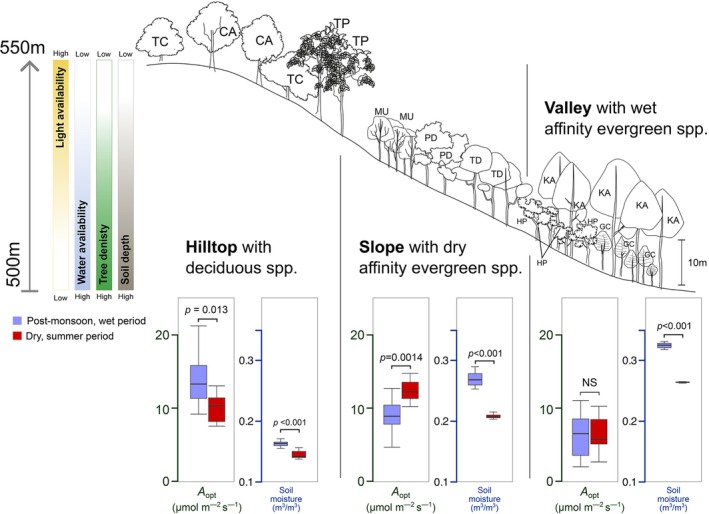
Schematic depicting the distribution of the tree species studied in the Western Ghats, India, and differences in photosynthetic rates at thermal optimum (green ordinate) and surface soil moisture content (blue ordinate, in m^3^/m^3^) as boxplots. Bars on the top left indicate the relative change in tree density, irradiance level and water availability changes from hilltops to valley areas. Codes for the species are as follows: *Terminalia paniculata* (TP), *Terminalia chebula* (TC), *Careya arborea* (CA), *Psydrax dicoccos* (PD), *Tetrapilus dioicus* (TD), *Memecylon umbellatum* (MU), *Hopea ponga* (HP), *Knema attenuata* (KA) and *Garcinia cambogioides* var. *cambogioides* (GC).

*A*_opt_ of trees from the three habitats probed, hilltops, slopes and valley areas, differed distinctly (Figure 4). *A*_opt_ variation across topography was similar to studies in the tropics (Harris and Medina 2013) and temperate sites (Tange 1996). Along the slope, surface water availability varies, especially during the dry season. Surface water availability was lowest at the hilltop, intermediate on the hill slope and highest in the valley areas. Our results from surface soil moisture measurement revealed that hilltop soils are exposed to less variable water levels between the two periods, while water in soils on slopes, as well as valley areas, increases significantly during the wet period. Although surface soil moisture showed a significant effect on *A*_*opt*_, the relationship did not show the effect of seasons. This indicates diverse rooting and seasonally different water access depths for these species that need to be explored further.

### 4.2 Some Evergreens Photosynthesise Optimally During Warm Periods Instead of the Wet Period

We found notable species‐wide dry and wet period differences in photosynthetic rates at thermal optima among co‐occurring tree species. Consistent with previous studies, *A*_opt_ of deciduous tree species was higher during the wet period, suggesting that these species harness water availability during the wet periods to maximise photosynthesis (Eamus et al. 1999; Craven et al. 2011). Similarly, seedling studies involving evergreen and deciduous species in Panama and Australia have reported higher photosynthetic rates during wet periods (Montagu and Woo 1999; Craven et al. 2011).

In contrast, evergreen species exhibited two distinct responses. *A*_opt_ of three evergreen species was the same for dry and wet periods, similar to findings from studies such as Cai et al. (2009). This is not surprising given that these species had the highest water availability across topographic classes, even in the dry season (Figure 1). However, for three other evergreen species, contrary to our expectation, *A*_opt_ was higher during the dry period than during the wet period. This behaviour is similar to findings from Venezuelan forests, where light‐saturated photosynthetic rates in some evergreens were higher during the dry period (Ávila‐Lovera et al. 2019), suspecting deeper roots among evergreens compared to deciduous in that site. Contrary to the general trend of higher photosynthesis during wet periods (Mujawamariya et al. 2023), these evergreen trees thrive in warmer, drier conditions. Their responses to limited water availability, likely due to access to deeper water sources (Nie et al. 2011) or other mechanisms such as thermal acclimation, suggest a higher thermal niche optimum.

Modelling studies also show that in some tropical forests with high annual rainfall or short dry periods, photosynthetic activity (including evergreens) is either less seasonal or peaked during drier periods (Uribe et al. 2021). Some seedling studies from India and Brazil also reported higher photosynthetic rates during dry periods (Ribeiro et al. 2009; Abhilash and Devakumar 2023). Another Amazonian study (Green et al. 2020) using chlorophyll fluorescence as a proxy for photosynthesis observed increased photosynthesis during drier periods in some of the wettest parts of the Amazonian forest, attributing the trends to younger/new leaves during this period. Our data, in contrast, do not show such linkages, as the seasonal differences and maxima spanned leaf age and leaf habit categories. For example, one dry‐affinity evergreen species, *Memecylon umbellatum*, had slightly younger leaves during the dry period, while the other two dry‐affinity species had older leaves (Supplementary Figure S4). This variation suggests that leaf age (phenology) does not fully explain the higher dry period photosynthetic rates. Thus, the mechanisms involved in the dry period increase in leaf‐level photosynthesis require further investigation.

Seasonal differences in *A*_opt_ among evergreens in our study were measured for three species that are commonly found in relatively dry areas along the slope (Pascal 1988; Krishnadas et al. 2021). These differing topographic associations also likely indicate variation in water access strategies and rooting depth among these species. Studies have shown that evergreen trees often access deeper water layers to maintain photosynthesis throughout the year (Hasselquist et al. 2010; Brinkmann et al. 2019). We may speculate that hilltops, often known for shallower soil depth, have relatively limited water availability compared to slopes and valleys, which would have deeper soil layers (Guha and Jain 2020) with higher water availability. Altogether, we add evidence from measurements on adult trees, in situ, that some evergreen species that are associated with drier microclimates achieve higher photosynthetic rates at thermal optima during the dry and warm period rather than the wet period.

### 4.3 Stomatal Conductance Seasonal Differences

We found that stomatal conductance responses to temperature across species and seasons differ among the co‐occurring species we studied (Figure 3). One reason could be that individual species' performance is affected by different water stress conditions (Schwartz et al. 2022), with seasonal differences in water availability in our study serving as a proxy for long‐term differences in water availability. Such variation also occurs within microclimates (Chitra‐Tarak et al. 2021; Ding et al. 2021). Topography‐based differences in water access and photosynthetic strategy can buffer some species against the effect of water scarcity during the summer period (Esteban et al. 2021).

Supporting season and topographic differences in *g*_s_ response, we find a diverse set of temperature responses, as well as seasonal differences among the tree species studied. For all three deciduous species, as expected, *g*_s_ generally declined with temperature, typically reaching higher *g*_s_ during the wet period compared to the dry period – similar to a study from a seasonally dry forest in Amazonia (Vourlitis et al. 2008; Sendall et al. 2009). Evergreen species, in contrast, demonstrated more diverse responses. Firstly, *g*_s_ response was generally similar between the dry and wet periods among evergreen species, while one evergreen species, *Psydrax dicoccos*, showed a seasonally distinct response and recorded higher *g*_s_ during the dry period, contrasting with other species studied. While *g*_s_ of *Memecylon umbellatum* was also higher during the dry period, it was statistically indifferent from wet period *g*_s_ at thermal optimum. Another evergreen species, *Hopea ponga*, although seasonally indifferent, recorded an increase in *g*_s_ with temperature, possibly indicating a thermal protection mechanism via evaporative cooling (Urban et al. 2017). This species also recorded a broad temperature response curve (Figure 2A) without a decline in *A*_net_ with higher temperature and was found in dominant patches in the vicinity, possibly indicating a diverse water use and leaf temperature regulation strategy in this species. It is also interesting to note that a different species of *Hopea (H. ferrea)* measured in a relatively drier tropical forest in Thailand (Ishida et al. 2014) recorded a significant dry period decline in contrast to our results where we find that *H. ponga* maintained the same photosynthetic rates during dry and wet periods.

### 4.4 Leaf Phenology, Light Availability and Microclimate Variation

Amazonian studies link leaf phenology to seasonal trends in photosynthesis (Wu et al. 2016; Chen et al. 2020). However, our data (Supplementary Figure S4) suggest a weak role for phenology in explaining photosynthetic differences. Notably, the leaves of deciduous trees were younger during the drier summer period (Supplementary Figure S4). While assimilation rates of young leaves are typically higher than mature leaves, for example, Green et al. (2020), *A*_opt_ values of the deciduous species in our study were higher during the wet period when the leaves were older compared to the dry period, indicating a stronger control of water availability rather than leaf age for these species. While a study in Panama (Kitajima et al. 1997) reported elevated photosynthesis, measured as O_2_ evolution rate, during dry periods – attributed to greater light availability in the absence of water stress – our findings contrast this pattern. In our study, leaf‐level PAR, recorded via the external IRGA sensor during measurements, showed no significant relationship with *A*_opt_ variation. Among the environmental variables tested (light availability and surface soil moisture), only surface soil moisture exhibited a limited explanatory power for photosynthetic rates at thermal optima.

To further test if the varied seasonal differences in *A*_opt_ were linked to rooting depth, we used an openly available dataset for our site to test the difference in midday and predawn leaf water potential data (Gloor et al. 2023) and found no effects on *A*_opt_. While surface soil moisture measurements provide some insights, effective rooting depth variation across species and soil moisture along the root depth is necessary to fully understand seasonal water access.

### 4.5 Implications for Understanding Drought Sensitivity

Our results reveal diverse photosynthetic and water use strategies among co‐occurring tree species, influenced by their topographic position. Wet‐affinity valley evergreens likely benefit from year‐round shallow groundwater access, while hilltop deciduous species may be more vulnerable to dry period water stress. Dry‐affinity slope evergreens showed higher dry period photosynthetic rates. While all species exhibit stomatal regulation that inherently optimises carbon gain relative to water loss (Andriyas et al. 2021), those with traits that enable more effective optimisation under water‐limited conditions are likely to be more drought tolerant. However, it is important to note that drought tolerance may also involve other physiological or ecological adaptations beyond stomatal behaviour, especially in extreme environments or at the community level (Blonder et al. 2023). Under predicted excess water conditions in the Western Ghats (Sarkar and Maity 2022), increased photosynthetic gains may be favoured over water conservation.

Understanding the interplay between water availability, species‐specific traits and topographic variation is crucial for predicting forest responses to climate change. For instance, topography‐based differences in water access and photosynthetic strategy can buffer some species against water scarcity (Esteban et al. 2021; Kühnhammer et al. 2023), suggesting drought could affect species within a community differently. While seasonal variations may not fully replicate future warming conditions, they provide insights into species' physiological trait plasticity and heterogeneous sensitivity to water stress (Janssen et al. 2020).

Our findings contribute to understanding seasonal drought tolerance and resilience mechanisms in co‐occurring tropical forest adult trees, particularly linked to shallow water tables that may provide crucial drought refuges (Costa et al. 2023).

## 5 Conclusion

Temperature response of photosynthesis measured in situ on adult trees during the dry summer and early post‐monsoon wet periods in a seasonally dry tropical forest site in the Central Western Ghats of India revealed significant interspecific variations. We observed distinct patterns in CO_2_ assimilation rates at thermal optimum and stomatal conductance among tree species found at different positions along the hill slopes. Deciduous species exhibit higher photosynthetic rates during the wet period, reflecting their characteristic seasonal pattern. In contrast, for evergreen species we found two patterns: among valley species, photosynthetic rates were the same during the wet and dry periods, while evergreen species on the slopes unexpectedly had higher photosynthetic rates in the drier period compared to the wet period, possibly indicating a preference for warmer temperatures. Our results demonstrate that co‐occurring tree species, including evergreens, exhibit diverse seasonal variations in photosynthesis at their thermal optima.

## Author Contributions

R.T. was responsible for the writing, conceptualisation, methodology, investigation, data curation, formal analysis and the writing of the original draft, the review and editing. B.H. did the writing and reviewing and was part of the methodology and investigation. S.H. took part in the writing in the form of reviewing and editing, as well as the investigation. P.B. and R.B.M. were part of the investigation. K.G.S.A. did the reviewing and editing. C.G. was part of the investigation, the writing, reviewing and editing, fund acquisition for microclimate loggers. R.M. did the reviewing and editing of the written text. D.B. did the writing, reviewing and editing, as well as the funding acquisition. D.G. did the writing, reviewing and editing and the supervision. E.G. was part of the writing, reviewing and editing, the supervision and funding acquisition. All authors read and approved the final manuscript.

## Supporting information


**Data S1:** Supporting Information.

## Data Availability

The data that support the findings of this study are openly available in the online Supporting Information 04. TOMST‐logger data will be made available upon request.

## References

[ppl70410-bib-0001] Abhilash, K. P. , and A. S. Devakumar . 2023. “Seasonal Photosynthesis Variations of Dominant Tree Species Used in Different Urban Landscapes.” International Journal of Environment and Climate Change 13: 562–571.

[ppl70410-bib-0002] Andriyas, T. , N. Leksungnoen , and P. Tor‐Ngern . 2021. “Comparison of Water‐Use Characteristics of Tropical Tree Saplings With Implications for Forest Restoration.” Scientific Reports 11: 1745. 33462324 10.1038/s41598-021-81334-0 PMC7813824

[ppl70410-bib-0003] Asargew, M. F. , Y. Masutomi , K. Kobayashi , and M. Aono . 2024. “Water Stress Changes the Relationship Between Photosynthesis and Stomatal Conductance.” Science of the Total Environment 907: 11. 10.1016/j.scitotenv.2023.167886 37858817

[ppl70410-bib-0004] Ávila‐Lovera, E. , R. Urich , I. Coronel , and W. Tezara . 2019. “Seasonal Gas Exchange and Resource‐Use Efficiency in Evergreen Versus Deciduous Species From a Tropical Dry Forest.” Tree Physiology 39: 1561–1571. 31135926 10.1093/treephys/tpz060

[ppl70410-bib-0005] Blonder, B. W. , L. M. T. Aparecido , K. R. Hultine , et al. 2023. “Plant Water Use Theory Should Incorporate Hypotheses About Extreme Environments, Population Ecology, and Community Ecology.” New Phytologist 238: 2271–2283. 36751903 10.1111/nph.18800

[ppl70410-bib-0006] Borjigidai, A. , K. Hikosaka , T. Hirose , T. Hasegawa , M. Okada , and K. Kobayashi . 2006. “Seasonal Changes in Temperature Dependence of Photosynthetic Rate in Rice Under a Free‐Air CO(2) Enrichment.” Annals of Botany 97: 549–557. 16399793 10.1093/aob/mcl001 PMC2803663

[ppl70410-bib-0007] Brinkmann, N. , W. Eugster , N. Buchmann , and A. Kahmen . 2019. “Species‐Specific Differences in Water Uptake Depth of Mature Temperate Trees Vary With Water Availability in the Soil.” Plant Biology 21: 71–81. 30184305 10.1111/plb.12907

[ppl70410-bib-0008] Cai, Z.‐Q. , S. A. Schnitzer , and F. Bongers . 2009. “Seasonal Differences in Leaf‐Level Physiology Give Lianas a Competitive Advantage Over Trees in a Tropical Seasonal Forest.” Oecologia 161: 25–33. 19418072 10.1007/s00442-009-1355-4 PMC2700874

[ppl70410-bib-0009] Carvalho, N. S. , L. O. Anderson , C. A. Nunes , et al. 2021. “Spatio‐Temporal Variation in Dry Season Determines the Amazonian Fire Calendar.” Environmental Research Letters 16: 125009.

[ppl70410-bib-0010] Cavanaugh, J. E. , and A. A. Neath . 2019. “The Akaike Information Criterion: Background, Derivation, Properties, Application, Interpretation, and Refinements.” Wiley Interdisciplinary Reviews: Computational Statistics 11: e1460.

[ppl70410-bib-0011] Chen, X. , F. Maignan , N. Viovy , et al. 2020. “Novel Representation of Leaf Phenology Improves Simulation of Amazonian Evergreen Forest Photosynthesis in a Land Surface Model.” Journal of Advances in Modeling Earth Systems 12: e2018MS001565.

[ppl70410-bib-0012] Chitra‐Tarak, R. , C. Xu , S. Aguilar , et al. 2021. “Hydraulically‐Vulnerable Trees Survive on Deep‐Water Access During Droughts in a Tropical Forest.” New Phytologist 231: 1798–1813. 33993520 10.1111/nph.17464 PMC8457149

[ppl70410-bib-0013] Choury, Z. , A. Wujeska‐Klause , A. Bourne , et al. 2022. “Tropical Rainforest Species Have Larger Increases in Temperature Optima With Warming Than Warm‐Temperate Rainforest Trees.” New Phytologist 234: 1220–1236. 35263440 10.1111/nph.18077 PMC9311211

[ppl70410-bib-0014] Comita, L. S. , and B. M. J. Engelbrecht . 2009. “Seasonal and Spatial Variation in Water Availability Drive Habitat Associations in a Tropical Forest.” Ecology 90: 2755–2765. 19886485 10.1890/08-1482.1

[ppl70410-bib-0015] Corredor‐Londoño, G.‐A. , J.‐W. Beltrán , A.‐M. Torres‐González , and A. Sardi‐Saavedra . 2020. “Phenological Synchrony and Seasonality of Eight Tree Species in a Fragmented Landscape in the Colombian Andes.” Revista de Biología Tropical 68: 987–1000.

[ppl70410-bib-0016] Costa, F. R. C. , J. Schietti , S. C. Stark , and M. N. Smith . 2023. “The Other Side of Tropical Forest Drought: Do Shallow Water Table Regions of Amazonia Act as Large‐Scale Hydrological Refugia From Drought?” New Phytologist 237: 714–733. 35037253 10.1111/nph.17914

[ppl70410-bib-0017] Craven, D. , D. Dent , D. Braden , M. S. Ashton , G. P. Berlyn , and J. S. Hall . 2011. “Seasonal Variability of Photosynthetic Characteristics Influences Growth of Eight Tropical Tree Species at Two Sites With Contrasting Precipitation in Panama.” Forest Ecology and Management 261: 1643–1653.

[ppl70410-bib-0018] Crous, K. Y. , J. Uddling , and M. G. De Kauwe . 2022. “Temperature Responses of Photosynthesis and Respiration in Evergreen Trees From Boreal to Tropical Latitudes.” New Phytologist 234: 353–374. 35007351 10.1111/nph.17951 PMC9994441

[ppl70410-bib-0019] Cunningham, S. , and J. Read . 2002. “Comparison of Temperate and Tropical Rainforest Tree Species: Photosynthetic Responses to Growth Temperature.” Oecologia 133: 112–119. 28547297 10.1007/s00442-002-1034-1

[ppl70410-bib-0020] Das, A. , H. Nagendra , M. Anand , and M. Bunyan . 2015. “Topographic and Bioclimatic Determinants of the Occurrence of Forest and Grassland in Tropical Montane Forest‐Grassland Mosaics of the Western Ghats, India.” PLoS One 10: e0130566. 26121353 10.1371/journal.pone.0130566 PMC4488301

[ppl70410-bib-0021] Devi, N. L. , F. Q. Brearley , and S. K. Tripathi . 2023. “Phenological Diversity Among Sub‐Tropical Moist Forest Trees of North‐Eastern India.” Journal of Tropical Ecology 39: e29.

[ppl70410-bib-0022] Ding, Y. , Y. Nie , H. Chen , K. Wang , and J. I. Querejeta . 2021. “Water Uptake Depth Is Coordinated With Leaf Water Potential, Water‐Use Efficiency and Drought Vulnerability in Karst Vegetation.” New Phytologist 229: 1339–1353. 32989748 10.1111/nph.16971

[ppl70410-bib-0023] Docherty, E. M. , E. Gloor , D. Sponchiado , et al. 2023. “Long‐Term Drought Effects on the Thermal Sensitivity of Amazon Forest Trees.” Plant, Cell & Environment 46: 185–198. 10.1111/pce.14465 PMC10092618 36230004

[ppl70410-bib-0024] Eamus, D. , B. Myers , G. Duff , and D. Williams . 1999. “Seasonal Changes in Photosynthesis of Eight Savanna Tree Species.” Tree Physiology 19: 665–671. 12651322 10.1093/treephys/19.10.665

[ppl70410-bib-0025] Esteban, E. J. L. , C. V. Castilho , K. L. Melgaço , and F. R. C. Costa . 2021. “The Other Side of Droughts: Wet Extremes and Topography as Buffers of Negative Drought Effects in an Amazonian Forest.” New Phytologist 229: 1995–2006. 33048346 10.1111/nph.17005

[ppl70410-bib-0026] Eze, C. E. , K. Winter , and M. Slot . 2024. “Vapor‐Pressure‐Deficit‐Controlled Temperature Response of Photosynthesis in Tropical Trees.” Photosynthetica 62: 318–325. 39649359 10.32615/ps.2024.034 PMC11622557

[ppl70410-bib-0027] Garen, J. C. , H. A. Branch , I. Borrego , B. Blonder , J. R. Stinziano , and S. T. Michaletz . 2022. “Gas Exchange Analysers Exhibit Large Measurement Error Driven by Internal Thermal Gradients.” New Phytologist 236: 369–384. 35762843 10.1111/nph.18347

[ppl70410-bib-0028] Gjindali, A. , H. A. Herrmann , J.‐M. Schwartz , G. N. Johnson , and P. I. Calzadilla . 2021. “A Holistic Approach to Study Photosynthetic Acclimation Responses of Plants to Fluctuating Light.” Frontiers in Plant Science 12: 668512. 33936157 10.3389/fpls.2021.668512 PMC8079764

[ppl70410-bib-0029] Gjindali, A. , and G. N. Johnson . 2023. “Photosynthetic Acclimation to Changing Environments.” Biochemical Society Transactions 51: 473–486. 36892145 10.1042/BST20211245 PMC10212544

[ppl70410-bib-0030] Gloor, E. , D. Barua , D. R. Galbraith , B. Hegde , R. Sunny , and R. Tiwari . 2023. Leaf Water Potential of Tropical Forest Tree Species. NERC EDS Environmental Information Data Centre. 10.5285/252b6a14-8a0e-4a6f-a879-99dff46fec71.

[ppl70410-bib-0031] Grace, J. , D. U. U. Okali , and F. E. Fasehun . 1982. “Stomatal Conductance of Two Tropical Trees During the Wet Season in Nigeria.” Journal of Applied Ecology 19: 659.

[ppl70410-bib-0032] Green, J. K. , J. Berry , P. Ciais , Y. Zhang , and P. Gentine . 2020. “Amazon Rainforest Photosynthesis Increases in Response to Atmospheric Dryness.” Science Advances 6: eabb7232. 33219023 10.1126/sciadv.abb7232 PMC7679161

[ppl70410-bib-0033] Guha, S. , and V. Jain . 2020. “Role of Inherent Geological and Climatic Characteristics on Landscape Variability in the Tectonically Passive Western Ghat, India.” Geomorphology Amst 350: 106840.

[ppl70410-bib-0034] Harris, N. L. , and E. Medina . 2013. “Changes in Leaf Properties Across an Elevation Gradient in the Luquillo Mountains, Puerto Rico.” Ecological Bulletins 54: 169–180.

[ppl70410-bib-0035] Hasselquist, N. J. , M. F. Allen , and L. S. Santiago . 2010. “Water Relations of Evergreen and Drought‐Deciduous Trees Along a Seasonally Dry Tropical Forest Chronosequence.” Oecologia 164: 881–890. 20658152 10.1007/s00442-010-1725-y PMC2981736

[ppl70410-bib-0036] Hernández, G. G. , K. Winter , and M. Slot . 2020. “Similar Temperature Dependence of Photosynthetic Parameters in Sun and Shade Leaves of Three Tropical Tree Species.” Tree Physiology 40: 637–651. 32083285 10.1093/treephys/tpaa015

[ppl70410-bib-0037] Hikosaka, K. , K. Ishikawa , A. Borjigidai , O. Muller , and Y. Onoda . 2005. “Temperature Acclimation of Photosynthesis: Mechanisms Involved in the Changes in Temperature Dependence of Photosynthetic Rate.” Journal of Experimental Botany 57: 291–302. 16364948 10.1093/jxb/erj049

[ppl70410-bib-0038] Ishida, A. , S. Diloksumpun , P. Ladpala , et al. 2006. “Contrasting Seasonal Leaf Habits of Canopy Trees Between Tropical Dry‐Deciduous and Evergreen Forests in Thailand.” Tree Physiology 26: 643–656. 16452078 10.1093/treephys/26.5.643

[ppl70410-bib-0039] Ishida, A. , J.‐Y. Yamazaki , H. Harayama , et al. 2014. “Photoprotection of Evergreen and Drought‐Deciduous Tree Leaves to Overcome the Dry Season in Monsoonal Tropical Dry Forests in Thailand.” Tree Physiology 34: 15–28. 24336612 10.1093/treephys/tpt107

[ppl70410-bib-0040] Janssen, T. , K. Fleischer , S. Luyssaert , K. Naudts , and H. Dolman . 2020. “Drought Resistance Increases From the Individual to the Ecosystem Level in Highly Diverse Neotropical Rainforest: A Meta‐Analysis of Leaf, Tree and Ecosystem Responses to Drought.” Biogeosciences 17: 2621–2645.

[ppl70410-bib-0041] Joshi, R. K. , A. Mishra , R. Gupta , and S. C. Garkoti . 2024. “Leaf and Tree Age‐Related Changes in Leaf Ecophysiological Traits, Nutrient, and Adaptive Strategies of *Alnus nepalensis* in the Central Himalaya.” Journal of Biosciences 49: 1–14. 38287679

[ppl70410-bib-0042] June, T. , J. R. Evans , and G. D. Farquhar . 2004. “A Simple New Equation for the Reversible Temperature Dependence of Photosynthetic Electron Transport: A Study on Soybean Leaf.” Functional Plant Biology 31: 275–283. 32688899 10.1071/FP03250

[ppl70410-bib-0043] Kailash, B. R. , B. Charles , G. Ravikanth , S. Setty , and K. Kadirvelu . 2022. “Identifying the Potential Global Distribution and Conservation Areas for *Terminalia chebula* , an Important Medicinal Tree Species Under Changing Climate Scenario.” Tropical Ecology 63: 584–595.

[ppl70410-bib-0044] Kattge, J. , and W. Knorr . 2007. “Temperature Acclimation in a Biochemical Model of Photosynthesis: A Reanalysis of Data From 36 Species.” Plant, Cell & Environment 30: 1176–1190. 10.1111/j.1365-3040.2007.01690.x 17661754

[ppl70410-bib-0045] Kitajima, K. , S. S. Mulkey , and S. J. Wright . 1997. “Seasonal Leaf Phenotypes in the Canopy of a Tropical Dry Forest: Photosynthetic Characteristics and Associated Traits.” Oecologia 109: 490–498. 28307332 10.1007/s004420050109

[ppl70410-bib-0046] Köpp Hollunder, R. , M. L. Garbin , F. Rubio Scarano , and P. Mariotte . 2022. “Regional and Local Determinants of Drought Resilience in Tropical Forests.” Ecology and Evolution 12: e8943. 35646321 10.1002/ece3.8943 PMC9130645

[ppl70410-bib-0047] Kositsup, B. , P. Montpied , P. Kasemsap , P. Thaler , and E. Dreyer . 2008. “Photosynthetic Capacity and Temperature Responses of Photosynthesis of Rubber Trees (*Hevea brasiliensis* Müll. Arg.) Acclimate to Changes in Ambient Temperatures.” Trees 23: 357–365.

[ppl70410-bib-0048] Krishnadas, M. , A. Kumar , and L. S. Comita . 2016. “Environmental Gradients Structure Tropical Tree Assemblages at the Regional Scale.” Journal of Vegetation Science 27: 1117–1128.

[ppl70410-bib-0049] Krishnadas, M. , M. Sankaran , N. Page , et al. 2021. “Seasonal Drought Regulates Species Distributions and Assembly of Tree Communities Across a Tropical Wet Forest Region.” Global Ecology and Biogeography 30: 1847–1862.

[ppl70410-bib-0050] Kühnhammer, K. , J. van Haren , A. Kübert , et al. 2023. “Deep Roots Mitigate Drought Impacts on Tropical Trees Despite Limited Quantitative Contribution to Transpiration.” Science of the Total Environment 893: 164763. 37308023 10.1016/j.scitotenv.2023.164763 PMC10331952

[ppl70410-bib-0051] Liu, J. , Y. Ryu , X. Luo , et al. 2024. “Evidence for Widespread Thermal Acclimation of Canopy Photosynthesis.” Nat Plants 10: 1919–1927. 39516387 10.1038/s41477-024-01846-1 PMC11649563

[ppl70410-bib-0052] Montagu, K. D. , and K. C. Woo . 1999. “Recovery of Tree Photosynthetic Capacity From Seasonal Drought in the Wet ‐ Dry Tropics: The Role of Phyllode and Canopy Processes in *Acacia auriculiformis* .” Functional Plant Biology 26: 135.

[ppl70410-bib-0053] Mott, K. A. , and D. Peak . 2011. “Alternative Perspective on the Control of Transpiration by Radiation.” Proceedings of the National Academy of Sciences 108: 19820–19823. 10.1073/pnas.1113878108 PMC3241800 22106306

[ppl70410-bib-0054] Mujawamariya, M. , M. Wittemann , M. E. Dusenge , et al. 2023. “Contrasting Warming Responses of Photosynthesis in Early‐ and Late‐Successional Tropical Trees.” Tree Physiology 43: 1104–1117. 36971469 10.1093/treephys/tpad035 PMC10335848

[ppl70410-bib-0055] Muller, O. , T. Hirose , M. J. A. Werger , and K. Hikosaka . 2011. “Optimal Use of Leaf Nitrogen Explains Seasonal Changes in Leaf Nitrogen Content of an Understorey Evergreen Shrub.” Annals of Botany 108: 529–536. 21757476 10.1093/aob/mcr167 PMC3158686

[ppl70410-bib-0056] Naidu, C. , and P. M. Swamy . 1995. “Seasonal Pattern of Photosynthetic Rate and Its Relationship With Chlorophyll Content, Ribulose‐1,5‐Bisphosphate Carboxylase Activity and Biomass Production.” Biology Plantarum 37: 349–354.

[ppl70410-bib-0057] Nie, Y.‐P. , H.‐S. Chen , K.‐L. Wang , W. Tan , P.‐Y. Deng , and J. Yang . 2011. “Seasonal Water Use Patterns of Woody Species Growing on the Continuous Dolostone Outcrops and Nearby Thin Soils in Subtropical China.” Plant and Soil 341: 399–412.

[ppl70410-bib-0058] Pascal, J. P. 1988. Wet Evergreen Forests of the Western Ghats of India. Institut francais de Pondichery.

[ppl70410-bib-0059] Peng, J. , Y. Feng , X. Wang , et al. 2021. “Effects of Nitrogen Application Rate on the Photosynthetic Pigment, Leaf Fluorescence Characteristics, and Yield of Indica Hybrid Rice and Their Interrelations.” Scientific Reports 11: 7485. 33820934 10.1038/s41598-021-86858-z PMC8021548

[ppl70410-bib-0060] Pinheiro, J. , D. Bates , S. DebRoy , D. S. And , and R Core Team . 2018. “Nlme: Linear and Nonlinear Mixed Effects Models.” In R Package. Comprehensive R Archive Network (CRAN) Repository.

[ppl70410-bib-0061] R Core Team . 2025. R: A Language and Environment for Statistical Computing. Comprehensive R Archive Network (CRAN) Repository.

[ppl70410-bib-0062] Rey‐Sánchez, A. C. , M. Slot , J. M. Posada , and K. Kitajima . 2016. “Spatial and Seasonal Variation in Leaf Temperature Within the Canopy of a Tropical Forest.” Climate Research 71: 75–89.

[ppl70410-bib-0063] Ribeiro, R. V. , E. C. Machado , M. G. Santos , and R. F. Oliveira . 2009. “Seasonal and Diurnal Changes in Photosynthetic Limitation of Young Sweet Orange Trees.” Environmental and Experimental Botany 66: 203–211.

[ppl70410-bib-0064] Santos, V. A. H. F. D. , M. J. Ferreira , J. V. F. C. Rodrigues , et al. 2018. “Causes of Reduced Leaf‐Level Photosynthesis During Strong El Niño Drought in a Central Amazon Forest.” Global Change Biology 24: 4266–4279. 29723915 10.1111/gcb.14293

[ppl70410-bib-0065] Sarkar, S. , and R. Maity . 2022. “Future Characteristics of Extreme Precipitation Indicate the Dominance of Frequency Over Intensity: A Multi‐Model Assessment From CMIP6 Across India.” Journal of Geophysical Research: Atmospheres 127: e2021JD035539.

[ppl70410-bib-0066] Schmitt, S. , S. Trueba , S. Coste , et al. 2022. “Seasonal Variation of Leaf Thickness: An Overlooked Component of Functional Trait Variability.” Plant Biology 24: 458–463. 35120262 10.1111/plb.13395

[ppl70410-bib-0067] Schwartz, N. B. , D. Medvigy , J. Tijerin , et al. 2022. “Intra‐Annual Variation in Microclimatic Conditions in Relation to Vegetation Type and Structure in Two Tropical Dry Forests Undergoing Secondary Succession.” Forest Ecology and Management 511: 120132.

[ppl70410-bib-0068] Sendall, K. M. , G. L. Vourlitis , and F. A. Lobo . 2009. “Seasonal Variation in the Maximum Rate of Leaf Gas Exchange of Canopy and Understory Tree Species in an Amazonian Semi‐Deciduous Forest.” Brazilian Journal of Plant Physiology 21: 65–74.

[ppl70410-bib-0069] Shi, C. , G. Sun , H. Zhang , et al. 2014. “Effects of Warming on Chlorophyll Degradation and Carbohydrate Accumulation of Alpine Herbaceous Species During Plant Senescence on the Tibetan Plateau.” PLoS One 9: e107874. 25232872 10.1371/journal.pone.0107874 PMC4169446

[ppl70410-bib-0070] Shigwan, B. K. , A. Kulkarni , V. Smrithy , and M. N. Datar . 2024. “An Overview of Tree Ecology and Forest Studies in the Northern Western Ghats of India.” iForest 17: 213–221.

[ppl70410-bib-0071] Slot, M. , and K. Winter . 2017a. “Photosynthetic Acclimation to Warming in Tropical Forest Tree Seedlings.” Journal of Experimental Botany 68: 2275–2284. 28453647 10.1093/jxb/erx071 PMC5447879

[ppl70410-bib-0072] Slot, M. , and K. Winter . 2017b. “In Situ Temperature Relationships of Biochemical and Stomatal Controls of Photosynthesis in Four Lowland Tropical Tree Species.” Plant, Cell & Environment 40: 3055–3068. 10.1111/pce.13071 28926102

[ppl70410-bib-0073] Stahl, C. , B. Hérault , V. Rossi , B. Burban , C. Bréchet , and D. Bonal . 2013. “Depth of Soil Water Uptake by Tropical Rainforest Trees During Dry Periods: Does Tree Dimension Matter?” Oecologia 173: 1191–1201. 23852028 10.1007/s00442-013-2724-6

[ppl70410-bib-0074] Still, C. J. , A. Sibley , G. Page , F. C. Meinzer , and S. Sevanto . 2019. “When a Cuvette Is Not a Canopy: A Caution About Measuring Leaf Temperature During Gas Exchange Measurements.” Agricultural and Forest Meteorology 279: 107737.

[ppl70410-bib-0075] Tange, T. 1996. “Seasonal Changes in Photosynthesis of Young *Cryptomeria japonica* Growing on Ridges and Foot‐Slopes.” Forest Ecology and Management 89: 93–99.

[ppl70410-bib-0076] Urban, J. , M. Ingwers , M. A. McGuire , and R. O. Teskey . 2017. “Stomatal Conductance Increases With Rising Temperature.” Plant Signaling & Behavior 12: e1356534. 28786730 10.1080/15592324.2017.1356534 PMC5616154

[ppl70410-bib-0077] Uribe, M. R. , C. A. Sierra , and J. S. Dukes . 2021. “Seasonality of Tropical Photosynthesis: A Pantropical Map of Correlations With Precipitation and Radiation and Comparison to Model Outputs.” Journal of Geophysical Research: Biogeosciences 126: e2020JG006123.

[ppl70410-bib-0079] Vourlitis, G. L. , J. de Souza Nogueira , F. de Almeida Lobo , et al. 2008. “Energy Balance and Canopy Conductance of a Tropical Semi‐Deciduous Forest of the Southern Amazon Basin.” Water Resources Research 44: 1–14.

[ppl70410-bib-0078] Vourlitis, G. , J. S. Nogueira , N. P. Filho , et al. 2005. “The Sensitivity of Diel CO_2_ and H_2_O Vapor Exchange of a Tropical Transitional Forest to Seasonal Variation in Meteorology and Water Availability.” Earth Interactions 9: 1–23.

[ppl70410-bib-0080] Wada, N. , I. Kondo , R. Tanaka , et al. 2023. “Dynamic Seasonal Changes in Photosynthesis Systems in Leaves of Asarum Tamaense, an Evergreen Understorey Herbaceous Species.” Annals of Botany 131: 423–436. 36579472 10.1093/aob/mcac156 PMC10072104

[ppl70410-bib-0081] Way, D. A. , and W. Yamori . 2014. “Thermal Acclimation of Photosynthesis: On the Importance of Adjusting Our Definitions and Accounting for Thermal Acclimation of Respiration.” Photosynthesis Research 119: 89–100. 23812760 10.1007/s11120-013-9873-7

[ppl70410-bib-0082] Wild, J. , M. Kopecký , M. Macek , M. Šanda , J. Jankovec , and T. Haase . 2019. “Climate at Ecologically Relevant Scales: A New Temperature and Soil Moisture Logger for Long‐Term Microclimate Measurement.” Agricultural and Forest Meteorology 268: 40–47.

[ppl70410-bib-0083] Wittemann, M. , M. X. Andersson , B. Ntirugulirwa , L. Tarvainen , G. Wallin , and J. Uddling . 2022. “Temperature Acclimation of Net Photosynthesis and Its Underlying Component Processes in Four Tropical Tree Species.” Tree Physiology 42: 1188–1202. 35038330 10.1093/treephys/tpac002 PMC9190752

[ppl70410-bib-0084] Wu, J. , L. P. Albert , A. P. Lopes , et al. 2016. “Leaf Development and Demography Explain Photosynthetic Seasonality in Amazon Evergreen Forests.” Science 351: 972–976. 26917771 10.1126/science.aad5068

[ppl70410-bib-0085] Yamaguchi, D. P. , T. Nakaji , T. Hiura , and K. Hikosaka . 2016. “Effects of Seasonal Change and Experimental Warming on the Temperature Dependence of Photosynthesis in the Canopy Leaves of *Quercus serrata* .” Tree Physiology 36: 1283–1295. 27107017 10.1093/treephys/tpw021

[ppl70410-bib-0086] Yamasaki, T. , T. Yamakawa , Y. Yamane , H. Koike , K. Satoh , and S. Katoh . 2002. “Temperature Acclimation of Photosynthesis and Related Changes in Photosystem II Electron Transport in Winter Wheat.” Plant Physiology 128: 1087–1097. 11891263 10.1104/pp.010919 PMC152220

[ppl70410-bib-0087] Yamori, W. , K. Hikosaka , and D. A. Way . 2014. “Temperature Response of Photosynthesis in C3, C4, and CAM Plants: Temperature Acclimation and Temperature Adaptation.” Photosynthesis Research 119: 101–117. 23801171 10.1007/s11120-013-9874-6

[ppl70410-bib-0088] Yamori, W. , K. Noguchi , Y. T. Hanba , and I. Terashima . 2006. “Effects of Internal Conductance on the Temperature Dependence of the Photosynthetic Rate in Spinach Leaves From Contrasting Growth Temperatures.” Plant & Cell Physiology 47: 1069–1080. 16816408 10.1093/pcp/pcj077

[ppl70410-bib-0089] Yasumura, Y. , K. Hikosaka , and T. Hirose . 2006. “Seasonal Changes in Photosynthesis, Nitrogen Content and Nitrogen Partitioning in Lindera Umbellata Leaves Grown in High or Low Irradiance.” Tree Physiology 26: 1315–1323. 16815833 10.1093/treephys/26.10.1315

[ppl70410-bib-0090] Zhang, C. , Y. Su , L. Liu , et al. 2023. “Seasonal and Long‐Term Dynamics in Forest Microclimate Effects: Global Pattern and Mechanism.” npj Climate and Atmospheric Science 6: 1–12.

[ppl70410-bib-0091] Zhang, J.‐L. , J.‐J. Zhu , and K.‐F. Cao . 2007. “Seasonal Variation in Photosynthesis in Six Woody Species With Different Leaf Phenology in a Valley Savanna in Southwestern China.” Trees 21: 631–643.

