# Supplementary material for: Contrasting Seasonal Variation of Photosynthesis in Evergreen and Deciduous Tree Species From a Tropical Forest
Source: Physiol Plant. 2025 Jul 14;177(4):e70410. doi: 10.1111/ppl.70410 (PMC12257110; doi:10.1111/ppl.70410)
Supplement: Supplementary file 1 — Data S1: Supporting Information. [file PPL-177-e70410-s001.zip › 3_Supplementary-Section-01.pdf]

Supplementary Figure 1.

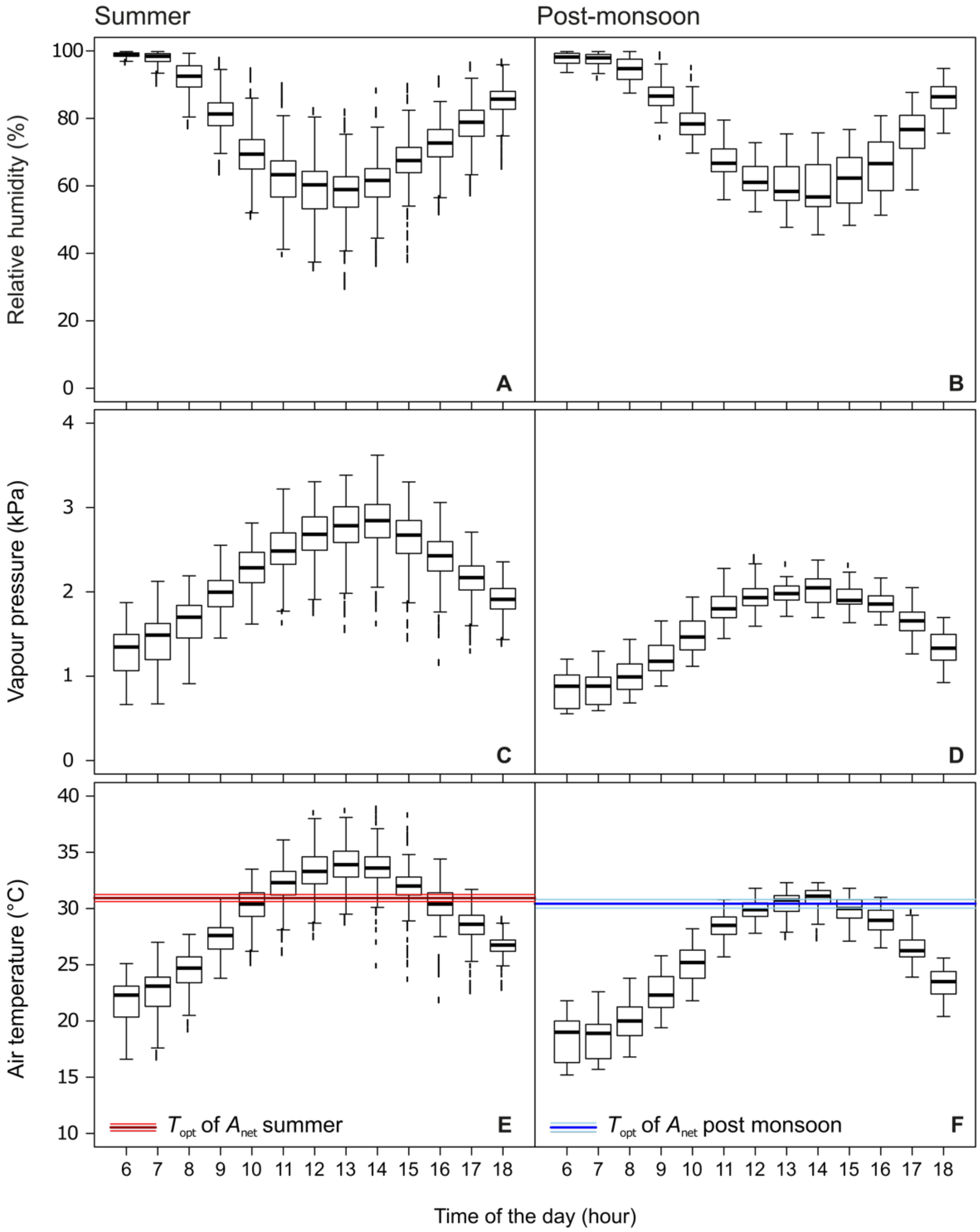

Diurnal changes in relative humidity (Panels A and B), vapour pressure deficit (Panels C and D) and air temperature (Panels E and F) over the two seasons at the study site.

Supplementary Section 2.

Significant fits are marked bold

Species

(9 tree species)

Parameter	Species	Season	Species: Season	Pseudo R^2
T_{opt}	F_8 = 8.2, **p <.0001**	F_1 = 3.26, p = 0.08	F_8 = 1.82, p = 0.11	0.61
A_{opt}	F_8 = 24.9, **p <.0001**	F_1 = 0.003, p =0.95	F_8 = 8.9, p **<.0001**	0.82
g_s at T_{opt}	F_8 = 23.9, **p <.0001**	F_1 = 7.8, **p =0.008**	F_8 = 13.4, **p <.0001**	0.84
E at T_{opt}	F_8 = 11.1, **p <.0001**	F_1 = 6.4, **p =0.016**	F_8 = 6.4, **p <.0001**	0.72
VPD at T_{opt}	F_8 = 2.8, **p =0.016**	F_1 = 0.36, p =0.55	F_8 = 1.9, p =0.08	0.40
A/g_s at T_{opt}	F_8 = 3.7, **p =0.0031**	F_1 = 0.16, p =0.68	F_8 = 3.7, **p =0.003**	0.51
A/E at T_{opt}	F_8 = 2.3, **p =0.03**	F_1 = 0.0001, p =0.99	F_8 = 3.19, **p=0.008**	0.44

Leaf habit

(3 deciduous and 6 evergreen species)

Parameter	Leaf habit	Season	Leaf habit: Season	Pseudo R^2
T_{opt}	F_1 = 0.38, p =0.53	F_1 = 0.90, p = 0.34	F_1 = 0.07, p = 0.78	0.02
A_{opt}	F_1 = 13.7, **p =0.0005**	F_1 = 0.003, p =0.95	F_1 = 10.1, **p =0.002**	0.29
g_s at T_{opt}	F_1 = 17.14, **p=0.0001**	F_1 = 2.3, p =0.13	F_1 = 23.5, **p <.0001**	0.43
E at T_{opt}	F_1 = 11.5, **p =0.0014**	F_1 = 3.1, p =0.08	F_1 = 17.5, **p=0.0001**	0.36
VPD at T_{opt}	F_1 = 1.8, p =0.18	F_1 = 0.15, p =0.69	F_1 = 0.006, p =0.93	0.03
A/g_s at T_{opt}	F_1 = 1.6, p =0.21	F_1 = 0.21, p =0.64	F_1 = 9.1, **p =0.004**	0.16
A/E at T_{opt}	F_1 = 0.33, p =0.56	F_1 = 0.003, p =0.95	F_1 = 5.4, **p=0.02**	0.09

Species: only evergreens (6 species)

Parameter	Species	Season	Species: Season	Pseudo R^2
T_{opt}	F_5 = 8.9, **p=0.0001**	F_1 = 1.67, p = 0.21	F_5 = 1.73, p = 0.17	0.61
A_{opt}	F_5 = 25.8, **p <.0001**	F_1 = 17.1, **p =0.0004**	F_5 = 2.46, p =0.06	0.81
g_s at T_{opt}	F_5 = 20.9, **p <.0001**	F_1 = 11.1, **p =0.003**	F_5 = 2.1, p=0.11	0.77
E at T_{opt}	F_5 = 11.1, **p <.0001**	F_1 = 3.5, p =0.07	F_5 = 1.8, p =0.15	0.66
VPD at T_{opt}	F_5 = 4.1, **p =0.009**	F_1 = 0.37, p =0.54	F_5 = 3.5, **p =0.01**	0.51
A/g_s at T_{opt}	F_5 = 3.63, **p =0.015**	F_1 = 2.8, p =0.11	F_5 = 1.3, p =0.29	0.42
A/E at T_{opt}	F_5 = 2.56, p =0.056	F_1 = 1.96, p =0.18	F_5 = 2.1, p=0.1	0.41

Species: only deciduous (3 species)

Parameter	Species	Season	Species: Season	Pseudo R^2
T_{opt}	F_2 = 9.5, p=0.0077	F_1 = 3.44, p = 0.10	F_2 = 5.1, p = 0.04	0.67
A_{opt}	F_2 = 11.1, p=0.0029	F_1 = 25.8, p = 0.0005	F_2 = 6.03, p = 0.02	0.77
g_s at T_{opt}	F_2 = 12.8, p =0.0017	F_1 = 88.4, p <0.001	F_2 = 1.6, p=0.24	0.86
E at T_{opt}	F_2 = 4.6, p =0.037	F_1 = 34.4, p =0.0002	F_2 = 0.38, p =0.69	0.71
VPD at T_{opt}	F_2 = 0.88, p =0.44	F_1 = 0.04, p =0.84	F_2 = 0.07, p =0.92	0.09
A/g_s at T_{opt}	F_2 = 4.51, p =0.04	F_1 = 48.1, p <0.001	F_2 = 9.6, p =0.004	0.80
A/E at T_{opt}	F_2 = 1.5, p =0.27	F_1 = 25.5, p =0.0005	F_2 = 6.1, p=0.018	0.69

Supplementary Figure 3.

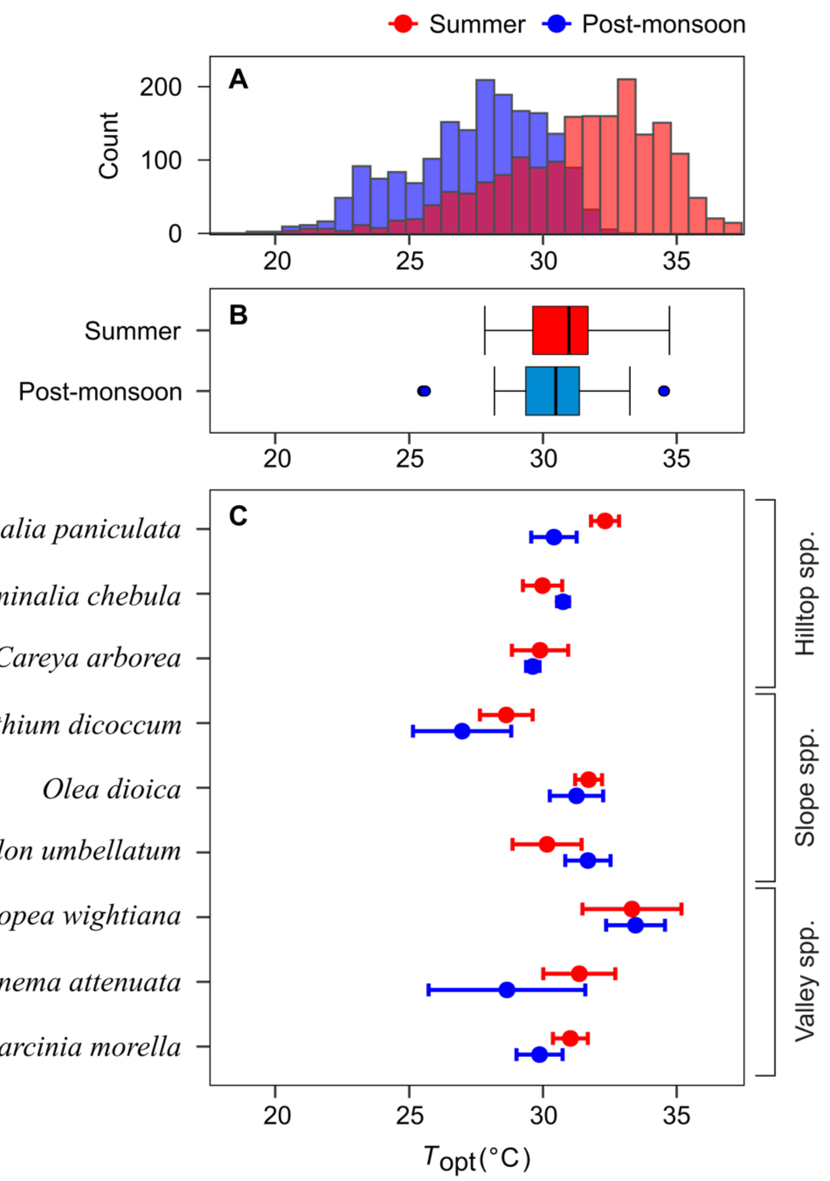

Thermal optima (T_{opt}) CO_2 assimilation rate for tree species in the Western Ghats, India measured *in situ*, *in natura* during the post-monsoon period, 2020 and peak summer period, 2021. Panel A shows a histogram of daytime air temperature during the two periods measured for 6 weeks at 10-minute intervals. Panel B shows aggregate species aggregate T_{opt} across the two seasons while panel C show species means ± SE.

Supplementary Figure 4.

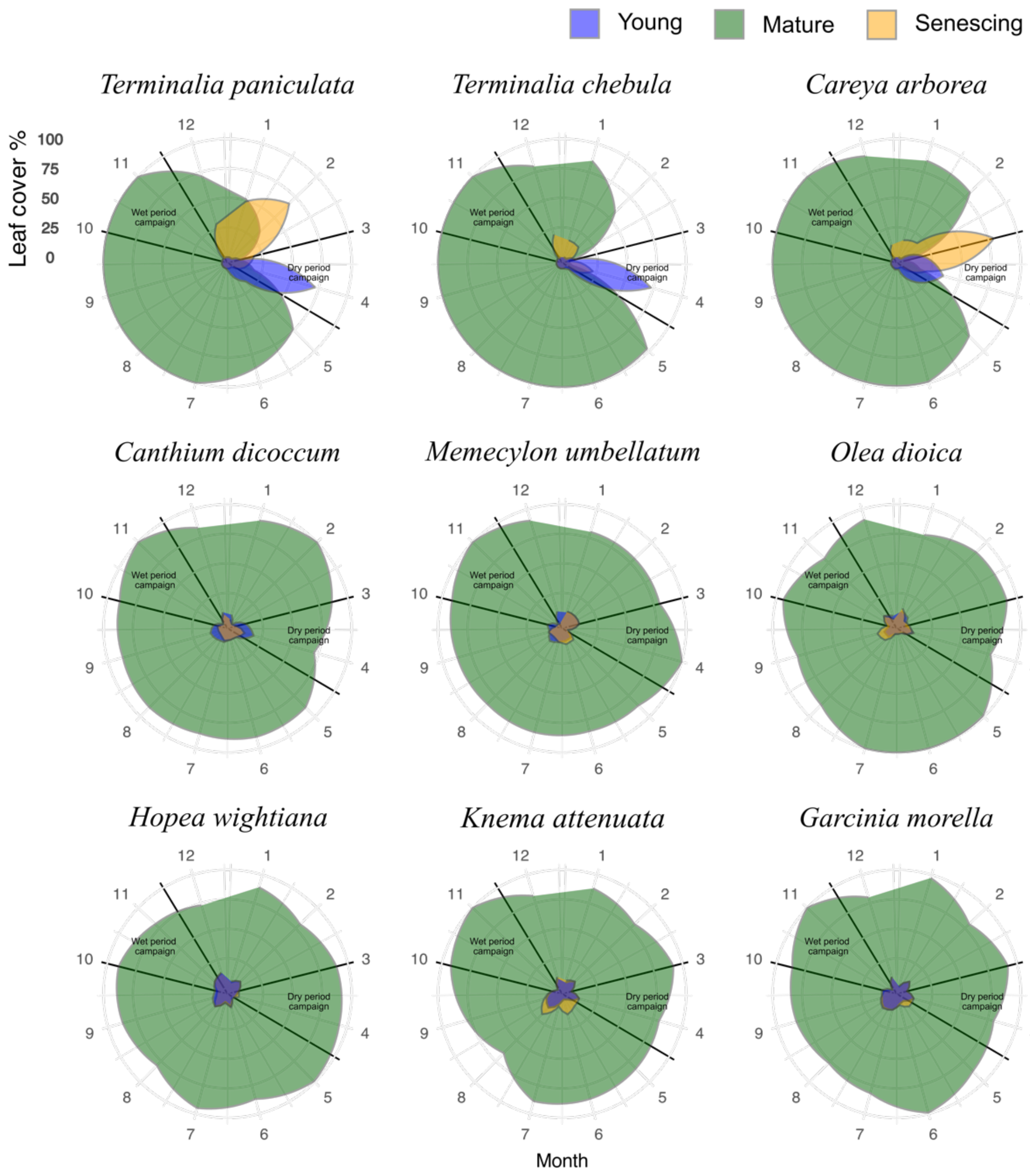

Leaf phenological cycles for the nine tree species were recorded through monthly field observations of canopy cover and the percentage of leaves at different stages. Each panel represents data for one species. Photosynthesis measurement campaigns are shown in vertical lines. In both periods, fully open and mature leaves were subjected to measurement.
