# Supplementary material for: Contrasting Seasonal Variation of Photosynthesis in Evergreen and Deciduous Tree Species From a Tropical Forest
Source: Physiol Plant. 2025 Jul 14;177(4):e70410. doi: 10.1111/ppl.70410 (PMC12257110; doi:10.1111/ppl.70410)
Supplement: Supplementary file 1 — Data S1: Supporting Information. [file PPL-177-e70410-s001.zip › 3_Supplementary-Section-02-Kannada-Language version.pdf]

The Kannada language version of the original peer reviewed manuscript Tiwari et al., (2025) in Physiologia Plantarum. This section has not been reviewed and is only provided as supplementary material. Please refer to the English version of the manuscript for the peer-reviewed version (DOI: 10.1111/ppl.70410). Kannada language version was written by Rakesh Tiwari and Somashekhara Achar KG. Refer to the glossary of technical terms at the end of this document for original English terms and corresponding Kannada terms used.

ಉಷ್ಣವಲಯ ಅರಣ್ಯದ ನಿತ್ಯಹರಿದ್ವರ್ಣ ಮತ್ತು ಪರ್ಣಪಾತಿ ವೃಕ್ಷ ಪ್ರಭೇಧಗಳಲ್ಲಿ ದ್ಯುತಿಸಂಶ್ಲೇಷಣೆಯ ಋತುಮಾನದ ವ್ಯತಿರಿಕ್ತ ವ್ಯತ್ಯಾಸಗಳು

Rakesh Tiwari[1, 2, 3]* | Balachandra Hegde[3,4] | Shrihari Hegde[5,3] | Peddiraju Bandaru[6] | Ramesh Babu M[7] | Somashekhara Achar KG[8,9] | Caroline Greiser[10] | Robert Muscarella[2] | Deepak Barua[6] | David Galbraith[1] | Emanuel Gloor[1]

[1] School of Geography, University of Leeds, United Kingdom
[2] Plant Ecology and Evolution, Institute of Ecology and Genetics, Uppsala Universitet, Sweden
[3] Sahyadri Ecological Observatory, Sirsi, India
[4] Department of Environmental Science, Kuvempu University, India
[5] Mangalore University, India
[6] Indian Institute of Science Education and Research, Pune, India
[7] Department of Electronic Media, Bangalore University, Bengaluru, India
[8] IDSG Government College, Chikkamagaluru, India
[9] Panchavati Research Academy for Nature, India
[10] Department of Physical Geography, Stockholm University, Sweden

* Corresponding author

ಸಂಕ್ಷೇಪ

ಜಲ ಲಭ್ಯತೆಯಲ್ಲಿನ ಸೂಕ್ಷ್ಮ ವಾತಾವರಣ ವ್ಯತ್ಯಾಸಗಳು ಸಹ-ಸಂಭವಿಸುವ ಉಷ್ಣವಲಯದ ವೃಕ್ಷ ಪ್ರಭೇದಗಳಲ್ಲಿ, ವಿಶೇಷವಾಗಿ ಬಲವಾದ ಋತು ಅವಲಂಬಿತ ವಾತಾವರಣ ಹೊಂದಿರುವ ಕಾಡುಗಳಲ್ಲಿ, ಋತು ಅವಲಂಬಿತ ನೀರಿನ ಬಳಕೆ ಮತ್ತು ದ್ಯುತಿಸಂಶ್ಲೇಷಕ ವ್ಯತ್ಯಾಸಕ್ಕೆ ಕಾರಣವಾಗಬಹುದು. ಭಾರತದ ಪಶ್ಚಿಮ ಘಟ್ಟಗಳಲ್ಲಿನ ಉಷ್ಣವಲಯದ ಅರಣ್ಯ ತಾಣವನ್ನು ನಾವು ಅಧ್ಯಯನ ಮಾಡಿದ್ದೇವೆ ಮತ್ತು ವ್ಯತಿರಿಕ್ತ ಪರ್ಣ ಸ್ವಭಾವ ಮತ್ತು ಸ್ಥಳಾಕೃತಿ ಸಂಬಂಧಗಳನ್ನು ಹೊಂದಿರುವ ಒಂಬತ್ತು ವೃಕ್ಷ ಪ್ರಭೇದಗಳಲ್ಲಿ ದ್ಯುತಿಸಂಶ್ಲೇಷಕ CO_2 ಉಪಗ್ರಹಣ ದರಗಳಲ್ಲಿನ (A_{net}) ಋತು ಅವಲಂಬಿತ ವ್ಯತ್ಯಾಸಗಳನ್ನು ನಿರೂಪಿಸಿದ್ದೇವೆ: ಶುಷ್ಕ ಬೆಟ್ಟದ ತುದಿಗಳಲ್ಲಿ ಪರ್ಣಪಾತಿ ಪ್ರಭೇದಗಳು, ಇಳಿಜಾರುಗಳಲ್ಲಿ ಶುಷ್ಕಪ್ರಿಯ ನಿತ್ಯಹರಿದ್ವರ್ಣ ವೃಕ್ಷಗಳು ಮತ್ತು ಕಣಿವೆಗಳಲ್ಲಿ ಆರ್ದ್ರ-ಪ್ರಿಯ ನಿತ್ಯಹರಿದ್ವರ್ಣ ವೃಕ್ಷಗಳು. ಮೇಲ್ಮೈ ಮಣ್ಣಿನ ತೇವಾಂಶವು ಬೆಟ್ಟದ ತುದಿಗಳಲ್ಲಿ ಕಡಿಮೆ, ಇಳಿಜಾರುಗಳಲ್ಲಿ ಮಧ್ಯಮ ಮತ್ತು ಕಣಿವೆಗಳಲ್ಲಿ ಅತ್ಯಧಿಕವಾಗಿತ್ತು, ಶುಷ್ಕ ಅವಧಿಗೆ ಹೋಲಿಸಿದರೆ ಆರ್ದ್ರ ಅವಧಿಯಲ್ಲಿ ಹೆಚ್ಚಿನ ಮಟ್ಟಗಳು ಕಂಡುಬಂದಿವೆ. ನಿರೀಕ್ಷೆಯಂತೆ, ಶುಷ್ಕ ಬೆಟ್ಟದ ಶಿಖರಗಳಲ್ಲಿನ ಪರ್ಣಪಾತಿ ಪ್ರಭೇದಗಳು ಆರ್ದ್ರ ಅವಧಿಯಲ್ಲಿ ಆದರ್ಶ ತಾಪಮಾನದಲ್ಲಿ (T_{opt}) ಹೆಚ್ಚಿನ ದ್ಯುತಿಸಂಶ್ಲೇಷಕ ದರವನ್ನು ತೋರಿಸಿದವು, ಆದರೆ ನಿತ್ಯಹರಿದ್ವರ್ಣ ಪ್ರಭೇದಗಳು ಒಟ್ಟಾರೆ ಋತು ಅವಲಂಬಿತ ವ್ಯತ್ಯಾಸಗಳನ್ನು ತೋರಿಸಲಿಲ್ಲ. ಕುತೂಹಲಕಾರಿಯಾಗಿ, ಶುಷ್ಕಪ್ರಿಯ ಇಳಿಜಾರಿನ ನಿತ್ಯಹರಿದ್ವರ್ಣ ಪ್ರಭೇದಗಳು ಆರ್ದ್ರ ಅವಧಿಗೆ ಹೋಲಿಸಿದರೆ ಶುಷ್ಕ ಅವಧಿಯಲ್ಲಿ ಆದರ್ಶ ತಾಪಮಾನದಲ್ಲಿ ಹೆಚ್ಚಿನ A_{net} ಅನ್ನು ತೋರಿಸಿದವು, ಇದು ಕಡಿಮೆ ಮಣ್ಣಿನ ತೇವಾಂಶದ ಹೊರತಾಗಿಯೂ, ಶುಷ್ಕ ಅವಧಿಯಲ್ಲಿ ಸಾಕಷ್ಟು ಜಲ ಲಭ್ಯತೆ ಮತ್ತು ಈ ಪ್ರಭೇದಗಳಲ್ಲಿ ಉಷ್ಣತರ ತಾಪ ಗುಣ ವಿಶಿಷ್ಟ ಕ್ಷೇತ್ರ ಆದ್ಯತೆ ಅಥವಾ ಉಷ್ಣ ರೂಪಾಂತರವನ್ನು ಸೂಚಿಸುತ್ತದೆ. ಎಲ್ಲಾ ಪ್ರಭೇದಗಳಲ್ಲಿ, T_{opt} ನಲ್ಲಿ ಒಂದು ನಿತ್ಯಹರಿದ್ವರ್ಣ ಪ್ರಭೇದವನ್ನು ಹೊರತುಪಡಿಸಿ, ಮಳೆಗಾಲದಲ್ಲಿ ಪತ್ರರಂಧ್ರ ವಾಹಕತೆಯು (g_s) ಸಾಮಾನ್ಯವಾಗಿ ಹೆಚ್ಚಿತ್ತು. ನಮ್ಮ ಸಂಶೋಧನೆಗಳು ವಿಭಿನ್ನ ಪರ್ಣ ಸ್ವಭಾವ ಮತ್ತು ಸ್ಥಳಾಕೃತಿ ಸಂಬಂಧಗಳಲ್ಲಿ ಉಷ್ಣವಲಯದ ವೃಕ್ಷ ಪ್ರಭೇದಗಳಲ್ಲಿ ದ್ಯುತಿಸಂಶ್ಲೇಷಣೆಯಲ್ಲಿನ ವ್ಯತ್ಯಾಸಗಳನ್ನು ವಿವರಿಸುತ್ತದೆ.

ಪ್ರಮುಖ ಪದಗಳು: ದ್ಯುತಿಸಂಶ್ಲೇಷಣೆ, ಉಷ್ಣವಲಯದ ಅರಣ್ಯ, ವೃಕ್ಷಗಳು, ಋತು ಅವಲಂಬಿತ ಸಮಾಯೋಜ್ಯ, ಪತ್ರರಂಧ್ರ ವಾಹಕತೆ, ಋತು-ಧರ್ಮ, ಸ್ಥಳಾಕೃತಿ, ಮಣ್ಣಿನ ತೇವಾಂಶ, ಪಶ್ಚಿಮ ಘಟ್ಟಗಳು

೧ | ಪ್ರಸ್ತಾವನೆ

ಜಾಗತಿಕವಾಗಿ, ಉಷ್ಣವಲಯದ ಕಾಡುಗಳು ವಿವಿಧ ಋತುಚಕ್ರ ಪದ್ಧತಿಗಳನ್ನು ಪ್ರದರ್ಶಿಸುತ್ತವೆ, ಅವು ಋತುಮಾನ ರಹಿತ ದಿಂದ ಬಲವಾದ ಋತು ಅವಲಂಬಿತ ವಾತಾವರಣದವರೆಗೆ ವಿಭಿನ್ನ ಆರ್ದ್ರ ಮತ್ತು ಶುಷ್ಕ ಅವಧಿಗಳೊಂದಿಗೆ ಇರುತ್ತವೆ (Carvalho et al. 2021). ಶುಷ್ಕ ಉಷ್ಣವಲಯದ ಕಾಡುಗಳು, ಋತುಮಾನಗಳಲ್ಲಿ ಜಲ ಲಭ್ಯತೆ ಮತ್ತು ವಾಯು ಉಷ್ಣತೆಯ ನಿರ್ದಿಷ್ಟ ವ್ಯತಿರಿಕ್ತ ಪರಿಸ್ಥಿತಿಗಳನ್ನು ಅನುಭವಿಸುತ್ತವೆ.

ಈ ಋತು ಅವಲಂಬನೆಯು ವೃಕ್ಷ ಪ್ರಭೇದಗಳ ನಡುವಿನ ಕ್ರಿಯಾಪ್ರಕ್ರಿಯೆಗಳ ಮೇಲೆ ಪ್ರಭಾವ ಬೀರುತ್ತದೆ, ಇದು ಪರ್ಣ ಸ್ವಭಾವ ಮತ್ತು ನೀರಿನ ಸಂಬಂಧಗಳಲ್ಲಿ ವ್ಯತ್ಯಾಸವನ್ನು ಪ್ರದರ್ಶಿಸುತ್ತದೆ (Rey-Sánchez et al. 2016). ಇದಲ್ಲದೆ, ವೃಕ್ಷ ಪ್ರಭೇದಗಳಲ್ಲಿ ಸ್ಥಳಾಕೃತಿ ಮತ್ತು ಸ್ಥಾನಿಕ ವಿತರಣೆ ಎಲೆ ಋತುಧರ್ಮದಂತಹ ಸೂಕ್ಷ್ಮ ವಾತಾವರಣ ಅಂಶಗಳು (Zhang et al. 2023), ಸಸ್ಯವರ್ಗದ ಮೇಲೆ ಋತು ಅವಲಂಬಿತ ವ್ಯತ್ಯಾಸಗಳ ಮಾರ್ಪಡಿಸಬಹುದು (Schwartz et al. 2022). ಈ ಋತು ಅವಲಂಬಿತ ಶುಷ್ಕ ಉಷ್ಣವಲಯದ ಕಾಡುಗಳಲ್ಲಿ ಸಹವರ್ತಿ ವೃಕ್ಷ ಪ್ರಭೇದಗಳ ನಡುವಿನ ಕ್ರಿಯಾಪ್ರಕ್ರಿಯೆಗಳಲ್ಲಿನ ಋತು ಅವಲಂಬಿತ ವ್ಯತ್ಯಾಸದ ಉತ್ತಮ ತಿಳುವಳಿಕೆಯು ಬರ ಮತ್ತು ಭವಿಷ್ಯದ ತಾಪಮಾನ ಏರಿಕೆಗೆ ವೃಕ್ಷ ಪ್ರಭೇದಗಳ ಸೂಕ್ಷ್ಮತೆಯನ್ನು ಅರ್ಥಮಾಡಿಕೊಳ್ಳಲು ಸಹಾಯಕವಾಗುತ್ತದೆ (Köpp Hollunder et al. 2022). ಉದಾಹರಣೆಗೆ, ದ್ಯುತಿಸಂಶ್ಲೇಷಣೆಯಲ್ಲಿನ ಋತು ಅವಲಂಬಿತ ವ್ಯತ್ಯಾಸದ ವ್ಯಾಪ್ತಿ - ಪ್ರಾಥಮಿಕ ಇಂಗಾಲದ ಉಪಗ್ರಹಣ ಪ್ರಕ್ರಿಯೆ - ವಿಶೇಷವಾಗಿ ಋತು ಅವಲಂಬಿತ ವೈವಿಧ್ಯತೆ ಮತ್ತು ಸೂಕ್ಷ್ಮ ವಾತಾವರಣ ಪ್ರಭಾವಿತ ಜಲ ಲಭ್ಯತೆಯ ವ್ಯತ್ಯಾಸಗಳು ಮರಗಳಲ್ಲಿ ಹೇಗೆ ಭಿನ್ನವಾಗಿರುತ್ತದೆ ಎನ್ನುವುದು ಸ್ಪಷ್ಟವಾಗಿಲ್ಲ.

ದ್ಯುತಿಸಂಶ್ಲೇಷಣೆಯಲ್ಲಿನ ಋತುಮಾನದ ವ್ಯತ್ಯಾಸವು ವಾಯು, ಉಷ್ಣತೆ, ಜಲ ಲಭ್ಯತೆ, ಬೆಳಕಿನ ಮಟ್ಟಗಳು ಮತ್ತು ಹಗಲಿನ ಅವಧಿ ಅಥವಾ ದ್ಯುತಿ ಅವಧಿ ಸೇರಿದಂತೆ ಪರಿಸರ ಅಂಶಗಳ ನಡುವಿನ ಸಂಕೀರ್ಣ ಪರಸ್ಪರ ಕ್ರಿಯೆಗಳಿಂದ ನಡೆಸಲ್ಪಡುತ್ತದೆ (Yamaguchi et al. 2016). ಜೀವರಾಸಾಯನಿಕ ಕಾರ್ಯವಿಧಾನಗಳು ಸಹ ಒಂದು ಪಾತ್ರವನ್ನು ವಹಿಸುತ್ತವೆ, ಕಿಣ್ವ ಮತ್ತು ದ್ಯುತಿಸಂಶ್ಲೇಷಕ ಎಲೆಕ್ಟ್ರಾನ್ ಸಾಗಣೆ (Wada et al. 2023) ಮತ್ತು ಮೈಟೊಕಾಂಡ್ರಿಯದ ಉಸಿರಾಟ (Way and Yamori 2014) ದಲ್ಲಿನ ಹೊಂದಾಣಿಕೆಗಳನ್ನು ಒಳಗೊಂಡಿರುತ್ತದೆ. ಈ ಅಂಶಗಳು ಹೆಚ್ಚಾಗಿ ಎಲೆಯ ಸಾರಜನಕ ಅಂಶ, ವರ್ಣದ್ರವ್ಯಗಳು ಮತ್ತು ಎಲೆಯ ಋತು-ಧರ್ಮ ಪ್ರತಿಫಲಿಸುತ್ತದೆ (Yasumura et al. 2006; Muller et al. 2011). ಉದಾಹರಣೆಗೆ, ಎಲೆಯ ಸಾರಜನಕ ಅಂಶವು ಸಾಮಾನ್ಯವಾಗಿ ಆರಂಭಿಕ ಬೆಳವಣಿಗೆಯ ಹಂತಗಳಲ್ಲಿ ಗರಿಷ್ಠಗೊಳ್ಳುತ್ತದೆ ಮತ್ತು ವೃದ್ಧಾಪ್ಯದೊಂದಿಗೆ ಕಡಿಮೆಯಾಗುತ್ತದೆ (Joshi et al. 2024). ಅದೇ ರೀತಿ, ಎಲೆ ವರ್ಣದ್ರವ್ಯದ ಅಂಶ, ವಿಶೇಷವಾಗಿ ಕ್ಲೋರೊಫಿಲ್, ಎಲೆ ಸಾರಜನಕದ ಜೊತೆಗೆ ಗರಿಷ್ಠವಾಗಿರುತ್ತದೆ, ಆದರೆ ದ್ಯುತಿ-ರಕ್ಷಣೆಯಲ್ಲಿ ಪ್ರಮುಖ ಪಾತ್ರ ವಹಿಸುವ ಕ್ಯಾರೊಟಿನಾಯ್ಡ್‌ಗಳು ಸಣ್ಣ ಋತು ಅವಲಂಬಿತ ಬದಲಾವಣೆಗಳನ್ನು ತೋರಿಸುತ್ತವೆ (Shi et al. 2014; Peng et al. 2021; Wada et al. 2023). ಎಲೆಗಳು ತಮ್ಮ ಗರಿಷ್ಠ ಬೆಳವಣಿಗೆಯ ಅವಧಿಯನ್ನು ಮೀರಿ ವಯಸ್ಸಾದಂತೆ, ಎಲೆ ವರ್ಣದ್ರವ್ಯ ಮತ್ತು ಸಾರಜನಕ ಅಂಶದಲ್ಲಿನ ಕಡಿತದಿಂದಾಗಿ ದ್ಯುತಿಸಂಶ್ಲೇಷಕ ಸಾಮರ್ಥ್ಯವು ಕಡಿಮೆಯಾಗುತ್ತದೆ (Yasumura et al. 2006). ಸಹವರ್ತಿ ವೃಕ್ಷ ಪ್ರಭೇದಗಳಲ್ಲಿ, ವಿಶೇಷವಾಗಿ ಹೆಚ್ಚಿನ ಜೀವವೈವಿಧ್ಯತೆಯನ್ನು ಹೊಂದಿರುವ ಕಾಡುಗಳಲ್ಲಿ ವೈವಿಧ್ಯಮಯ ಋತು-ಧರ್ಮ (Corredor-Londoño et al. 2020; Devi et al. 2023; Wada et al. 2023) ದ್ಯುತಿಸಂಶ್ಲೇಷಣೆಯಲ್ಲಿ ವೈವಿಧ್ಯಮಯ ಋತು ಅವಲಂಬಿತ ಮಾದರಿಗಳಿಗೆ ಕಾರಣವಾಗಬಹುದು.

ದ್ಯುತಿಸಂಶ್ಲೇಷಣೆ ನಿಯತಾಂಕಗಳಲ್ಲಿನ ಋತು ಅವಲಂಬಿತ ವ್ಯತ್ಯಾಸಗಳು ಋತು ಅವಲಂಬಿತ ಪರಿಸರ ಬದಲಾವಣೆಗಳಿಗೆ ಒಗ್ಗಿಕೊಳ್ಳುವ ಪ್ರತಿಕ್ರಿಯೆಗಳನ್ನು ಸಹ ಸೂಚಿಸುತ್ತವೆ (Wittemann et al. 2022). ಒಗ್ಗಿಕೊಳ್ಳುವಿಕೆಯು ಶಾರೀರಿಕ, ರಚನಾತ್ಮಕ ಅಥವಾ ಜೀವರಾಸಾಯನಿಕ ಹೊಂದಾಣಿಕೆಗಳನ್ನು ಒಳಗೊಂಡಿರುತ್ತದೆ, ಇದು ದ್ಯುತಿಸಂಶ್ಲೇಷಣೆಯ ಆದರ್ಶ ತಾಪಮಾನ (T_{opt}), ಆದರ್ಶ ತಾಪಮಾನದಲ್ಲಿ ದ್ಯುತಿಸಂಶ್ಲೇಷಣೆ ದರ (A_{opt}) ಮತ್ತು ಗರಿಷ್ಠ RuBP ಕಾರ್ಬಾಕ್ಸಿಲೇಷನ್ ದರಗಳಲ್ಲಿ ಬದಲಾವಣೆಗಳನ್ನು ಉಂಟುಮಾಡುತ್ತದೆ. ಉಷ್ಣ ಆದರ್ಶ ತಾಪಮಾನ ದಲ್ಲಿನ ಬದಲಾವಣೆಗಳು ರುಬಿಸ್ಕೊ ಕಾರ್ಬಾಕ್ಸಿಲೇಷನ್ ಸಕ್ರಿಯಗೊಳಿಸುವ ಶಕ್ತಿಯಲ್ಲಿನ ಬದಲಾವಣೆಗಳೊಂದಿಗೆ (Hikosaka 2005; Borjigidai et al. 2006) ಮತ್ತು ವಿಭಿನ್ನ ದ್ಯುತಿಸಂಶ್ಲೇಷಕ ಮಾರ್ಗಗಳಲ್ಲಿ ಇತರ ತಾಪಮಾನ-ಅವಲಂಬಿತ ಪ್ರಕ್ರಿಯೆಗಳು ಸಂಬಂಧ ಹೊಂದಿವೆ (Yamori et al. 2014). ಸಾಮಾನ್ಯವಾಗಿ, ಉಷ್ಣವಲಯದ ವೃಕ್ಷ ಪ್ರಭೇದಗಳು ತಮ್ಮ ದ್ಯುತಿಸಂಶ್ಲೇಷಣೆಯನ್ನು ಮಧ್ಯಮ ತಾಪಮಾನಕ್ಕೆ ಸಮಶೀತೋಷ್ಣ ಅಥವಾ ಬೋರಿಯಲ್ ಪ್ರಭೇದಗಳಿಗಿಂತ ಕಡಿಮೆ ಪ್ರಮಾಣದಲ್ಲಿ ಒಗ್ಗಿಕೊಳ್ಳುತ್ತವೆ, (Slot and Winter 2017a; Wittemann et al. 2022; Liu et al. 2024). ಒಗ್ಗಿಕೊಳ್ಳುವಿಕೆಯ ವ್ಯಾಪ್ತಿಯು ಪ್ರಭೇದಗಳಲ್ಲಿ ಬದಲಾಗುತ್ತದೆ; ಉದಾಹರಣೆಗೆ, ಕಡಿಮೆ ಎತ್ತರದ ಪ್ರಭೇದಗಳು ಮಲೆನಾಡಿನ ಪ್ರಭೇದಗಳಿಗಿಂತ ತಾಪಮಾನ ಹೆಚ್ಚಳಕ್ಕೆ ಹೆಚ್ಚು ಬಲವಾಗಿ ಒಗ್ಗಿಕೊಳ್ಳುತ್ತವೆ (Wittemann et al. 2022). ಸಾಮಾನ್ಯವಾಗಿ, ಹೆಚ್ಚಿನ ಬೆಳವಣಿಗೆಯ ತಾಪಮಾನದೊಂದಿಗೆ ದ್ಯುತಿಸಂಶ್ಲೇಷಕ ಆದರ್ಶ ತಾಪಮಾನ ಹೆಚ್ಚಳ (Yamasaki et al. 2002; Hikosaka 2005; Choury et al. 2022). T_{opt} ನ ಋತು ಅವಲಂಬಿತ ಒಗ್ಗಿಕೊಳ್ಳುವಿಕೆಯು ವರ್ಷವಿಡೀ ದ್ಯುತಿಸಂಶ್ಲೇಷಕ ದಕ್ಷತೆಯನ್ನು ಗರಿಷ್ಠಗೊಳಿಸಲು ಅಥವಾ ನಿರ್ವಹಿಸಲು ಪ್ರಭೇದಗಳ ತಂತ್ರವನ್ನು ಸೂಚಿಸುತ್ತದೆ (Kattge and Knorr 2007), ಇದರ ಬಗೆಗಿನ ಹೆಚ್ಚಿನ ಜ್ಞಾನವನ್ನು ಬೆಳೆಗಳ ಅಥವಾ ಸಸಿಗಳ ಮೇಲಿನ ಅಧ್ಯಯನಗಳಿಂದ ಪಡೆಯಲಾಗಿದೆ (Gjindali et al. 2021; Gjindali and Johnson 2023). ಆದ್ದರಿಂದ, ಋತು ಅವಲಂಬಿತ ಪರಿಸರ ವ್ಯತ್ಯಾಸಗಳೊಂದಿಗೆ ಉಷ್ಣವಲಯದ ಕಾಡುಗಳಲ್ಲಿನ ವಯಸ್ಕ ಮರಗಳ ಮೇಲಿನ ಸಂಶೋಧನೆಯಲ್ಲಿ ಗಮನಾರ್ಹ ಅಂತರವಿದೆ.

ಋತುಮಾನದ ಅಂತರಗಳನ್ನು ನಿರೂಪಿಸುವ ಎಲೆ-ಮಟ್ಟದ ಅಧ್ಯಯನಗಳು ಸಾಮಾನ್ಯವಾಗಿ ಋತುಮಾನದ ಶುಷ್ಕ ಅವಧಿಯಲ್ಲಿ, ವಿಶೇಷವಾಗಿ ಪರ್ಣಪಾತಿ ಪ್ರಭೇದಗಳಲ್ಲಿ ದ್ಯುತಿಸಂಶ್ಲೇಷಣೆ ದರಗಳಲ್ಲಿನ ಕುಸಿತವನ್ನು ವರದಿ ಮಾಡುತ್ತವೆ (Eamus et al. 1999; Zhang et al. 2007). ಪತ್ರ ಋತುಧರ್ಮ ಮತ್ತು ಜಲ ಲಭ್ಯತೆಯನ್ನು ಹೆಚ್ಚಾಗಿ ಈ ಋತುಮಾನದ ವ್ಯತ್ಯಾಸಗಳನ್ನು ವಿವರಿಸುವ ಪ್ರಮುಖ ಅಂಶಗಳಾಗಿ ಗುರುತಿಸಲಾಗುತ್ತದೆ (Eamus et al. 1999; Zhang et al. 2007). ಉದಾಹರಣೆಗೆ, ಪನಾಮದಲ್ಲಿನ ಅಧ್ಯಯನಗಳು ಮರದ ಸಸಿಗಳಲ್ಲಿ ಶುಷ್ಕ ಋತುವಿನಲ್ಲಿ ಕಡಿಮೆಯಾದ ದ್ಯುತಿಸಂಶ್ಲೇಷಣೆ ದರಗಳನ್ನು ಕಂಡುಕೊಂಡಿವೆ (Craven et al. 2011). ಆಸ್ಟ್ರೇಲಿಯನ್ ಅಕೇಶಿಯಾ ಪ್ರಭೇದ ಸಸಿಗಳಲ್ಲಿ (Montagu and Woo 1999) ಇದೇ ರೀತಿಯ ಶುಷ್ಕ-ಅವಧಿಯ ಕುಸಿತಗಳನ್ನು ಮಾಪನ ಮಾಡಲಾಗಿದೆ. ಥೈಲ್ಯಾಂಡ್‌ನ ಉಷ್ಣವಲಯದಲ್ಲಿ ನಡೆಸಿದ ವಯಸ್ಕ ಮರಗಳ ಎಲೆ-ಮಟ್ಟದ ಮೂಲ ಸ್ಥಾನಿಕ ಮಾಪನಗಳು ಒಂದು ನಿತ್ಯಹರಿದ್ವರ್ಣ ಪ್ರಭೇದಕ್ಕೆ ವರ್ಷವಿಡೀ ಸ್ಥಿರವಾದ ದ್ಯುತಿಸಂಶ್ಲೇಷಣೆ ದರಗಳನ್ನು ತೋರಿಸಿದವು, ಆದರೆ ಎರಡು ಇತರ ನಿತ್ಯಹರಿದ್ವರ್ಣ ಪ್ರಭೇದಗಳು ಶುಷ್ಕ ಋತುವಿನಲ್ಲಿ ಕುಸಿತವನ್ನು ಪ್ರದರ್ಶಿಸಿದವು (Ishida et al. 2006). ಅದೇ ರೀತಿ, ಅಮೆಜೋನಿಯನ್

ಕಾಡಿನಲ್ಲಿ ನಡೆಸಿದ ಅಧ್ಯಯನವು ಶುಷ್ಕ ಋತುವಿನಲ್ಲಿ ಮೇಲಾವರಣ ಮರಗಳಲ್ಲಿ ದ್ಯುತಿಸಂಶ್ಲೇಷಣೆ ದರದಲ್ಲಿ ಇಳಿಕೆ ಕಂಡುಬಂದಿದೆ, ಆದರೆ ಅದೇ ಅವಧಿಯಲ್ಲಿ ಅಧೋವಿತಾನ ಪ್ರಭೇದಗಳ ದ್ಯುತಿಸಂಶ್ಲೇಷಣೆ ದರಗಳು ಸ್ವಲ್ಪ ಕಡಿಮೆಯಾಗಿದೆ (Santos et al. 2018). ಹೀಗಾಗಿ, ದ್ಯುತಿಸಂಶ್ಲೇಷಣೆ ದರದಲ್ಲಿ ಶುಷ್ಕ ಅವಧಿಯ ಕಡಿತವನ್ನು ಸಾಮಾನ್ಯವಾಗಿ ವರದಿ ಮಾಡಲಾಗುತ್ತದೆ. ಇದಕ್ಕೆ ವ್ಯತಿರಿಕ್ತವಾಗಿ, ಕೆಲವು ಅಧ್ಯಯನಗಳು ಬೇಸಿಗೆಯಲ್ಲಿ ಪರ್ಣಪಾತಿ ವೃಕ್ಷ ಪ್ರಭೇದಗಳಲ್ಲಿ (Naidu and Swamy 1995) ಮತ್ತು ಭಾರತದಲ್ಲಿನ ಅರೆ-ಪರ್ಣಪಾತಿ, ಪರ್ಣಪಾತಿ ಮತ್ತು ನಿತ್ಯಹರಿದ್ವರ್ಣ ವೃಕ್ಷ ಪ್ರಭೇದಗಳ ಸಸಿಗಳಲ್ಲಿ (Abhilash and Devakumar 2023) ಹೆಚ್ಚಿನ ದ್ಯುತಿಸಂಶ್ಲೇಷಣೆ ದರಗಳನ್ನು ವರದಿ ಮಾಡಿವೆ. ಈ ವೈವಿಧ್ಯ ಪ್ರತಿಕ್ರಿಯೆಗಳು ಸೂಕ್ಷ್ಮ ವಾತಾವರಣಗಳಂತಹ ಇತರ ಪ್ರಾದೇಶಿಕ ಅಂಶಗಳು ದ್ಯುತಿಸಂಶ್ಲೇಷಣೆಯಲ್ಲಿನ ಋತು ಅವಲಂಬಿತ ವ್ಯತ್ಯಾಸಗಳ ಮೇಲೆ ಪ್ರಭಾವ ಬೀರಬಹುದು ಎಂದು ಸೂಚಿಸುತ್ತವೆ - ವಿಶೇಷವಾಗಿ ಋತು ಅವಲಂಬಿತವಾದ ಶುಷ್ಕ ಉಷ್ಣವಲಯದ ಕಾಡುಗಳಲ್ಲಿ.

ದ್ಯುತಿಸಂಶ್ಲೇಷಣೆ ದರಗಳಲ್ಲಿನ ಋತು ಅವಲಂಬಿತ ವ್ಯತ್ಯಾಸಗಳ ಜೊತೆಗೆ, ಪತ್ರ-ರಂಧ್ರ ವಾಹಕತೆ (g_s) ಯಂತಹ ಅನಿಲ ವಿನಿಮಯದ ಇತರ ಅಂಶಗಳು ಋತು ಅವಲಂಬಿತ ವ್ಯತ್ಯಾಸವನ್ನು ಪ್ರದರ್ಶಿಸಬಹುದು. ಸಾಮಾನ್ಯ ವಾತಾವರಣದ ಪರಿಸ್ಥಿತಿಗಳಲ್ಲಿ, ಪತ್ರ-ರಂಧ್ರ ವಾಹಕತೆ ದ್ಯುತಿಸಂಶ್ಲೇಷಣೆ ದರಗಳೊಂದಿಗೆ ನಿಕಟ ಸಂಬಂಧ ಹೊಂದಿರುತ್ತದೆ (Slot and Winter 2017b). ಆದಾಗ್ಯೂ, ಹೆಚ್ಚಿನ ತಾಪಮಾನದಲ್ಲಿ, g_s ಪ್ರತಿಕ್ರಿಯೆಗಳು ಹೆಚ್ಚಾಗಿ ದ್ಯುತಿಸಂಶ್ಲೇಷಣೆಯಿಂದ ಭಿನ್ನವಾಗಿರುತ್ತದೆ (Asargew et al. 2024). ಪತ್ರ-ರಂಧ್ರ ವಾಹಕತೆಯ ತಾಪಮಾನ ಪ್ರತಿಕ್ರಿಯೆಯ ಆಕಾರವು ಬದಲಾಗುತ್ತದೆ. g_s ತಾಪಮಾನದಲ್ಲಿ ರೇಖೀಯ ಇಳಿಕೆ ಕಂಡುಬಂದರೂ (Urban et al. 2017; Eze et al. 2024), ಕೆಲವು ವರದಿಗಳು ಹೆಚ್ಚಿನ ತಾಪಮಾನದಲ್ಲಿ g_s ಹೆಚ್ಚಳವನ್ನು ಮತ್ತು ಕೆಲವು ಸಂದರ್ಭಗಳಲ್ಲಿ ಗರಿಷ್ಠ ಪ್ರತಿಕ್ರಿಯೆಯನ್ನು ಸೂಚಿಸುತ್ತವೆ (Yamori et al. 2006; Hernández et al. 2020). ಸಾಮಾನ್ಯವಾಗಿ, ಪರ್ಣಪಾತಿ ಮರಗಳಲ್ಲಿ ಆರ್ದ್ರ ಅವಧಿಯಲ್ಲಿ g_s ಹೆಚ್ಚಾಗಿರುತ್ತದೆ (Grace et al. 1982), ಆದರೆ ಕೆಲವು ಅಧ್ಯಯನಗಳು ನಿತ್ಯಹರಿದ್ವರ್ಣ ಪ್ರಭೇದಗಳ g_s ಗಮನಾರ್ಹವಾಗಿ ಬದಲಾಗುವುದಿಲ್ಲ ಎಂದು ತೋರಿಸುತ್ತವೆ (Andriyas et al. 2021). ಆದಾಗ್ಯೂ, ಋತು ಅವಲಂಬಿತ ಉಷ್ಣವಲಯದ ಕಾಡುಗಳಲ್ಲಿನ ಮರಗಳು, ವಿಶೇಷವಾಗಿ ವಯಸ್ಕ ಮರಗಳು, ವಿಶೇಷವಾಗಿ ಶುಷ್ಕ ಪರಿಸ್ಥಿತಿಗಳಲ್ಲಿ ಅವುಗಳ ಪತ್ರ-ರಂಧ್ರ ವಾಹಕತೆಯ ತಂತ್ರಗಳಲ್ಲಿ ಸಂಭಾವ್ಯವಾಗಿ ಭಿನ್ನವಾಗಿರಬಹುದು. ಈ ಋತು ಅವಲಂಬಿತ ವ್ಯತ್ಯಾಸವು ಶುಷ್ಕ ಮತ್ತು ಆರ್ದ್ರ ಅವಧಿಗಳಲ್ಲಿ ವಾಯು ಉಷ್ಣತೆ, ಆವಿಯ ಒತ್ತಡ ಮತ್ತು ಜಲ ಲಭ್ಯತೆ (Comita and Engelbrecht 2009; Schwartz et al. 2022), ಬೇರಿನ ಆಳ ಮತ್ತು ನೀರಿನ ಲಭ್ಯತೆ ವೈವಿಧ್ಯ (Stahl et al. 2013) ಮತ್ತು ಮಣ್ಣಿನ ಜಲ ಲಭ್ಯತೆ (Vourlitis et al. 2008; Schmitt et al. 2022) ವ್ಯತ್ಯಾಸಗಳಿಂದಾಗಿರಬಹುದು. ಹೀಗಾಗಿ, ಅಸ್ತಿತ್ವದಲ್ಲಿರುವ ಸಾಹಿತ್ಯವು ದ್ಯುತಿಸಂಶ್ಲೇಷಣೆಯಲ್ಲಿನ ಋತು ಅವಲಂಬಿತ ವ್ಯತ್ಯಾಸಗಳ ಕುರಿತು ಕೆಲವು ಒಳನೋಟಗಳನ್ನು ಒದಗಿಸಿದರೂ, ಋತು ಅವಲಂಬಿತವಾಗಿ ಶುಷ್ಕ ಉಷ್ಣವಲಯದ ಕಾಡಿನಲ್ಲಿ ವಯಸ್ಕ ಮರಗಳಲ್ಲಿ ದ್ಯುತಿಸಂಶ್ಲೇಷಣೆಯ ತಾಪಮಾನ ಪ್ರತಿಕ್ರಿಯೆಯಲ್ಲಿನ ಋತು ಅವಲಂಬಿತ ವ್ಯತ್ಯಾಸಗಳ ಬಗ್ಗೆ ಸೀಮಿತ ತಿಳುವಳಿಕೆ ತೋರುತ್ತದೆ.

ಈ ಅಂತರವನ್ನು ಪರಿಹರಿಸಲು, ಭಾರತದ ಮಧ್ಯ-ಪಶ್ಚಿಮ ಘಟ್ಟಗಳ ಅರಣ್ಯದಲ್ಲಿ ಒಂಬತ್ತು ವೃಕ್ಷ ಪ್ರಭೇದಗಳನ್ನು ನಾವು ಅಧ್ಯಯನ ಮಾಡಿದ್ದೇವೆ, ಇದು ಜಾಗತಿಕ ಜೀವವೈವಿಧ್ಯ ತಾಣವಾಗಿದ್ದು, ಇದು ವಿಶಿಷ್ಟವಾದ ಶುಷ್ಕ, ಉಷ್ಣತರ ಬೇಸಿಗೆಯ ಅವಧಿಗಳನ್ನು ನಂತರ ನಾಲ್ಕು ತಿಂಗಳ ಅವಧಿಯ ಮಳೆಗಾಲವನ್ನು ಹೊಂದಿದೆ. ಈ ಪ್ರದೇಶದಲ್ಲಿ ದ್ಯುತಿಸಂಶ್ಲೇಷಣೆ ತಾಪಮಾನ ಸಂವೇದನೆ ಅಥವಾ ಋತು ಅವಲಂಬನೆ ಅಧ್ಯಯನ ಇದುವರೆಗೂ ಮಾಡಲಾಗಿಲ್ಲ. ಭೂದೃಶ್ಯದಾದ್ಯಂತ ಸ್ಥಳಾಕೃತಿ, ಜಲ ಲಭ್ಯತೆ ಮತ್ತು ಸೂಕ್ಷ್ಮ ವಾತಾವರಣ ವ್ಯತ್ಯಾಸಗಳು (Das et al. 2015) ವಿಶಿಷ್ಟವಾದ ಸೂಕ್ಷ್ಮ ಆವಾಸಸ್ಥಾನಗಳನ್ನು ಸೃಷ್ಟಿಸುತ್ತವೆ. ಆರ್ದ್ರ-ಅವಲಂಬಿತ, ಸಾಮಾನ್ಯವಾಗಿ ನಿತ್ಯಹರಿದ್ವರ್ಣ ಪ್ರಭೇದಗಳು ತಗ್ಗು ಕಣಿವೆಗಳಲ್ಲಿ ಕಂಡುಬರುತ್ತವೆ, ಪರ್ಣಪಾತಿ ಮರಗಳು ಆಳವಿಲ್ಲದ ಮಣ್ಣನ್ನು ಹೊಂದಿರುವ ಎತ್ತರದ ಬೆಟ್ಟದ ತುದಿಗಳಲ್ಲಿ ಕಂಡುಬರುತ್ತವೆ ಮತ್ತು ತುಲನಾತ್ಮಕವಾಗಿ ಶುಷ್ಕ-ಅವಲಂಬಿತ ನಿತ್ಯಹರಿದ್ವರ್ಣ ಪ್ರಭೇದಗಳು ಮಧ್ಯಮ ಜಲ ಲಭ್ಯತೆಯೊಂದಿಗೆ ಬೆಟ್ಟದ ಇಳಿಜಾರುಗಳಲ್ಲಿ ವಾಸಿಸುತ್ತವೆ (Pascal 1988; Krishnadas et al. 2016). ಸ್ಥಳಾಕೃತಿಯ ವ್ಯತ್ಯಾಸಗಳು ಬೆಟ್ಟದ ಇಳಿಜಾರುಗಳಲ್ಲಿ ಅವುಗಳ ಸ್ಥಾನವನ್ನು ಅವಲಂಬಿಸಿ ಪ್ರಭೇದಗಳು ಅನುಭವಿಸುವ ಋತು ಅವಲಂಬಿತ ಜಲ ಲಭ್ಯತೆ ಮತ್ತು ಸೂಕ್ಷ್ಮ ಪರಿಸರ ತಾಪಮಾನವನ್ನು ಮತ್ತಷ್ಟು ವೈವಿಧ್ಯಗೊಳಿಸುತ್ತವೆ. ಇದು ಈ ಪ್ರಶ್ನೆಗೆ ಕಾರಣವಾಗುತ್ತದೆ: ಸೂಕ್ಷ್ಮ ಆವಾಸಸ್ಥಾನ ಮತ್ತು ತಾಪ ಗುಣ ವಿಶಿಷ್ಟ ಕ್ಷೇತ್ರ ಆದ್ರತೆ ವ್ಯತ್ಯಾಸಗಳು ಸಹ-ಸಂಭವಿಸುವ ವೃಕ್ಷ ಪ್ರಭೇದಗಳಲ್ಲಿ ದ್ಯುತಿಸಂಶ್ಲೇಷಕ ಉಷ್ಣ ಸಂವೇದನೆಯಲ್ಲಿನ ವ್ಯತ್ಯಾಸಕ್ಕೆ ಹೇಗೆ ಸಂಬಂಧಿಸಿವೆ?

ದ್ಯುತಿಸಂಶ್ಲೇಷಣೆ ಮತ್ತು ಪತ್ರ-ರಂಧ್ರ ವಾಹಕತೆ (g_s) ಉಷ್ಣ ಸಂವೇದನೆಯಲ್ಲಿನ ಋತು ಅವಲಂಬಿತ ವ್ಯತ್ಯಾಸಗಳನ್ನು ನಿರೂಪಿಸಲು, ನಾವು ಎರಡು ಋತುಗಳಲ್ಲಿ ಒಂಬತ್ತು ವೃಕ್ಷ ಪ್ರಭೇದಗಳ ಗುಂಪಿಗೆ CO_2 ಉಪಗ್ರಹಣ ದರ (A_{net}) ಮತ್ತು g_s ತಾಪಮಾನ ಪ್ರತಿಕ್ರಿಯೆಗಳನ್ನು ಮಾಪನ ಮಾಡಿದ್ದೇವೆ. ಆದರ್ಶ ತಾಪಮಾನದಲ್ಲಿ (T_{opt}), ದ್ಯುತಿಸಂಶ್ಲೇಷಣೆ ದರಗಳು ಪ್ರಕಾಶ ಪೂರಿತ, ಸಾಮಾನ್ಯ ಪರಿಸರ ಮಟ್ಟದ CO_2 ಸಾಂದ್ರತೆ ಮತ್ತು *RH* ಅಡಿಯಲ್ಲಿ ಅವುಗಳ ಗರಿಷ್ಠ ಸಾಮರ್ಥ್ಯದಲ್ಲಿ (A_{opt}) ಇರುತ್ತವೆ. ಆದ್ದರಿಂದ, ಶುಷ್ಕ ಮತ್ತು ಆರ್ದ್ರ ಅವಧಿಗಳಲ್ಲಿ ದ್ಯುತಿಸಂಶ್ಲೇಷಣೆಯಲ್ಲಿ (A_{opt}) ಋತು ಅವಲಂಬಿತ ವ್ಯತ್ಯಾಸದ ಮಾಪನಯನ್ನು ಒದಗಿಸಬಹುದು. ಪತ್ರ-ರಂಧ್ರ ವಾಹಕತೆ ವ್ಯತ್ಯಾಸದ ಸೂಚಕವಾಗಿ, ನಾವು T_{opt} ನ g_s ದರವನ್ನು ಮಾಪನ ಮಾಡಿದೆವು, ಆದರ್ಶ g_s ಎರಡು ಅಧ್ಯಯನ ಅವಧಿಗಳಲ್ಲಿ ಹೆಚ್ಚಾಗಿ ಎಲೆ ಪರಿಸರದಲ್ಲಿ ಮತ್ತು ಅತ್ಯಧಿಕ ದ್ಯುತಿಸಂಶ್ಲೇಷಣೆ ದರ ಪರಿಸ್ಥಿತಿಗಳನ್ನು ಸೂಚಿಸುತ್ತದೆ. ನಮ್ಮ ಅಧ್ಯಯನವು ಈ ಕೆಳಗಿನ ಪ್ರಶ್ನೆಗಳನ್ನು ಪರಿಹರಿಸುತ್ತದೆ:

೧) ಋತುಮಾನದ ಉಷ್ಣವಲಯದ ಕಾಡಿನಲ್ಲಿ ಶುಷ್ಕ ಮತ್ತು ಆರ್ದ್ರ ಋತುಗಳ ನಡುವೆ ಸಹವರ್ತಿ ವೃಕ್ಷ ಪ್ರಭೇದಗಳಲ್ಲಿ ದ್ಯುತಿಸಂಶ್ಲೇಷಣೆ ದರ ಮತ್ತು ಪತ್ರ-ರಂಧ್ರವಾಹಕತೆಯ ತಾಪಮಾನದ ಪ್ರತಿಕ್ರಿಯೆಗಳು ಎಷ್ಟರ ಮಟ್ಟಿಗೆ ಭಿನ್ನವಾಗಿವೆ?

೨) ದ್ಯುತಿಸಂಶ್ಲೇಷಣೆ ದರಗಳು ಮತ್ತು ಪತ್ರ-ರಂಧ್ರವಾಹಕತೆಯಲ್ಲಿರುವ ಋತು ಅವಲಂಬಿತ ವ್ಯತ್ಯಾಸಗಳು ಪ್ರಭೇದಗಳ ವಿಭಿನ್ನ ಸ್ಥಳಾಕೃತಿಯ ಸ್ಥಾನಗಳಿಗೆ ಸಂಬಂಧಿಸಿವೆಯೇ?

ಆರ್ದ್ರ ಅವಧಿಯಲ್ಲಿ ಎಲ್ಲಾ ವೃಕ್ಷ ಪ್ರಭೇದಗಳಲ್ಲಿ ದ್ಯುತಿಸಂಶ್ಲೇಷಣೆ ದರಗಳು ಹೆಚ್ಚಿರುತ್ತವೆ ಎಂದು ನಾವು ಊಹಿಸುತ್ತೇವೆ. ಆರ್ದ್ರ ಮತ್ತು ಶುಷ್ಕ ಅವಧಿಗಳ ನಡುವಿನ ದ್ಯುತಿಸಂಶ್ಲೇಷಣೆ ದರ ವ್ಯತ್ಯಾಸಗಳು ಬೆಟ್ಟದ ತುದಿಯ ಪರ್ಣಪಾತಿ ಪ್ರಭೇದಗಳಲ್ಲಿ ಅತೀ ಹೆಚ್ಚಾಗಿಯೂ, ಬೆಟ್ಟದ ಇಳಿಜಾರಿನ ಪ್ರಭೇದಗಳಲ್ಲಿ ಮಧ್ಯಮವಾಗಿಯೂ ಮತ್ತು ಕಣಿವೆಯ ಪ್ರಭೇದಗಳಲ್ಲಿ ಕಡಿಮೆ ಇರುತ್ತವೆ ಎಂದು ನಾವು ಊಹಿಸುತ್ತೇವೆ. ಸಸಿಗಳಲ್ಲಿ ಈ ಹಿಂದೆ ಗಮನಿಸಿದಂತೆ (Kositsup et al. 2008; Slot and Winter 2017a) ಮತ್ತು ಪ್ರಾಯೋಗಿಕ ತಾಪಮಾನ ಏರಿಕೆಗೆ ಪ್ರತಿಕ್ರಿಯೆಯಾಗಿ (Crous et al. 2022) ಶುಷ್ಕ ಅವಧಿಯಲ್ಲಿ T_{opt} ಹೆಚ್ಚಾಗುತ್ತದೆ ಎಂದು ನಾವು ನಿರೀಕ್ಷಿಸುತ್ತೇವೆ. ಹೆಚ್ಚುವರಿಯಾಗಿ, ಆರ್ದ್ರ ಅವಧಿಯಲ್ಲಿ T_{opt} ಲ್ಲಿ ಪತ್ರ-ರಂಧ್ರ ವಾಹಕತೆ ಸಾಮಾನ್ಯವಾಗಿ ಹೆಚ್ಚಾಗಿರುತ್ತದೆ ಎಂದು ನಾವು ನಿರೀಕ್ಷಿಸುತ್ತೇವೆ, ಆದರೆ ವಿಭಿನ್ನ ನೀರಿನ ಪ್ರವೇಶ ತಂತ್ರಗಳು ಮತ್ತು ಸ್ಥಳಲಕ್ಷಣ ಸಂಬಂಧಗಳಲ್ಲಿ ಋತು ಅವಲಂಬಿತ ವ್ಯತ್ಯಾಸಗಳ ಪ್ರಮಾಣವು ಪ್ರಭೇದಗಳಲ್ಲಿ ಬದಲಾಗಬಹುದು.

೨ । ವಸ್ತುಗಳು ಮತ್ತು ವಿಧಾನಗಳು

೨.೧. । ಅಧ್ಯಯನ ತಾಣ ಮತ್ತು ವೃಕ್ಷ ಪ್ರಭೇದಗಳು

ಭಾರತದ ಕರ್ನಾಟಕದಲ್ಲಿ ಉತ್ತರ ಕನ್ನಡ ಜಿಲ್ಲೆಯ (೧೪.೪೭೯೧೫೭°, ೭೪.೭೫೮೩೦೪°, ೫೨೩ ಮೀ.) ಮಧ್ಯ ಪಶ್ಚಿಮ ಘಟ್ಟಗಳ ಭೂದೃಶ್ಯದಲ್ಲಿರುವ ಅರಣ್ಯ ಸ್ಥಳದಲ್ಲಿ ನಾವು ಮೂಲ ಸ್ಥಾನಿಕ ಮಾಪನಗಳನ್ನು ನಡೆಸಿದ್ದೇವೆ (ಚಿತ್ರ ೧). ಅಧ್ಯಯನ ಸ್ಥಳವು ಉಬ್ಬು ತಗ್ಗುಗಳ ಭೂಪ್ರದೇಶವನ್ನು ಹೊಂದಿದೆ, ಕಣಿವೆಗಳಲ್ಲಿ ಸಮುದ್ರ ಮಟ್ಟಕ್ಕಿಂತ ೫೦೦–೫೫೦ ಮೀ ಮತ್ತು ಬೆಟ್ಟದ ತುದಿಗಳಲ್ಲಿ ಸುಮಾರು ೫೫೦–೬೦೦ ಮೀ. ವರೆಗೆ ಎತ್ತರವಿದೆ. ನಾವು ಮೂರು ಸ್ಥಳಾಕೃತಿಯ ಸ್ಥಾನಗಳಲ್ಲಿ ಪ್ರತಿಯೊಂದರಲ್ಲೂ ಮೂರು ವೃಕ್ಷ ಪ್ರಭೇದಗಳ ಗುಂಪನ್ನು ಆಯ್ಕೆ ಮಾಡಿದ್ದೇವೆ. ಕಡಿಮೆ ವಿತರಣೆ ಸಾಂದ್ರತೆಯ ಪರ್ಣಪಾತಿ ವೃಕ್ಷ ಪ್ರಭೇದಗಳಿಂದ ಕೂಡಿದ ಬೆಟ್ಟದ ತುದಿಗಳಲ್ಲಿ, ನಾವು ಆರಿಸಿಕೊಂಡ ಪ್ರಭೇದಗಳು: ಕವಲು (*Careya arborea* Roxb.; Lecythidaceae), ಅಳಲೆ - (*Terminalia chebula* Retz.; Combretaceae), ಮತ್ತು ಹುನಾಲು (*T. paniculata* B.Heyne ex Roth; Combretaceae) - ಎಲ್ಲವೂ ಪರ್ಣಪಾತಿ ವೃಕ್ಷಗಳು. ಶುಷ್ಕ ಮಣ್ಣುಗಳನ್ನು ಹೊಂದಿರುವ ಬೆಟ್ಟದ ಇಳಿಜಾರುಗಳಲ್ಲಿ, ನಾವು ಮೂರು ನಿತ್ಯಹರಿದ್ವರ್ಣ ವೃಕ್ಷ ಪ್ರಭೇದಗಳನ್ನು ಆಯ್ಕೆ ಮಾಡಿದ್ದೇವೆ: ಅಡಚರೆ (*Memecylon umbellatum* Burm.f.; Melastomataceae), ಹಣಗೆರೆ (*Psydrax dicoccos*; (Gaertn.) Merr.; Rubiaceae), ಮತ್ತು ಹೆಕ್ಕೆರಕಲು (*Tetrapilus dioicus* Roxb.; Oleaceae). ಕೊನೆಯದಾಗಿ, ಆರ್ದ್ರ ಕಣಿವೆಗಳಲ್ಲಿ, ನಾವು ಮೂರು ನಿತ್ಯಹರಿದ್ವರ್ಣ ಪ್ರಭೇದಗಳನ್ನು ಆಯ್ಕೆ ಮಾಡಿದ್ದೇವೆ: ಹೈಗ (*Hopea ponga* Wall.; Dipterocarpaceae), ಹೆಡಾಗಲ (*Knema attenuata* (Hook.f. & Th.) Warb.; Myristicaceae), ಮತ್ತು ಅರಿಶಿನಂಡಿ (*Garcinia cambogioides* var. *cambogioides*; Clusiaceae). C. arborea ಮತ್ತು *T. paniculata* ದಂತಹ ಬೆಟ್ಟದ ತುದಿಯ ಮರಗಳು ಕಾಡ್ಗಿಚ್ಚು ಪೀಡಿತ ಪ್ರದೇಶಗಳೊಂದಿಗೆ ಸಂಬಂಧ ಹೊಂದಿವೆ, ಆದರೆ *T. chebula* ಮಿಶ್ರ ಪರ್ಣಪಾತಿ ಕಾಡುಗಳಲ್ಲಿ ಕಂಡುಬರುತ್ತದೆ, ಮಧ್ಯ ಪಶ್ಚಿಮ ಘಟ್ಟಗಳು ಇದರ ಅತ್ಯಂತ ಸೂಕ್ತವಾದ ಆವಾಸಸ್ಥಾನವಾಗಿದೆ (Kailash et al. 2022). ಬೆಟ್ಟದ ಇಳಿಜಾರುಗಳಲ್ಲಿ *M. umbellatum* ಉತ್ತರ ಪಶ್ಚಿಮ ಘಟ್ಟಗಳಲ್ಲಿನ ಮಧ್ಯಮ-ಎತ್ತರದ ಕಾಡುಗಳನ್ನು ನಿರೂಪಿಸುತ್ತದೆ, ಇದು *Syzygium* ಪ್ರಭೇದಗಳೊಂದಿಗೆ ಸಂಬಂಧ ಹೊಂದಿದೆ ಮತ್ತು *Actinodaphne* ಪ್ರಭೇದಗಳು, ೧೦೦೦ ಮೀ. ವರೆಗೆ ಆರ್ದ್ರ ಮತ್ತು ಶುಷ್ಕ ಕಾಡುಗಳಲ್ಲಿ ಕಂಡುಬರುತ್ತವೆ (Shigwan et al. 2024), ಮತ್ತು ಹೆಚ್ಚಾಗಿ *T. dioicus* ಜೊತೆ ಸಹ-ಸಂಭವಿಸುತ್ತವೆ. ಕಣಿವೆಗಳಲ್ಲಿ, *Hopea* ಪ್ರಭೇದಗಳು ಅಬಾಧಿತ ನಿತ್ಯಹರಿದ್ವರ್ಣ ಕಾಡುಗಳನ್ನು ಸೂಚಿಸುತ್ತವೆ ಮತ್ತು ಅಧ್ಯಯನ ಸ್ಥಳದ ಬಳಿ ಏಕಪ್ರಭುತ್ವದ ಕ್ಷೇತ್ರಗಳನ್ನು ರಚಿಸುವುದು ಸಹ ಗಮನಿಸಬಹುದು. *K. attenuata* ಮತ್ತು *Garcinia* ಪ್ರಭೇದಗಳು ಆರ್ದ್ರ ಕಣಿವೆಗಳನ್ನು ನಿರೂಪಿಸುತ್ತವೆ. ನಮ್ಮ ಅಧ್ಯಯನ ಸ್ಥಳದಲ್ಲಿ, ಪರ್ಣಪಾತಿ ಪ್ರಭೇದಗಳು ಪ್ರಧಾನವಾಗಿ ಬೆಟ್ಟದ ತುದಿಗಳು ಮತ್ತು ತೆರೆದ ಪ್ರದೇಶಗಳನ್ನು ಆಕ್ರಮಿಸುತ್ತವೆ, ಆದರೆ ನಿತ್ಯಹರಿದ್ವರ್ಣ ಪ್ರಭೇದಗಳು ಇಳಿಜಾರು ಮತ್ತು ಕಣಿವೆಗಳಲ್ಲಿ ಪ್ರಾಬಲ್ಯ ಹೊಂದಿವೆ, ಇದು ಸ್ಪಷ್ಟವಾದ ಸ್ಥಳಾಕೃತಿಯ ಪ್ರತ್ಯೇಕತೆಯನ್ನು ದರ್ಶಿಸುತ್ತದೆ. ಇದಕ್ಕೆ ವಿರುದ್ಧವಾಗಿ, ಬೆಟ್ಟದ ಇಳಿಜಾರು ಮತ್ತು ಕಣಿವೆ ಪ್ರದೇಶಗಳಲ್ಲಿ ಪರ್ಣಪಾತಿ ಪ್ರಭೇದಗಳನ್ನು ಕಾಣುವುದು ಕಷ್ಟ. ಈ ಪ್ರತ್ಯೇಕತೆಯು ಪರ್ಣಪಾತಿ ಮತ್ತು ನಿತ್ಯಹರಿದ್ವರ್ಣ ಪ್ರಕಾರಗಳಿಗೆ ಎಲ್ಲಾ ವಲಯಗಳಲ್ಲಿ ನೇರ ಹೋಲಿಕೆಗಳನ್ನು ಮಾಡುವ ನಮ್ಮ ಸಾಮರ್ಥ್ಯವನ್ನು ಸೀಮಿತಗೊಳಿಸಿದೆ. ಆಯ್ದ ಒಂಬತ್ತು ಪ್ರಭೇದಗಳು ಒಟ್ಟಾಗಿ ಈ ಪರಿಸರ ವ್ಯವಸ್ಥೆಯಲ್ಲಿ ಆಯ್ದ ಸಾಮಾನ್ಯ ವೃಕ್ಷ ಪ್ರಭೇದಗಳನ್ನು ಪ್ರತಿನಿಧಿಸುತ್ತವೆ (Pascal 1988) ಮತ್ತು ಪಶ್ಚಿಮ ಘಟ್ಟಗಳ ಭೂದೃಶ್ಯದಾದ್ಯಂತ ನೀರಿನ ಸಂಬಂಧದ ಸಾಮಾನ್ಯ ಮಾದರಿಗಳನ್ನು ಪ್ರತಿಬಿಂಬಿಸುತ್ತವೆ (Krishnadas et al. 2021), ಸ್ಥಳಾಕೃತಿಯ ಸ್ಥಾನಗಳಿಗೆ ನಿಕಟ ಸಂಬಂಧ ಹೊಂದಿವೆ. ಇಳಿಜಾರಿನ ಉದ್ದಕ್ಕೂ ಮರಗಳ ವಿಶಿಷ್ಟ ವಿತರಣೆ ಮತ್ತು ಭೂಪ್ರದೇಶದಿಂದಾಗಿ, ನೆಲದಿಂದ ಸುಮಾರು ೨–೫ ಮೀ. ಎತ್ತರದ ಕೊಂಬೆಗಳನ್ನು ಮಾಪನಕ್ಕಾಗಿ ಬಳಸಬಹುದಾಗಿದೆ. ಮಾದರಿಯನ್ನು ತೆಗೆದುಕೊಂಡ ಪ್ರತಿಯೊಂದು ಮರಕ್ಕೂ ನಾವು ಮಾಪನಕ್ಕಾಗಿ ಒಂದು ಕೊಂಬೆಯನ್ನು ಆರಿಸಿದ್ದೇವೆ, ಅದನ್ನು ಮರಕ್ಕೆ ಹಾನಿಯಾಗದಂತೆ ಅಥವಾ ಬೇರ್ಪಡಿಸದೆ ನೆಲದ ಕಡೆಗೆ ಬಾಗಿಸಿ, ಎಲೆಗಳನ್ನು ಮಾಪನ ಮಾಡಲು ಅನುಕೂಲವಾಗುವಂತೆ ಹಗ್ಗಗಳನ್ನು ಬಳಸಿ ಅದನ್ನು ಸ್ಥಿರಗೊಳಿಸಿದ್ದೇವೆ. ನಾವು ಪ್ರತಿ ಪ್ರಭೇದಕ್ಕೆ ಕನಿಷ್ಠ ಮೂರು ಜೈವಿಕ ಪ್ರತಿಕೃತಿಗಳು/ಮಾದರಿ ಅಥವಾ ಪ್ರತ್ಯೇಕ ಮರಗಳನ್ನು ಮಾಪನ ಮಾಡಿದ್ದೇವೆ, ಆರ್ದ್ರ ಮತ್ತು ಶುಷ್ಕ ಅವಧಿಯ ಅಭಿಯಾನಗಳಲ್ಲಿ ಕೂಡ ಅದೇ ಮರಗಳನ್ನು ಮಾಪನ ಮಾಡಿದ್ದೇವೆ.

೨.೨. । ಅಧ್ಯಯನ ಅವಧಿಗಳು

ನಾವು ಎರಡು ಅವಧಿಗಳಲ್ಲಿ ಮೂಲ ಸ್ಥಾನಿಕ ಮಾಪನಗಳನ್ನು ನಡೆಸಿದ್ದೇವೆ: ೨೦೨೦ ರಲ್ಲಿ ಮುಂಗಾರು ನಂತರದ ಆರಂಭಿಕ ಅವಧಿ ಮತ್ತು ೨೦೨೧ ರಲ್ಲಿ ಶುಷ್ಕ ಬೇಸಿಗೆಯ ಅವಧಿ. 'ಆರ್ದ್ರ ಅವಧಿ' ಎಂದು ಕರೆಯಲ್ಪಡುವ ಆರಂಭಿಕ ಮುಂಗಾರು ಅಭಿಯಾನವನ್ನು ೨೦೨೦ ರ ನವೆಂಬರ್ ನಿಂದ ಡಿಸೆಂಬರ್ ಮಧ್ಯದವರೆಗೆ ನಡೆಸಲಾಯಿತು, ಇದು ೪ ತಿಂಗಳ ಅವಧಿಯ ಮುಂಗಾರು ಮಳೆಯ ನಂತರ ಸುಮಾರು ೧–೧.೫ ತಿಂಗಳುಗಳ ನಂತರ (ಚಿತ್ರ ೧, ಫಲಕ ೧). ಎರಡನೇ ಅಭಿಯಾನವು ಮಾರ್ಚ್ ಅಂತ್ಯದಿಂದ ಏಪ್ರಿಲ್ ಮಧ್ಯದವರೆಗೆ ನಡೆಯಿತು ಮತ್ತು ಬೇಸಿಗೆಯ ಆರಂಭದಿಂದ ಮಧ್ಯದ ಅವಧಿಗೆ ಹೊಂದಿಕೆಯಾಯಿತು

(ಇನ್ನು ಮುಂದೆ ಬೇಸಿಗೆ ಎಂದು ಕರೆಯಲಾಗುತ್ತದೆ). ಬೇಸಿಗೆಯ ಅವಧಿಯು ತಿಂಗಳಿಗೆ ೧೦೦ ಮೀ.ಮೀ. ಮಳೆಯಿಂದ ಸುಮಾರು ೪-೫ ತಿಂಗಳುಗಳವರೆಗೆ (ಡಿಸೆಂಬರ್ ನಿಂದ ಮೇ ಮಧ್ಯದವರೆಗೆ ಮುಂಗಾರು ಪ್ರಾರಂಭವಾಗುವವರೆಗೆ) ನಿರೂಪಿಸಲ್ಪಟ್ಟಿದೆ. ಸಾಮಾನ್ಯವಾಗಿ, ಏಪ್ರಿಲ್ ಅಂತ್ಯ ಮತ್ತು ಮೇ ಆರಂಭದಲ್ಲಿ ವಾಯು ಉಷ್ಣತೆಯು ಈ ಪ್ರದೇಶದಲ್ಲಿ ಅತ್ಯಧಿಕ ಮಟ್ಟವನ್ನು ತಲುಪುತ್ತದೆ. ಬೇಸಿಗೆಯ ಅವಧಿಯ ಸರಾಸರಿ ಗರಿಷ್ಠ ವಾಯು ಉಷ್ಣತೆಯು ೩೮.೪ °C ಆಗಿದ್ದು, ಇದು ಮುಂಗಾರು ನಂತರದ ಅವಧಿಗಿಂತ (೩೪ °C) ಸುಮಾರು ೪ °C ಬೆಚ್ಚಗಿರುತ್ತದೆ. ಬೇಸಿಗೆಯ ಅವಧಿಯಲ್ಲಿ ಸಾಪೇಕ್ಷ ಆರ್ದ್ರತೆ (*RH*) ~೭೦.೪ % (೫೯.೬–೮೨.೩) ಆಗಿದ್ದರೆ, ಮುಂಗಾರು ನಂತರದ ಅವಧಿಯಲ್ಲಿ ಇದು ೭೫.೬% (೬೫.೩–೮೭.೦) ಆಗಿತ್ತು. ಈ ಸ್ಥಳದ ಒಟ್ಟು ಸರಾಸರಿ ವಾರ್ಷಿಕ ಮಳೆ ಸುಮಾರು ೪೦೦೦ ಮಿ.ಮೀ. ತಾಪಮಾನ ಮತ್ತು ಮಳೆಯ ದತ್ತಾಂಶವು ಅಧ್ಯಯನ ಸ್ಥಳದಲ್ಲಿ ಸ್ಥಾಪಿಸಲಾದ WatchDog 2000 ಸ್ವಯಂಚಾಲಿತ ವಾತಾವರಣ ಮಾಪಕದಿಂದ (Spectrum Technologies, Illinois, USA) ಬಂದಿದೆ. ಆವಿಯ ಒತ್ತಡದ ಕೊರತೆ (*VPD*) ಆರ್ದ್ರ ಅವಧಿಯಲ್ಲಿ ದಿನವಿಡೀ ೨ kPa ನಲ್ಲಿ ಸ್ಥಿರವಾಗಿರುತ್ತದೆ ಆದರೆ ಶುಷ್ಕ ಅವಧಿಯಲ್ಲಿ ೨ ರಿಂದ ೩ kPa ವರೆಗೆ ಬದಲಾಗುತ್ತದೆ. ಪೂರಕ ಚಿತ್ರ ೧ ವಾಯು ಉಷ್ಣತೆ, *RH* ಮತ್ತು ಶುಷ್ಕ ಮತ್ತು ಆರ್ದ್ರ ಅವಧಿಗಳ ನಡುವಿನ ಎಲೆಯಿಂದ ಗಾಳಿಗೆ *VPD* ಯಲ್ಲಿನ ದೈನಂದಿನ ವ್ಯತ್ಯಾಸಗಳನ್ನು ವಿವರಿಸುತ್ತದೆ.

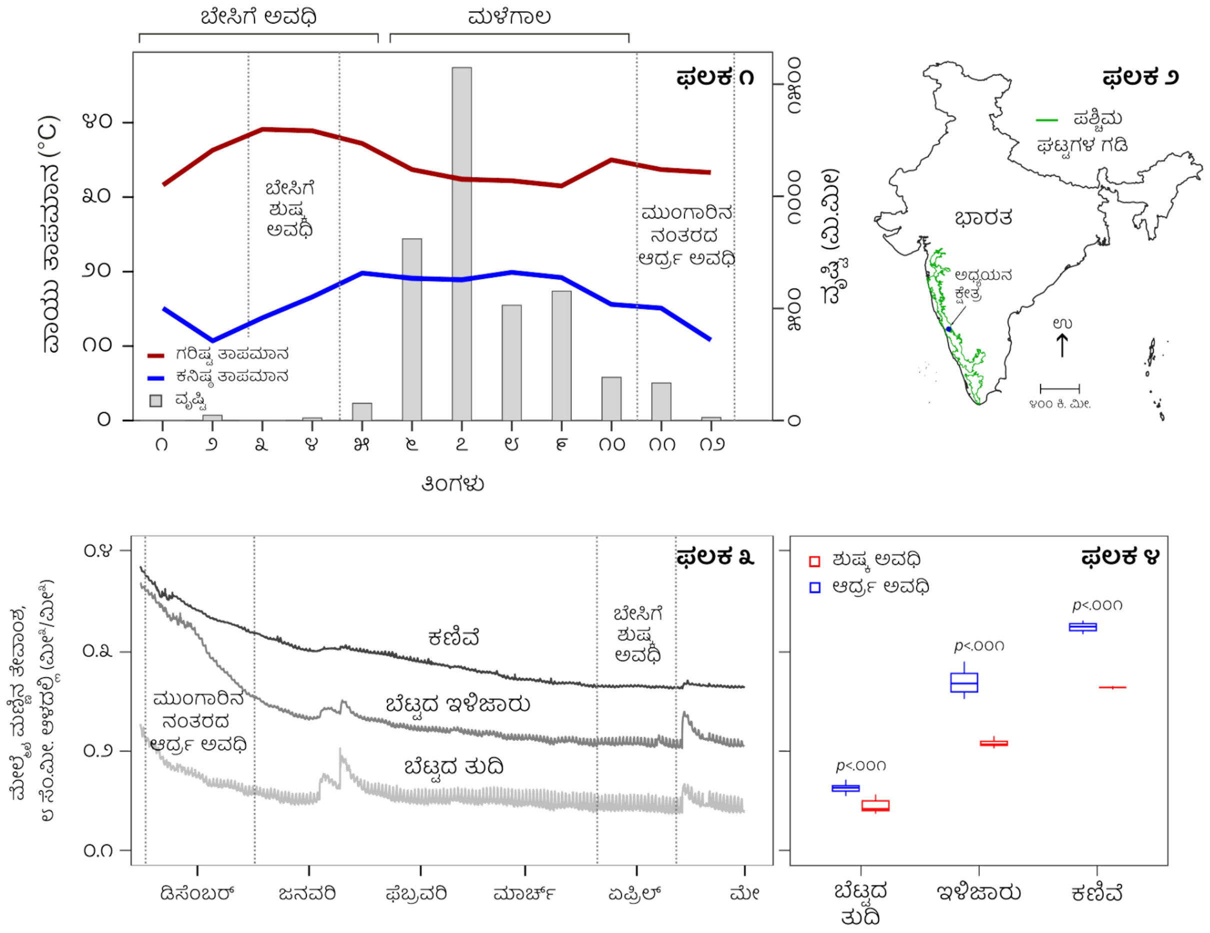

ಚಿತ್ರ ೧: ಫಲಕ ೧) ಭಾರತದ ಪಶ್ಚಿಮ ಘಟ್ಟಗಳಲ್ಲಿನ ಅಧ್ಯಯನ ಸ್ಥಳದ ಮಾಸಿಕ ವಾಯು ಉಷ್ಣತೆ (ಪ್ರಾಥಮಿಕ ಲಂಬಾಕ್ಷ) ಮತ್ತು ಮಳೆ (ದ್ವಿತೀಯ ಲಂಬಾಕ್ಷ). ದತ್ತಾಂಶವು ೨೦೧೦–೨೦೨೧ರ ಅವಧಿಯಲ್ಲಿ ಸಂಗ್ರಹಿಸಲಾದ WatchDog 2000 ವಾತಾವರಣ ಮಾಪಕ ಕೇಂದ್ರದಿಂದ ಪಡೆದ ದತ್ತಾಂಶವನ್ನು ಬಳಸಿಕೊಂಡು ಲೆಕ್ಕಹಾಕಿದ ಮಾಸಿಕ ಸರಾಸರಿಗಳಾಗಿವೆ. ನಕ್ಷೆ (ಫಲಕ ೨) ಭಾರತದಲ್ಲಿ ಅಧ್ಯಯನ ಸ್ಥಳ ಮತ್ತು ಪಶ್ಚಿಮ ಘಟ್ಟಗಳ ಗಡಿಯನ್ನು ತೋರಿಸಲಾಗಿದೆ. ಫಲಕ ೩) ನಂತರದ ವರ್ಷದಲ್ಲಿ ಮಾಪನ ಮಾಡಲಾದ ಮೂರು ಸ್ಥಳಾಕೃತಿ ಗುಂಪುಗಳಿಗೆ ಅನುಗುಣವಾಗಿ ಮೂರು ಬಿಂದುಗಳಿಗೆ ಮೇಲ್ಮೈ ಮಣ್ಣಿನ ತೇವಾಂಶವನ್ನು ತೋರಿಸುತ್ತದೆ. ಫಲಕ ೪, ಫಲಕ ೩ ರಲ್ಲಿನ ದತ್ತಾಂಶದಿಂದ ಪಡೆದ ಮೂರು ಸ್ತರಗಳಿಗೆ ಸರಾಸರಿ ಮೇಲ್ಮೈ ಮಣ್ಣಿನ ತೇವಾಂಶವನ್ನು ತೋರಿಸುತ್ತದೆ.

೨.೩ । CO_2 ಉಪಗ್ರಹಣ ದರ ತಾಪಮಾನ ಪ್ರತಿಕ್ರಿಯೆ ಮಾಪನ

ನಾವು CO_2 ಉಪಗ್ರಹಣ ದರದ ತಾಪಮಾನ ಪ್ರತಿಕ್ರಿಯೆಯನ್ನು A_{net}, ಪ್ರತಿದೀಪಕ ಪತ್ರಕೋಶ (Li-6400-40) ಹೊಂದಿರುವ portable infrared gas analyser (IRGA) Li-6400 XT (LiCor, Lincoln, USA) ಬಳಸಿ ಮಾಪನ ಮಾಡಿದ್ದೇವೆ. ನೆಲದಿಂದ ಸುಮಾರು ೨–೫ ಮೀ ಎತ್ತರದಿಂದ ವಯಸ್ಕ ಮರಗಳ ಸೂರ್ಯನ ಬೆಳಕಿಗೆ ಒಡ್ಡಿಕೊಂಡ ಕೊಂಬೆಗಳಿಂದ ಕಡಿಮೆ ಅಥವಾ ಮಧ್ಯ-ಮೇಲಾವರಣ, ಸಂಪೂರ್ಣವಾಗಿ ಪ್ರಬುದ್ಧ ಮತ್ತು ಆರೋಗ್ಯಕರ ಎಲೆಗಳನ್ನು ನಾವು ಮಾಪನ ಮಾಡಿದ್ದೇವೆ. ಒಂದು ವಿಶಿಷ್ಟ ಮಾಪನ ಅನುಕ್ರಮವು ಪತ್ರಕೋಶದಲ್ಲಿ ಎಲೆಗಳನ್ನು ವಿವಿಧ ತಾಪಮಾನ ಬಿಂದುಗಳಿಗೆ ಕ್ರಮೇಣ ಬಿಸಿ ಮಾಡುವುದನ್ನು ಒಳಗೊಂಡಿತ್ತು. ಬಳಸಿದ ಎಲೆ ತಾಪಮಾನಗಳು: ೨೦, ೨೪, ೨೮, ೩೨, ೩೬, ೪೦, ೪೪, ಮತ್ತು ೪೮ °C. ವಿಕಿರಣ ಮಟ್ಟವನ್ನು ೧೦೦೦ µmol $m^{-೨}$ $s^{-೧}$, CO_2 ಸಾಂದ್ರತೆಯನ್ನು ೪೦೦ µmol $mol^{-೧}$ ಗೆ ಹೊಂದಿಸಲಾಗಿದೆ. ತಾಪಮಾನ ಪ್ರತಿಕ್ರಿಯೆ ಮಾಪನದ ಸಮಯದಲ್ಲಿ ಉಪಕರಣದಿಂದ ದಾಖಲಿಸಲಾದ

ಬಾಹ್ಯ PAR (ದ್ಯುತಿಸಂಶ್ಲೇಷಕವಾಗಿ ಸಕ್ರಿಯ ವಿಕಿರಣ) ಸಂವೇದಕ ದತ್ತಾಂಶವನ್ನು ಸೂಕ್ಷ್ಮ ಆವಾಸಸ್ಥಾನ ಬೆಳಕಿನ ಮಟ್ಟವನ್ನು ಸೂಚಿಸಲು ಬಳಸಲಾಗುತ್ತಿತ್ತು.

IRGA ದಲ್ಲಿ ತೇವಾಂಶದ ಘನೀಕರಣವನ್ನು ತಡೆಗಟ್ಟಲು, ನಾವು ಕಡಿಮೆ ಎಲೆ ತಾಪಮಾನವನ್ನು ಇಬ್ಬನಿ ಬಿಂದುವಿಗಿಂತ ೪ °C ಮೇಲೆ ಹೊಂದಿಸಿದ್ದೇವೆ, ಇದು ನಾವು ಮಾಪನ ಸಾಧ್ಯವಾಗುವ ಕಡಿಮೆ ತಾಪಮಾನವನ್ನು ಸೀಮಿತಗೊಳಿಸಿದೆವು. ಎಲೆಗಳು ಆರಂಭಿಕ ಪತ್ರಕೋಶದ ಸ್ಥಿತಿಯಲ್ಲಿ ಕನಿಷ್ಠ ೧೦ ನಿಮಿಷಗಳ ಕಾಲ ಸ್ಥಿರವಾಗಲು ನಾವು ಅವಕಾಶ ಮಾಡಿಕೊಟ್ಟೆವು. ಸ್ಥಿರೀಕರಣದ ನಂತರ, ಕನಿಷ್ಠ ೬–೭ ನಿಮಿಷಗಳ ಸ್ಥಿರ ಪರಿಸ್ಥಿತಿಗಳ ನಂತರ ದತ್ತಾಂಶವನ್ನು ದಾಖಲಿಸಲಾಯಿತು. ದತ್ತಾಂಶ ಸಂಗ್ರಹಣಾನಂತರ, ಪತ್ರಕೋಶದ ಗಾಳಿಯನ್ನು ~೪ °C ಹೆಚ್ಚಿನ ತಾಪಮಾನಕ್ಕೆ ಹೊಂದಿಸಲಾಯಿತು ಮತ್ತು ನಂತರ ಇದೇ ರೀತಿಯ ಸ್ಥಿರೀಕರಣ ಅನುಕ್ರಮವನ್ನು ಅನುಸರಿಸಲಾಯಿತು.

ನಾವು ಉಪಕರಣವು ತಲುಪಬಹುದಾದ ನಾಮಮಾತ್ರದ ಅತ್ಯಧಿಕ ತಾಪಮಾನವನ್ನು ತಲುಪಲು ಪ್ರಯತ್ನಿಸಿದಾಗ, ಎಲೆಯ ತಾಪಮಾನವು ಆಗಾಗ್ಗೆ ಸಾಧ್ಯವಾದಷ್ಟು ಹೆಚ್ಚಿನ ಪತ್ರಕೋಶದ ತಾಪಮಾನಕ್ಕಿಂತ ಕಡಿಮೆಯಿರುತ್ತದೆ, ಇದು ನಾವು ಮಾಪನ ಮಾಡ ಬಹುದಾದ ತಾಪಮಾನ ಶ್ರೇಣಿಯ ಮೇಲಿನ ತುದಿಯನ್ನು ಸೀಮಿತಗೊಳಿಸುತ್ತದೆ. ಬೇಸಿಗೆಯ ಅವಧಿಗೆ ಎಲೆಯ ತಾಪಮಾನ (T_{leaf}) ವ್ಯಾಪ್ತಿಯು ೨೧.೪–೪೫.೦ °C ಆಗಿತ್ತು ಮತ್ತು ಮುಂಗಾರು ನಂತರದ ಅವಧಿಯಲ್ಲಿ, ಇದು ೨೩.೨–೪೧.೧ °C ಆಗಿತ್ತು. ಪತ್ರಕೋಶದ ಗಾಳಿಯ *RH* ಅನ್ನು ಸುಮಾರು ೬೦±೫ % ನಲ್ಲಿ ನಿರ್ವಹಿಸಲಾಯಿತು. ಆದಾಗ್ಯೂ, ೩೫°C ಗಿಂತ ಹೆಚ್ಚಿನ ತಾಪಮಾನದಲ್ಲಿ ಶುಷ್ಕ ಮತ್ತು ಆರ್ದ್ರ ಅವಧಿಯ ಅಭಿಯಾನಗಳಲ್ಲಿ, *RH* ಮಟ್ಟಗಳು ಸುಮಾರು ೪೦% ಕ್ಕೆ ಇಳಿದವು. ದ್ಯುತಿಸಂಶ್ಲೇಷಣೆ ತಾಪಮಾನ ಪ್ರತಿಕ್ರಿಯೆ ನಿಯತಾಂಕಗಳನ್ನು ಅಂದಾಜು ಮಾಡಲು, ನಾವು June ಮತ್ತು ಇತರರ (2004) ಮಾದರಿಯನ್ನು CO_2 ಉಪಗ್ರಹಣ ದರ ಪ್ರತಿಕ್ರಿಯೆಗೆ ಈ ಕೆಳಗಿನಂತೆ ಅಳವಡಿಸಿದ್ದೇವೆ:

$$A_{net}(T_{leaf}) = A_{opt} \times e^{-\left(\frac{T_{leaf}-T_{opt}}{\Omega}\right)^2}$$ ಸಮೀಕರಣ ೧

ಇಲ್ಲಿ A_{net} ಎಂಬುದು ನಿರ್ದಿಷ್ಟ ಎಲೆ ತಾಪಮಾನ, CO_2 ಸಾಂದ್ರತೆ ಮತ್ತು ವಿಕಿರಣ ಮಟ್ಟದಲ್ಲಿ ದ್ಯುತಿಸಂಶ್ಲೇಷಕ CO_2 ಉಪಗ್ರಹಣ ದರವಾಗಿದೆ. T_{opt} ಎಂಬುದು A_{net} ನ ಆದರ್ಶ ತಾಪಮಾನದ ಮೌಲ್ಯವಾಗಿದೆ ಮತ್ತು A_{net} ಆದರ್ಶ ತಾಪಮಾನದ ಅಥವಾ ಗರಿಷ್ಠ CO_2 ಸಂಯೋಜನಾದರದಲ್ಲಿ A_{net} ಆಗಿದೆ. ವಕ್ರರೇಖೆಯ ಶಿಖರದ ಅಗಲವನ್ನು ಪ್ರತಿನಿಧಿಸುವ ನಿಯತಾಂಕ Ω, T_{opt} ಮತ್ತು T_{opt} ನಲ್ಲಿ A_{net} ಅದರ ಮೌಲ್ಯದ ೩೭% ಕ್ಕೆ ಇಳಿಯುವ ತಾಪಮಾನದ ನಡುವಿನ ತಾಪಮಾನ ವ್ಯತ್ಯಾಸವಾಗಿದೆ; ಆದರ್ಶ ತಾಪಮಾನಕ್ಕಿಂತ ಕಡಿಮೆ ಮತ್ತು ಅಧಿಕ ತಾಪಮಾನಗಳಲ್ಲಿ, Cunningham ಮತ್ತು Read (2002) ರ ಪ್ಯಾರಾಬೋಲಿಕ್ ತಾಪಮಾನ ಪ್ರತಿಕ್ರಿಯೆ ವಕ್ರರೇಖೆಗೆ ಹೋಲಿಸಿದರೆ, June ಮತ್ತು ಇತರರ (2004) ಅಸಮಪಾರ್ಶ್ವದ ಶಿಖರದ ಕಾರ್ಯವು ನಮ್ಮ ದತ್ತಾಂಶದಲ್ಲಿ, ವಿಶೇಷವಾಗಿ ಶಿಖರವನ್ನು ಸೆರೆಹಿಡಿಯುವಲ್ಲಿ (T_{opt}) ಉತ್ತಮ ಗಣಿತೀಯ ಫಲನ ಒದಗಿಸಿತು. ಪ್ರತಿ ಪ್ರಭೇದದ ಅವಧಿಗೆ ತಾಪಮಾನ ಪ್ರತಿಕ್ರಿಯೆ ವಕ್ರರೇಖೆಗಳನ್ನು ಅಳವಡಿಸಲಾಗಿದೆ, ಪ್ರತಿಕೃತಿ ಪ್ರತ್ಯೇಕ ಮರದ ಮಾಪನಗಳನ್ನು ಸಂಯೋಜಿಸಲಾಗಿದೆ (ಚಿತ್ರ ೨, ಫಲಕ ೧). ಪ್ರಭೇದಗಳ ಸರಾಸರಿ T_{opt} ಅನ್ನು ಪ್ರತ್ಯೇಕ ಮರದ ಪ್ರತಿಕೃತಿ ದತ್ತಾಂಶಕ್ಕೆ ಪ್ರತ್ಯೇಕವಾಗಿ ವಕ್ರಾಕೃತಿಗಳನ್ನು ಅಳವಡಿಸುವ ಮೂಲಕ ಲೆಕ್ಕಹಾಕಲಾಗಿದೆ. ಇದಕ್ಕೆ ವ್ಯತಿರಿಕ್ತವಾಗಿ, ಚಿತ್ರ ೨ ರಲ್ಲಿ ತೋರಿಸಿರುವ ವಕ್ರಾಕೃತಿಗಳು, ಫಲಕ ೧ ಎಲ್ಲಾ ಪ್ರತಿಕೃತಿ ಮರದ ದತ್ತಾಂಶವನ್ನು ಒಟ್ಟುಗೂಡಿಸುವ ಮೂಲಕ ಪಡೆದ ಸಂಯೋಜಿತ ಗಣಿತೀಯ ಫಲನಗಳನ್ನು ಪ್ರತಿನಿಧಿಸುತ್ತದೆ. ಪತ್ರ-ರಂಧ್ರ ವಾಹಕತೆ (g_s) ಮೇಲೆ ತಾಪಮಾನದ ಪರಿಣಾಮವನ್ನು ಪರೀಕ್ಷಿಸಲು, ದ್ವಿಘಾತ ಪದವನ್ನು ಹೊಂದಿರುವ ಮತ್ತು ಇಲ್ಲದ ರೇಖೀಯ ಮಾದರಿಗಳನ್ನು Akaike ಮಾಹಿತಿ ಮಾನದಂಡವನ್ನು (Cavanaugh and Neath 2019) ಬಳಸಿಕೊಂಡು ಹೋಲಿಸಲಾಯಿತು ಮತ್ತು ಅತ್ಯುತ್ತಮ ಗಣಿತೀಯ ಫಲನ ಮಾದರಿಯನ್ನು ಬಳಸಲಾಯಿತು. ಗಮನಿಸಿದ ಕ್ರಿಯಾಪ್ರಕ್ರಿಯೆಗಳ ಪ್ರತಿಕ್ರಿಯೆಗಳ ವೈವಿಧ್ಯತೆಯನ್ನು ಉಲ್ಲೇಖ ಮಾಡಲು ನಾವು ಎಲೆಯ ತಾಪಮಾನ T_{leaf} ವಿರುದ್ಧ g_s ಆಲೇಖಾ ಚಿತ್ರಗಳನ್ನು ಪ್ರಸ್ತುತಪಡಿಸುತ್ತೇವೆ. ಎಲೆ ಮತ್ತು ಸುತ್ತಮುತ್ತಲಿನ ಗಾಳಿಯ ನಡುವಿನ ನೀರಿನ ಆವಿ ವಿನಿಮಯ ಸೇರಿದಂತೆ ಎಲೆಯ ತಾಪಮಾನದ ಪ್ರತಿಕ್ರಿಯೆಗಳು ಮತ್ತು ಪರಿಸರ ಅಂಶಗಳಿಂದ g_s ಪ್ರಭಾವಿತವಾಗಿರುವುದರಿಂದ, ನಾವು T_{leaf} ಅನ್ನು ಪ್ರಾಥಮಿಕ ಮುನ್ಸೂಚಕವಾಗಿ ಬಳಸಿಕೊಂಡು g_s ಅನ್ನು ಮಾದರಿ ಮಾಡಿದ್ದೇವೆ. AIC ಮೌಲ್ಯವನ್ನು ಆಧರಿಸಿ ಉತ್ತಮ ದತ್ತಾಂಶ ಗಣಿತೀಯ ಫಲನ ರೇಖೀಯ ಅಥವಾ ದ್ವಿಘಾತ ಸಂಬಂಧಗಳನ್ನು ಆಯ್ಕೆ ಮಾಡಲಾಗಿದೆ. ತಾಪಮಾನದ ಇಳಿಜಾರುಗಳಲ್ಲಿ ಎಲೆಯಿಂದ ಗಾಳಿಗೆ ಆವಿ ಒತ್ತಡದ ಕೊರತೆಯಲ್ಲಿ (*VPD*) ಅನುಗುಣವಾದ ಬದಲಾವಣೆಗಳನ್ನು ಲೆಕ್ಕಹಾಕಲು, ನಾವು *VPD* ಮತ್ತು T_{leaf} ನಡುವೆ ರೇಖೀಯ ಹಿಂಜರಿತ ಮಾದರಿಯನ್ನು ಅಳವಡಿಸಿದ್ದೇವೆ. ಈ ಮಾದರಿಯನ್ನು ಬಳಸಿಕೊಂಡು, ನಾವು ಪ್ರತಿ T_{leaf} ಮಾಪನಕ್ಕಾಗಿ *VPD* ಮೌಲ್ಯಗಳನ್ನು ಲೆಕ್ಕ ಹಾಕಿದ್ದೇವೆ ಮತ್ತು ಅವುಗಳನ್ನು ದ್ವಿತೀಯ ಅಬ್ಸಿಸ್ಸಾ ಎಂದು ರೂಪಿಸಿದ್ದೇವೆ. ಚಿತ್ರ ೩ ಎಲೆ ಕೊಠಡಿಯಲ್ಲಿ ಸಾಪೇಕ್ಷ ಆರ್ದ್ರತೆ *RH* ನಿಯಂತ್ರಣದ ಮೂಲಕ ಸಾಧಿಸಿದ *VPD* ಮಟ್ಟವನ್ನು ಪ್ರದರ್ಶಿಸುತ್ತದೆ, ಆದರೆ ಪೂರಕ ಚಿತ್ರ ೧ ಅಧ್ಯಯನ ಸ್ಥಳದಲ್ಲಿ ದಾಖಲಾದ ದೈನಂದಿನ *VPD* ಏರಿಳಿತಗಳನ್ನು ವಿವರಿಸುತ್ತದೆ.

IRGA Li-6400 (Mott and Peak 2011; Still et al. 2019; Garen et al. 2022) ನಲ್ಲಿ ಸಾಮಾನ್ಯವಾಗಿ ಬಳಸುವ ಕಳಪೆ ವಿದ್ಯುತ್ ನಿರೋಧಿತ ತಾಪಸಂಧಿಳನ್ನು ಬಳಸಿಕೊಂಡು ಎಲೆಯ ತಾಪಮಾನ ಮಾಪನಗಳಲ್ಲಿನ ದೋಷಗಳ ಬಗ್ಗೆ ಹಲವಾರು ಅಧ್ಯಯನಗಳು ಕಳವಳಗಳನ್ನು ಎತ್ತಿ ತೋರಿಸುತ್ತವೆ. ಆದಾಗ್ಯೂ, Docherty ಮತ್ತು ಇತರರು (2023) ಹೆಚ್ಚಿನ ಪ್ರಮಾಣದ ಪಕ್ಷಪಾತ (Li-6400) ಮತ್ತು ತುಲನಾತ್ಮಕವಾಗಿ ಕಡಿಮೆ ಪಕ್ಷಪಾತ (Li-6800) ಹೊಂದಿರುವ ಉಪಕರಣವನ್ನು ಬಳಸಿಕೊಂಡು ಮಾಪನಗಳಿಂದ ಪಡೆದ ತಾಪಮಾನ ಪ್ರತಿಕ್ರಿಯೆ ನಿಯತಾಂಕಗಳಲ್ಲಿನ ವ್ಯತ್ಯಾಸಗಳನ್ನು ಪರೀಕ್ಷಿಸಿದರು ಮತ್ತು ಯಾವುದೇ ಸಂಖ್ಯಾಶಾಸ್ತ್ರೀಯ ವ್ಯತ್ಯಾಸವನ್ನು ಕಂಡುಹಿಡಿಯಲಿಲ್ಲ. ನಮ್ಮ ಅಧ್ಯಯನದಲ್ಲಿ, ಮಾಪನ ತಾಪಮಾನವು ಸಾಧಿಸಬಹುದಾದ ವ್ಯಾಪ್ತಿಯನ್ನು ವ್ಯಾಪಿಸಿರುವುದರಿಂದ ಮತ್ತು A_{net} ಸಾಮಾನ್ಯವಾಗಿ T_{opt} ನಲ್ಲಿ ಗರಿಷ್ಠ A_{net} ಅನ್ನು ಮೀರಿ ಕಡಿಮೆಯಾಗುವುದರಿಂದ, A_{opt} ನ ಸಂಪೂರ್ಣ ಮೌಲ್ಯಗಳು ಉಪಕರಣದ ತಾಪಮಾನ ಪಕ್ಷಪಾತದಿಂದ ಪ್ರಭಾವಿತವಾಗಬಾರದು. T_{opt} ನ ನಿಜವಾದ ಮೌಲ್ಯಗಳು ಹೆಚ್ಚು ನೇರವಾಗಿ ಪರಿಣಾಮ ಬೀರಬಹುದು ಎಂದು ನಾವು ಗುರುತಿಸಿದರೂ,

ಅದೇ ಉಪಕರಣವನ್ನು ಬಳಸಿಕೊಂಡು ಮಾಪನವನ್ನು ನಡೆಸಲಾಗಿರುವುದರಿಂದ ಸಂಗ್ರಹಿಸಿದ ದತ್ತಾಂಶದಲ್ಲಿ ಈ ಪರಿಣಾಮಗಳು ಹೋಲುತ್ತವೆ. ಆದ್ದರಿಂದ, ನಾವು ವರದಿ ಮಾಡುವ ಪ್ರಭೇದ-ವ್ಯಾಪಿ ವ್ಯತ್ಯಾಸಗಳನ್ನು ಎಚ್ಚರಿಕೆಯಿಂದ ಅರ್ಥೈಸಿಕೊಳ್ಳಬಹುದು.

೨.೪. । ಮೇಲ್ಮೈ ಮಣ್ಣಿನ ತೇವಾಂಶ

ನಂತರದ ವರ್ಷದಲ್ಲಿ (೨೦೨೩–೨೦೨೪) ಮೂರು TMT-4 Standard ದತ್ತಾಂಶ ಅಭಿಲೇಖಯಂತ್ರಗಳು (Wild et al. 2019) (TOMST, Prague, Czech Republic) ಸಂಭಾವ್ಯ ಸ್ಥಳಾಕೃತಿಯ ಸ್ತರಗಳಲ್ಲಿ ಮೇಲ್ಮೈ ಮಣ್ಣಿನ ತೇವಾಂಶದಲ್ಲಿ (ಮಣ್ಣಿನ ಮೇಲ್ಮೈಯಿಂದ ೮ ಸೆಂ.ಮೀ ಆಳದಲ್ಲಿ ೧೫ ನಿಮಿಷಗಳ ಅಂತರದಲ್ಲಿ) ಗಮನಾರ್ಹ ಋತು ಅವಲಂಬಿತ ವ್ಯತ್ಯಾಸಗಳು ಕಂಡುಬಂದವು. ಮಣ್ಣಿನ ಸ್ಥೂಲ ಸಾಂದ್ರತೆಯ ವ್ಯಾಪ್ತಿಯ ೧.೨೫–೧.೨೮ ಗ್ರಾಂ/ಸೆಂ.ಮೀ.^{೩} ಗಾಗಿ ಮಾಪನಾಂಕ ನಿರ್ಣಯ ಗುಣಾಂಕಗಳನ್ನು ವಾಚನಗಳನ್ನು ಪರಿಮಾಣಾತ್ಮಕ ಮಣ್ಣಿನ ತೇವಾಂಶವಾಗಿ ಪರಿವರ್ತಿಸಲು ಬಳಸಲಾಯಿತು. ಅಭಿಲೇಖಯಂತ್ರಗಳು ಬೆಟ್ಟದ ತುದಿ, ಇಳಿಜಾರು ಮತ್ತು ಕಣಿವೆ ಪ್ರದೇಶಗಳನ್ನು ಪ್ರತಿನಿಧಿಸುವ ಕಾರ್ಯತಂತ್ರವಾಗಿ ನೆಲೆಗೊಂಡಿದ್ದವು ಮತ್ತು ಮಣ್ಣಿನ ಮೇಲ್ಮೈಯಿಂದ ೮ ಸೆಂ.ಮೀ ಆಳದಲ್ಲಿ ೧೫ ನಿಮಿಷಗಳ ಆವರ್ತನದಲ್ಲಿ ದತ್ತಾಂಶವನ್ನು ಸೆರೆಹಿಡಿಯಲು ಹೊಂದಿಸಲಾಗಿತ್ತು.

ಮೇಲ್ಮೈ ಮಣ್ಣಿನ ತೇವಾಂಶದ ಮಟ್ಟಗಳು ಸಾಮಾನ್ಯವಾಗಿ ಬೆಟ್ಟದ ತುದಿಗಳಲ್ಲಿ ಕಡಿಮೆ, ನಂತರ ಇಳಿಜಾರುಗಳು ಮತ್ತು ಕಣಿವೆ ಪ್ರದೇಶಗಳಲ್ಲಿ ಅತ್ಯಧಿಕವಾಗಿರುತ್ತವೆ (ಚಿತ್ರ ೧, ಫಲಕ ೩ ಮತ್ತು ೪). ಎಲ್ಲಾ ಮೂರು ಸ್ತರಗಳಿಗೆ, ಮಣ್ಣಿನ ತೇವಾಂಶವು ಋತು ಅವಲಂಬಿತವಾಗಿ ಭಿನ್ನವಾಗಿತ್ತು ಮತ್ತು ಶುಷ್ಕ ಅವಧಿಗೆ ಹೋಲಿಸಿದರೆ ಆರ್ದ್ರ ಅವಧಿಯಲ್ಲಿ ಹೆಚ್ಚಿತ್ತು. ಕಣಿವೆ ಮತ್ತು ಇಳಿಜಾರಿನ ಸ್ಥಾನಗಳಲ್ಲಿ ಮೇಲ್ಮೈ ಮಣ್ಣಿನ ತೇವಾಂಶದಲ್ಲಿ ಅತಿದೊಡ್ಡ ಋತು ಅವಲಂಬಿತ ವ್ಯತ್ಯಾಸ ದಾಖಲಾಗಿದೆ, ಆದರೆ ಬೆಟ್ಟಗಳ ತುದಿಗಳಲ್ಲಿ ಇದಕ್ಕೆ ವಿರುದ್ಧವಾಗಿ, ಕಡಿಮೆ ಋತು ಅವಲಂಬಿತ ವ್ಯತ್ಯಾಸ ಕಂಡುಬಂದಿದೆ. ಕಣಿವೆ ಪ್ರದೇಶದಲ್ಲಿನ ಶುಷ್ಕ ಅವಧಿಯ ಮಣ್ಣಿನ ತೇವಾಂಶದ ಮಟ್ಟಗಳು ಆರ್ದ್ರ ಅವಧಿಯ ಇಳಿಜಾರಿನ ಮಟ್ಟಗಳಿಗೆ ಹೋಲಿಸಬಹುದು.

೨.೫. । ದತ್ತಾಂಶ ವಿಶ್ಲೇಷಣೆ

R ಆವೃತ್ತಿ ೪.೪.೩ (R Core Team, 2025) ರಲ್ಲಿ ದತ್ತಾಂಶ ವಿಶ್ಲೇಷಣೆಯನ್ನು ನಡೆಸಲಾಯಿತು. ತಾಪಮಾನ ಪ್ರತಿಕ್ರಿಯೆ ಕಾರ್ಯಕ್ಕಾಗಿ, A_{net} ತಾಪಮಾನ ಕಾರ್ಯವನ್ನು ಸಮೀಕರಣ ೧ ಕ್ಕೆ ಹೊಂದಿಸಲು ನಾವು 'stats' ಪ್ಯಾಕೇಜ್‌ನ 'nls' ಕಾರ್ಯವನ್ನು ಬಳಸಿದ್ದೇವೆ. T_{opt} ಮತ್ತು A_{opt} ಅನ್ನು ಅಳವಡಿಸಲಾದ ಮಾದರಿಗಳಿಂದ ಲೆಕ್ಕಹಾಕಲಾಗಿದೆ. ನಿಯತಾಂಕ ವಿಧಾನಗಳಲ್ಲಿನ ಪ್ರಭೇದ-ಮಟ್ಟದ ಋತು ಅವಲಂಬಿತ ವ್ಯತ್ಯಾಸಗಳನ್ನು ಜೋಡಿಯಾಗಿರುವ T-ಪರೀಕ್ಷೆಗಳನ್ನು ಬಳಸಿಕೊಂಡು ಪರೀಕ್ಷಿಸಲಾಗಿದೆ. T_{opt} ವ್ಯತ್ಯಾಸದಲ್ಲಿ A_{opt}, T_{opt}, ಮತ್ತು g_s ಅನ್ನು ವಿವರಿಸುವ ಎರಡು-ಮಾರ್ಗದ ಪುನರಾವರ್ತಿತ ಮಾಪನ ರೇಖೀಯ ಮಿಶ್ರ-ಪರಿಣಾಮದ ವಿಶ್ಲೇಷಣೆ (ANOVA) ಮಾದರಿಗಳನ್ನು 'nlme' ಪ್ಯಾಕೇಜ್‌ನಲ್ಲಿ 'lme' ಬಳಸಿಕೊಂಡು ಅಳವಡಿಸಲಾಗಿದೆ (Pinheiro et al. 2018). ಋತು, ಪ್ರಭೇದಗಳು ಮತ್ತು ಪರ್ಣ ಸ್ವಭಾವವನ್ನು (ಪರ್ಣಪಾತಿ ಮತ್ತು ನಿತ್ಯಹರಿದ್ವರ್ಣ) ಸ್ಥಿರ ಪರಿಣಾಮಗಳಾಗಿ ಸೇರಿಸಲಾಗಿದೆ ಮತ್ತು ಮಾದರಿಗಳಲ್ಲಿ ವೃಕ್ಷಗಳನ್ನು (ಮರಗಳು) ಯಾದೃಚ್ಛಿಕ ಪರಿಣಾಮವಾಗಿ ಸೇರಿಸಲಾಗಿದೆ. ಪೂರಕ ವಿಭಾಗ ೨ ಎಲ್ಲಾ ಮಾದರಿ ಹೊಂದಾಣಿಕೆಗಳನ್ನು ಪ್ರಸ್ತುತಪಡಿಸುತ್ತದೆ. ಎಲ್ಲಾ ವಿಧಾನಗಳನ್ನು ± SE ಗಳೊಂದಿಗೆ ಪ್ರಸ್ತುತಪಡಿಸಲಾಗುತ್ತದೆ ಮತ್ತು ೯೫% ವಿಶ್ವಾಸ ಮಧ್ಯಂತರಕ್ಕೆ (CI = ೯೫%, α = 0.0೫) ಗಮನಾರ್ಹ ಫಲಿತಾಂಶಗಳನ್ನು ಪ್ರಸ್ತುತಪಡಿಸಲಾಗುತ್ತದೆ.

೩ । ಫಲಿತಾಂಶಗಳು

೩.೧. । ದ್ಯುತಿಸಂಶ್ಲೇಷಕ $CO_೨$ ಉಪಗ್ರಹಣ ದರದ ಆದರ್ಶ ತಾಪಮಾನ

ಒಂಬತ್ತು ವೃಕ್ಷ ಪ್ರಭೇದಗಳ ಮೌಲ್ಯಗಳನ್ನು ಒಟ್ಟುಗೂಡಿಸಿ, $CO_೨$ ಉಪಗ್ರಹಣ ದರಕ್ಕೆ ಸರಾಸರಿ ಆದರ್ಶ ತಾಪಮಾನ (T_{opt}) ಆರ್ದ್ರ (೩೦.೪೩ ± ೦.೩೧ °C) ಮತ್ತು ಶುಷ್ಕ (೩೦.೯೩ ± ೦.೩೧ °C) ಅವಧಿಗಳ ನಡುವೆ ಯಾವುದೇ ಋತು ಅವಲಂಬಿತ ವ್ಯತ್ಯಾಸವನ್ನು ತೋರಿಸಲಿಲ್ಲ ($t_{೪೯}$ = ೦.೯೯, p=೦.೩೨೫). ಪ್ರಭೇದಗಳ ಸರಾಸರಿ T_{opt} ಆರ್ದ್ರ ಅವಧಿಯಲ್ಲಿ ಸರಾಸರಿ ಗರಿಷ್ಠ ವಾಯು ಉಷ್ಣತೆಗೆ ಹತ್ತಿರದಲ್ಲಿದೆ (ಪೂರಕ ಚಿತ್ರ ೩) ಆದರೆ ಶುಷ್ಕ ಅವಧಿಯಲ್ಲಿ, ಪ್ರಭೇದಗಳ ಸರಾಸರಿ T_{opt} ಸರಾಸರಿ ಗರಿಷ್ಠ ವಾಯು ಉಷ್ಣತೆಗಿಂತ ಸುಮಾರು ೩°C ಕಡಿಮೆಯಾಗಿದೆ. T_{opt} ವ್ಯತ್ಯಾಸಗಳು ಪ್ರಭೇದಗಳಿಗೆ ಗಮನಾರ್ಹವಾಗಿ ಸಂಬಂಧಿಸಿವೆ ($F_{೯}$ = ೮.೨೧, p < .೦೦೧), ಹೊರತು ಋತುವಿಗೆ ಅಲ್ಲ ($F_{೩}$ = ೩.೨, p = 0.02) ಹೆಚ್ಚುವರಿಯಾಗಿ, ಮಿಶ್ರ ಪರಿಣಾಮಗಳ ಮಾದರಿಯು ಪ್ರಭೇದಗಳು ಮತ್ತು ಋತುವಿನ ನಡುವೆ ಯಾವುದೇ ಗಮನಾರ್ಹ ಪರಸ್ಪರ ಕ್ರಿಯೆಯನ್ನು ತೋರಿಸಲಿಲ್ಲ ($F_{೯}$ = ೧.೮೧, p = ೦.೧೦೬) (ಪೂರಕ ವಿಭಾಗ ೨ ಮತ್ತು ಚಿತ್ರ ೩).

೩.೨. । ಆದರ್ಶ ತಾಪಮಾನದಲ್ಲಿ $CO_೨$ ಉಪಗ್ರಹಣ ದರ (A_{opt})

ಪ್ರಭೇದ-ಮಟ್ಟದ A_{net} ತಾಪಮಾನ ಪ್ರತಿಕ್ರಿಯೆ ವಕ್ರಾಕೃತಿಗಳನ್ನು ಚಿತ್ರ ೨, ಫಲಕ ೧ ನಲ್ಲಿ ಪ್ರಸ್ತುತಪಡಿಸಲಾಗಿದೆ. ಮಾಪನ ಮಾಡಲಾದ ಎಲ್ಲಾ ಒಂಬತ್ತು ಪ್ರಭೇದಗಳನ್ನು ಸಂಯೋಜಿಸುವ ಸರಾಸರಿ A_{opt}, ಆರ್ದ್ರ ಮತ್ತು ಶುಷ್ಕ ಅವಧಿಗಳಲ್ಲಿ ಕ್ರಮವಾಗಿ ೯.೬೮ ± ೦.೫೯ ಮತ್ತು ೯.2೧ ± ೦.೮೩ μmol $CO_೨$ $m^{-೨}$ $s^{-೧}$ ನಲ್ಲಿ ಹೋಲುತ್ತದೆ, ಮತ್ತು ಋತು ಅವಲಂಬಿತ ವ್ಯತ್ಯಾಸವಿಲ್ಲ. A_{opt} ಗಾಗಿ ಮಿಶ್ರ ಪರಿಣಾಮಗಳ ಮಾದರಿಯು ಗಮನಾರ್ಹ ಪ್ರಭೇದಯ ಪರಿಣಾಮವನ್ನು ತೋರಿಸಿದೆ ($F_{೮}$ = ೨೪.೮, p < .೦೦೧) ಆದರೆ ಯಾವುದೇ ಋತುವಿನ ಪರಿಣಾಮಗಳಿಲ್ಲ ($F_{೧}$ = ೦.೦೦೩, p = ೦.೯೫). ಆದಾಗ್ಯೂ, ಋತು ಮತ್ತು ಪ್ರಭೇದಗಳ ಸಂವಾದಾತ್ಮಕ ಪರಿಣಾಮ ($F_{೮}$ = ೮.೯, p < .೦೦೧) ಗಮನಾರ್ಹವಾಗಿತ್ತು, ಇದು A_{opt} ನಲ್ಲಿ ಋತು ಅವಲಂಬಿತ ವ್ಯತ್ಯಾಸಗಳು ಪ್ರಭೇದಗಳ ಮೇಲೆ ಅವಲಂಬಿತವಾಗಿವೆ ಎಂದು ಸೂಚಿಸುತ್ತದೆ. ಎಲೆಯ ಎಲೆ ಋತುಧರ್ಮ ವನ್ನು ಸ್ಥಿರ ಪರಿಣಾಮವೆಂದು ಪರೀಕ್ಷಿಸುವಾಗ, ಪರ್ಣ ಸ್ವಭಾವ A_{opt} ಮೇಲೆ ಗಮನಾರ್ಹ ಪರಿಣಾಮವನ್ನು ತೋರಿಸಿದೆ ($F_{೧}$ = ೧೩.2, p < .೦೦೧) ಮತ್ತು ಇದು ಎಲೆ ಋತುಧರ್ಮ ಮತ್ತು ಋತುವಿನ ನಡುವಿನ ಪರಸ್ಪರ ಕ್ರಿಯೆ ($F_{೧}$ = ೧೦.೧, p = ೦.೦೦೨) ಯಲ್ಲೂ ಕೂಡ ಕಂಡು ಬಂದಿದೆ. ಆದಾಗ್ಯೂ, ಋತುವು ಮಾತ್ರ ಗಮನಾರ್ಹವಾದ ಮುನ್ಸೂಚಕವಾಗಿರಲಿಲ್ಲ

($F_{೧}$=0.00೩, p=0.೯೫). ಮಿಶ್ರ ಪರಿಣಾಮಗಳ ಮಾದರಿಗಳ ಫಲಿತಾಂಶಗಳು ಪೂರಕ ವಿಭಾಗ ೨ ರಲ್ಲಿವೆ.

ಪರ್ಣಪಾತಿ ಮತ್ತು ನಿತ್ಯಹರಿದ್ವರ್ಣ ವರ್ಗಗಳ ನಡುವೆ A_{opt} ಗಮನಾರ್ಹವಾಗಿ ಭಿನ್ನವಾಗಿತ್ತು ($F_{೧}$=೧೧.೯, p=0.00೧). ಎಲೆ ಋತುಧರ್ಮ (ಅಂದರೆ, ಪರ್ಣಪಾತಿ ಮತ್ತು ನಿತ್ಯಹರಿದ್ವರ್ಣ) ಪ್ರಭೇದಗಳನ್ನು ವರ್ಗೀಕರಿಸುವುದು ವಿಭಿನ್ನ ಮಾದರಿಗಳನ್ನು ಬಹಿರಂಗಪಡಿಸಿತು. ನಿರ್ದಿಷ್ಟವಾಗಿ ಹೇಳುವುದಾದರೆ, ಶುಷ್ಕ ಬೇಸಿಗೆಯ ಅವಧಿಗೆ (೯.೯೩ ± 0.೬೭ μ mol $CO_{೨}$ $m^{-೨}$ $s^{-೧}$) ಹೋಲಿಸಿದರೆ ಆರ್ದ್ರ (೧೩.೭೮ ± ೧.೧೭ μ mol $CO_{೨}$ $m^{-೨}$ $s^{-೧}$) ಅವಧಿಯಲ್ಲಿ ಹೆಚ್ಚಿನ A_{opt} ನೊಂದಿಗೆ ಪರ್ಣಪಾತಿ ಪ್ರಭೇದಗಳು ಗಮನಾರ್ಹವಾದ ಋತು ಅವಲಂಬಿತ ವ್ಯತ್ಯಾಸವನ್ನು ($t_{೧೪}$ = ೨.೮೫, p = 0.0೧೨) ತೋರಿಸಿದವು, ಆದರೆ ನಿತ್ಯಹರಿದ್ವರ್ಣಗಳು ಎರಡು ಋತುಗಳಲ್ಲಿ ಬದಲಾಗಲಿಲ್ಲ.

ಶುಷ್ಕ ಅವಧಿಯಲ್ಲಿ ನಿತ್ಯಹರಿದ್ವರ್ಣ ಮತ್ತು ಪರ್ಣಪಾತಿ ಪ್ರಭೇದಗಳ ನಡುವೆ A_{opt} ಅನ್ನು ಹೋಲಿಸಬಹುದಾದರೂ, ಪರ್ಣಪಾತಿ ಪ್ರಭೇದಗಳ A_{opt} (೧೩.೭೮ ± ೧.೧೭ μ mol $CO_{೨}$ $m^{-೨}$ $s^{-೧}$) ಆರ್ದ್ರ ಅವಧಿಯಲ್ಲಿ ನಿತ್ಯಹರಿದ್ವರ್ಣ ಪ್ರಭೇದಗಳಿಗಿಂತ (೭.೫೬ ± 0.೭೨ μ mol $CO_{೨}$ $m^{-೨}$ $s^{-೧}$) ಹೆಚ್ಚಿತ್ತು ($t_{೧೬}$ = ೪.೫೩, p < 0.000೧). ನಿತ್ಯಹರಿದ್ವರ್ಣ ಪ್ರಭೇದಗಳು ಅವುಗಳ ವಿಶಿಷ್ಟ ಸ್ಥಳಾಕೃತಿಯ ಸ್ಥಾನವನ್ನು ಅವಲಂಬಿಸಿರುವ ಎರಡು ವಿಭಿನ್ನ ಪ್ರತಿಕ್ರಿಯೆಗಳನ್ನು ಮತ್ತಷ್ಟು ತೋರಿಸಿದವು. ಆರ್ದ್ರ ಕಣಿವೆಗಳ ವಿಶಿಷ್ಟವಾದ ಮೂರು ನಿತ್ಯಹರಿದ್ವರ್ಣ ಪ್ರಭೇದಗಳಿಗೆ, A_{opt} ಶುಷ್ಕ (೬.೪೫ ± 0.೮೧ μ mol $CO_{೨}$ $m^{-೨}$ $s^{-೧}$) ಮತ್ತು ಆರ್ದ್ರ ಅವಧಿಗಳ ನಡುವೆ (೬.0೧ ± ೧.೧ μ mol $CO_{೨}$ $m^{-೨}$ $s^{-೧}$) ಋತು ಅವಲಂಬಿತವಾಗಿ ಭಿನ್ನವಾಗಿರಲಿಲ್ಲ. ಇದಕ್ಕೆ ವ್ಯತಿರಿಕ್ತವಾಗಿ, ಮೂರು ಇಳಿಜಾರಿನ ಸಂಯೋಜಿತ ನಿತ್ಯಹರಿದ್ವರ್ಣಗಳು ಆರ್ದ್ರ (೮.೯೫ ± 0.೭೪ μ mol $CO_{೨}$ $m^{-೨}$ $s^{-೧}$) ಕ್ಕೆ ಹೋಲಿಸಿದರೆ ಶುಷ್ಕ ಅವಧಿಯಲ್ಲಿ (೧೨.೩೬ ± 0.೪೭ μ mol $CO_{೨}$ $m^{-೨}$ $s^{-೧}$) ಹೆಚ್ಚಿನ A_{opt} ನೊಂದಿಗೆ ಋತು ಅವಲಂಬಿತವಾಗಿ ಗಮನಾರ್ಹವಾಗಿ ಭಿನ್ನವಾಗಿವೆ.

ಎಲೆ ಮಟ್ಟದ PAR ದತ್ತಾಂಶವು A_{opt} ವ್ಯತ್ಯಾಸವನ್ನು ವಿವರಿಸಲಿಲ್ಲ (p=0.೪೯). ಮೇಲ್ಮೈ ಮಣ್ಣಿನ ತೇವಾಂಶವು A_{opt} ($F_{೨}$=೧೧.೧೫, p=0.00೪೬) ಅನ್ನು ಋತು ಅವಲಂಬಿತ ಪರಿಣಾಮಗಳಿಲ್ಲದೆ ಗಮನಾರ್ಹವಾಗಿ ಪ್ರಭಾವಿಸಿತು, ಇದು ಪ್ರಭೇದಗಳ ನಡುವಿನ A_{opt} ವ್ಯತ್ಯಾಸಗಳನ್ನು ಭಾಗಶಃ ವಿವರಿಸುತ್ತದೆ. ಋತು ಅವಲಂಬಿತ ವ್ಯತ್ಯಾಸಗಳನ್ನು ಹೊಂದಿರುವ ಮೂರು ನಿತ್ಯಹರಿದ್ವರ್ಣ ಪ್ರಭೇದಗಳು ಹೆಚ್ಚಾಗಿ ಶುಷ್ಕ ಸ್ಥಳಗಳೊಂದಿಗೆ ಸಂಬಂಧ ಹೊಂದಿವೆ ಮತ್ತು ಬೆಟ್ಟದ ಇಳಿಜಾರುಗಳಲ್ಲಿ ಕಂಡುಬರುತ್ತವೆ. ಇದಕ್ಕೆ ವ್ಯತಿರಿಕ್ತವಾಗಿ, ಬೆಟ್ಟದ ತುದಿಗೆ ಸಂಬಂಧಿಸಿದ ಪರ್ಣಪಾತಿ ಪ್ರಭೇದಗಳು ಆರ್ದ್ರ ಅವಧಿಯಲ್ಲಿ ಹೆಚ್ಚಿನ A_{opt} ಅನ್ನು ತೋರಿಸಿದವು. ಒಟ್ಟಾರೆಯಾಗಿ, ಪರ್ಣಪಾತಿ ಪ್ರಭೇದಗಳು ಮತ್ತು ಇಳಿಜಾರುಗಳಲ್ಲಿ ಮೂರು ಶುಷ್ಕ-ಸಂಬಂಧಿತ ನಿತ್ಯಹರಿದ್ವರ್ಣ ಪ್ರಭೇದಗಳು A_{opt} ನಲ್ಲಿ ಗಮನಾರ್ಹ ಮತ್ತು ವ್ಯತಿರಿಕ್ತ ಋತು ಅವಲಂಬಿತ ವ್ಯತ್ಯಾಸಗಳನ್ನು ತೋರಿಸಿದವು.

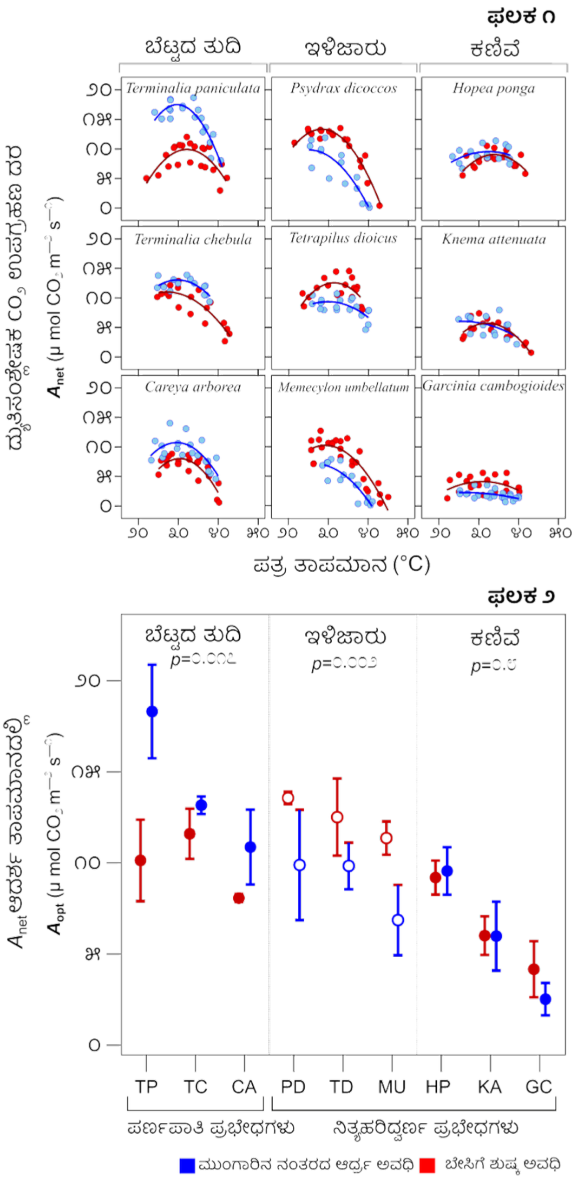

ಚಿತ್ರ ೨: ಭಾರತದ ಪಶ್ಚಿಮ ಘಟ್ಟಗಳಲ್ಲಿರುವ ೯ ವೃಕ್ಷ ಪ್ರಭೇದಗಳಿಗೆ ದ್ಯುತಿಸಂಶ್ಲೇಷಕ $CO_{೨}$ ಉಪಗ್ರಹಣ ದರ ತಾಪಮಾನ ಪ್ರತಿಕ್ರಿಯೆ ವಕ್ರಾಕೃತಿಗಳು (ಫಲಕ ೧, ೩x೩ ಕೋಷ್ಟಕ) ಸ್ಥಳದಲ್ಲೇ ಮಾಪನ ಮಾಡಲಾಗಿದೆ. ನೀಲಿ ಘಟಕಗಳು ಆರ್ದ್ರ ಅವಧಿಯನ್ನು ಪ್ರತಿನಿಧಿಸುತ್ತವೆ ಮತ್ತು ಕೆಂಪು ಘಟಕಗಳು ಶುಷ್ಕ ಅವಧಿಯನ್ನು ಸೂಚಿಸುತ್ತವೆ. ಫಲಕ ೨ ಪ್ರಭೇದಗಳ ಸರಾಸರಿ A_{opt} ಅನ್ನು ತೋರಿಸುತ್ತದೆ, ಇದು ಆದರ್ಶ ತಾಪಮಾನದಲ್ಲಿ ಗರಿಷ್ಠ ನಿವ್ವಳ $CO_{೨}$ ಉಪಗ್ರಹಣ ದರವಾಗಿದೆ (ಫಲಕ ೧ ಯಲ್ಲಿ ವಕ್ರಾಕೃತಿಗಳ ಶಿಖರಗಳು). ೧000 μmol $m^{-೨}$ $s^{-೧}$, ೪00 μmol $mol^{-೧}$ ನ [$CO_{೨}$] ವಿಕಿರಣದಲ್ಲಿ ಮಾಪನಗಳನ್ನು ನಡೆಸಲಾಯಿತು ಮತ್ತು *RH* ಅನ್ನು ೫0–೬0% ವ್ಯಾಪ್ತಿಯಲ್ಲಿ ನಿರ್ವಹಿಸಲಾಯಿತು. ಫಲಕ ೨ ರಲ್ಲಿ ತೆರೆದ ವೃತ್ತಗಳು A_{opt} ನಲ್ಲಿ ಗಮನಾರ್ಹ ಋತು ಅವಲಂಬಿತ ವ್ಯತ್ಯಾಸಗಳನ್ನು ಹೊಂದಿರುವ ನಿತ್ಯಹರಿದ್ವರ್ಣ ಪ್ರಭೇದಗಳನ್ನು ಸೂಚಿಸುತ್ತವೆ.

ಫಲಕ ೨ ರಲ್ಲಿ ತೋರಿಸಿರುವ ಪ್ರಭೇದಗಳ ಸಂಕೇತಾಕ್ಷರಗಳು ಈ ಕೆಳಗಿನಂತೆ ಆವರಣದಲ್ಲಿವೆ: *Terminalia paniculata* (TP), *Terminalia chebula* (TC), *Careya arborea* (CA), *Psydrax dicoccos* (PD), *Tetrapilus dioicus* (TD), *Memecylon umbellatum* (MU), *Hopea ponga* (HP), *Knema attenuata* (KA) and *Garcinia cambogiodes* var. *cambogioides* (GC)

೩.೩ । ಆದರ್ಶ ತಾಪಮಾನದಲ್ಲಿ ಪತ್ರರಂಧ್ರ ವಾಹಕತೆ ದರದಲ್ಲಿನ ವ್ಯತ್ಯಾಸಗಳು

ತಾಪಮಾನಕ್ಕೆ ಪತ್ರ-ರಂಧ್ರವಾಹಕತೆಯ ಪ್ರತಿಕ್ರಿಯೆಯು ಪ್ರಭೇದಗಳಲ್ಲಿ ಬದಲಾಗುತ್ತದೆ (ಚಿತ್ರ ೩). T_{opt} ನಲ್ಲಿ, g_s ಪ್ರಭೇದಗಳಲ್ಲಿ ಭಿನ್ನವಾಗಿರುತ್ತವೆ ($F_{೮}$=೩.೯, p=೦.೦೨೭) ಆದರೆ ಶುಷ್ಕ ಮತ್ತು ಆರ್ದ್ರ ಅವಧಿಗಳ ನಡುವೆ ಅಲ್ಲ, ಇದು ವಿಭಿನ್ನ ಪ್ರಭೇದಗಳ ಪ್ರತಿಕ್ರಿಯೆಗಳನ್ನು ಸೂಚಿಸುತ್ತದೆ. ಪರ್ಣಪಾತಿ ಬೆಟ್ಟದ ತುದಿಯ ಪ್ರಭೇದಗಳು ಶುಷ್ಕ ಅವಧಿಯಲ್ಲಿ ಕಡಿಮೆ g_s ಅನ್ನು ತೋರಿಸಿದವು, ಈ ಪ್ರಭೇದಗಳು ಶುಷ್ಕ ಅವಧಿಯಲ್ಲಿ ಪತ್ರ-ರಂಧ್ರನೀರಿನ ನಷ್ಟವನ್ನು ನಿಯಂತ್ರಿಸುತ್ತವೆ ಎಂದು ಸೂಚಿಸುತ್ತದೆ. ಕಣಿವೆ-ಸಂಬಂಧಿತ ಮೂರು ನಿತ್ಯಹರಿದ್ವರ್ಣ ಪ್ರಭೇದಗಳ g_s ಪ್ರತಿಕ್ರಿಯೆಯು ಋತು ಅವಲಂಬಿತವಾಗಿ ಭಿನ್ನವಾಗಿರಲಿಲ್ಲ. ಇಳಿಜಾರು-ಸಂಬಂಧಿತ ನಿತ್ಯಹರಿದ್ವರ್ಣ ಪ್ರಭೇದಗಳಿಗೆ, g_s ಪ್ರತಿಕ್ರಿಯೆ ಮಿಶ್ರಣವಾಗಿತ್ತು. ನಿರ್ದಿಷ್ಟವಾಗಿ ಹೇಳುವುದಾದರೆ, *Tetrapilus dioicus* ಯಾವುದೇ ಋತು ಅವಲಂಬಿತ ವ್ಯತ್ಯಾಸವನ್ನು ತೋರಿಸದಿದ್ದರೂ, ಇತರ ಪ್ರಭೇದಗಳು (*P. dicoccus* ಮತ್ತು *M. umbellatum*) ಶುಷ್ಕ ಅವಧಿಯಲ್ಲಿ ಹೆಚ್ಚಿನ g_s ಅನ್ನು ತೋರಿಸಿದವು (ವಿಶೇಷವಾಗಿ *P. dicoccus* ಲ್ಲಿ ಸ್ಪಷ್ಟವಾಗಿದೆ).

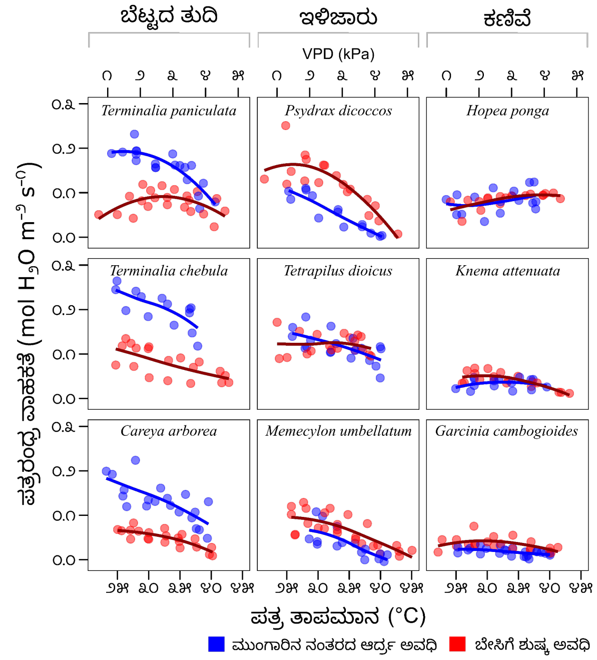

ಚಿತ್ರ ೩: ಭಾರತದ ಪಶ್ಚಿಮ ಘಟ್ಟಗಳಲ್ಲಿನ ಒಂಬತ್ತು ವೃಕ್ಷ ಪ್ರಭೇದಗಳಿಗೆ ನೀರಿಗೆ ಪತ್ರ-ರಂಧ್ರವಾಹಕತೆಯ (g_s) ತಾಪಮಾನದ ಪ್ರತಿಕ್ರಿಯೆಯನ್ನು ಮುಂಗಾರು ನಂತರದ ಆರ್ದ್ರ ಮತ್ತು ಬೇಸಿಗೆಯ ಶುಷ್ಕ ಅವಧಿಗಳಲ್ಲಿ ಮೂಲ ಸ್ಥಾನಿಕ ದಲ್ಲಿ ಮಾಪನ ಮಾಡ ಲಾಗುತ್ತದೆ. ದತ್ತಾಂಶ ಬಿಂದುವಿಗೆ ಅನುಗುಣವಾದ *VPD* ಅನ್ನು g_s, T_{leaf} ಮತ್ತು *VPD* ಮಾದರಿಯ ಆಧಾರದ ಮೇಲೆ ದ್ವಿತೀಯ x-ಅಕ್ಷದಲ್ಲಿ ಪ್ರತಿನಿಧಿಸಲಾಗುತ್ತದೆ. ವಕ್ರಾಕೃತಿಗಳು ರೇಖೀಯ ಅಥವಾ ದ್ವಿಘಾತ ಗಣಿತೀಯ ಫಲನಗಳಾಗಿವೆ (AIC ಆಧರಿಸಿ ಆಯ್ಕೆ ಮಾಡಲಾದ ಅತ್ಯುತ್ತಮ ಗಣಿತೀಯ ಫಲನ).

೪ । ಚರ್ಚೆ

ಭಾರತದ ಪಶ್ಚಿಮ ಘಟ್ಟಗಳಲ್ಲಿ ಸಹವರ್ತಿ ಉಷ್ಣವಲಯದ ಅರಣ್ಯ ವೃಕ್ಷ ಪ್ರಭೇದಗಳಲ್ಲಿ T_{opt} ನಲ್ಲಿ ಆದರ್ಶ ಉಷ್ಣ A_{opt} ಮತ್ತು g_s ನಲ್ಲಿ ದ್ಯುತಿಸಂಶ್ಲೇಷಣೆ ದರಗಳಲ್ಲಿನ ಋತು ಅವಲಂಬಿತ ವ್ಯತ್ಯಾಸಗಳ ಮೂಲ ಸ್ಥಾನಿಕಮಾಪನಗಳನ್ನು ನಾವು ನಡೆಸಿದ್ದೇವೆ. ನೀರು ಹೇರಳವಾಗಿದ್ದಾಗ ಹೆಚ್ಚಿನ ಪತ್ರ-ರಂಧ್ರ ವಾಹಕತೆ ದರಗಳಿಂದ ನಡೆಸಲ್ಪಡುವ ಆರ್ದ್ರ ಅವಧಿಯಲ್ಲಿ ಪರ್ಣಪಾತಿ ಪ್ರಭೇದಗಳು ನಿರಂತರವಾಗಿ ಹೆಚ್ಚಿನ A_{opt} ಅನ್ನು ತೋರಿಸಿದವು. ಇದಕ್ಕೆ ವ್ಯತಿರಿಕ್ತವಾಗಿ, ಪರೀಕ್ಷಿಸಿದ ನಿತ್ಯಹರಿದ್ವರ್ಣ ಪ್ರಭೇದಗಳಿಗೆ A_{opt} ಮತ್ತು g_s ನಲ್ಲಿನ ಋತು ಅವಲಂಬಿತ ಬದಲಾವಣೆಗಳು ಸ್ಥಳಾಕೃತಿಯ ಸ್ಥಾನವನ್ನು ಅವಲಂಬಿಸಿವೆ. ಪರ್ಣಪಾತಿ ಪ್ರಭೇದಗಳಲ್ಲಿ ಕಂಡುಬಂದದ್ದಕ್ಕೆ ವ್ಯತಿರಿಕ್ತವಾಗಿ, ಬೆಟ್ಟದ ಇಳಿಜಾರುಗಳಿಂದ ಬಂದ ನಿತ್ಯಹರಿದ್ವರ್ಣ ಪ್ರಭೇದಗಳು ಶುಷ್ಕ ಬೇಸಿಗೆಯ ಅವಧಿಯಲ್ಲಿ ಹೆಚ್ಚಿನ A_{opt} ಅನ್ನು ಹೊಂದಿದ್ದವು. ಶುಷ್ಕ ಋತುವಿನಲ್ಲಿಯೂ ಸಹ ನೀರಿನ ಮಿತಿಗಳನ್ನು ಅನುಭವಿಸದ ಕಣಿವೆಯ ನಿತ್ಯಹರಿದ್ವರ್ಣ ಪ್ರಭೇದಗಳು A_{opt} ಮತ್ತು g_s ನಲ್ಲಿ ಯಾವುದೇ ಋತು ಅವಲಂಬಿತ ಬದಲಾವಣೆಗಳನ್ನು ತೋರಿಸಲಿಲ್ಲ. ನಮ್ಮ ಫಲಿತಾಂಶಗಳು ಜಲ ಲಭ್ಯತೆ, ಸೂಕ್ಷ್ಮ ವಾತಾವರಣ ಪರಿಸ್ಥಿತಿಗಳು, ಪತ್ರ ಋತುಧರ್ಮ, ಪರ್ಣ ಸ್ವಭಾವಗಳು ಮತ್ತು ದ್ಯುತಿಸಂಶ್ಲೇಷಣೆಯ ಉಷ್ಣ ಒಗ್ಗಿಸುವಿಕೆಯಲ್ಲಿ ಋತು ಅವಲಂಬಿತ ದೀರ್ಘಕಾಲಿಕ ವಾತಾವರಣ ವ್ಯತ್ಯಾಸದ ಸಂಕೀರ್ಣ ಪರಿಣಾಮಗಳನ್ನು ಪ್ರದರ್ಶಿಸುತ್ತವೆ.

೪.೧ । ದ್ಯುತಿಸಂಶ್ಲೇಷಣೆಯ ಋತುಮಾನದ ಮೇಲೆ ಸ್ಥಳಾಕೃತಿ ಸ್ಥಾನದ ಪ್ರಭಾವ

ಪರೀಕ್ಷಿಸಿದ ಮೂರು ಆವಾಸಸ್ಥಾನಗಳಾದ ಬೆಟ್ಟದ ತುದಿಗಳು, ಇಳಿಜಾರುಗಳು ಮತ್ತು ಕಣಿವೆ ಪ್ರದೇಶಗಳಿಂದ ಮರಗಳ ಆಯ್ಕೆಯು ಸ್ಪಷ್ಟವಾಗಿ ಭಿನ್ನವಾಗಿತ್ತು (ಚಿತ್ರಗಳು ೪). ಭೂಗೋಳದಾದ್ಯಂತ A_{opt} ವ್ಯತ್ಯಾಸವು ಉಷ್ಣವಲಯ (Harris and Medina 2013) ಮತ್ತು ಸಮಶೀತೋಷ್ಣ ತಾಣಗಳಲ್ಲಿನ ಅಧ್ಯಯನಗಳಿಗೆ ಹೋಲುತ್ತದೆ (Tange 1996). ಇಳಿಜಾರಿನ ಉದ್ದಕ್ಕೂ ಮೇಲ್ಮೈ ಜಲ ಲಭ್ಯತೆಯು ಬದಲಾಗುತ್ತದೆ, ವಿಶೇಷವಾಗಿ ಶುಷ್ಕ ಋತುವಿನಲ್ಲಿ. ಮೇಲ್ಮೈ ಜಲ ಲಭ್ಯತೆಯು ಬೆಟ್ಟದ ತುದಿಯಲ್ಲಿ ಕಡಿಮೆ, ಬೆಟ್ಟದ ಇಳಿಜಾರಿನಲ್ಲಿ ಮಧ್ಯಂತರ ಮತ್ತು ಕಣಿವೆ ಪ್ರದೇಶಗಳಲ್ಲಿ ಅತ್ಯಧಿಕವಾಗಿದೆ. ಮೇಲ್ಮೈ ಮಣ್ಣಿನ ತೇವಾಂಶ ಮಾಪನದಿಂದ ನಮ್ಮ ಫಲಿತಾಂಶಗಳು ಬೆಟ್ಟದ ತುದಿಯ ಮಣ್ಣು ಎರಡು ಅವಧಿಗಳ ನಡುವೆ ಕಡಿಮೆ ಬದಲಾವಣೆಗೆ ಒಳಪಡುವ ನೀರಿನ ಮಟ್ಟಗಳಿಗೆ ಒಡ್ಡಿಕೊಳ್ಳುತ್ತದೆ ಎಂದು ಬಹಿರಂಗಪಡಿಸಿದೆ, ಆದರೆ ಇಳಿಜಾರುಗಳಲ್ಲಿನ ಮಣ್ಣಿನಲ್ಲಿ ಮತ್ತು ಕಣಿವೆ ಪ್ರದೇಶಗಳಲ್ಲಿ ನೀರು ಆರ್ದ್ರ ಅವಧಿಯಲ್ಲಿ ಗಮನಾರ್ಹವಾಗಿ ಹೆಚ್ಚಾಗುತ್ತದೆ. ಮೇಲ್ಮೈ ಮಣ್ಣಿನ ತೇವಾಂಶವು A_{opt} ಮೇಲೆ ಗಮನಾರ್ಹ ಪರಿಣಾಮವನ್ನು ತೋರಿಸಿದರೂ, ಸಂಬಂಧವು ಋತುಗಳ ಪರಿಣಾಮವನ್ನು ತೋರಿಸಲಿಲ್ಲ. ಇದು ಮತ್ತಷ್ಟು ಅನ್ವೇಷಿಸಬೇಕಾದ ಈ ಪ್ರಭೇದಗಳಿಗೆ ವೈವಿಧ್ಯಮಯ ಬೇರೂರಿಸುವಿಕೆ ಮತ್ತು ಋತು ಅವಲಂಬಿತವಾಗಿ ವಿಭಿನ್ನ ನೀರಿನ ಪ್ರವೇಶ ಆಳವನ್ನು ಸೂಚಿಸುತ್ತದೆ.

೪.೨ । ಕೆಲವು ನಿತ್ಯಹರಿದ್ವರ್ಣಗಳು ಮಳೆಗಾಲದ ಬದಲು ಉಷ್ಣತರ ಅವಧಿಗಳಲ್ಲಿ ಅತ್ಯುತ್ತಮವಾಗಿ ದ್ಯುತಿಸಂಶ್ಲೇಷಣೆ ಮಾಡುತ್ತವೆ.

ಸಹವರ್ತಿ ವೃಕ್ಷ ಪ್ರಭೇದಗಳಲ್ಲಿ ಆದರ್ಶ ತಾಪಮಾನದಲ್ಲಿ ದ್ಯುತಿಸಂಶ್ಲೇಷಣೆ ದರಗಳಲ್ಲಿ ಪ್ರಭೇದ-ವ್ಯಾಪಿ ಶುಷ್ಕ ಮತ್ತು ಆರ್ದ್ರ

ಅವಧಿಯ ವ್ಯತ್ಯಾಸಗಳನ್ನು ನಾವು ಕಂಡುಕೊಂಡಿದ್ದೇವೆ. ಹಿಂದಿನ ಅಧ್ಯಯನಗಳಿಗೆ ಅನುಗುಣವಾಗಿ, ಪರ್ಣಪಾತಿ ವೃಕ್ಷ ಪ್ರಭೇದಗಳ A_{opt} ಆರ್ದ್ರ ಅವಧಿಯಲ್ಲಿ ಹೆಚ್ಚಾಗಿತ್ತು, ಈ ಪ್ರಭೇದಗಳು ದ್ಯುತಿಸಂಶ್ಲೇಷಣೆಯನ್ನು ಗರಿಷ್ಠಗೊಳಿಸಲು ಆರ್ದ್ರ ಅವಧಿಗಳಲ್ಲಿ ಜಲ ಲಭ್ಯತೆಯನ್ನು ಬಳಸಿಕೊಳ್ಳುತ್ತವೆ ಎಂದು ಸೂಚಿಸುತ್ತದೆ (Eamus et al. 1999; Craven et al. 2011). ಅದೇ ರೀತಿ, ಪನಾಮ ಮತ್ತು ಆಸ್ಟ್ರೇಲಿಯಾದಲ್ಲಿ ನಿತ್ಯಹರಿದ್ವರ್ಣ ಮತ್ತು ಪರ್ಣಪಾತಿ ಪ್ರಭೇದಗಳನ್ನು ಒಳಗೊಂಡ ಮೊಳಕೆ ಅಧ್ಯಯನಗಳು ಆರ್ದ್ರ ಅವಧಿಗಳಲ್ಲಿ ಹೆಚ್ಚಿನ ದ್ಯುತಿಸಂಶ್ಲೇಷಣೆ ದರಗಳನ್ನು ವರದಿ ಮಾಡಿವೆ (Montagu and Woo 1999; Craven et al. 2011).

ಇದಕ್ಕೆ ವ್ಯತಿರಿಕ್ತವಾಗಿ, ನಿತ್ಯಹರಿದ್ವರ್ಣ ಪ್ರಭೇದಗಳು ಎರಡು ವಿಭಿನ್ನ ಪ್ರತಿಕ್ರಿಯೆಗಳನ್ನು ಪ್ರದರ್ಶಿಸಿದವು. Cai ಮತ್ತು ಇತರರ (2009) ಸಂಶೋಧನೆಗಳಿಗೆ ಹೋಲುವ ಮೂರು ನಿತ್ಯಹರಿದ್ವರ್ಣ ಪ್ರಭೇದಗಳ A_{opt} ಶುಷ್ಕ ಮತ್ತು ಆರ್ದ್ರ ಅವಧಿಗಳಿಗೆ ಒಂದೇ ಆಗಿತ್ತು. ಈ ಪ್ರಭೇದಗಳು ಶುಷ್ಕ ಋತುವಿನಲ್ಲಿಯೂ ಸಹ ಸ್ಥಳಾಕೃತಿ ವರ್ಗಗಳಲ್ಲಿ ಅತ್ಯಧಿಕ ಜಲ ಲಭ್ಯತೆಯನ್ನು ಹೊಂದಿದ್ದವು (ಚಿತ್ರ ೧) ಎಂಬ ಅಂಶದಿಂದ ಇದು ಆಶ್ಚರ್ಯವೇನಿಲ್ಲ. ಆದಾಗ್ಯೂ, ನಮ್ಮ ನಿರೀಕ್ಷೆಗೆ ವಿರುದ್ಧವಾಗಿ, ಇತರ ಮೂರು ನಿತ್ಯಹರಿದ್ವರ್ಣ ಪ್ರಭೇದಗಳಿಗೆ, ಆರ್ದ್ರ ಅವಧಿಗಿಂತ ಶುಷ್ಕ ಅವಧಿಯಲ್ಲಿ A_{opt} ಹೆಚ್ಚಿತ್ತು. ಈ ನಡವಳಿಕೆಯು ವೆನೆಜುವೆಲಾದ ಕಾಡುಗಳಿಂದ ಬಂದ ಸಂಶೋಧನೆಗಳಿಗೆ ಹೋಲುತ್ತದೆ, ಅಲ್ಲಿ ಕೆಲವು ನಿತ್ಯಹರಿದ್ವರ್ಣಗಳಲ್ಲಿ ಪ್ರಕಾಶ ಪೂರಿತ ದ್ಯುತಿಸಂಶ್ಲೇಷಣೆ ದರಗಳು ಶುಷ್ಕ ಅವಧಿಯಲ್ಲಿ ಹೆಚ್ಚಿದ್ದವು (Ávila-Lovera et al. 2019), ಆ ಸ್ಥಳದಲ್ಲಿ ಪರ್ಣಪಾತಿ ಸಸ್ಯಗಳಿಗೆ ಹೋಲಿಸಿದರೆ ನಿತ್ಯಹರಿದ್ವರ್ಣಗಳಲ್ಲಿ ಆಳವಾದ ಬೇರುಗಳನ್ನು ಶಂಕಿಸಲಾಗಿದೆ. ಆರ್ದ್ರ ಅವಧಿಗಳಲ್ಲಿ (Mujawamariya et al. 2023) ಹೆಚ್ಚಿನ ದ್ಯುತಿಸಂಶ್ಲೇಷಣೆಯ ಸಾಮಾನ್ಯ ಪ್ರವೃತ್ತಿಗೆ ವಿರುದ್ಧವಾಗಿ, ಈ ನಿತ್ಯಹರಿದ್ವರ್ಣ ಮರಗಳು ಉಷ್ಣತರ, ಶುಷ್ಕ ಪರಿಸ್ಥಿತಿಗಳಲ್ಲಿ ಬೆಳೆಯುತ್ತವೆ. ಸೀಮಿತ ಜಲ ಲಭ್ಯತೆಗೆ ಅವುಗಳ ಪ್ರತಿಕ್ರಿಯೆಗಳು, ಬಹುಶಃ ಆಳವಾದ ನೀರಿನ ಮೂಲಗಳ ಲಭ್ಯತೆಯಿಂದ (Nie et al. 2011) ಅಥವಾ ಉಷ್ಣ ಒಗ್ಗಿಕೊಳ್ಳುವಿಕೆಯಂತಹ ಇತರ ಕಾರ್ಯವಿಧಾನಗಳು, ಹೆಚ್ಚಿನ ಉಷ್ಣತರ ತಾಪ ಗುಣ ವಿಶಿಷ್ಟ ಕ್ಷೇತ್ರ ಆದ್ಯತೆ ಉತ್ತಮೀಕರಣ ಸೂಚಿಸುತ್ತವೆ.

ಹೆಚ್ಚಿನ ವಾರ್ಷಿಕ ಮಳೆ ಅಥವಾ ಕಡಿಮೆ ಶುಷ್ಕ ಅವಧಿಗಳನ್ನು ಹೊಂದಿರುವ ಕೆಲವು ಉಷ್ಣವಲಯದ ಕಾಡುಗಳಲ್ಲಿ, ದ್ಯುತಿಸಂಶ್ಲೇಷಕ ಚಟುವಟಿಕೆ (ನಿತ್ಯಹರಿದ್ವರ್ಣಗಳು ಸೇರಿದಂತೆ) ಕಡಿಮೆ ಋತು ಅವಲಂಬಿತವಾಗಿರುತ್ತದೆ ಅಥವಾ ಶುಷ್ಕ ಅವಧಿಗಳಲ್ಲಿ ಗರಿಷ್ಠವಾಗಿರುತ್ತದೆ ಎಂದು ಮಾಡೆಲಿಂಗ್ ಅಧ್ಯಯನಗಳು ತೋರಿಸುತ್ತವೆ (Uribe et al. 2021). ಭಾರತ ಮತ್ತು ಬ್ರೆಜಿಲ್‌ನ ಕೆಲವು ಮೊಳಕೆ ಅಧ್ಯಯನಗಳು ಶುಷ್ಕ ಅವಧಿಗಳಲ್ಲಿ ಹೆಚ್ಚಿನ ದ್ಯುತಿಸಂಶ್ಲೇಷಣೆ ದರಗಳನ್ನು ವರದಿ ಮಾಡಿವೆ (Ribeiro et al. 2009; Abhilash and Devakumar 2023). ದ್ಯುತಿಸಂಶ್ಲೇಷಣೆಗೆ ಪ್ರಾಕ್ಸಿಯಾಗಿ ಕ್ಲೋರೊಫಿಲ್ ಪ್ರತಿದೀಪಕತೆಯನ್ನು ಬಳಸುವ ಮತ್ತೊಂದು ಅಮೆಜೋನಿಯನ್ ಅಧ್ಯಯನ (Green et al. 2020) ಅಮೆಜೋನಿಯನ್ ಕಾಡಿನ ಕೆಲವು ಆರ್ದ್ರ ಭಾಗಗಳಲ್ಲಿ ಶುಷ್ಕ ಅವಧಿಗಳಲ್ಲಿ ಹೆಚ್ಚಿದ ದ್ಯುತಿಸಂಶ್ಲೇಷಣೆಯನ್ನು ಗಮನಿಸಿದೆ, ಈ ಅವಧಿಯಲ್ಲಿ ಕಿರಿಯ/ಹೊಸ ಎಲೆಗಳಿಗೆ ಪ್ರವೃತ್ತಿಗಳು ಕಾರಣವಾಗಿವೆ. ಇದಕ್ಕೆ ವಿರುದ್ಧವಾಗಿ ನಮ್ಮ ದತ್ತಾಂಶವು ಋತುಮಾನದ ವ್ಯತ್ಯಾಸಗಳು ಮತ್ತು ಗರಿಷ್ಠ ವಿಸ್ತಾರವಾದ ಎಲೆ ವಯಸ್ಸು ಮತ್ತು ಪರ್ಣ ಸ್ವಭಾವ ವರ್ಗಗಳಂತಹ ಸಂಪರ್ಕಗಳನ್ನು ತೋರಿಸುವುದಿಲ್ಲ, ಉದಾಹರಣೆಗೆ, ಶುಷ್ಕ-ಸಂಬಂಧದ ನಿತ್ಯಹರಿದ್ವರ್ಣ ಪ್ರಭೇದವಾದ *Memecylon umbellatum* ಶುಷ್ಕ ಅವಧಿಯಲ್ಲಿ ಸ್ವಲ್ಪ ಕಿರಿಯ ಎಲೆಗಳನ್ನು ಹೊಂದಿದ್ದರೆ, ಇತರ ಎರಡು ಶುಷ್ಕ-ಸಂಬಂಧದ ಪ್ರಭೇದಗಳು ಹಳೆಯ ಎಲೆಗಳನ್ನು ಹೊಂದಿದ್ದವು (ಪೂರಕ ಚಿತ್ರ ೪). ಈ ಬದಲಾವಣೆಯು ಎಲೆಯ ವಯಸ್ಸು (ಎಲೆ ಋತುಧರ್ಮ) ಹೆಚ್ಚಿನ ಶುಷ್ಕ ಅವಧಿಯ ದ್ಯುತಿಸಂಶ್ಲೇಷಣೆ ದರಗಳನ್ನು ಸಂಪೂರ್ಣವಾಗಿ ವಿವರಿಸುವುದಿಲ್ಲ ಎಂದು ಸೂಚಿಸುತ್ತದೆ. ಹೀಗಾಗಿ, ಎಲೆಯ ಮಟ್ಟದ ದ್ಯುತಿಸಂಶ್ಲೇಷಣೆಯಲ್ಲಿ ಶುಷ್ಕ ಅವಧಿಯ ಹೆಚ್ಚಳದಲ್ಲಿ ಒಳಗೊಂಡಿರುವ ಕಾರ್ಯವಿಧಾನಗಳಿಗೆ ಹೆಚ್ಚಿನ ಸಂಶೋಧನೆ ಅಗತ್ಯವಿದೆ.

ನಮ್ಮ ಅಧ್ಯಯನದಲ್ಲಿ ನಿತ್ಯಹರಿದ್ವರ್ಣಗಳಲ್ಲಿ A_{opt} ನಲ್ಲಿ ಋತುಮಾನದ ವ್ಯತ್ಯಾಸಗಳನ್ನು ಇಳಿಜಾರಿನ ಉದ್ದಕ್ಕೂ ತುಲನಾತ್ಮಕವಾಗಿ ಶುಷ್ಕ ಪ್ರದೇಶಗಳಲ್ಲಿ ಸಾಮಾನ್ಯವಾಗಿ ಕಂಡುಬರುವ ಮೂರು ಪ್ರಭೇದಗಳಿಗೆ ಮಾಪನ ಮಾಡ ಲಾಗಿದೆ (Pascal 1988; Krishnadas et al. 2021). ಈ ವಿಭಿನ್ನ ಸ್ಥಳಾಕೃತಿ ಸಂಬಂಧಗಳು ಈ ಪ್ರಭೇದಗಳಲ್ಲಿ ನೀರಿನ ಪ್ರವೇಶ ತಂತ್ರಗಳು ಮತ್ತು ಬೇರೂರಿಸುವ ಆಳದಲ್ಲಿನ ವ್ಯತ್ಯಾಸವನ್ನು ಸೂಚಿಸುತ್ತವೆ. ವರ್ಷವಿಡೀ ದ್ಯುತಿಸಂಶ್ಲೇಷಣೆಯನ್ನು ನಿರ್ವಹಿಸಲು ನಿತ್ಯಹರಿದ್ವರ್ಣ ಮರಗಳು ಹೆಚ್ಚಾಗಿ ಆಳವಾದ ನೀರಿನ ಪದರಗಳನ್ನು ಪ್ರವೇಶಿಸುತ್ತವೆ ಎಂದು ಅಧ್ಯಯನಗಳು ತೋರಿಸಿವೆ (Hasselquist et al. 2010; Brinkmann et al. 2019). ಆಳವಿಲ್ಲದ ಮಣ್ಣಿನ ಬೆಟ್ಟದ ತುದಿಗಳು, ಹೆಚ್ಚಿನ ಜಲ ಲಭ್ಯತೆಯೊಂದಿಗೆ ಆಳವಾದ ಮಣ್ಣಿನ ಪದರಗಳನ್ನು (Guha and Jain 2020) ಹೊಂದಿರುವ ಇಳಿಜಾರು ಮತ್ತು ಕಣಿವೆಗಳಿಗೆ ಹೋಲಿಸಿದರೆ ತುಲನಾತ್ಮಕವಾಗಿ ಸೀಮಿತ ಜಲ ಲಭ್ಯತೆಯನ್ನು ಹೊಂದಿವೆ ಎಂದು ನಾವು ಊಹಿಸಬಹುದು. ಒಟ್ಟಾರೆಯಾಗಿ, ಶುಷ್ಕ ಸೂಕ್ಷ್ಮ ವಾತಾವರಣಗಳೊಂದಿಗೆ ಸಂಬಂಧ ಹೊಂದಿರುವ ಕೆಲವು ನಿತ್ಯಹರಿದ್ವರ್ಣ ಪ್ರಭೇದಗಳು ಆರ್ದ್ರ ಅವಧಿಗಿಂತ ಶುಷ್ಕ ಮತ್ತು ಬೆಚ್ಚಗಿನ ಅವಧಿಯಲ್ಲಿ ಉಷ್ಣ ಆದರ್ಶ ತಾಪಮಾನದಲ್ಲಿ ಹೆಚ್ಚಿನ ದ್ಯುತಿಸಂಶ್ಲೇಷಣೆ ದರವನ್ನು ಸಾಧಿಸುತ್ತವೆ ಎಂದು ನಾವು ವಯಸ್ಕ ಮರಗಳ ಮೇಲಿನ ಮೂಲ ಸ್ಥಾನಿಕ ಮಾಪನಗಳಿಂದ ಪುರಾವೆಗಳನ್ನು ಪ್ರಕಾಶಿಸುತ್ತಿದ್ದೇವೆ.

೪.೩ । ಪತ್ರ-ರಂಧ್ರ ವಾಹಕತೆ ಋತು ಅವಲಂಬಿತ ವ್ಯತ್ಯಾಸಗಳು

ನಾವು ಅಧ್ಯಯನ ಮಾಡಿದ ಸಹವರ್ತಿ ಪ್ರಭೇದಗಳಲ್ಲಿ ತಾಪಮಾನಕ್ಕೆ (ಪ್ರಭೇದಗಳು ಮತ್ತು ಋತುಗಳಲ್ಲಿ) ಪತ್ರ-ರಂಧ್ರ ವಾಹಕತೆಯ ಪ್ರತಿಕ್ರಿಯೆಗಳು ಭಿನ್ನವಾಗಿವೆ ಎಂದು ನಾವು ಕಂಡುಕೊಂಡಿದ್ದೇವೆ (ಚಿತ್ರ ೩). ಒಂದು ಕಾರಣವೆಂದರೆ ಪ್ರತ್ಯೇಕ ಪ್ರಭೇದಗಳ ಕಾರ್ಯಕ್ಷಮತೆಯು ವಿಭಿನ್ನ ನೀರಿನ ಒತ್ತಡದ ಪರಿಸ್ಥಿತಿಗಳಿಂದ ಪ್ರಭಾವಿತವಾಗಿರುತ್ತದೆ (Schwartz et al. 2022), ನಮ್ಮ ಅಧ್ಯಯನದಲ್ಲಿ ಜಲ ಲಭ್ಯತೆಯಲ್ಲಿನ ಋತು ಅವಲಂಬಿತ ವ್ಯತ್ಯಾಸಗಳು, ಜಲ ಲಭ್ಯತೆಯಲ್ಲಿನ ದೀರ್ಘಕಾಲೀನ ವ್ಯತ್ಯಾಸಗಳಿಗೆ ದ್ಯೋತಕವಾಗಿ ಕಾರ್ಯನಿರ್ವಹಿಸುತ್ತವೆ. ಅಂತಹ ವ್ಯತ್ಯಾಸವು ಸೂಕ್ಷ್ಮ ವಾತಾವರಣಗಳಲ್ಲಿಯೂ ಕಂಡುಬರುತ್ತದೆ (Chitra-Tarak et al. 2021; Ding et al. 2021). ನೀರಿನ ಪ್ರವೇಶ ಮತ್ತು ದ್ಯುತಿಸಂಶ್ಲೇಷಕ ತಂತ್ರದಲ್ಲಿನ ಸ್ಥಳಾಕೃತಿ ಆಧಾರಿತ ವ್ಯತ್ಯಾಸಗಳು ಬೇಸಿಗೆಯ ಅವಧಿಯಲ್ಲಿ ನೀರಿನ ಕೊರತೆಯ ಪರಿಣಾಮದ ವಿರುದ್ಧ ಕೆಲವು ಪ್ರಭೇದಗಳನ್ನು ರಕ್ಷಣೆಮಾಡಬಹುದು (Esteban et al. 2021) .

g_s ಪ್ರತಿಕ್ರಿಯೆಯಲ್ಲಿ ಋತುಮಾನ ಮತ್ತು ಸ್ಥಳಾಕೃತಿಯ ವ್ಯತ್ಯಾಸಗಳನ್ನು ಬೆಂಬಲಿಸುತ್ತಾ, ಅಧ್ಯಯನ ಮಾಡಲಾದ ವೃಕ್ಷ ಪ್ರಭೇದಗಳಲ್ಲಿ ವೈವಿಧ್ಯಮಯ ತಾಪಮಾನ ಪ್ರತಿಕ್ರಿಯೆಗಳು ಹಾಗೂ ಋತು ಅವಲಂಬಿತ ವ್ಯತ್ಯಾಸಗಳನ್ನು ನಾವು ಕಾಣುತ್ತೇವೆ. ನಿರೀಕ್ಷೆಯಂತೆ ಎಲ್ಲಾ ಮೂರು ಪರ್ಣಪಾತಿ ಪ್ರಭೇದಗಳಿಗೆ, g_s ಸಾಮಾನ್ಯವಾಗಿ ತಾಪಮಾನದೊಂದಿಗೆ ಕಡಿಮೆಯಾಗುತ್ತಿತ್ತು, ಸಾಮಾನ್ಯವಾಗಿ ಶುಷ್ಕ ಅವಧಿಗೆ ಹೋಲಿಸಿದರೆ ಆರ್ದ್ರ ಅವಧಿಯಲ್ಲಿ ಹೆಚ್ಚಿನ g_s ತಲುಪುತ್ತಿತ್ತು, ಇದು ಅಮೆಜೋನಿಯಾದಲ್ಲಿನ ಋತು ಅವಲಂಬಿತ ಶುಷ್ಕ ಕಾಡಿನ ಅಧ್ಯಯನದಂತೆ (Vourlitis et al. 2008; Sendall et al. 2009) ಇದೆ. ಇದಕ್ಕೆ ವಿರುದ್ಧವಾಗಿ ನಿತ್ಯಹರಿದ್ವರ್ಣ ಪ್ರಭೇದಗಳು ಹೆಚ್ಚು ವೈವಿಧ್ಯಮಯ ಪ್ರತಿಕ್ರಿಯೆಗಳನ್ನು ಪ್ರದರ್ಶಿಸಿದವು. ಮೊದಲನೆಯದಾಗಿ, ನಿತ್ಯಹರಿದ್ವರ್ಣ ಪ್ರಭೇದಗಳಲ್ಲಿ ಶುಷ್ಕ ಮತ್ತು ಆರ್ದ್ರ ಅವಧಿಗಳ ನಡುವೆ g_s ಪ್ರತಿಕ್ರಿಯೆ ಸಾಮಾನ್ಯವಾಗಿ ಹೋಲುತ್ತದೆ, ಆದರೆ ಒಂದು ನಿತ್ಯಹರಿದ್ವರ್ಣ ಪ್ರಭೇದವಾದ *Psydrax dicoccos*, ಋತು ಅವಲಂಬಿತವಾಗಿ ವಿಭಿನ್ನ ಪ್ರತಿಕ್ರಿಯೆಯನ್ನು ತೋರಿಸಿತು ಮತ್ತು ಅಧ್ಯಯನ ಮಾಡಿದ ಇತರ ಪ್ರಭೇದಗಳಿಗೆ ವ್ಯತಿರಿಕ್ತವಾಗಿ ಶುಷ್ಕ ಅವಧಿಯಲ್ಲಿ ಹೆಚ್ಚಿನ g_s ಅನ್ನು ದಾಖಲಿಸಿತು. *Memecylon umbellatum*ನ g_s ಕೂಡ ಶುಷ್ಕ ಅವಧಿಯಲ್ಲಿ ಹೆಚ್ಚಿದ್ದರೂ, ಉಷ್ಣ ಗರಿಷ್ಠತೆಯಲ್ಲಿ ಆರ್ದ್ರ ಅವಧಿ g_s ನಿಂದ ಸಂಖ್ಯಾಶಾಸ್ತ್ರೀಯವಾಗಿ ನಗಣ್ಯ ವಾಗಿತ್ತು. ಮತ್ತೊಂದು ನಿತ್ಯಹರಿದ್ವರ್ಣ ಪ್ರಭೇದವಾದ *Hopea ponga*, ಋತುಮಾನಕ್ಕೆ ಅನುಗುಣವಾಗಿ ಅಸಡ್ಡೆ ಹೊಂದಿದ್ದರೂ, ತಾಪಮಾನದೊಂದಿಗೆ g_s ಹೆಚ್ಚಳವನ್ನು ದಾಖಲಿಸಿದೆ, ಬಹುಶಃ ಆವಿಯಾಗುವ ತಂಪಾಗಿಸುವಿಕೆಯ ಮೂಲಕ ಉಷ್ಣ ಸಂರಕ್ಷಣಾ ಕಾರ್ಯವಿಧಾನವನ್ನು ಸೂಚಿಸುತ್ತದೆ (Urban et al. 2017). ಈ ಪ್ರಭೇದವು ಹೆಚ್ಚಿನ ತಾಪಮಾನದೊಂದಿಗೆ A_{net} ಲ್ಲಿ ಇಳಿಕೆಯಿಲ್ಲದ ವಿಶಾಲವಾದ ತಾಪಮಾನ ಪ್ರತಿಕ್ರಿಯೆ ರೇಖೆಯನ್ನು (ಚಿತ್ರ ೧, ಫಲಕ ೧) ದಾಖಲಿಸಿದೆ ಮತ್ತು ಸುತ್ತಮುತ್ತಲಿನ ಪ್ರಬಲ ಪ್ರದೇಶಗಳಲ್ಲಿ ಕಂಡುಬಂದಿದೆ, ಬಹುಶಃ ಈ ಪ್ರಭೇದದಲ್ಲಿ ವೈವಿಧ್ಯಮಯ ನೀರಿನ ಬಳಕೆ ಮತ್ತು ಎಲೆ-ತಾಪಮಾನ ನಿಯಂತ್ರಣ ತಂತ್ರವನ್ನು ಸೂಚಿಸುತ್ತದೆ. ಥೈಲ್ಯಾಂಡ್‌ನ ತುಲನಾತ್ಮಕವಾಗಿ ಶುಷ್ಕ ಉಷ್ಣವಲಯದ ಕಾಡಿನಲ್ಲಿ ಮಾಪನ ಮಾಡ ಲಾದ *Hopea* (*H. ferrea*) ದ ವಿಭಿನ್ನ ಪ್ರಭೇದವು (Ishida et al. 2014) ಗಮನಾರ್ಹವಾದ ಶುಷ್ಕ-ಅವಧಿಯ ಕುಸಿತವನ್ನು ದಾಖಲಿಸಿದೆ ಎಂಬುದನ್ನು ಗಮನಿಸುವುದು ಆಸಕ್ತಿದಾಯಕವಾಗಿದೆ, ಅಲ್ಲಿ *H. ponga* ಶುಷ್ಕ ಮತ್ತು ಆರ್ದ್ರ ಅವಧಿಗಳಲ್ಲಿ ಅದೇ ದ್ಯುತಿಸಂಶ್ಲೇಷಣೆ ದರಗಳನ್ನು ಕಾಯ್ದುಕೊಂಡಿದೆ ಎಂದು ನಾವು ಕಂಡುಕೊಂಡಿದ್ದೇವೆ.

೪.೪. | ಎಲೆಗಳ ಋತು-ಧರ್ಮ, ಬೆಳಕಿನ ಲಭ್ಯತೆ ಮತ್ತು ಸೂಕ್ಷ್ಮವಾತಾವರಣ ವ್ಯತ್ಯಾಸ

ಅಮೆಜೋನಿಯನ್ ಅಧ್ಯಯನಗಳು ಪತ್ರ ಋತು-ಧರ್ಮವನ್ನು ದ್ಯುತಿಸಂಶ್ಲೇಷಣೆಯ ಋತು ಅವಲಂಬಿತ ಪ್ರವೃತ್ತಿಗಳಿಗೆ ಸಂಪರ್ಕಿಸುತ್ತವೆ (Wu et al. 2016; Chen et al. 2020). ಆದಾಗ್ಯೂ, ನಮ್ಮ ದತ್ತಾಂಶ (ಪೂರಕ ಚಿತ್ರ ೪) ದ್ಯುತಿಸಂಶ್ಲೇಷಕ ವ್ಯತ್ಯಾಸಗಳನ್ನು ವಿವರಿಸುವಲ್ಲಿ ಪತ್ರ ಋತು-ಧರ್ಮ ದುರ್ಬಲ ಪಾತ್ರವನ್ನು ಮಾತ್ರ ಸೂಚಿಸುತ್ತದೆ. ಗಮನಾರ್ಹವಾಗಿ, ಬೇಸಿಗೆಯ (ಶುಷ್ಕ) ಅವಧಿಯಲ್ಲಿ ಪರ್ಣಪಾತಿ ಮರಗಳ ಎಲೆಗಳು ಕಿರಿಯವಾಗಿದ್ದವು (ಪೂರಕ ಚಿತ್ರ ೪). ಎಳೆಯ ಎಲೆಗಳ ಉಪಗ್ರಹಣ ದರಗಳು ಸಾಮಾನ್ಯವಾಗಿ ಪ್ರೌಢ ಎಲೆಗಳಿಗಿಂತ ಹೆಚ್ಚಾಗಿರುತ್ತದೆ (ಉದಾಹರಣೆಗೆ Green ಮತ್ತು ಇತರರು, (2020)), ನಮ್ಮ ಅಧ್ಯಯನದಲ್ಲಿ ಪರ್ಣಪಾತಿ ಪ್ರಭೇದಗಳ A_{opt} ಮೌಲ್ಯಗಳು ಶುಷ್ಕ ಅವಧಿಗೆ ಹೋಲಿಸಿದರೆ ಎಲೆಗಳು ಹಳೆಯದಾಗಿದ್ದಾಗ ಆರ್ದ್ರ ಅವಧಿಯಲ್ಲಿ ಹೆಚ್ಚಾಗಿದ್ದವು, ಇದು ಈ ಪ್ರಭೇದಗಳಿಗೆ ಎಲೆಗಳ ವಯಸ್ಸಿನ ಬದಲು ಜಲ ಲಭ್ಯತೆಯ ಬಲವಾದ ನಿಯಂತ್ರಣವನ್ನು ಸೂಚಿಸುತ್ತದೆ. ಪನಾಮದಲ್ಲಿ (Kitajima et al. 1997) ನಡೆಸಿದ ಅಧ್ಯಯನವು ಶುಷ್ಕ ಅವಧಿಗಳಲ್ಲಿ ಹೆಚ್ಚಿದ ದ್ಯುತಿಸಂಶ್ಲೇಷಣೆ (O_2 ವಿಕಸನ ದರ ಎಂದು ಮಾಪನ ಮಾಡ ಲಾಗುತ್ತದೆ) ವರದಿ ಮಾಡಿದೆ - ನೀರಿನ ಒತ್ತಡದ ಅನುಪಸ್ಥಿತಿಯಲ್ಲಿ ಹೆಚ್ಚಿನ ಬೆಳಕಿನ ಲಭ್ಯತೆಗೆ ಕಾರಣವಾಗಿದೆ - ನಮ್ಮ ಸಂಶೋಧನೆಗಳು ಈ ಮಾದರಿಯನ್ನು ವ್ಯತಿರಿಕ್ತಗೊಳಿಸುತ್ತವೆ. ನಮ್ಮ ಅಧ್ಯಯನದಲ್ಲಿ, ಮಾಪನಗಳ ಸಮಯದಲ್ಲಿ ಬಾಹ್ಯ IRGA ಸಂವೇದಕದ ಮೂಲಕ ದಾಖಲಿಸಲಾದ ಎಲೆ-ಮಟ್ಟದ PAR, A_{opt} ವ್ಯತ್ಯಾಸದೊಂದಿಗೆ ಯಾವುದೇ ಮಹತ್ವದ ಸಂಬಂಧವನ್ನು ತೋರಿಸಲಿಲ್ಲ. ಪರೀಕ್ಷಿಸಲಾದ ಪರಿಸರ ಅಸ್ಥಿರಗಳಲ್ಲಿ (ಬೆಳಕಿನ ಲಭ್ಯತೆ ಮತ್ತು ಮೇಲ್ಮೈ ಮಣ್ಣಿನ ತೇವಾಂಶ), ಮೇಲ್ಮೈ ಮಣ್ಣಿನ ತೇವಾಂಶ ಮಾತ್ರ ಉಷ್ಣ ಆದರ್ಶ ತಾಪಮಾನದಲ್ಲಿ ದ್ಯುತಿಸಂಶ್ಲೇಷಕ ದರಗಳಿಗೆ ಸೀಮಿತ ವಿವರಣಾತ್ಮಕ ಶಕ್ತಿಯನ್ನು ಪ್ರದರ್ಶಿಸಿತು.

A_{opt} ನಲ್ಲಿನ ವಿವಿಧ ಋತು ಅವಲಂಬಿತ ವ್ಯತ್ಯಾಸಗಳು ಬೇರೂರಿಸುವ ಆಳಕ್ಕೆ ಸಂಬಂಧಿಸಿವೆಯೇ ಎಂದು ಮತ್ತಷ್ಟು ಪರೀಕ್ಷಿಸಲು, ಮಧ್ಯಾಹ್ನ ಮತ್ತು ಮುಂಜಾನೆಯ ಎಲೆ ನೀರಿನ ಸಂಭಾವ್ಯ ದತ್ತಾಂಶದಲ್ಲಿನ ವ್ಯತ್ಯಾಸವನ್ನು ಪರೀಕ್ಷಿಸಲು ನಾವು ನಮ್ಮ ಅಧ್ಯಯನ ಕ್ಷೇತ್ರಕ್ಕಾಗಿ ಮುಕ್ತವಾಗಿ ಲಭ್ಯವಿರುವ ದತ್ತಾಂಶವನ್ನು ಬಳಸಿದ್ದೇವೆ (Gloor et al. 2023) ಮತ್ತು A_{opt} ಮೇಲೆ ಯಾವುದೇ ಪರಿಣಾಮಗಳು ಕಂಡುಬಂದಿಲ್ಲ. ಮೇಲ್ಮೈ ಮಣ್ಣಿನ ತೇವಾಂಶ ಮಾಪನಗಳು ಕೆಲವು ಒಳನೋಟಗಳನ್ನು ಒದಗಿಸುತ್ತವೆಯಾದರೂ, ಪ್ರಭೇದಗಳಲ್ಲಿ ಪರಿಣಾಮಕಾರಿ ಬೇರೂರಿಸುವ ಆಳ ವ್ಯತ್ಯಾಸ ಮತ್ತು ಬೇರಿನ ಆಳದ ಉದ್ದಕ್ಕೂ ಮಣ್ಣಿನ ತೇವಾಂಶವು ಋತು ಅವಲಂಬಿತ ನೀರಿನ ಪ್ರವೇಶವನ್ನು ಸಂಪೂರ್ಣವಾಗಿ ಅರ್ಥಮಾಡಿಕೊಳ್ಳಲು ಅವಶ್ಯಕವಾಗಿದೆ.

ಅಮೆಜೋನಿಯನ್ ಅಧ್ಯಯನಗಳು ಪತ್ರ ಋತು-ಧರ್ಮವನ್ನು ದ್ಯುತಿಸಂಶ್ಲೇಷಣೆಯ ಋತು ಅವಲಂಬಿತ ಪ್ರವೃತ್ತಿಗಳಿಗೆ ಸಂಪರ್ಕಿಸುತ್ತವೆ (Wu et al. 2016; Chen et al. 2020). ಆದಾಗ್ಯೂ, ನಮ್ಮ ದತ್ತಾಂಶ (ಪೂರಕ ಚಿತ್ರ ೪) ದ್ಯುತಿಸಂಶ್ಲೇಷಕ ವ್ಯತ್ಯಾಸಗಳನ್ನು ವಿವರಿಸುವಲ್ಲಿ ಪತ್ರ ಋತು-ಧರ್ಮ ದುರ್ಬಲ ಪಾತ್ರವನ್ನು ಮಾತ್ರ ಸೂಚಿಸುತ್ತದೆ. ಗಮನಾರ್ಹವಾಗಿ, ಬೇಸಿಗೆಯ (ಶುಷ್ಕ) ಅವಧಿಯಲ್ಲಿ ಪರ್ಣಪಾತಿ ಮರಗಳ ಎಲೆಗಳು ಕಿರಿಯವಾಗಿದ್ದವು (ಪೂರಕ ಚಿತ್ರ ೪). ಎಳೆಯ ಎಲೆಗಳ ಉಪಗ್ರಹಣ ದರಗಳು ಸಾಮಾನ್ಯವಾಗಿ ಪ್ರೌಢ ಎಲೆಗಳಿಗಿಂತ ಹೆಚ್ಚಾಗಿರುತ್ತದೆ (ಉದಾಹರಣೆಗೆ Green ಮತ್ತು ಇತರರು, (2020)), ನಮ್ಮ ಅಧ್ಯಯನದಲ್ಲಿ ಪರ್ಣಪಾತಿ ಪ್ರಭೇದಗಳ A_{opt} ಮೌಲ್ಯಗಳು ಶುಷ್ಕ ಅವಧಿಗೆ ಹೋಲಿಸಿದರೆ ಎಲೆಗಳು ಹಳೆಯದಾಗಿದ್ದಾಗ ಆರ್ದ್ರ ಅವಧಿಯಲ್ಲಿ ಹೆಚ್ಚಾಗಿದ್ದವು, ಇದು ಈ ಪ್ರಭೇದಗಳಿಗೆ ಎಲೆಗಳ ವಯಸ್ಸಿನ ಬದಲು ಜಲ ಲಭ್ಯತೆಯ ಬಲವಾದ ನಿಯಂತ್ರಣವನ್ನು ಸೂಚಿಸುತ್ತದೆ. ಪನಾಮದಲ್ಲಿ (Kitajima et al. 1997) ನಡೆಸಿದ ಅಧ್ಯಯನವು ಶುಷ್ಕ ಅವಧಿಗಳಲ್ಲಿ ಹೆಚ್ಚಿದ ದ್ಯುತಿಸಂಶ್ಲೇಷಣೆ (O_2 ವಿಕಸನ ದರ ಎಂದು ಮಾಪನ ಮಾಡ ಲಾಗುತ್ತದೆ) ವರದಿ ಮಾಡಿದೆ - ನೀರಿನ ಒತ್ತಡದ ಅನುಪಸ್ಥಿತಿಯಲ್ಲಿ ಹೆಚ್ಚಿನ

ಬೆಳಕಿನ ಲಭ್ಯತೆಗೆ ಕಾರಣವಾಗಿದೆ - ನಮ್ಮ ಸಂಶೋಧನೆಗಳು ಈ ಮಾದರಿಯನ್ನು ವ್ಯತಿರಿಕ್ತಗೊಳಿಸುತ್ತವೆ. ನಮ್ಮ ಅಧ್ಯಯನದಲ್ಲಿ ಮಾಪನಗಳ ಸಮಯದಲ್ಲಿ ಬಾಹ್ಯ IRGA ಸಂವೇದಕದ ಮೂಲಕ ದಾಖಲಿಸಲಾದ ಎಲೆ-ಮಟ್ಟದ PAR, A_{opt} ವ್ಯತ್ಯಾಸದೊಂದಿಗೆ ಯಾವುದೇ ಮಹತ್ವದ ಸಂಬಂಧವನ್ನು ತೋರಿಸಲಿಲ್ಲ. ಪರೀಕ್ಷಿಸಲಾದ ಪರಿಸರ ಅಸ್ಥಿರಗಳಲ್ಲಿ (ಬೆಳಕಿನ ಲಭ್ಯತೆ ಮತ್ತು ಮೇಲ್ಮೈ ಮಣ್ಣಿನ ತೇವಾಂಶ), ಮೇಲ್ಮೈ ಮಣ್ಣಿನ ತೇವಾಂಶ ಮಾತ್ರ ಉಷ್ಣ ಆದರ್ಶ ತಾಪಮಾನದಲ್ಲಿ ದ್ಯುತಿಸಂಶ್ಲೇಷಕ ದರಗಳಿಗೆ ಸೀಮಿತ ವಿವರಣಾತ್ಮಕ ಶಕ್ತಿಯನ್ನು ಪ್ರದರ್ಶಿಸಿತು.

೪.೫.। ಬರ ಸಂವೇದನೆಯನ್ನು ಅರ್ಥಮಾಡಿಕೊಳ್ಳುವಲ್ಲಿನ ಪರಿಣಾಮಗಳು

ನಮ್ಮ ಫಲಿತಾಂಶಗಳು ಸಹವರ್ತಿ ವೃಕ್ಷ ಪ್ರಭೇದಗಳಲ್ಲಿ ವೈವಿಧ್ಯಮಯ ದ್ಯುತಿಸಂಶ್ಲೇಷಕ ಮತ್ತು ನೀರಿನ ಬಳಕೆಯ ತಂತ್ರಗಳನ್ನು ಬಹಿರಂಗಪಡಿಸುತ್ತವೆ, ಅವುಗಳ ಸ್ಥಳಾಕೃತಿಯ ಸ್ಥಾನದಿಂದ ಪ್ರಭಾವಿತವಾಗಿರುತ್ತದೆ. ಆರ್ದ್ರ-ಸಂಬಂಧದ ಕಣಿವೆಯ ನಿತ್ಯಹರಿದ್ವರ್ಣಗಳು ವರ್ಷಪೂರ್ತಿ ಆಳವಿಲ್ಲದ ಅಂತರ್ಜಲ ಪ್ರವೇಶದಿಂದ ಪ್ರಯೋಜನ ಪಡೆಯುವ ಸಾಧ್ಯತೆಯಿದೆ, ಆದರೆ ಬೆಟ್ಟದ ತುದಿಯ ಪರ್ಣಪಾತಿ ಪ್ರಭೇದಗಳು ಶುಷ್ಕ-ಅವಧಿಯ ನೀರಿನ ಒತ್ತಡಕ್ಕೆ ಹೆಚ್ಚು ದುರ್ಬಲವಾಗಿರಬಹುದು. ಶುಷ್ಕ-ಸಂಬಂಧದ ಇಳಿಜಾರಿನ ನಿತ್ಯಹರಿದ್ವರ್ಣಗಳು ಹೆಚ್ಚಿನ ಶುಷ್ಕ-ಅವಧಿಯ ದ್ಯುತಿಸಂಶ್ಲೇಷಕ ದರಗಳನ್ನು ತೋರಿಸಿವೆ. ಎಲ್ಲಾ ಪ್ರಭೇದಗಳು ನೀರಿನ ನಷ್ಟಕ್ಕೆ ಸಂಬಂಧಿಸಿದಂತೆ ಇಂಗಾಲದ ಲಾಭವನ್ನು ಅಂತರ್ಗತವಾಗಿ ಅತ್ಯುತ್ತಮವಾಗಿಸುವ ಪತ್ರ-ರಂಧ್ರ ನಿಯಂತ್ರಣವನ್ನು ಪ್ರದರ್ಶಿಸುತ್ತವೆ (Andriyas et al. 2021), ನೀರು-ಸೀಮಿತ ಪರಿಸ್ಥಿತಿಗಳಲ್ಲಿ ಹೆಚ್ಚು ಪರಿಣಾಮಕಾರಿ ಉತ್ತಮೀಕರಣ ಸಕ್ರಿಯಗೊಳಿಸುವ ಗುಣಲಕ್ಷಣಗಳನ್ನು ಹೊಂದಿರುವವರು ಹೆಚ್ಚು ಬರ ಸಹಿಷ್ಣುರಾಗಿರುತ್ತಾರೆ. ಆದಾಗ್ಯೂ, ಬರ ಸಹಿಷ್ಣುತೆಯು ಪತ್ರ-ರಂಧ್ರ ನಡವಳಿಕೆಯನ್ನು ಮೀರಿದ ಇತರ ಕ್ರಿಯಾಪ್ರಕ್ರಿಯೆಗಳ ಅಥವಾ ಪರಿಸರ ರೂಪಾಂತರಗಳನ್ನು ಸಹ ಒಳಗೊಂಡಿರಬಹುದು ಎಂಬುದನ್ನು ಗಮನಿಸುವುದು ಮುಖ್ಯ, ವಿಶೇಷವಾಗಿ ತೀವ್ರ ಪರಿಸರದಲ್ಲಿ ಅಥವಾ ಸಮುದಾಯ ಮಟ್ಟದಲ್ಲಿ (Blonder et al. 2023). ಪಶ್ಚಿಮ ಘಟ್ಟಗಳಲ್ಲಿ ಊಹಿಸಲಾದ ಹೆಚ್ಚುವರಿ ನೀರಿನ ಪರಿಸ್ಥಿತಿಗಳಲ್ಲಿ (Sarkar and Maity 2022), ಹೆಚ್ಚಿದ ದ್ಯುತಿಸಂಶ್ಲೇಷಕ ಲಾಭಗಳು ನೀರಿನ ಸಂರಕ್ಷಣೆಗಿಂತ ಒಲವು ತೋರಬಹುದು.

A_{opt} ನಲ್ಲಿನ ವಿವಿಧ ಋತು ಅವಲಂಬಿತ ವ್ಯತ್ಯಾಸಗಳು ಬೇರೂರಿಸುವ ಆಳಕ್ಕೆ ಸಂಬಂಧಿಸಿವೆಯೇ ಎಂದು ಮತ್ತಷ್ಟು ಪರೀಕ್ಷಿಸಲು, ಮಧ್ಯಾಹ್ನ ಮತ್ತು ಮುಂಜಾನೆಯ ಎಲೆ ನೀರಿನ ಸಂಭಾವ್ಯ ದತ್ತಾಂಶದಲ್ಲಿನ ವ್ಯತ್ಯಾಸವನ್ನು ಪರೀಕ್ಷಿಸಲು ನಾವು ನಮ್ಮ ಅಧ್ಯಯನ ಕ್ಷೇತ್ರಕ್ಕಾಗಿ ಮುಕ್ತವಾಗಿ ಲಭ್ಯವಿರುವ ದತ್ತಾಂಶವನ್ನು ಬಳಸಿದ್ದೇವೆ (Gloor et al. 2023) ಮತ್ತು A_{opt} ಮೇಲೆ ಯಾವುದೇ ಪರಿಣಾಮಗಳು ಕಂಡುಬಂದಿಲ್ಲ. ಮೇಲ್ಮೈ ಮಣ್ಣಿನ ತೇವಾಂಶ ಮಾಪನಗಳು ಕೆಲವು ಒಳನೋಟಗಳನ್ನು ಒದಗಿಸುತ್ತವೆಯಾದರೂ, ಪ್ರಭೇದಗಳಲ್ಲಿ ಪರಿಣಾಮಕಾರಿ ಬೇರೂರಿಸುವ ಆಳ ವ್ಯತ್ಯಾಸ ಮತ್ತು ಬೇರಿನ ಆಳದ ಉದ್ದಕ್ಕೂ ಮಣ್ಣಿನ ತೇವಾಂಶವು ಋತು ಅವಲಂಬಿತ ನೀರಿನ ಪ್ರವೇಶವನ್ನು ಸಂಪೂರ್ಣವಾಗಿ ಅರ್ಥಮಾಡಿಕೊಳ್ಳಲು ಅವಶ್ಯಕವಾಗಿದೆ.

ಜಲ ಲಭ್ಯತೆ, ಪ್ರಭೇದ-ನಿರ್ದಿಷ್ಟ ಲಕ್ಷಣಗಳು ಮತ್ತು ಸ್ಥಳಾಕೃತಿಯ ವ್ಯತ್ಯಾಸದ ನಡುವಿನ ಪರಸ್ಪರ ಕ್ರಿಯೆಯನ್ನು ಅರ್ಥಮಾಡಿಕೊಳ್ಳುವುದು ದೀರ್ಘಕಾಲಿಕ ವಾತಾವರಣ ಬದಲಾವಣೆಗೆ ಅರಣ್ಯ ಪ್ರತಿಕ್ರಿಯೆಗಳನ್ನು ಊಹಿಸಲು ನಿರ್ಣಾಯಕವಾಗಿದೆ. ಉದಾಹರಣೆಗೆ, ನೀರಿನ ಪ್ರವೇಶ ಮತ್ತು ದ್ಯುತಿಸಂಶ್ಲೇಷಕ ತಂತ್ರದಲ್ಲಿನ ಸ್ಥಳಾಕೃತಿ ಆಧಾರಿತ ವ್ಯತ್ಯಾಸಗಳು ಕೆಲವು ಪ್ರಭೇದಗಳನ್ನು ನೀರಿನ ಕೊರತೆಯಿಂದ ರಕ್ಷಿಸಬಹುದು (Esteban et al. 2021; Kühnhammer et al. 2023), ಇದು ಬರವು ಸಮುದಾಯದೊಳಗಿನ ಪ್ರಭೇದಗಳ ಮೇಲೆ ವಿಭಿನ್ನವಾಗಿ ಪರಿಣಾಮ ಬೀರುತ್ತದೆ ಎಂದು ಸೂಚಿಸುತ್ತದೆ. ಋತು ಅವಲಂಬಿತ ವ್ಯತ್ಯಾಸಗಳು ಭವಿಷ್ಯದ ತಾಪಮಾನ ಏರಿಕೆಯ ಪರಿಸ್ಥಿತಿಗಳನ್ನು ಸಂಪೂರ್ಣವಾಗಿ ಪುನರಾವರ್ತಿಸದಿದ್ದರೂ, ಅವು ಪ್ರಭೇದಗಳ ಕ್ರಿಯಾಪ್ರಕ್ರಿಯೆಗಳ ಲಕ್ಷಣ ಸಮಾಯೋಜ್ಯ ಮತ್ತು ನೀರಿನ ಒತ್ತಡಕ್ಕೆ ವೈವಿಧ್ಯಮಯ ಸಂವೇದನೆಯ ಬಗ್ಗೆ ಒಳನೋಟಗಳನ್ನು ಒದಗಿಸುತ್ತವೆ (Janssen et al. 2020).

ನಮ್ಮ ಸಂಶೋಧನೆಗಳು ಉಷ್ಣವಲಯದ ಅರಣ್ಯದಲ್ಲಿ ಬೆಳೆಯುವ ವಯಸ್ಕ ಮರಗಳಲ್ಲಿ, ವಿಶೇಷವಾಗಿ ನಿರ್ಣಾಯಕ ಬರ ಆಶ್ರಯಗಳನ್ನು ಒದಗಿಸಬಹುದಾದ ಆಳವಿಲ್ಲದ ನೀರಿನ ಮಟ್ಟಗಳಿಗೆ ಸಂಬಂಧಿಸಿರುವ ಋತು ಅವಲಂಬಿತ ಬರ ಸಹಿಷ್ಣುತೆ ಮತ್ತು ಸ್ಥಿತಿಸ್ಥಾಪಕತ್ವ ಕಾರ್ಯವಿಧಾನಗಳನ್ನು ಅರ್ಥಮಾಡಿಕೊಳ್ಳಲು ಕೊಡುಗೆ ನೀಡುತ್ತವೆ (Costa et al. 2023).

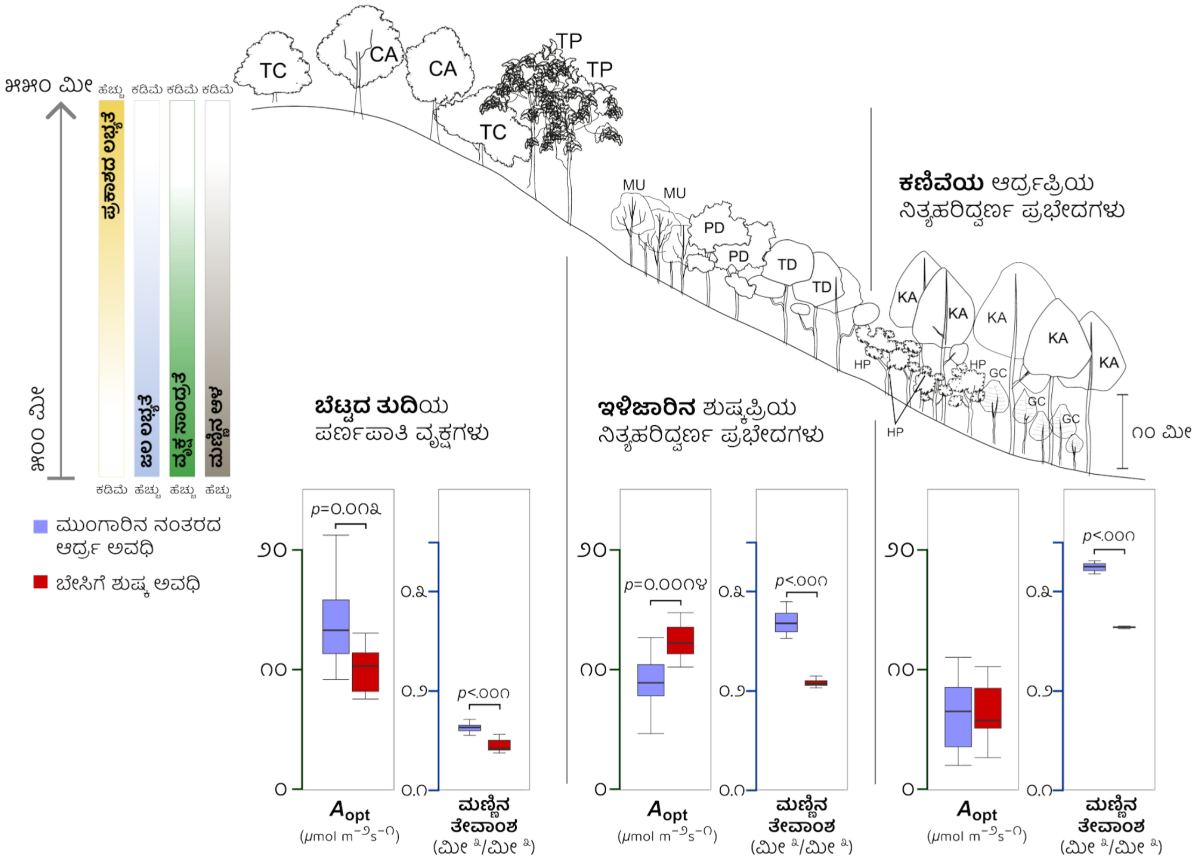

ಚಿತ್ರ ೪: ಭಾರತದ ಪಶ್ಚಿಮ ಘಟ್ಟಗಳಲ್ಲಿ ಅಧ್ಯಯನ ಮಾಡಲಾದ ವೃಕ್ಷ ಪ್ರಭೇದಗಳ ವಿತರಣೆ ಮತ್ತು ಆದರ್ಶ ತಾಪಮಾನ (ಹಸಿರು ಲಂಬಾಕ್ಷ) ಮತ್ತು ಮೇಲ್ಮೈ ಮಣ್ಣಿನ ತೇವಾಂಶದಲ್ಲಿ (ನೀಲಿ ಲಂಬಾಕ್ಷ, ಮೀ^{೩} ಮೀ$^{-೩}$ ನಲ್ಲಿ) ದ್ಯುತಿಸಂಶ್ಲೇಷಣೆ ದರಗಳಲ್ಲಿನ ವ್ಯತ್ಯಾಸಗಳನ್ನು ಆಯತ ಆಲೇಖಾ ಚಿತ್ರಗಳಾಗಿ ಚಿತ್ರಿಸಲಾಗಿದೆ. ಮೇಲಿನ ಎಡಭಾಗದಲ್ಲಿರುವ ಆಯತಗಳು ಬೆಟ್ಟದ ತುದಿಗಳಿಂದ ಕಣಿವೆ ಪ್ರದೇಶಗಳಿಗೆ ಮರದ ಸಾಂದ್ರತೆ, ವಿಕಿರಣ ಮಟ್ಟ ಮತ್ತು ಜಲ ಲಭ್ಯತೆಯ ಬದಲಾವಣೆಗಳಲ್ಲಿನ ಸಾಪೇಕ್ಷ ಬದಲಾವಣೆಯನ್ನು ಸೂಚಿಸುತ್ತವೆ. ಪ್ರಭೇದಗಳ ಸಂಕೇತಾಕ್ಷರಗಳು ಈ ಕೆಳಗಿನಂತೆ ಆವರಣದಲ್ಲಿವೆ: *Terminalia paniculata* (TP), *Terminalia chebula* (TC), *Careya arborea* (CA), *Psydrax dicoccos* (PD), *Tetrapilus dioicus* (TD), *Memecylon umbellatum* (MU), *Hopea ponga* (HP), *Knema attenuata* (KA) and *Garcinia cambogioides* var. *cambogioides* (GC).

೫ । ಸಾರಾಂಶ

ಭಾರತದ ಮಧ್ಯ ಪಶ್ಚಿಮ ಘಟ್ಟಗಳಲ್ಲಿನ ಋತು ಅವಲಂಬಿತವಾಗಿ ಶುಷ್ಕ ಉಷ್ಣವಲಯದ ಅರಣ್ಯ ಸ್ಥಳದಲ್ಲಿ ಶುಷ್ಕ ಬೇಸಿಗೆ ಮತ್ತು ಮುಂಗಾರು ನಂತರದ ಆರಂಭಿಕ ಆರ್ದ್ರ ಅವಧಿಗಳಲ್ಲಿ ಮಾಪನ ಮಾಡಲಾದ ದ್ಯುತಿಸಂಶ್ಲೇಷಣೆಯ ತಾಪಮಾನ ಪ್ರತಿಕ್ರಿಯೆಯು ಗಮನಾರ್ಹವಾದ ಅಂತರ-ನಿರ್ದಿಷ್ಟ ವ್ಯತ್ಯಾಸಗಳನ್ನು ಬಹಿರಂಗಪಡಿಸಿತು. ಬೆಟ್ಟದ ಇಳಿಜಾರುಗಳಲ್ಲಿ ವಿವಿಧ ಸ್ಥಾನಗಳಲ್ಲಿ ಕಂಡುಬರುವ ವೃಕ್ಷ ಪ್ರಭೇದಗಳಲ್ಲಿ ಆದರ್ಶ ತಾಪಮಾನ ಮತ್ತು ಪತ್ರ-ರಂಧ್ರ ವಾಹಕತೆಯಲ್ಲಿ CO_2 ಉಪಗ್ರಹಣ ದರಗಳಲ್ಲಿ ನಾವು ವಿಭಿನ್ನ ಮಾದರಿಗಳನ್ನು ಗಮನಿಸಿದ್ದೇವೆ. ಪರ್ಣಪಾತಿ ಪ್ರಭೇದಗಳು ಆರ್ದ್ರ ಅವಧಿಯಲ್ಲಿ ಹೆಚ್ಚಿನ ದ್ಯುತಿಸಂಶ್ಲೇಷಣೆ ದರಗಳನ್ನು ಪ್ರದರ್ಶಿಸುತ್ತವೆ, ಅವುಗಳ ವಿಶಿಷ್ಟ ಋತು ಅವಲಂಬಿತ ಮಾದರಿಯನ್ನು ಪ್ರತಿಬಿಂಬಿಸುತ್ತವೆ. ಇದಕ್ಕೆ ವ್ಯತಿರಿಕ್ತವಾಗಿ, ನಿತ್ಯಹರಿದ್ವರ್ಣ ಪ್ರಭೇದಗಳಿಗೆ ನಾವು ಎರಡು ಮಾದರಿಗಳನ್ನು ಕಂಡುಕೊಂಡಿದ್ದೇವೆ: ಕಣಿವೆ ಪ್ರಭೇದಗಳಿಗೆ ದ್ಯುತಿಸಂಶ್ಲೇಷಣೆ ದರಗಳು ಆರ್ದ್ರ ಮತ್ತು ಶುಷ್ಕ ಅವಧಿಯಲ್ಲಿ ಒಂದೇ ಆಗಿದ್ದವು, ಆದರೆ ಇಳಿಜಾರುಗಳಲ್ಲಿನ ನಿತ್ಯಹರಿದ್ವರ್ಣ ಪ್ರಭೇದಗಳಲ್ಲಿ ಆರ್ದ್ರ ಅವಧಿಗೆ ಹೋಲಿಸಿದರೆ ಶುಷ್ಕ ಅವಧಿಯಲ್ಲಿ ಅನಿರೀಕ್ಷಿತವಾಗಿ ಹೆಚ್ಚಿನ ದ್ಯುತಿಸಂಶ್ಲೇಷಣೆ ದರಗಳನ್ನು ಹೊಂದಿದ್ದವು, ಇದು ಬಹುಶಃ ಉಷ್ಣತರ ತಾಪಮಾನ ಆದ್ಯತೆಯನ್ನು ಸೂಚಿಸುತ್ತದೆ. ನಿತ್ಯಹರಿದ್ವರ್ಣಗಳು ಸೇರಿದಂತೆ ಸಹ-ಸಂಭವಿಸುವ ವೃಕ್ಷ ಪ್ರಭೇದಗಳು ಅವುಗಳ ಉಷ್ಣ ಆದರ್ಶ ತಾಪಮಾನದಲ್ಲಿ ದ್ಯುತಿಸಂಶ್ಲೇಷಣೆಯಲ್ಲಿ ವೈವಿಧ್ಯಮಯ ಋತು ಅವಲಂಬಿತ ವ್ಯತ್ಯಾಸಗಳನ್ನು ಪ್ರದರ್ಶಿಸುತ್ತವೆ ಎಂದು ನಮ್ಮ ಫಲಿತಾಂಶಗಳು ತೋರಿಸುತ್ತವೆ.

ಕೃತಜ್ಞತೆ

COVID-19 ಸಾಂಕ್ರಾಮಿಕ ಸಮಯದ ಸೇವೆಗಾಗಿ ಜಗತ್ತಿನಾದ್ಯಂತದ ಆರೋಗ್ಯ ಕಾರ್ಯಕರ್ತರ ಸಮುದಾಯಕ್ಕೆ ನಮ್ಮ ಧನ್ಯವಾದಗಳು. ಉಪ್ಸಾಲಾದಲ್ಲಿ ಪೋಸ್ಟ್‌ಡಾಕ್ಟರಲ್ ಅನುದಾನಕ್ಕಾಗಿ Wenner-Gren ಫೌಂಡೇಶನ್‌ಗೆ RT ಧನ್ಯವಾದಗಳನ್ನು ಅರ್ಪಿಸುತ್ತಾರೆ. ಮಾರ್ಗದರ್ಶನ ನೀಡಿದ ಶೃಂಗೇರಿಯ ದಕ್ಷಿಣಾಮ್ನಾಯ ಶ್ರೀ ಶಾರದಾ ಪೀಠದ ಶಾರದ-ಚಂದ್ರಮೌಳೀಶ್ವರ ಮತ್ತು ಉಭಯ ಜಗದ್ಗುರುಗಳಿಗೆ RT ಮಾಡುವ ವಿನಮ್ರ ಪ್ರಣಾಮಗಳು.

ಆಕರ

Abhilash KP, Devakumar AS (2023) Seasonal photosynthesis variations of dominant tree species used in different urban landscapes. International Journal of Environment and Climate Change 13: 562–571

Andriyas T, Leksungnoen N, Tor-Ngern P (2021) Comparison of water-use characteristics of tropical tree saplings with implications for forest restoration. Sci Rep 11: 1745

Asargew MF, Masutomi Y, Kobayashi K, Aono M (2024) Water stress changes the relationship between photosynthesis and stomatal conductance. Sci Total Environ 907: 11

Ávila-Lovera E, Urich R, Coronel I, Tezara W (2019) Seasonal gas exchange and resource-use efficiency in evergreen versus deciduous species from a tropical dry forest. Tree Physiol 39: 1561–1571

Blonder BW, Aparecido LMT, Hultine KR, Lombardozzi D, Michaletz ST, Posch BC, Slot M, Winter K (2023) Plant water use theory should incorporate hypotheses about extreme environments, population ecology, and community ecology. New Phytol 238: 2271–2283

Borjigidai A, Hikosaka K, Hirose T, Hasegawa T, Okada M, Kobayashi K (2006) Seasonal changes in temperature dependence of photosynthetic rate in rice under a free-air CO2 enrichment. Ann Bot 97: 549–557

Brinkmann N, Eugster W, Buchmann N, Kahmen A (2019) Species-specific differences in water uptake depth of mature temperate trees vary with water availability in the soil. Plant Biol (Stuttg) 21: 71–81

Cai Z-Q, Schnitzer SA, Bongers F (2009) Seasonal differences in leaf-level physiology give lianas a competitive advantage over trees in a tropical seasonal forest. Oecologia 161: 25–33

Carvalho NS, Anderson LO, Nunes CA, PessÃ´a ACM, Silva Junior CH, Reis JBC, Shimabukuro YE, Berenguer E, Barlow J, AragÃ£o LEO (2021) Spatio-temporal variation in dry season determines the Amazonian fire calendar. Environ Res Lett 16: 125009

Cavanaugh JE, Neath AA (2019) The Akaike information criterion: Background, derivation, properties, application, interpretation, and refinements. Wiley Interdiscip Rev Comput Stat 11: e1460

Chen X, Maignan F, Viovy N, Bastos A, Goll D, Wu J, Liu L, Yue C, Peng S, Yuan W, Conceição AC, O'Sullivan M, Ciais P (2020) Novel representation of leaf phenology improves simulation of amazonian evergreen forest photosynthesis in a land surface model. J Adv Model Earth Syst 12:

Chitra-Tarak R, Xu C, Aguilar S, Anderson-Teixeira KJ, Chambers J, Detto M, Faybishenko B, Fisher RA, Knox RG, Koven CD, Kueppers LM, Kunert N, Kupers SJ, McDowell NG, Newman BD, Paton SR, Pérez R, Ruiz L, Sack L, Warren JM, Wolfe BT, Wright C, Wright SJ, Zailaa J, McMahon SM (2021) Hydraulically-vulnerable trees survive on deep-water access during droughts in a tropical forest. New Phytol 231: 1798–1813

Choury Z, Wujeska-Klause A, Bourne A, Bown NP, Tjoelker MG, Medlyn BE, Crous KY (2022) Tropical rainforest species have larger increases in temperature optima with warming than warm-temperate rainforest trees. New Phytol 234: 1220–1236

Comita LS, Engelbrecht BMJ (2009) Seasonal and spatial variation in water availability drive habitat associations in a tropical forest. Ecology 90: 2755–2765

Corredor-Londoño G-A, Beltrán J-W, Torres-González A-M, Sardi-Saavedra A (2020) Phenological synchrony and seasonality of eight tree species in a fragmented landscape in the Colombian Andes. Revista de Biología Tropical 68: 987–1000

Costa FRC, Schietti J, Stark SC, Smith MN (2023) The other side of tropical forest drought: do shallow water table regions of Amazonia act as large-scale hydrological refugia from drought? New Phytol 237: 714–733

Craven D, Dent D, Braden D, Ashton MS, Berlyn GP, Hall JS (2011) Seasonal variability of photosynthetic characteristics influences growth of eight tropical tree species at two sites with contrasting precipitation in Panama. For Ecol Manage 261: 1643–1653

Crous KY, Uddling J, De Kauwe MG (2022) Temperature responses of photosynthesis and respiration in evergreen trees from boreal to tropical latitudes. New Phytol 234: 353–374

Cunningham SC, Read J (2002) Comparison of Temperate and Tropical Rainforest Tree Species: Photosynthetic Responses to Growth Temperature. Oecologia 133: 112–119

Das A, Nagendra H, Anand M, Bunyan M (2015) Topographic and Bioclimatic Determinants of the Occurrence of Forest and Grassland in Tropical Montane Forest-Grassland Mosaics of the Western Ghats, India. PLoS One 10: e0130566

Devi NL, Brearley FQ, Tripathi SK (2023) Phenological diversity among sub-tropical moist forest trees of north-eastern India. J Trop Ecol 39: e29

Ding Y, Nie Y, Chen H, Wang K, Querejeta JI (2021) Water uptake depth is coordinated with leaf water potential, water-use efficiency and drought vulnerability in karst vegetation. New Phytol 229: 1339–1353

Docherty EM, Gloor E, Sponchiado D, Gilpin M, Pinto CAD, Junior HM, Coughlin I, Ferreira L, Junior JAS, da Costa ACL, Meir P, Galbraith D (2023) Long-term drought effects on the thermal sensitivity of Amazon forest trees. Plant Cell Environ 46: 185–198

Eamus D, Myers B, Duff G, Williams D (1999) Seasonal changes in photosynthesis of eight savanna tree species. Tree Physiol 19: 665–671

Esteban EJL, Castilho CV, Melgaço KL, Costa FRC (2021) The other side of droughts: wet extremes and topography as buffers of negative drought effects in an Amazonian forest. New Phytol 229: 1995–2006

Eze CE, Winter K, Slot M (2024) Vapor-pressure-deficit-controlled temperature response of photosynthesis in tropical trees. Photosynthetica 62: 318–325

Garen JC, Branch HA, Borrego I, Blonder B, Stinziano JR, Michaletz ST (2022) Gas exchange analysers exhibit large measurement error driven by internal thermal gradients. New Phytol 236: 369–384

Gjindali A, Herrmann HA, Schwartz J-M, Johnson GN, Calzadilla PI (2021) A holistic approach to study photosynthetic acclimation responses of plants to fluctuating light. Front Plant Sci 12: 668512

Gjindali A, Johnson GN (2023) Photosynthetic acclimation to changing environments. Biochem Soc Trans 51: 473–486

Gloor E, Barua D, Galbraith DR, Hegde B, Sunny R, Tiwari R (2023) Leaf water potential of tropical forest tree species, Sirsi, Western Ghats, India, 2020-2021. doi.org/10.5285/252b6a14-8a0e-4a6f-a879-99dff46fec71

Grace J, Okali DUU, Fasehun FE (1982) Stomatal conductance of two tropical trees during the wet season in Nigeria. J Appl Ecol 19: 659

Green JK, Berry J, Ciais P, Zhang Y, Gentine P (2020) Amazon rainforest photosynthesis increases in response to atmospheric dryness. Sci Adv 6:

Guha S, Jain V (2020) Role of inherent geological and climatic characteristics on landscape variability in the tectonically

passive Western Ghat, India. Geomorphology (Amst) 350: 106840

Harris NL, Medina E (2013) Changes in leaf properties across an elevation gradient in the Luquillo Mountains, Puerto Rico. Ecol Bull 169–180

Hasselquist NJ, Allen MF, Santiago LS (2010) Water relations of evergreen and drought-deciduous trees along a seasonally dry tropical forest chronosequence. Oecologia 164: 881–890

Hernández GG, Winter K, Slot M (2020) Similar temperature dependence of photosynthetic parameters in sun and shade leaves of three tropical tree species. Tree Physiol 40: 637–651

Hikosaka K (2005) Nitrogen partitioning in the photosynthetic apparatus of Plantago asiatica leaves grown under different temperature and light conditions: similarities and differences between temperature and light acclimation. Plant Cell Physiol 46: 1283–1290

Ishida A, Diloksumpun S, Ladpala P, Staporn D, Panuthai S, Gamo M, Yazaki K, Ishizuka M, Puangchit L (2006) Contrasting seasonal leaf habits of canopy trees between tropical dry-deciduous and evergreen forests in Thailand. Tree Physiol 26: 643–656

Ishida A, Yamazaki J-Y, Harayama H, Yazaki K, Ladpala P, Nakano T, Adachi M, Yoshimura K, Panuthai S, Staporn D, Maeda T, Maruta E, Diloksumpun S, Puangchit L (2014) Photoprotection of evergreen and drought-deciduous tree leaves to overcome the dry season in monsoonal tropical dry forests in Thailand. Tree Physiol 34: 15–28

Janssen T, Fleischer K, Luyssaert S, Naudts K, Dolman H (2020) Drought resistance increases from the individual to the ecosystem level in highly diverse Neotropical rainforest: a meta-analysis of leaf, tree and ecosystem responses to drought. Biogeosciences 17: 2621–2645

Joshi RK, Mishra A, Gupta R, Garkoti SC (2024) Leaf and tree age-related changes in leaf ecophysiological traits, nutrient, and adaptive strategies of Alnus nepalensis in the central Himalaya. J Biosci 49: 1–14

June T, Evans JR, Farquhar GD (2004) A simple new equation for the reversible temperature dependence of photosynthetic electron transport: A study on soybean leaf. Funct Plant Biol 31:

Kailash BR, Charles B, Ravikanth G, Setty S, Kadirvelu K (2022) Identifying the potential global distribution and conservation areas for Terminalia chebula, an important medicinal tree species under changing climate scenario. Trop Ecol 63: 584–595

Kattge J, Knorr W (2007) Temperature acclimation in a biochemical model of photosynthesis: a reanalysis of data from 36 species. Plant Cell Environ 30: 1176–1190

Kitajima K, Mulkey SS, Wright SJ (1997) Seasonal leaf phenotypes in the canopy of a tropical dry forest: photosynthetic characteristics and associated traits. Oecologia 109: 490–498

Köpp Hollunder R, Garbin ML, Rubio Scarano F, Mariotte P (2022) Regional and local determinants of drought resilience in tropical forests. Ecol Evol 12: e8943

Kositsup B, Montpied P, Kasemsap P, Thaler P, Dreyer E (2008) Photosynthetic capacity and temperature responses of photosynthesis of rubber trees (Hevea brasiliensis Müll. Arg.) acclimate to changes in ambient temperatures. Trees 23:

Krishnadas M, Kumar A, Comita LS (2016) Environmental gradients structure tropical tree assemblages at the regional scale. J Veg Sci 27: 1117–1128

Krishnadas M, Sankaran M, Page N, Joshi J, Machado S, Nataraj N, Chengappa SK, Kumar V, Kumar A, Krishnamani R (2021) Seasonal drought regulates species distributions and assembly of tree communities across a tropical wet forest region. Glob Ecol Biogeogr 30: 1847–1862

Kühnhammer K, van Haren J, Kübert A, Bailey K, Dubbert M, Hu J, Ladd SN, Meredith LK, Werner C, Beyer M (2023) Deep roots mitigate drought impacts on tropical trees despite limited quantitative contribution to transpiration. Sci Total Environ 893: 164763

Liu J, Ryu Y, Luo X, Dechant B, Stocker B, Keenan T, Gentine P, Li X, Li B, Harrison S, Prentice I (2024) Evidence for widespread thermal acclimation of canopy photosynthesis. Research Square doi.org/10.21203/rs.3.rs-4013319/v1

Montagu KD, Woo KC (1999) Recovery of tree photosynthetic capacity from seasonal drought in the wet - dry tropics: the role of phyllode and canopy processes in Acacia auriculiformis. Funct Plant Biol 26: 135

Mott KA, Peak D (2011) Alternative perspective on the control of transpiration by radiation. Proceedings of the National Academy of Sciences 108: 19820–19823

Mujawamariya M, Wittemann M, Dusenge ME, Manishimwe A, Ntirugulirwa B, Zibera E, Nsabimana D, Wallin G, Uddling J (2023) Contrasting warming responses of photosynthesis in early- and late-successional tropical trees. Tree Physiol 43: 1104–1117

Muller O, Hirose T, Werger MJA, Hikosaka K (2011) Optimal use of leaf nitrogen explains seasonal changes in leaf nitrogen content of an understorey evergreen shrub. Ann Bot 108: 529–536

Naidu C, Swamy PM (1995) Seasonal pattern of photosynthetic rate and its relationship with chlorophyll content, ribulose-1,5-bisphosphate carboxylase activity and biomass production. Biol Plant 37: 349–354

Nie Y-P, Chen H-S, Wang K-L, Tan W, Deng P-Y, Yang J (2011) Seasonal water use patterns of woody species growing on the continuous dolostone outcrops and nearby thin soils in subtropical China. Plant Soil 341: 399–412

Pascal JP (1988) Wet evergreen forests of the Western Ghats of India. Institut francais de Pondichery, Pondichery

Peng J, Feng Y, Wang X, Li J, Xu G, Phonenasay S, Luo Q, Han Z, Lu W (2021) Effects of nitrogen application rate on the photosynthetic pigment, leaf fluorescence characteristics, and yield of indica hybrid rice and their interrelations. Sci Rep 11: 7485

Pinheiro J, Bates D, DebRoy S, And DS, R Core Team, (2018) nlme: Linear and nonlinear mixed effects models. In: R package

R Core Team, (2025) R: A language and environment for statistical computing

Rey-Sánchez AC, Slot M, Posada JM, Kitajima K (2016) Spatial and seasonal variation in leaf temperature within the canopy of a tropical forest. Clim Res 71: 75–89

Ribeiro RV, Machado EC, Santos MG, Oliveira RF (2009) Seasonal and diurnal changes in photosynthetic limitation of young sweet orange trees. Environ Exp Bot 66: 203–211

Santos VAHFD, Ferreira MJ, Rodrigues JVFC, Garcia MN, Ceron JVB, Nelson BW, Saleska SR (2018) Causes of reduced leaf-level photosynthesis during strong El Niño drought in a Central Amazon forest. Glob Chang Biol 24: 4266–4279

Sarkar S, Maity R (2022) Future Characteristics of Extreme Precipitation Indicate the Dominance of Frequency Over Intensity: A Multi-Model Assessment From CMIP6 Across India. J Geophys Res D: Atmos 127:

Schmitt S, Trueba S, Coste S, Ducouret É, Tysklind N, Heuertz M, Bonal D, Burban B, Hérault B, Derroire G (2022) Seasonal

variation of leaf thickness: An overlooked component of functional trait variability. Plant Biol 24: 458–463

Schwartz NB, Medvigy D, Tijerin J, Pérez-Aviles D, Rivera-Polanco D, Pereira D, Vargas G. G, Werden L, Du D, Arnold L, Powers JS (2022) Intra-annual variation in microclimatic conditions in relation to vegetation type and structure in two tropical dry forests undergoing secondary succession. For Ecol Manage 511: 120132

Sendall KM, Vourlitis GL, Lobo FA (2009) Seasonal variation in the maximum rate of leaf gas exchange of canopy and understory tree species in an Amazonian semi-deciduous forest. Braz J Plant Physiol 21: 65–74

Shi C, Sun G, Zhang H, Xiao B, Ze B, Zhang N, Wu N (2014) Effects of Warming on Chlorophyll Degradation and Carbohydrate Accumulation of Alpine Herbaceous Species during Plant Senescence on the Tibetan Plateau. PLoS One 9: e107874

Shigwan BK, Kulkarni A, Smrithy V, Datar MN (2024) An overview of tree ecology and forest studies in the Northern Western Ghats of India. IForest 17: 213–221

Slot M, Winter K (2017a) Photosynthetic acclimation to warming in tropical forest tree seedlings. J Exp Bot 68: 2275–2284

Slot M, Winter K (2017b) In situ temperature relationships of biochemical and stomatal controls of photosynthesis in four lowland tropical tree species. Plant Cell Environ 40: 3055–3068

Stahl C, Hérault B, Rossi V, Burban B, Bréchet C, Bonal D (2013) Depth of soil water uptake by tropical rainforest trees during dry periods: does tree dimension matter? Oecologia 173: 1191–1201

Still CJ, Sibley A, Page G, Meinzer FC, Sevanto S (2019) When a cuvette is not a canopy: A caution about measuring leaf temperature during gas exchange measurements. Agric For Meteorol 279: 107737

Tange T (1996) Seasonal changes in photosynthesis of young Cryptomeria japonica growing on ridges and foot-slopes. For Ecol Manage 89: 93–99

Urban J, Ingwers MW, McGuire MA, Teskey RO (2017) Increase in leaf temperature opens stomata and decouples net photosynthesis from stomatal conductance in *Pinus taeda* and *Populus deltoides x nigra*. J Exp Bot 68: 1757–1767

Uribe MR, Sierra CA, Dukes JS (2021) Seasonality of tropical photosynthesis: A pantropical map of correlations with precipitation and radiation and comparison to model outputs. J Geophys Res Biogeosci 126:

Vourlitis GL, de Souza Nogueira J, de Almeida Lobo F, Sendall KM, de Paulo SR, Dias CAA, Pinto OB, de Andrade NLR (2008) Energy balance and canopy conductance of a tropical semi-deciduous forest of the southern Amazon Basin. Water Resources Research 44:

Wada N, Kondo I, Tanaka R, Kishimoto J, Miyagi A, Kawai-Yamada M, Mizokami Y, Noguchi K (2023) Dynamic seasonal changes in photosynthesis systems in leaves of Asarum tamaense, an evergreen understorey herbaceous species. Ann Bot 131: 423–436

Way DA, Yamori W (2014) Thermal acclimation of photosynthesis: on the importance of adjusting our definitions and accounting for thermal acclimation of respiration. Photosynth Res 119: 89–100

Wild J, Kopecký M, Macek M, Šanda M, Jankovec J, Haase T (2019) Climate at ecologically relevant scales: A new temperature and soil moisture logger for long-term microclimate measurement. Agric For Meteorol 268: 40–47

Wittemann M, Andersson MX, Ntirugulirwa B, Tarvainen L, Wallin G, Uddling J (2022) Temperature acclimation of net photosynthesis and its underlying component processes in four tropical tree species. Tree Physiol 42: 1188–1202

Wu J, Albert LP, Lopes AP, Restrepo-Coupe N, Hayek M, Wiedemann KT, Guan K, Stark SC, Christoffersen B, Prohaska N, Tavares JV, Marostica S, Kobayashi H, Ferreira ML, Campos KS, da Silva R, Brando PM, Dye DG, Huxman TE, Huete AR, Nelson BW, Saleska SR (2016) Leaf development and demography explain photosynthetic seasonality in Amazon evergreen forests. Science 351: 972–976

Yamaguchi DP, Nakaji T, Hiura T, Hikosaka K (2016) Effects of seasonal change and experimental warming on the temperature dependence of photosynthesis in the canopy leaves of Quercus serrata. Tree Physiol 36: 1283–1295

Yamasaki T, Yamakawa T, Yamane Y, Koike H, Satoh K, Katoh S (2002) Temperature Acclimation of Photosynthesis and Related Changes in Photosystem II Electron Transport in Winter Wheat. Plant Physiol 128: 1087–1097

Yamori W, Hikosaka K, Way DA (2014) Temperature response of photosynthesis in C3, C4, and CAM plants: temperature acclimation and temperature adaptation. Photosynth Res 119: 101–117

Yamori W, Noguchi K, Hanba YT, Terashima I (2006) Effects of internal conductance on the temperature dependence of the photosynthetic rate in spinach leaves from contrasting growth temperatures. Plant Cell Physiol 47: 1069–1080

Yasumura Y, Hikosaka K, Hirose T (2006) Seasonal changes in photosynthesis, nitrogen content and nitrogen partitioning in Lindera umbellata leaves grown in high or low irradiance. Tree Physiol 26: 1315–1323

Zhang C, Su Y, Liu L, Wu J, Huang G, Li X, Bi C, Yan W, Lafortezza R (2023) Seasonal and long-term dynamics in forest microclimate effects: global pattern and mechanism. Npj Clim Atmos Sci 6: 1–12

Zhang J-L, Zhu J-J, Cao K-F (2007) Seasonal variation in photosynthesis in six woody species with different leaf phenology in a valley savanna in southwestern China. Trees 21: 631–643

Glossary of English and Kannada terms used

Air – ವಾಯು, assimilation rate – ಉಪಗ್ರಹಣ ದರ, brevideciduous – ಅರೆ-ಪರ್ಣಪಾತಿ / ಅರೆ-ಪತನಶೀಲ, buffer – ರಕ್ಷಣೆ, bulk density – ಸ್ಥೂಲ ಸಾಂದ್ರತೆ, canopy (tree) – ಮೇಲಾವರಣ, carbon dioxide – ಇಂಗಾಲದ ಡೈಆಕ್ಸೈಡ್, climate – ದೀರ್ಘಕಾಲಿಕ ವಾತಾವರಣ, co-occurring – ಸಹ-ಸಂಭವಿಸುವ / ಸಹವರ್ತಿ, coexisting – ಸಹವಾಸಿ, data – ದತ್ತಾಂಶ, deciduous – ಪರ್ಣಪಾತಿ, deciduous leaf habit – ಪರ್ಣಪಾತಿ ಪರ್ಣ ಸ್ವಭಾವ, dry affinity – ಶುಷ್ಕ-ಅವಲಂಬಿತ, evergreen leaf habit – ನಿತ್ಯಹರಿದ್ವರ್ಣ ಪರ್ಣ ಸ್ವಭಾವ, mathametical model fit – ಗಣಿತೀಯ ಫಲನ, fluorescence – ಪ್ರತಿದೀಪಕ, in situ – ಮೂಲ ಸ್ಥಾನಿಕ, insulated thermo couple – ವಿದ್ಯುತ್ ನಿರೋಧಿತ ತಾಪಸಂಧಿ, irreversible – ಅನಿವರ್ತಿತ, juveniles – ವೃಕ್ಷ ತರು, landscape – ಭೂದೃಶ್ಯ, leaf chamber – ಪತ್ರಕೋಶ, leaf habit – ಪರ್ಣ ಸ್ವಭಾವ, leaf phenology – ಎಲೆ ಋತುಧರ್ಮ/ ಪತ್ರ ಋತು ವಿಕಾಸ (ಶಾಸ್ತ್ರ), light saturated – ಪ್ರಕಾಶ ಪೂರಿತ, logger – ಅಭಿಲೇಖಯಂತ್ರ, measure/measurement – ಮಾಪನ, microclimate – ಸೂಕ್ಷ್ಮ ವಾತಾವರಣ, niche preference – ತಾಪ ಗುಣ ವಿಶಿಷ್ಟ ಕ್ಷೇತ್ರ ಆದ್ಯತೆ, optimal – ಆದರ್ಶ, optimisation – ಉತ್ತಮೀಕರಣ, parameter – ನಿಯತಾಂಕ, petri plate – ಪೆಟ್ರೀ-ತಟ್ಟೆ, phenology – ಋತು-ಧರ್ಮ, photoperiod – ದ್ಯುತಿ ಅವಧಿ, photoprotection – ದ್ಯುತಿ ರಕ್ಷಣೆ, photosynthesis – ದ್ಯುತಿಸಂಶ್ಲೇಷಣೆ, photosynthetic CO_2 assimilation – ಇಂಗಾಲದ ಡೈಆಕ್ಸೈಡ್ ದ್ಯುತಿಸಂಶ್ಲೇಶಕ ಸಂಯೋಜನೆ, photosystem – ದ್ಯುತಿ ಸಂಹಿತೆ, physiology – ಕ್ರಿಯಾಪ್ರಕ್ರಿಯೆ, plastic – ಸಮಾಯೋಜ್ಯ, ನಮ್ಯತೆ / ಪರಿವರ್ತನೀಯತೆ, plot – ಆಲೇಖಾ ಚಿತ್ರ, proxy – ಪ್ರತಿನಿಧಿಯಾಗಿ, quadratic (second order) – ದ್ವಿಘಾತ, reversible – ನಿವರ್ತಿತ, season – ಋತು / ಋತುಮಾನ, seasonal – ಋತುಅವಲಂಬಿತ, seasonal difference – ಋತುಭೇದ, seedling – ಸಸಿ, species – ಪ್ರಭೇದ, ವೃಕ್ಷ ಪ್ರಭೇದ, species specific – ಪ್ರಭೇದ ನಿರ್ದಿಷ್ಟ, stomata – ಪತ್ರ ರಂಧ್ರ, stomatal conductance – ಪತ್ರರಂಧ್ರ ವಾಹಕತೆ, subcanopy (tree) – ಉಪ-ಮೇಲಾವರಣ, thermal optima – ಆದರ್ಶ ತಾಪಮಾನ, thermal tolerance – ಉಷ್ಣ ಸಹಿಷ್ಣುತೆ, topography – ಸ್ಥಳಾಕೃತಿ, traits – ಲಕ್ಷಣಗಳು, transpiration – ಭಾಷ್ಪವಿಸರ್ಜನೆ, tree species – ವೃಕ್ಷ ಪ್ರಭೇದ, understorey tree – ಅಧೋವಿತಾನ ವೃಕ್ಷ / ಕೆಳ ಆವರಣ ವೃಕ್ಷ, undulating – ಏರಿಳಿತ, unit – ಮಾನ, warmer thermal niche preference – ಉಷ್ಣತರ ತಾಪ ಗುಣ ವಿಶಿಷ್ಟ ಕ್ಷೇತ್ರ ಆದ್ಯತೆ, water availability – ಜಲ ಲಭ್ಯತೆ, weather – ಪ್ರಸ್ತುತ ವಾತಾವರಣ, wet – ಆರ್ದ್ರ, wind – ಗಾಳಿ, y-axis – ಲಂಬಾಕ್ಷ
