# Supplementary material for: Contrasting Seasonal Variation of Photosynthesis in Evergreen and Deciduous Tree Species From a Tropical Forest
Source: Physiol Plant. 2025 Jul 14;177(4):e70410. doi: 10.1111/ppl.70410 (PMC12257110; doi:10.1111/ppl.70410)
Supplement: Supplementary file 1 — Data S1: Supporting Information. [file PPL-177-e70410-s001.zip › 3_Supplementary-Section-03-Sanskrit-Language version.pdf]

The Sanskrit language version of the original peer reviewed manuscript Tiwari et al., (2025) in Physiologia Plantarum. This section has not been reviewed and is only provided as supplementary material. Please refer to the English version of the manuscript for the peer-reviewed version (DOI 10.1111/ppl.70410). Sanskrit language version created by Rakesh Tiwari with support from Shankaranarayana Bhatta HS, Vasudeva HR, and Chethan TR. Refer to the technical terms glossary at the end of this document for original English terms and corresponding Sanskrit terms used.

उष्णवलयवने नित्यहरितपर्णपातिवृक्षप्रजातिषु प्रकाशसंश्लेषणस्य ऋतुकालिकाः विरुद्धभेदाः दृश्यन्ते

Rakesh Tiwari[1, 2, 3]* | Balachandra Hegde[3,4] | Shrihari Hegde[5,3] | Peddiraju Bandaru[6] | Ramesh Babu M[7] | Somashekhara Achar KG[8,9] | Caroline Greiser[10] | Robert Muscarella[2] | Deepak Barua[6] | David Galbraith[1] | Emanuel Gloor[1]

[1] School of Geography, University of Leeds, United Kingdom
[2] Plant Ecology and Evolution, Institute of Ecology and Genetics, Uppsala Universitet, Sweden
[3] Sahyadri Ecological Observatory, Sirsi, India
[4] Department of Environmental Science, Kuvempu University, India
[5] Mangalore University, India
[6] Indian Institute of Science Education and Research, Pune, India
[7] Department of Electronic Media, Bangalore University, Bengaluru, India
[8] IDSG Government College, Chikkamagaluru, India
[9] Panchavati Research Academy for Nature, India
[10] Department of Physical Geography, Stockholm University, Sweden

* Corresponding author

संक्षेपः

जलस्य उपलब्धतायां सूक्ष्मवातावरणविविधतायाः कारणेन सह-उपस्थितेषु उष्णवलय वृक्षप्रजातिषु जलप्रयोगे ऋतुविविधता तथा प्रकाशसंश्लेषणस्य दरः भवितुं शक्नोति, विशेषतः प्रबलऋतुवातावरणयुक्तेषु वनेषु । वयं भारतस्य सह्याद्री पर्वतश्रेणीषु एकस्य उष्णवलय वनस्थलस्य अध्ययनं कृतवन्तः तथा च विपरीतपर्णस्वभावैः स्थलाकृतिकसम्बन्धैः च नव वृक्षजातीषु प्रकाशसंश्लेषणीय-CO_2-उपग्रहणगतिषु (A_{net}) ऋतुविविधतायाः लक्षणं कृतवन्तः - शुष्कपर्वतशिखरेषु पतनशील प्रजातयः, सानुषु शुष्कप्रेमी नित्यहरिद्वर्ण पत्रस्वभाव वृक्षाः, उपत्यकेषु च आर्द्रप्रेमी नित्यहरिद्वर्ण पत्रस्वभाव वृक्षाः । भूपृष्ठीय मृदार्द्रता पर्वत शिखरेषु न्यूना, सानुषु मध्यमा, उपत्यकेषु च अधिका आसीत् शुष्कऋतुस्य तुलने आर्द्रऋतौ अधिकस्तरः अवलोकितः । यथा अपेक्षितं, शुष्कपर्वत शिखरेषु पर्णपातीजातयः आर्द्रऋतौ आदर्शतापमानेषु (T_{opt}) अधिकं प्रकाशसंश्लेषणदरं दर्शयन्ति स्म, यदा तु नित्यहरिद्वर्ण जातयः समग्ररूपेण ऋतुभेदं न दर्शयन्ति स्म रोचकं तत् अस्ति यत् शुष्कसानुषु नित्यहरिद्वर्ण जातयः आर्द्रऋतुस्य तुलने शुष्कऋतौ आदर्शतापमानेषु अधिकं A_{net} दर्शितवन्तः । येन एतेषु प्रजातीषु विशिष्टक्षेत्रप्राथमिकता वा तापपरिवर्तनशीलता वा सूचितं भवति न्यून मृदार्द्रतायाः । शुष्कऋतौ पर्याप्तजलस्य उपलब्धतायाः, तापपर्यावरणस्य च विशिष्टक्षेत्रीयप्राथमिकता वा तापअनुकूलनस्य च अभावेऽपि T_{opt}-स्थले एकं नित्यहरिद्वर्ण जातिं विहाय सर्वेषु जातिषु वर्षाऋतौ सामान्यतया पत्रछिद्रवाहकता (g_s) अधिका आसीत् । अस्माकं निष्कर्षाः भिन्नपत्रलक्षणैः स्थलाकृतिसम्बन्धैः च उष्णवलयवृक्षजातीनां प्रकाशसंश्लेषणस्य भेदं दर्शयन्ति ।

मुख्यशब्दाः – प्रकाशसंश्लेषणम्, उष्णवलयवनं, वृक्षाः, ऋतुपरिवर्तनशीलता , पत्रछिद्रवाहकता , ऋतुत्वं, स्थलाकृतिः, मृदार्द्रता, सह्याद्री पर्वतश्रेणी

१ प्रस्ताव

वैश्विकरूपेण उष्णवलयवनानि विविधऋतुप्रतिमानं प्रदर्शयन्ति, अचक्रीयतः प्रबलतया ऋतुकालपर्यन्तं विशिष्टार्द्रशुष्ककालैः सह (Carvalho et al. 2021) । शुष्क उष्णवलय वनेषु जलस्य उपलब्धतायां वायुतापमानस्य च विशिष्टऋतुविविधता भवति । इयं ऋतुत्वं वृक्षजातीनां कृयाप्रकृयतां प्रभावितं करोति, यत् क्रमेण पत्रस्वभावेषु जलसम्बन्धेषु च भेदेषु प्रकट्यते (Rey-Sánchez et al. 2016) । अपि च, सूक्ष्मवातावरणकारकाः, यथा स्थलाकृतिः, वृक्षजातीनां मध्ये पत्रऋतुत्वस्य स्थानिकवितरणं च (Zhang et al. 2023), वनस्पतिषु ऋतुविविधतां संयोजयितुं शक्नुवन्ति (Schwartz et al. 2022) । एतेषु ऋतुअवलम्बित शुष्कउष्णवलय वनेषु वृक्षजातीनां मध्ये प्रक्रियासु ऋतुविविधतायाः उत्तमसमझः अनावृष्टेः भविष्यस्य तापनस्य च प्रति वृक्षजातीनां संवेदनशीलतां अवगन्तुं साहाय्यं करिष्यति (Köpp Hollunder et al. 2022) । यथा, प्रकाशसंश्लेषणस्य ऋतुविविधतायाः विस्तारः – प्राथमिककार्बन्

उपग्रहणप्रक्रिया – वृक्षेषु कथं भिद्यते, विशेषतः ऋतुविविधतायाः दृष्ट्या, जलस्य उपलब्धतायां सूक्ष्मवातावरणप्रेरितविविधता कथं भिन्ना भवितुम् अर्हति इति अस्पष्टम् अस्ति ।

प्रकाशसंश्लेषणस्य ऋतुविविधता पर्यावरणीयकारकाणां मध्ये जटिल अन्तरकृयाभिः चालिता भवति, यत्र वायुः, तापमानं, जलस्य उपलब्धता, प्रकाशस्तरः, दिवसस्य दीर्घता वा प्रकाशकालः च सन्ति (Yamaguchi et al. 2016) । जैवरासायनिकतन्त्राणि अपि भूमिकां निर्वहन्ति, यत्र उत्प्रेरकाः तथा प्रकाशसंश्लेषणीय ऋणाणु परिवहनस्य समायोजनं (Wada et al. 2023) तथा माइटोकॉन्ड्रिया श्वसन (Way and Yamori 2014) च सन्ति । एते कारकाः प्रायः पत्रेषु सारजनक सामग्रीषु, वर्णकद्रव्येषु, पत्रस्य ऋतुत्वे च प्रतिबिम्बिताः भवन्ति (Yasumura et al. 2006; Muller et al. 2011) । उदाहरणार्थं, पत्रेषु सारजनकस्य मात्रा सामान्यतया प्रारम्भिकवृद्धिपदेषु सर्वाधिकं भवति तथा च वयसः सह न्यूनीभवति (Joshi et al. 2024) । तथैव पत्ररञ्जकसामग्री, विशेषतः क्लोरोफिल्, पत्र सारजनकेन सह अधिकतमं भवति, यदा तु प्रकाशसंरक्षणे महत्त्वपूर्णां भूमिकां निर्वहन्ति कैरोटीनॉयड्-धातुः ऋतुविविधतां अल्पं दर्शयति (Shi et al. 2014; Peng et al. 2021; Wada et al. 2023) । यथा यथा पत्राणि स्वस्य शिखरवृद्धिकालात् परं वृद्धाः भवन्ति तथा तथा पत्ररञ्जकस्य सारजनक सामग्रीयाश्च न्यूनतायाः कारणेन प्रकाशसंश्लेषणक्षमता न्यूनीभवति (Yasumura et al. 2006) । सहचरवृक्षजातीषु, विशेषतः उच्चजैवविविधतायुक्तेषु वनेषु, विविधऋतुत्वप्रकाशसंश्लेषणे विविधऋतुप्रतिमानं जनयितुं शक्नोति (Corredor-Londoño et al. 2020; Devi et al. 2023; Wada et al. 2023) ।

प्रकाशसंश्लेषण मापदण्डेषु ऋतुविविधता अपि ऋतुकालीन पर्यावरणपरिवर्तनस्य अभ्यस्तीकरण प्रतिक्रियाः सूचयति (Wittemann et al. 2022)। अभ्यस्तीकरणे शारीरिकं, संरचनात्मकं, जैवरासायनिकं वा परिवर्तनशीलता भवति यस्य परिणामेण प्रकाशसंश्लेषणस्य आदर्शतापमानस्य (T_{opt}), आदर्शतापमानस्य प्रकाशसंश्लेषणस्य दरस्य (A_{opt}), अधिकतमस्य RuBP कार्बोक्सिलेशन् दरस्य च परिवर्तनं भवति तापीय आदर्शतापमानस्य परिवर्तनं रुबिस्को कार्बोक्सिलेशनस्य (Hikosaka 2005; Borjigidai et al. 2006) तथा विभिन्नप्रकाशसंश्लेषणमार्गेषु अन्यतापमाननिर्भरप्रक्रियासु (Yamori et al. 2014) सक्रियकरणशक्तिपरिवर्तनेन सह सम्बद्धा अस्ति । सामान्यतया, उष्णवलयवृक्षजातयः समशीतोष्ण-अथवा बोरियल-जातीयानां अपेक्षया न्यूनतया मध्यमतापमानं प्रति स्वस्य प्रकाशसंश्लेषणं अनुकूलयन्ति (Slot and Winter 2017a; Wittemann et al. 2022; Liu et al. 2024) । अभ्यस्तीकरणस्य विस्तारः जातिषु भिन्नः भवति; उदाहरणार्थं, न्यून-उच्चतायुक्ताः प्रजातयः पर्वतीयजातीयानां अपेक्षया तापमानवृद्धेः अधिकं प्रबलतया अनुकूलतां प्राप्नुवन्ति (Wittemann et al. 2022) । सामान्यतया प्रकाशसंश्लेषणस्य आदर्शतापमानं अधिकतमस्थान तापमानेन सह वर्धते (Yamasaki et al. 2002; Hikosaka 2005; Choury et al. 2022) । T_{opt} इत्यस्य ऋतु परिवर्तनशीलता वर्षे पूर्णे प्रकाशसंश्लेषण दक्षतां अधिकतमं कर्तुं वा निर्वाहयितुं प्रजातीनां कार्य नीतिं निर्दिशति (Kattge and Knorr 2007), यद्यपि अस्य विषये अधिकांशं ज्ञानं सस्यानां अथवा किशोर व्रुक्षाणाम् अध्ययनात् प्राप्तम् अस्ति (Gjindali et al. 2021; Gjindali and Johnson 2023) । अतः ऋतुकालीन पर्यावरण विविधता युक्तेषु उष्णवलय वनेषु प्रौढवृक्षाणां विषये संशोधने महत्त्वपूर्णः अभावः अस्ति ।

ऋतु-अन्तरस्य विशेषतां दर्शयन्तः पत्र-स्तरीय-अध्ययनं प्रायः शुष्क-ऋतौ प्रकाश-संश्लेषण-दरेषु न्यूनतां ज्ञापयति, विशेषतः पतनशील-प्रजातीषु (Eamus et al. 1999; Zhang et al. 2007) । पत्रस्य ऋतुत्वं जलस्य उपलब्धता च प्रायः एतेषां ऋतुभेदानाम् व्याख्यानार्थं प्रमुखकारकाणां रूपेण चिह्निता भवति (Eamus et al. 1999; Zhang et al. 2007) । उदाहरणार्थं पनामादेशे अध्ययनेन किशोर वृक्षेषु शुष्कऋतौ प्रकाशसंश्लेषणस्य दरं न्यूनीकृतं ज्ञातम् (Craven et al. 2011) । ऑस्ट्रेलियादेशस्य *Acacia* जातीनां किशोर वृक्षेषु अपि तथैव शुष्कऋतुक्षयः मापितः अस्ति (Montagu and Woo 1999) । उष्णवलय थाईलैण्डदेशे कृते प्रौढवृक्षाणां पत्रस्तरीयमूलस्थानमापनेन एकस्य नित्यहरिद्वर्ण जातेः कृते वर्षे पूर्णे स्थिरप्रकाशसंश्लेषणदराः दर्शिताः, अन्ययोः नित्यहरिद्वर्ण जातीययोः शुष्कऋतौ क्षयः प्रदर्शितः (Ishida et al. 2006) । तथैव अमेजन-वने कृते अध्ययने शुष्कऋतौ वितानवृक्षेषु प्रकाशसंश्लेषणस्य दरस्य न्यूनता ज्ञाता, यदा तु अण्डरस्टोरी-जातीनां प्रकाशसंश्लेषणस्य दरं तस्मिन् एव काले किञ्चित् न्यूनीकृतम् (Santos et al. 2018) एवं प्रकाशसंश्लेषणस्य दरस्य शुष्कऋतुस्य न्यूनता सामान्यतया ज्ञायते । तस्य विपरीतम्, केषुचित् अध्ययनेषु ग्रीष्मर्तौ (Naidu and Swamy 1995) तथा भारते अर्धपर्णपाती, पर्णपाती, नित्यहरिद्वर्ण जातीनां किशोर वृक्षेषु (Abhilash and Devakumar 2023) । प्रकाशसंश्लेषणस्य दरं अधिकं ज्ञातम् अस्ति एताः विविधाः प्रतिक्रियाः सूचयन्ति यत् अन्ये स्थानिककारकाः, यथा सूक्ष्मवातावरणः, प्रकाशसंश्लेषणस्य ऋतुविविधतां प्रभावितुं शक्नुवन्ति - विशेषतः ऋतुअवलम्बित शुष्कउष्णवलय वनेषु ।

प्रकाशसंश्लेषणदरेषु ऋतुविविधतायाः अतिरिक्तं वायुविनिमयस्य अन्ये पक्षाः, यथा पत्रछिद्रवाहकता (g_s) ऋतुविविधतां प्रदर्शयितुं शक्नुवन्ति । सामान्यवायुमण्डलीयस्थितौ रसस्य चालकता प्रकाशसंश्लेषणदरेण सह निकटतया सम्बद्धा भवति (Slot and Winter 2017b) । परन्तु अधिकतापमानयोः g_s प्रतिक्रियाः प्रायः प्रकाशसंश्लेषणात् विचलिताः भवन्ति (Asargew et al. 2024) । पत्रछिद्रवाहकतायाः तापप्रतिक्रियायाः आकारः भिन्नः भवति । यद्यपि g_s तापमानेन सह रेखीयरूपेण न्यूनतां दर्शयति (Urban et al. 2017; Eze et al. 2024), तथापि केचन प्रतिवेदनाः g_s इत्यस्य वृद्धिं सूचयन्ति

तथा च, केषुचित् प्रकरणेषु, अधिकतापमानेषु शिखरप्रतिक्रियाम् (Ymori et al. 2006; Hernández et al. 2020) । सामान्यतया आर्द्रऋतौ पर्णपातवृक्षेषु g_s अधिकं भवति (Grace et al. 1982), परन्तु केचन अध्ययनाः दर्शयन्ति यत् नित्यहरिद्वर्ण जातीनां g_s महत्त्वपूर्णरूपेण परिवर्तनं न करोति (Andriyas et al. 2021) । परन्तु ऋतुअवलम्बितेषु उष्णवलय वनेषु विशेषतः प्रौढ वृक्षेषु - विशेषतः शुष्कपरिस्थितौ पत्रछिद्रवाहकता नीतिषु सम्भाव्यतया भिन्नता भवितुम् अर्हति । इदं ऋतुविविधता शुष्क-आर्द्र-ऋतुषु वायु-तापमानस्य, वाष्पबलम्स्य, जलस्य च उपलब्धतायां भेदस्य कारणेन भवितुम् अर्हति (Comita and Engelbrecht 2009; Schwartz et al. 2022), मूलस्यगहनतायां जलस्य उपलब्धतायां च भेदः (Stahl et al. 2013), तथा च मृदाजलस्य उपलब्धतायां (Vourlitis et al. 2008; Schmitt et al. 2022) । एवं यद्यपि विद्यमान संशोधना साहित्येषु प्रकाशसंश्लेषणस्य ऋतु विविधतायाः केचन अन्वेषणाः प्राप्यन्ते, तथापि ऋतुशुष्केषु उष्णवलय वनेषु प्रौढवृक्षेषु प्रकाशसंश्लेषणस्य तापमानप्रतिक्रियायां ऋतुविविधतायाः सीमितबोधः दृश्यते ।

एतस्य अभावस्य निवारणाय वयं भारतस्य सह्याद्री पर्वतश्रेणीवने, मध्य भागे नव वृक्षजातीनां अध्ययनं कृतवन्तः, यत् वैश्विक जैवविविधतायाः प्रधानक्षेत्रं अस्ति, यस्य विशेषता अस्ति यत् विशिष्टः शुष्कः, उष्णः ग्रीष्मकालः तदनन्तरं चतुर्मासिकवृष्टिऋतुः भवति । अस्मिन् प्रदेशे प्रकाशसंश्लेषणस्य तापमान संवेदनशीलतायाः ऋतुत्वस्य वा अध्ययनं न कृतम् । स्थलाकृतिः, जलस्य उपलब्धता, परिदृश्ये सूक्ष्मवातावरणविविधता च (Das et al. 2015) अद्वितीयसूक्ष्मनिवासस्थानानि निर्माति । आर्द्रतानिर्भराः, सामान्यतया नित्यहरिद्वर्ण वृक्ष प्रजातयः निम्नभूमियुक्तेषु उपत्यकेषु दृश्यन्ते, अतल्लीनमृदाभिः सह उच्चपर्वतशिखरेषु पतनशीला वृक्षाः दृश्यन्ते, तुल्यकालिकरूपेण शुष्कनिर्भराः नित्यहरिद्वर्ण जातयः मध्यमजलस्य उपलब्धतायुक्तेषु पर्वतसानुषु निवसन्ति (Pascal 1988; Krishnadas et al. 2016) । स्थलाकृतिकभेदाः पर्वतस्य पार्श्वे स्थितस्य आधारेण जातिभिः अनुभवितस्य ऋतुजलस्य उपलब्धतायाः सूक्ष्मपर्यावरणस्य च तापमानस्य अधिकं विविधतां जनयन्ति । एतेन एषः प्रश्नः भवति यत् सूक्ष्मनिवासस्थानस्य तापगुण क्षेत्र आभ्यता-अन्तरस्य च सह-उपस्थितवृक्षजातीनां मध्ये प्रकाशसंश्लेषण-तापीय-संवेदनशीलतायाः भेदेन सह कथं सम्बन्धः अस्ति?

प्रकाशसंश्लेषणस्य तथा पत्रछिद्रवाहकता (g_s) तापसंवेदनशीलतायाः ऋतु-अन्तरस्य लक्षणं ज्ञातुं वयं द्वयोः ऋतुयोः मध्ये नव-वृक्ष-जातीयानां समूहस्य कृते CO_2-उपग्रहणगतिः (A_{net}) तथा g_s-तापमान-प्रतिक्रियाः मापितवन्तः आदर्शतापमानस्य (T_{opt}) प्रकाशसंश्लेषणस्य दराः प्रकाशस्य, सामान्यपर्यावरणस्य CO_2 सान्द्रतायाः, *RH* इत्यस्य च अधीनं स्वस्य अधिकतमक्षमतायां (A_{opt}) भवन्ति । अतः शुष्क-आर्द्र-कालयोः प्रकाशसंश्लेषणस्य (A_{opt}) ऋतु-अन्तरस्य मापं दातुं शक्नोति । पत्रछिद्रवाहकता-चालकता-विविधतायाः सूचकरूपेण वयं T_{opt} इत्यस्य g_s-दरं मापितवन्तः, यत्र आदर्श-g_s अधिकतया पत्र-वातावरणस्य स्थितिः सूचयति तथा च अध्ययनकालद्वये प्रकाशपूरित -संश्लेषण-दरः अस्माकं अध्ययनं निम्नलिखितप्रश्नान् सम्बोधयति।

१) ऋतुकाले उष्णवलयवने सहचरवृक्षजातीषु शुष्क-आर्द्र-ऋतुषु प्रकाशसंश्लेषण-दरस्य, पत्रछिद्रवाहकतायाः च तापमान-प्रतिक्रियाः कियत्पर्यन्तं भिन्नाः भवन्ति ?

२) प्रकाशसंश्लेषणस्य दरस्य, रसस्य चालकतायां च ऋतुगतभेदाः प्रजातीनां भिन्न-भिन्न-स्थलाकृतिक-स्थानैः सह सम्बद्धाः सन्ति वा ?

आर्द्रऋतौ सर्वेषु वृक्षजातीषु प्रकाशसंश्लेषणस्य दरः अधिकः भविष्यति इति वयं पूर्वानुमानं कुर्मः । वयं पूर्वानुमानं कुर्मः यत् आर्द्र-शुष्क-ऋतुयोः प्रकाशसंश्लेषण-दर-अन्तराणि पर्वतशिखर-पर्णपात-जातीषु सर्वाधिकं, पर्वत-सानुजातेषु मध्यमं, उपत्यका-जातीषु च न्यूनं भविष्यति वयं अपेक्षामहे यत् शुष्कऋतौ Topt वर्धते, यथा पूर्वं किशोर वृक्षेषु विदितः (Kositsup et al. 2008; Slot and Winter 2017a) तथा प्रयोगात्मकतापनस्य प्रतिक्रियारूपेण (Crous et al. 2022) अवलोकितम्। तदतिरिक्तं वयं अपेक्षामहे यत् आर्द्रऋतौ T_{opt} सामान्यतया पत्रछिद्रवाहकता अधिका भविष्यति, परन्तु भिन्न-भिन्न-जल-प्रवेश-कार्यनीतिषु तथा स्थल-विशिष्ट-सम्बन्धेषु ऋतु-निर्भर-अन्तरस्य परिमाणं जातिषु भिन्नं भवितुम् अर्हति ।

२. सामग्रीः विधिः च

२.१. अध्ययनस्थलं वृक्षजातिः च

वयं भारतस्य कर्नाटकस्य उत्तरकन्नड मण्डलस्य मध्यसह्याद्री पर्वतश्रेणीपरिदृश्ये (१४.४७९१५७°, ७४.७५८३०४°, ५२३ मी.) एकस्मिन् वनस्थले मापनं कृतवन्तः (चित्रम् १) । अध्ययनस्थलस्य पर्वतीयः भूभागः अस्ति, यत्र उपत्यकेषु समुद्रतलात् ५००–५५० मी.पर्यन्तं, पर्वतशिखरेषु ५५०–६०० मी.पर्यन्तं च ऊर्ध्वता भवति वयं त्रयाणां स्थलाकृतिकस्थानानां प्रत्येकस्मिन् त्रीणि वृक्षजातीयसमूहानि चयनं कृतवन्तः । पर्वतशिखरेषु, येषु न्यूनवितरणघनत्वयुकताः पर्णपातीवृक्षजातयः प्रधानाः सन्ति, अस्माभिः चयनिताः प्रजातयः आसन्: - *Careya arborea* Roxb. (Lecythidaceae), *Terminalia chebula* Retz. (Combretaceae), *T. paniculata* B.Heyne ex Roth (Combretaceae) - सर्वे पर्णपाति वृक्षाः । शुष्कमृदाभिः सह पर्वतसानुषु वयं त्रीणि नित्यहरिद्वर्ण वृक्षजातयः चयनितवन्तः - *Memecylon umbellatum* Burm.f. (Melastomataceae), *Psydrax dicoccos*; (Gaertn.) Merr. (Rubiaceae), तथा च *Tetrapilus dioicus* Roxb. (Oleaceae) । अन्ते आर्द्रद्रोणीषु वयं त्रीणि नित्यहरिद्वर्ण वृक्ष प्रजातयः चयनितवन्तः - *Hopea ponga* Wall. (Dipterocarpaceae), *Knema attenuata* (Hook.f. & Th.)

Warb. (Myristicaceae), तथा *Garcinia cambogioides* var. *cambogioides* (Clusiaceae) । *C. arborea, T. paniculata* इत्यादयः पर्वतशिखरवृक्षाः वनअग्निप्रवणक्षेत्रैः सह सम्बद्धाः सन्ति, यदा तु *T. chebula* मिश्रितपर्णपातकवनेषु दृश्यते, यत्र मध्यसह्याद्री पर्वतश्रेणीः अस्य सर्वाधिकं उपयुक्तः निवासस्थानः अस्ति (Kailash et al., 2022) । पर्वतसानुषु *M. umbellatum* उत्तरसह्याद्री पर्वतश्रेणी मध्य-उच्चता-वनानां लक्षणं भवति, *Syzygium*-जातीयैः *Actinodaphne*-जातीयैः च सह सम्बद्धम् अस्ति, १००० मी.पर्यन्तं आर्द्र-शुष्क-वनेषु भवति (Shigwan et al. 2024), प्रायः *T. dioicus* इत्यनेन सह सह-उपस्थितं भवति । उपत्यकेषु *Hopea* जातयः अविक्षिप्तनित्यहरिद्वर्ण वनानि सूचयन्ति, अध्ययनस्थलस्य समीपे एकप्रधानस्थानानि अपि निर्माय दृश्यन्ते । *K. attenuata* तथा *Garcinia* इति जातिः आर्द्रद्रोणीनां लक्षणं भवति । अस्माकं अध्ययनक्षेत्रे पर्वतशिखरेषु मुक्तक्षेत्रेषु च पर्णपातीजातयः प्रधानाः सन्ति, यदा तु सानुषु उपत्यकासु च नित्यहरिद्वर्ण जातयः प्रधानाः सन्ति, येन स्पष्टं स्थलाकृतिकपृथक्त्वं प्रदर्श्यते तदपेक्षया पर्वतशिखरेषु, उपत्यकेषु च पर्णपातजातयः दुर्लभाः सन्ति । एतेन पृथक्करणेन पर्णपाती-नित्यहरिद्वर्ण जातीनां क्षेत्रान्तरेषु प्रत्यक्षतुलना कर्तुं अस्माकं क्षमता सीमितं जातम् । नव चयनितप्रजातयः सामूहिकरूपेण अस्मिन् पारिस्थितिकीतन्त्रे चयनितसामान्यवृक्षजातीनां प्रतिनिधित्वं कुर्वन्ति (Pascal 1988) तथा च सह्याद्री पर्वतश्रेणीपरिदृश्ये जलसम्बन्धानां सामान्यप्रतिमानं प्रतिबिम्बयन्ति (Krishnadas et al. 2021), यत् स्थलाकृतिकस्थानैः सह निकटतया सम्बद्धम् अस्ति । सानुभागे, भूभागे च वृक्षाणां विशिष्टवितरणस्य कारणात् प्रायः २–५ मी. भूमौ उपरि मापनार्थं उपयोक्तुं शक्यते स्म । प्रांशग्रहण कृतस्य प्रत्येकस्य वृक्षस्य कृते वयं मापनार्थं शाखां चित्वा वृक्षस्य क्षतिं न कृत्वा पृथक्करणं वा न कृत्वा भूमौ नत्वा पत्रमापनस्य सुविधायै पाशैः सुरक्षितं कृतवन्तः वयं प्रत्येकस्य प्रजातेः कृते न्यूनातिन्यूनं त्रीणि जैविकप्रतिकृतयः/प्रांशग्रहणानि वा वृक्षाणि मापितवन्तः, आर्द्रशुष्कऋतुयोः अभियानयोः समये समानवृक्षाणां मापनं कृतवन्तः।

२.२. अध्ययनकालाः

वयं द्वयोः अवधियोः मापनं कृतवन्तः - २०२० तमे वर्षे वर्षाऋतोः पश्चात् कालस्य प्रारम्भिककालः, २०२१ तमे वर्षे च शुष्कग्रीष्मकालः 'आर्द्रऋतुः' इति नाम्ना उल्लेखित प्रारम्भिकः वर्षऋतु-अभियानः २०२० तमस्य वर्षस्य नवम्बर-मासस्य मध्यभागपर्यन्तं, ४-मासस्य वर्षऋतु-कालस्य प्रायः १–१.५ मासानां अनन्तरं कृतः (चित्रम् १, पटलः १) । द्वितीयः अभियानः मार्चमासस्य अन्ते एप्रिलमासस्य मध्यभागपर्यन्तं यावत् आसीत्, सः प्रारम्भिकतः मध्यग्रीष्मकालपर्यन्तं (अतः ग्रीष्मकालः इति उच्यते) अनुरूपः आसीत् । ग्रीष्मकालस्य लक्षणं भवति यत् प्रतिमासं प्रायः ४–५ मासान् यावत् (डिसेम्बरमासात् मे-मासस्य मध्यभागपर्यन्तं वर्षऋतुस्य आरम्भपर्यन्तं) प्रायः १०० मि.मी. सामान्यतया एप्रिल मासस्य अन्ते मे-मासस्य आरम्भे च अस्मिन् प्रदेशे वायुतापमानं सर्वोच्चस्तरं प्राप्नोति । ग्रीष्मकालस्य माध्यम् अधिकतमं वायुतापमानं ३८.४ °C भवति, यत् वर्षाऋतोः पश्चात् कालस्य (३४ °C) अपेक्षया प्रायः ४ °C अधिकं उष्णं भवति । ग्रीष्मकालस्य सापेक्षिकं आर्द्रता (*RH*) ~७०.४% (५९.६–८२.३) आसीत् यदा तु वर्षाऋतोः पश्चात् काले ७५.६% (६५.३–८७.०) आसीत् अस्मिन् स्थले कुलम् माध्यमवार्षिकवृष्टिः प्रायः ४००० मि.मी. अध्ययनस्थले स्थापिते WatchDog २००० स्वचालित वर्तमान वातावरण स्थानकात् (Spectrum Technologies, Illinois, USA) तापमानस्य वर्षास्य च सञ्चिका: प्राप्ताः आर्द्रकाले वाष्पबलघातः (*VPD*) दिवाकाले २ किलोपास्कलपर्यन्तं नित्यं आसीत् किन्तु शुष्ककाले २ किलोपास्कलपर्यन्तं भिन्नः आसीत् पूरकचित्रे १ शुष्क-आर्द्र-कालयोः मध्ये वायु-तापमानस्य, *RH*, पत्र-वायु-वाष्पबलघातः-इत्यस्य च दैनिक-विविधतां दर्शयति ।

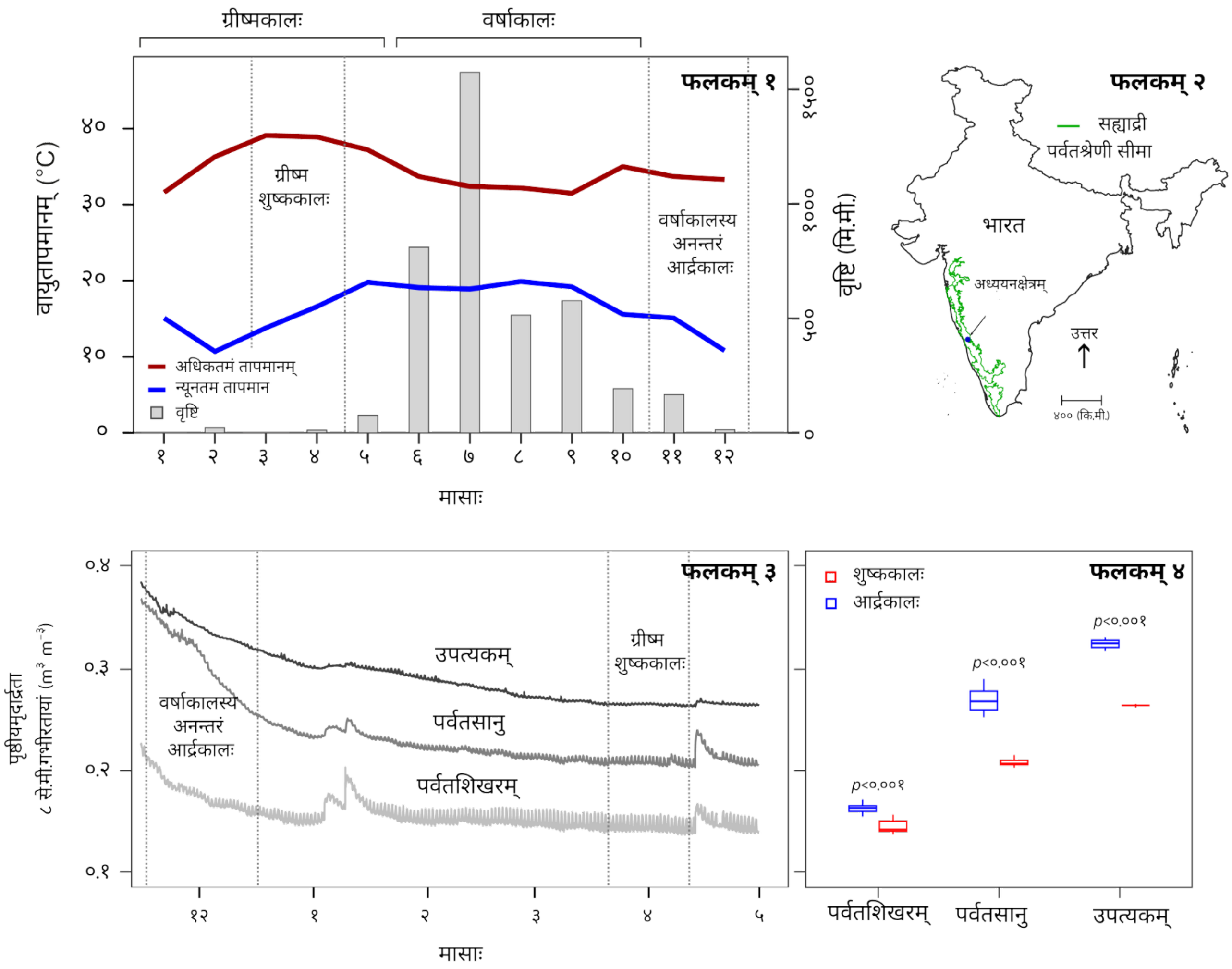

चित्र १: पटल १) भारतस्य सह्याद्री पर्वतश्रेणी अध्ययनस्थले मासिकवायुतापमानं (प्राथमिकअक्षः) वर्षा च (द्वितीय अक्षः)। दत्तांशः २०२०–२०२१ कालखण्डे एकत्रितस्य WatchDog २००० वर्तमान वातावरण स्थानकस्य सञ्चिकानां उपयोगेन गणित मासिक माध्यम् अस्ति । मानचित्रे (चित्रविभागः २) भारते अध्ययनस्थलस्य सह्याद्री पर्वतश्रेणी च सीमा दर्शिता अस्ति । पटल ३) परवर्षे मापितानां त्रयाणां स्थलाकृतिसमूहानां अनुरूपं त्रयाणां बिन्दूनां कृते पृष्ठीयमृदार्द्रतां दर्शयति । पटल ४ पटले ३ मध्ये प्राप्तानां त्रयाणां स्तरानाम् माध्यमपृष्ठभूमिआर्द्रतां दर्शयति ।

२.३. CO_2 उपग्रहणदर तापमान प्रतिक्रिया मापनम्

वयं A_{net}, प्रभाप्रत्युत्सर्ग पत्रकक्षः (Li-६४००-४०) इत्यनेन युक्तस्य पोर्टेबल इन्फ्रारेड गैस एनालाइजर (IRGA) Li-६४०० XT (LiCor, Lincoln, USA) इत्यस्य उपयोगेन CO_2 उपग्रहणदरस्य तापमानप्रतिक्रियां मापितवन्तः। वयं भूमौ प्रायः २–५ मी. उच्चतायां प्रौढवृक्षाणां सूर्यप्रकाशित शाखाभ्यः निम्न-मध्य-वितान पूर्ण-प्रौढ-स्वस्थ-पत्राणि मापितवन्तः । एकः विशिष्टः मापनक्रमः क्रमेण पत्राणां तापनं पत्रकक्षे भिन्न-भिन्न-तापमान-बिन्दुषु भवति स्म । प्रयुक्तानि पत्रस्य तापमानानि आसन् - २०, २४, २८, ३२, ३६, ४०, ४४, ४८ °C च । प्रकाश विकिरण स्तरं १००० μmol m$^{-२}$ s$^{-१}$, CO_2 सान्द्रता च ४०० μmol mol$^{-१}$ इति निर्धारितम् । संवेदनशील निवास स्थानस्य प्रकाशस्तरं सूचयितुं तापमान प्रतिक्रिया मापनस्य समये यन्त्रेण अभिलेखितानां बाह्य *PAR* (प्रकाशसंश्लेषणात्मक सक्रिय विकिरण) संवेदकदत्तांशस्य उपयोगः कृतः।

IRGA-मध्ये आर्द्रतायाः सघनीकरणं निवारयितुं वयं न्यूनतमं पत्रतापमानं ओष्णबिन्दु-बिन्दुस्थानतः उपरि ४ °C इति स्थापयामः, येन अस्माभिः मापनीयं न्यूनतमं तापमानं सीमितं जातम् वयं पत्राणि प्रारम्भिक पत्रम्यानस्थितौ न्यूनातिन्यूनं १० मि. स्थिरीकरणानन्तरं न्यूनातिन्यूनं ६–७ निमिशम् स्थिरस्थितेः अनन्तरं सञ्चिकानां अभिलेखनं कृतम् । सञ्चिकासंग्रहणानन्तरं पत्रस्य तापमानः ~४ °C अधिकं तापमानं यावत् समायोजितः ततः समानः स्थिरीकरणक्रमः अनुसृतः ।

यदा वयं उच्चतमं नाममात्रं तापमानं प्राप्तुं प्रयत्नम् अकरोम यत् यन्त्रं प्राप्तुं शक्नोति स्म तदा पत्रस्य तापमानं प्रायः अधिकतम संभव पत्रतापमानात् न्यूनं भवति स्म, येन अस्माभिः मापनीयस्य तापमान उपरितनं सीमा सीमितं भवति स्म । ग्रीष्मकालस्य पत्रस्य तापमानस्य (T_{leaf}) परिधिः २१.४–४५.० °C आसीत्, वर्षाऋतोः पश्चात् कालस्य कृते

च २३.२–४१.१ °C आसीत् । पत्रवायुस्य *RH* ६०±५% परिमितं भवति स्म । परन्तु ३५°C तः अधिके तापमाने शुष्क-आर्द्र-ऋतु-अभियानयोः *RH*-स्तरः ४०% इत्येव न्यूनः अभवत् । प्रकाशसंश्लेषण तापमान प्रतिसाद मापदण्डानां अनुमानं कर्तुं वयं जून इत्याख्यस्य प्रतिरूपं समायोजनम् कृतवन्तः । June (2004) इत्यनेन CO_2 उपग्रहणदर प्रतिक्रियायाः कृते निम्नलिखितरूपेण।

$$A_{net}(T_{leaf}) = A_{opt} \times e^{-\left(\frac{T_{leaf}-T_{opt}}{\Omega}\right)^2}$$ **समीकरणम् १**

अत्र A_{net} प्रकाशसंश्लेषक CO_2 उपग्रहणस्य दरः अस्ति । T_{opt} इति A_{net} इत्यस्य आदर्शतापमानमूल्यं तथा A_{opt} आदर्शतापमानस्य अथवा अधिकतम CO_2 उपग्रहणस्य दरः इति । Ω इति नियताङ्कः, यः वक्रस्य शिखरस्य विस्तारं प्रतिनिधियति, सः तापमानस्य अन्तरं भवति यस्मिन् तापमाने A_{net} T_{opt} तथा T_{opt} इत्यत्र स्वस्य मूल्यस्य ३७% यावत् पतति आदर्शतापमानात् अधः उपरि च तापमानेषु । Cunningham and Read (2002) इत्येतयोः परवलयिक तापमान प्रतिक्रिया वक्रस्य तुलने June (2004) इत्याख्यस्य असममितशिखर मानचित्राणम् भवति । इत्यनेन अस्माकं दत्तांशेषु विशेषतः शिखरस्य (T_{opt}) ग्रहणे उत्तमं गणितीयं कार्यं प्रदत्तम् । प्रत्येकं प्रजातिकालस्य कृते तापमान प्रतिक्रिया वक्राणि स्थापितानि, प्रतिकृतिं प्रत्येक वृक्षमापनं संयोजयित्वा (चित्रम् २, पटलम् १) । प्रत्येक वृक्षप्रतिकृतिदत्तांशयोः पृथक् पृथक् वक्राणि समायोजयित्वा प्रजातीनां कृते माध्यम् T_{opt} गणितम् । तस्य विपरीतम्, चित्रे २, फलक १ मध्ये दर्शिताः वक्राः सर्वेषां प्रतिकृतवृक्षदत्तांशस्य संयुक्तं समायोजयित्व प्रतिनिधित्वं कुर्वन्ति । पत्रछिद्रवाहकता (g_s) इत्यत्र तापमानस्य प्रभावस्य परीक्षणार्थं Akaike सूचनामापदण्डस्य (Cavanaugh and Neath 2019) उपयोगेन द्विघातपदयुक्तानां विना च रेखीयप्रतिमानानाम् तुलना कृता तथा च सर्वोत्तम समायोजन प्रतिरूपस्य उपयोगः कृतः वयं अवलोकितानां प्रक्रियाप्रतिक्रियाणां विविधतां दर्शयितुं पत्रतापमानस्य T_{leaf} विरुद्धम् g_s इत्यस्य चित्रणं प्रस्तुतामः। यतो हि g_s पत्रस्य तापमानप्रतिक्रियाभिः पर्यावरणीयकारकैः च प्रभावितः भवति, यत्र पत्रस्य परितः वायुस्य च मध्ये जलवाष्पविनिमयः अपि अस्ति, अतः वयं T_{leaf} इत्यस्य उपयोगेन g_s इत्यस्य प्रतिरूपणं प्राथमिकपूर्वसूचकरूपेण कृतवन्तः AIC मूल्यस्य आधारेण सर्वोत्तम समायोजयित्व रेखीय अथवा द्विघातदत्तांशसमायोजयित्व सम्बन्धानां चयनं कृतम् । तापमान-परिधिः पत्र-वायु-वाष्प-दाब-अभावे (*VPD*) तदनुरूप-परिवर्तनानां लेखानुरूपं वयं *VPD*-T_{leaf}-योः मध्ये रेखीय-प्रतिगमन-प्रतिरूपं समायोजनम् कृतवन्तः एतस्य प्रतिरूपस्य उपयोगेन वयं प्रत्येकस्य T_{leaf} मापनस्य *VPD* मूल्यानां गणनां कृत्वा तान् गौण-अब्सिस्सारूपेण चित्रणं कृतवन्तः । चित्र ३ पत्रकक्षे सापेक्षिक आर्द्रता *RH* नियन्त्रणद्वारा प्राप्तं *VPD* स्तरं प्रदर्शयति, यदा तु पूरकचित्र १ अध्ययनस्थले अभिलेखितानां दैनिक *VPD* दैनिकविविधता चित्रणं करोति ।

अनेकाः अध्ययनाः IRGA Li-६४०० इत्यस्मिन् सामान्यतया प्रयुक्तस्य दुर्बल-अवरोधित-उष्मायुग्मस्य उपयोगेन पत्र-तापमान मापनस्य त्रुटिविषये चिन्ताम् उत्पन्नं कृतवन्तः (Mott and Peak 2011; Still et al. 2019; Garen et al. 2022)। तथापि Docherty इत्यादयः (2023) इत्यनेन उच्चपरिमाणस्य पूर्वाग्रहस्य (Li-६४००) तथा अपेक्षाकृतं न्यूनपक्षपातस्य (Li-६७००) यन्त्रस्य उपयोगेन मापनात् प्राप्तेषु तापमान प्रतिक्रिया मापदण्डेषु भेदानाम् परीक्षणं कृतम्, तत्र कोऽपि सांख्यिकीयः अन्तरः न प्राप्तः । अस्माकं अध्ययने यतः मापनतापमानं साध्यपरिधिं व्याप्तवान् तथा च A_{net} सामान्यतया T_{opt} इत्यत्र अधिकतम A_{net} इत्यस्मात् परं न्यूनीकृतः, अतः A_{opt} इत्यस्य निरपेक्षमूल्यानि यन्त्रस्य तापमान पक्षपातेन प्रभावितानि न भवेयुः । यद्यपि वयं स्वीकुर्मः यत् T_{opt} इत्यस्य वास्तविकमूल्यानि अधिकं प्रत्यक्षतया प्रभावितानि भवितुम् अर्हन्ति तथापि संगृहीतदत्तांशेषु एते प्रभावाः समानाः सन्ति यतोहि मापनं समानयन्त्रस्य उपयोगेन कृतम् आसीत् अतः वयं यत् जातिव्यापीं भेदं निवेदयामः तस्य व्याख्या सावधानीपूर्वकं कर्तुं शक्यते।

२.४. पृष्ठीय मृदार्द्रता

तदनन्तरं वर्षे (२०२३–२०२४) त्रयः TMT-४ मानकदत्तांश लेखायन्त्राः (Wild et al. 2019) (TOMST, Prague, Czech Republic) सम्भाव्य स्थलाकृतिकस्तरयोः पृष्ठीय मृदार्द्रतायां (१५-निमिषा-अन्तराले मृदापृष्ठतः ७ सेमी गभीरतायां) महत्त्वपूर्णऋतुविविधतां अभिलेखितवन्तः पठनानि परिमाणात्मक मृदार्द्रतायां परिवर्तयितुं १.२५–१.२८ ग्राम/से.मी.। लेखायन्त्राः कार्यनीतिकरूपेण पर्वतशिखरस्य, सानुस्य, उपत्यकाक्षेत्रस्य च प्रतिनिधित्वार्थं स्थिताः आसन् तथा च मृदापृष्ठतः ८ से.मी.गभीरतायां १५ निमेषस्य आवृत्त्या सञ्चिकानां ग्रहणार्थं स्थापनम् कृताः आसन् ।

पृष्ठीयमृदार्द्रतायाः स्तरः सामान्यतया पर्वतशिखरेषु सर्वाधिकं न्यूनः आसीत्, तदनन्तरं सानुषु, उपत्यकेषु च (चित्रम् १, पटलाः ३, ४ च) आसन् । त्रयोऽपि स्तरानाम् कृते मृदार्द्रता ऋतुकाले भिन्ना आसीत्, शुष्कऋतुना अपेक्षया आर्द्रऋतौ अधिका आसीत् । पृष्ठीयमृदार्द्रतायाः बृहत्तमः ऋतुविविधता उपत्यकासु, प्रवणस्थानेषु च अभिलेखिता, तस्य विपरीतम्, पर्वतशिखरेषु न्यूनतमः ऋतुविविधता अवलोकिता उपत्यकायां शुष्कऋतुमृदार्द्रतायाः स्तरः आर्द्रऋतु सानुस्तरस्य तुलनीयः आसीत् ।

२.५. . दत्तांशविश्लेषणम्

R संस्करण ४.४.३ (R Core Team, 2025) इत्यस्मिन् तन्त्रांश उपयुज्य सञ्चिका विश्लेषणं कृतम् । तापमान प्रतिसादकार्यस्य कृते वयं ‘stats’ संकुलस्य ‘nls’ कार्यस्य उपयोगं कृतवन्तः यत् A_{net} तापमानफलकं समीकरणे १. T_{opt} तथा A_{opt} इत्येतयोः गणना समायोज्य गणितीयं प्रतिरूपम् कृता नियताङ्कः-माध्यमेषु प्रजाति-स्तरीय-ऋतु-अन्तराणां परीक्षणं युग्मित-टी-परीक्षायाः उपयोगेन कृतम् । T_{opt} विचरणे A_{opt}, T_{opt}, g_s च व्याख्यायमानाः द्विपक्षीयपुनरावृत्तिमापाः रेखीयमिश्रित-प्रभावविश्लेषणं (ANOVA) गणितीयं प्रतिरूपाः ‘nlme’ संकुलस्य ‘lme’ इत्यस्य उपयोगेन समायोजयित्व कृताः (Pinheiro et al. 2018)। ऋतुः, जातिः, पर्णप्रकारः च (पर्णपाती नित्यहरिद्वर्ण च) नियतप्रभावरूपेण समाविष्टः, वृक्षाः (वृक्षाः) च आदर्शेषु यादृच्छिकप्रभावरूपेण समाविष्टाः पूरकखण्डे २ सर्वाणि गणितीयं प्रतिरूपम् समायोजयित्व प्रस्तुतानि सन्ति। सर्वेषां साधनानां माध्य ± SEs सह प्रस्तुतं भवति तथा च महत्त्वपूर्णपरिणामाः ९५% विश्वासान्तरे (CI = ९५%, α = ०.०५) प्रस्तुताः सन्ति।

३. परिणामाः

३.१. प्रकाशसंश्लेषक CO२ उपग्रहणदरस्य आदर्शतापमानम्

नववृक्षजातीनां मूल्यानि संयोजयित्वा $CO_२$-उपग्रहणदरस्य माध्यम आदर्शतापमानं (T_{opt}) आर्द्र (३०.४३ ± ०.३१ °C) तथा शुष्क (३०.९३ ± ०.३१ °C) अवधिषु ($T_{४९}$ = ०.९९, p = ०.३२५) ऋतुकाले कोऽपि अन्तरः न दृश्यते स्म । आर्द्रकालस्य माध्यम् अधिकतम वायुतापमानस्य समीपे एव प्रजातेः माध्यम् T_{opt} आसीत् (पूरकचित्रम् ३), परन्तु शुष्ककाले प्रजातेः माध्यम् T_{opt} माध्यम् अधिकतम वायुतापमानात् प्रायः ३ °C न्यूनम् आसीत् । T_{opt}-भेदाः प्रजातीयैः सह ($F_{९}$ = ७.२१, p < ०.००१) महत्त्वपूर्णरूपेण सम्बद्धाः आसन्, परन्तु ऋतुसम्बद्धाः न आसन् ($F_{३}$ = ३.२, p = ०.०७) । तदतिरिक्तं मिश्रितप्रभावप्रतिरूपे प्रजातीनां ऋतुस्य च मध्ये कोऽपि महत्त्वपूर्णः अन्तरक्रियाः न दर्शिताः ($F_{९}$ = १.७१, p = ०.१०६) (पूरकखण्डः २ तथा चित्रम् ३)।

फलकम् १

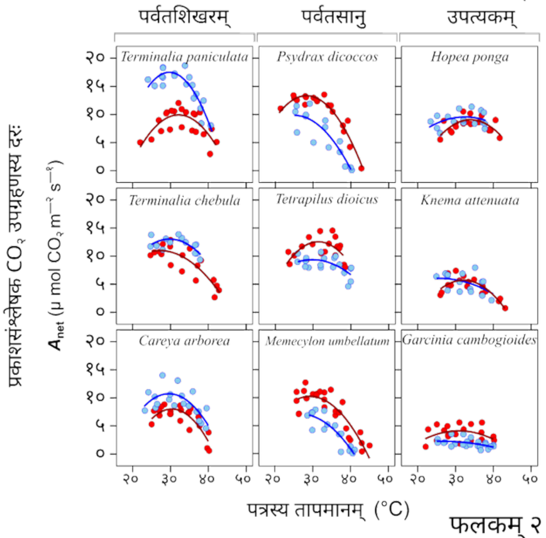

फलकम् २

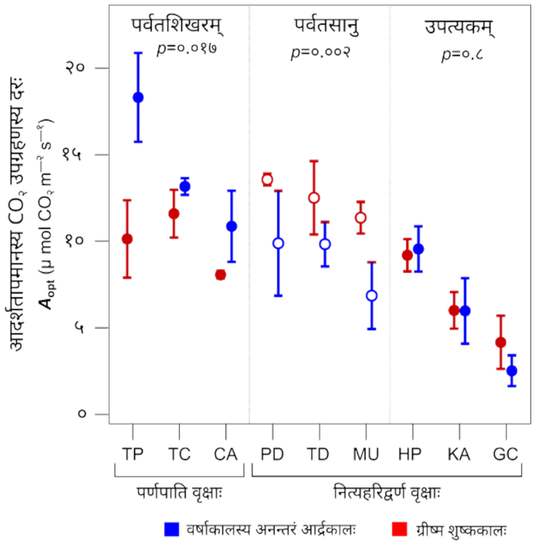

चित्र २: भारतस्य सह्याद्री पर्वतश्रेणीषु नव वृक्षजातीनां कृते प्रकाशसंश्लेषणीय $CO_२$ उपग्रहणगति तापमान प्रतिक्रिया वक्राणि (चित्रविभागः -१, ३x३ - सारणी) स्थाने एव मापितानि आसन् । नीलतत्त्वानि आर्द्रऋतुं, रक्ततत्त्वानि शुष्कऋतुं च सूचयन्ति । पटल २ प्रजातेः माध्यम A_{opt} दर्शयति, यत् आदर्शतापमानयोः अधिकतमं शुद्धं $CO_२$ उपग्रहणदरं (चित्रविभागः १ मध्ये वक्राणां शिखराः) भवति । मापनं १००० μmol $m^{-२}s^{-१}$, ४०० μmol mol−१ इत्यस्य [$CO_२$] सान्द्रता तथा *RH* ५०–६०% । परिधिमध्ये निर्वाहितम् पटल २ इत्यस्मिन् मुक्तवृत्ताः A_{opt} इत्यस्मिन् महत्त्वपूर्णऋतुविविधतायुक्तानि नित्यहरिद्वर्ण जातीयानि सूचयन्ति ।

पटल २ मध्ये दर्शितानां प्रजातीनां सङ्केताक्षराणि कोष्ठकेषु निम्नलिखितरूपेण सन्ति । *Terminalia paniculata* (TP), *Terminalia chebula* (TC), *Careya arborea* (CA), *Psydrax dicoccos* (PD), *Tetrapilus dioicus* (TD), *Memecylon umbellatum* (MU), *Hopea ponga* (HP), *Knema attenuata* (KA) and *Garcinia cambogiodes* var. *cambogioides* (GC)

३.२. आदर्शतापमानस्य CO२ उपग्रहणस्य दरः (A_{opt})

प्रजाति-स्तरस्य A_{net} तापमान प्रतिक्रियावक्राणि चित्रे २, फलक १ मध्ये प्रस्तुतानि सन्ति । माध्यम A_{opt}, मापितानां सर्वेषां नव प्रजातीनां संयोजनेन, आर्द्र-शुष्ककालयोः क्रमशः ९.६८ ± ०.५९ तथा ९.७१ ± ०.८३ μmol $CO_२$ $m^{-२}s^{-१}$ इत्यत्र समानः आसीत्, तथा च ऋतुकाले कोऽपि अन्तरः नासीत् A_{opt} । कृते मिश्रितप्रभावप्रतिरूपे महत्त्वपूर्णः प्रजातिप्रभावः ($F_७$ = २४.७, p < ०.००१) परन्तु ऋतुप्रभावः नासीत् ($F_१$= ०.००३, p = ०.९५) परन्तु ऋतुस्य प्रजातीनां च अन्तरक्रियाप्रभावः ($F_८$ = ८.९, p <०.००१) महत्त्वपूर्णः आसीत् । यत् सूचयति यत् A_{opt} इत्यस्मिन् ऋतुकाले भेदाः जातिनिर्भराः आसन् यदा पत्रस्य ऋतुत्वस्य परीक्षणं नियतप्रभावरूपेण कृतम् तदा पत्रचरित्रेण A_{opt} इत्यत्र महत्त्वपूर्णः प्रभावः दर्शितः ($F_१$ = १३.७, p <०.००१) । तथा च एतत् पत्रस्य ऋतुत्वस्य ऋतुस्य च अन्तरक्रियायां अपि दृष्टः ($F_१$ = १०.१, p = ०.००२) परन्तु केवलं ऋतुः महत्त्वपूर्णः पूर्वानुमानकः नासीत् ($F_१$=०.००३, p = ०.९५) । मिश्रितप्रभावप्रतिमानानाम् परिणामाः पूरकखण्डे २ सन्ति ।

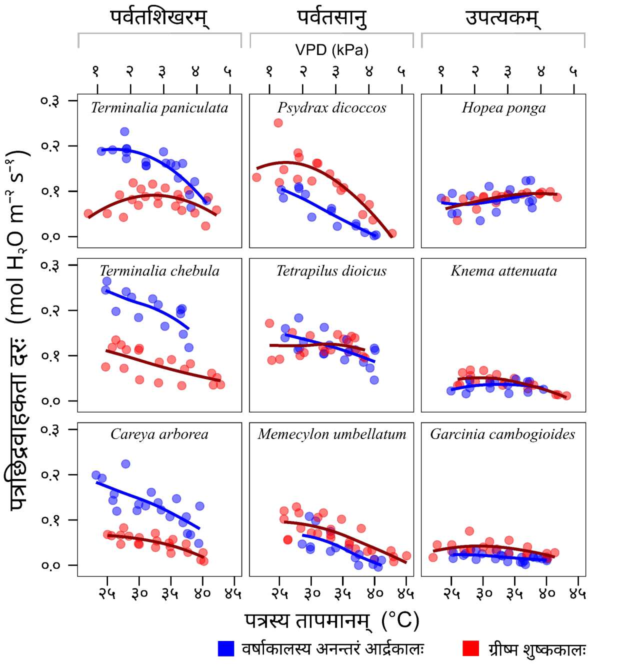

चित्र ३: वर्षाऋतोः पश्चात् आर्द्र-ग्रीष्म-शुष्ककालयोः मूलस्थले मापित भारतस्य सह्याद्री पर्वतश्रेणीषु नववृक्षजातीनां जलं प्रति पत्रछिद्रवाहकतायाः (g_s) तापमानप्रतिक्रिया। दत्तांश बिन्दुसम्बद्धं *VPD* g_s, T_{leaf} तथा *VPD* गणितीयं प्रतिरूपम् इत्यस्य आधारेण द्वितीयक x-अक्षे प्रतिनिधित्वं भवति । वक्राणि रेखीय-अथवा द्विघातीय-समायोजयित्व (AIC-आधारितं चयनितं सर्वोत्तम-समायोजयित्व) भवन्ति ।

पर्णपाती तथा नित्यहरिद्वर्ण वर्गयोः मध्ये A_{opt} महत्त्वपूर्णरूपेण भिन्नः आसीत् ($F_१$=११.९, p=०.००१) । पत्रऋतुनुसारं (अर्थात् पर्णपाती नित्यहरिद्वर्ण च) प्रजातीनां वर्गीकरणं कृत्वा भिन्नाः प्रतिमानाः ज्ञाताः । विशेषतया, पतनशील प्रजातयः शुष्कग्रीष्मकालस्य (९.९३ ± ०.६७ μmol $CO_२$ $m^{-२}s^{-१}$) तुलने आर्द्रस्य (१३.७७ ± १.१७ μmol $CO_२$ $m^{-२}s^{-१}$) समये अधिक A_{opt} सह महत्त्वपूर्णं ऋतुविविधतां ($T_{१४}$ = २.७५, p = ०.०१२) दर्शितवन्तः, यदा तु नित्यहरिद्वर्ण पत्रस्वभाव वृक्षाः ऋतुद्वयस्य मध्ये परिवर्तनं न कृतवन्तः ।

यद्यपि शुष्कऋतौ नित्यहरिद्वर्ण-पर्णपाती-जातीयानां मध्ये A_{opt} तुलनीयः आसीत् तथापि आर्द्रऋतौ ($T_{१६}$ = ४.५३,) पतनशील-प्रजातीनां (१३.७७ ± १.१७ μmol $CO_२$ $m^{-२}s^{-१}$) नित्यहरिद्वर्ण जातीयानां (७.५६ ± ०.७२ μmol $CO_२$ $m^{-२}s^{-१}$) अपेक्षया A_{opt} अधिकः आसीत् ($T_{१६}$ = ४.५३, p <०.००१) इति । नित्यहरिद्वर्ण जातयः अपि स्वस्य विशिष्टभूगोलस्थानस्य आधारेण द्वौ विशिष्टौ प्रतिक्रियाः दर्शितवन्तः । आर्द्रद्रोणिकानां विशिष्टानां त्रयाणां नित्यहरिद्वर्णजातीनां कृते A_{opt} शुष्कऋतुषु (६.४५±०.७१ μmol $CO_२$ $m^{-२}s^{-१}$) आर्द्रऋतुषु (६.०१±१.१ μmol $CO_२$ $m^{-२}s^{-१}$) च ऋतुकाले भिन्नः नासीत् । तस्य विपरीतम्, त्रयाणां सानुनां संयुक्तनित्यहरिद्वर्ण वृक्षाणां ऋतुस्य आधारेण महत्त्वपूर्णं भिन्नता आसीत्, आर्द्रस्य (७.९५ ± ०.७४ μmol $CO_२$ $m^{-२}s^{-१}$) तुलने शुष्कऋतुकाले (१२.३६ ± ०.४७ μmol $CO_२$ $m^{-२}s^{-१}$) अधिकं A_{opt} आसीत् ।

पत्र-स्तरीय-प्रकाश-दत्तांशैः A_{opt}-विविधतायाः व्याख्या न कृता (p = ०.४१) । पृष्ठीय मृदार्द्रता ऋतुप्रभावं विना A_{opt} ($F_२$ = ११.१५, p = ०.००४६) महत्त्वपूर्णतया प्रभावितवती, येन प्रजातीनां मध्ये A_{opt} भेदस्य आंशिकरूपेण व्याख्या कृता ऋतुभेदयुक्ताः त्रयः नित्यहरिद्वर्ण जातयः अधिकतया शुष्कस्थलेषु सम्बद्धाः आसन्, ते च पर्वतप्रदेशेषु दृश्यन्ते स्म । तस्य विपरीतम्, पर्वतप्रदेशैः सह सम्बद्धाः पर्णपातीजातयः आर्द्रऋतौ अधिकं A_{opt} दर्शयन्ति स्म । समग्रतया, पतनशील प्रजातयः तथा च सानुषु शुष्कसम्बद्धाः त्रीणि नित्यहरिद्वर्ण जातयः A_{opt} इत्यत्र महत्त्वपूर्णाः विपरीतरूपेण च ऋतुभेदाः दर्शिताः ।

३.३. आदर्शतापमानस्य पत्रछिद्रचालकतादरस्य भिन्नता

तापमानस्य प्रति पत्रस्य पत्रछिद्रप्रतिक्रिया जातिषु भिन्ना आसीत् (चित्रम् ३) । T_{opt} -मध्ये g_s-जातीनां मध्ये भिन्नता आसीत् (F_6 = ३.९, p = ०.०२७) परन्तु शुष्क-आर्द्र-कालयोः मध्ये न, यत् भिन्न-भिन्न-जातीय-प्रतिक्रियाः सूचयति । पर्णपातयुक्ताः पर्वतशिखरजातयः शुष्ककाले न्यूनतरं g_s दर्शितवन्तः । येन सूचितं यत् एताः प्रजातयः शुष्ककाले पत्रस्य पत्रछिद्रजलवाहकता हानिं नियन्त्रयन्ति स्म त्रयाणां उपत्यकासम्बद्धानां नित्यहरिद्वर्णजातीनां g_s प्रतिक्रिया ऋतुकाले भिन्ना नासीत् । सानुसम्बद्धानां नित्यहरिद्वर्ण जातीनां कृते g_s प्रतिक्रिया मिश्रिता आसीत् । विशेषतः *Tetrapilus dioicus* इत्यनेन ऋतुविविधता नासीत्, अन्यजातीषु (*P. dicoccus* तथा *M. umbellatum*) शुष्ककाले अधिकं g_s दर्शितवान् (विशेषतः *P. dicoccus* इत्यस्मिन् स्पष्टम्)

४. चर्चा

भारतस्य सह्याद्री पर्वतश्रेणीषु उष्णवलयवनवृक्षजातीनां समूहे वयं T_{opt} इत्यत्र आदर्शतापमानस्य, प्रकाशसंश्लेषणदरेषु A_{opt} तथा g_s इत्येतयोः ऋतुविविधतायाः मापनं कृतवन्तः। पतनशील प्रजातयः आर्द्रऋतौ निरन्तरं अधिकं A_{opt} दर्शितवन्तः यदा जलस्य प्रचुरता आसीत् तदा अधिक पत्रछिद्रवाहकता दरेन चालितम् । तस्य विपरीतम्, परीक्षितानां नित्यहरिद्वर्ण जातीनां कृते A_{opt} तथा g_s इत्येतयोः ऋतुविविधता स्थलाकृतिकस्थितेः उपरि निर्भरं भवति स्म । पर्णपातीजातीनां कृते यत् प्राप्तम् तस्य विपरीतम्, पर्वतसानुभ्यः नित्यहरिद्वर्ण जातीनां शुष्कग्रीष्मऋतौ अधिकं A_{opt} आसीत् । शुष्कऋतौ अपि जलसीमानां अनुभवं न कृतवन्तः उपत्यकाभ्यः नित्यहरिद्वर्ण जातयः A_{opt}, g_s इत्येतयोः ऋतुविविधतां न दर्शितवन्तः । अस्माकं परिणामाः जलस्य उपलब्धता, सूक्ष्मवातावरणस्थितिः, पत्रस्य ऋतुत्वं, पत्रलक्षणं, प्रकाशसंश्लेषणस्य तापीयपरिवर्तनशीलता च इत्येतयोः उपरि ऋतुअवलम्बित दीर्घकालीन वातावरणविविधतायाः जटिलप्रभावं प्रदर्शयन्ति ।

४.१. प्रकाशसंश्लेषणस्य ऋतुत्वे स्थलाकृतिकस्थितेः प्रभावः

परीक्षितेषु त्रयेषु निवासस्थानेषु, पर्वतशिखरेषु, सानुषु, उपत्यकासु च वृक्षाणां चयनं स्पष्टतया भिन्नम् आसीत् (चित्रम् ४) । एतत् भुप्रदेशे A_{opt} इत्यस्य भिन्नता उष्णवलयस्थलेषु (Harris and Medina 2013) समशीतोष्णस्थलेषु (Tange 1996) अध्ययनस्य सदृशं भवति । सानुपार्श्वे विशेषतः शुष्कऋतौ भूजलस्य उपलब्धता भिन्ना भवति । भूजलस्य उपलब्धता पर्वतशिखरे सर्वाधिकं न्यूना, पर्वतपार्श्वे मध्यवर्ती, उपत्यकाक्षेत्रेषु च सर्वाधिकं भवति । अस्माकं पृष्ठीयमृदार्द्रतामापनात् प्राप्तेषु परिणामेषु ज्ञातं यत् पर्वतशिखरभूमिः जलस्तरस्य संपर्कं प्राप्नोति यत् द्वयोः अवधियोः मध्ये अल्पं भिन्नं भवति, यदा तु सानुषु उपत्यकासु च मृदासु जलं आर्द्रऋतौ महतीं वर्धते यद्यपि पृष्ठीयमृदार्द्रतायाः A_{opt} इत्यत्र महत्त्वपूर्णः प्रभावः दृश्यते स्म तथापि सम्बन्धः ऋतुप्रभावं न दर्शितवान् । एतेन एतेषां जातिषु विषममूलीकरणं ऋतुकाले भिन्नजलप्रवेशगहनता च सूचिता यस्याः अन्वेषणस्य आवश्यकता वर्तते ।

४.२. केचन नित्यहरिद्वर्ण वृक्षेषु वर्षाऋतुः अपेक्षया उष्णऋतुषु अधिकतम प्रकाशसंश्लेषणं कुर्वन्ति ।

अस्माभिः सहचरवृक्षजातीषु आदर्शतापमानयोः प्रकाशसंश्लेषणस्य दरयोः प्रजातिव्यापी शुष्क-आर्द्रऋतु-अन्तराः प्राप्ताः । पूर्वाध्ययनस्य अनुरूपं, आर्द्रऋतौ पर्णपातीवृक्षजातीनां A_{opt} अधिकः आसीत्, येन सूचितं यत् एताः प्रजातयः प्रकाशसंश्लेषणं अधिकतमं कर्तुं आर्द्रऋतुषु जलस्य उपलब्धतायाः उपयोगं कुर्वन्ति (Eamus et al. 1999; Craven et al. 2011)। तथैव पनामा-ऑस्ट्रेलिया-देशयोः नित्यहरिद्वर्ण-पर्णपात-प्रजातयः सम्मिलिताः किशोर वृक्ष-अध्ययनेन आर्द्रऋतुषु प्रकाशसंश्लेषणस्य अधिकानि दराः ज्ञाताः (Montagu and Woo 1999; Craven et al. 2011) ।

तदपेक्षया नित्यहरिद्वर्ण जातीनां विशिष्टप्रतिक्रियाद्वयं प्रदर्शितम् । त्रयाणां नित्यहरिद्वर्ण जातीनां A_{opt} शुष्क-आर्द्र-कालयोः कृते समानः आसीत्, Cai et al. (2009) इति । एतत् आश्चर्यं न भवति यतोहि एतेषु जातिषु अपि शुष्कऋतौ स्थलाकृतिकवर्गेषु सर्वाधिकं जलस्य उपलब्धता आसीत् (चित्रम् १) परन्तु अस्माकं अपेक्षायाः विपरीतम् अन्येषां त्रयाणां नित्यहरिद्वर्ण जातीनां कृते आर्द्रऋतुः अपेक्षया शुष्कऋतौ A_{opt} अधिकः आसीत् । एषः व्यवहारः वेनेजुएलादेशस्य वनानां निष्कर्षाणां सदृशः अस्ति, यत्र शुष्कऋतौ केषुचित् नित्यहरिद्वर्णेषु प्रकाशसंश्लेषणस्य दरः अधिकः आसीत् (Ávila-Lovera et al. 2019), यत् सूचयति यत् नित्यहरिद्वर्ण वृक्षाणां मूलं तस्मिन् स्थाने पर्णपातीवनस्पतयः अपेक्षया गभीराः सन्ति आर्द्रकालस्य उच्चतर प्रकाशसंश्लेषणस्य सामान्यप्रवृत्तेः विपरीतम् (Mujawamariya et al. 2023), एते नित्यहरिद्वर्ण वृक्षाः उष्णशुष्कस्थितौ वर्धन्ते सीमितजलस्य उपलब्धतायाः प्रति तेषां प्रतिक्रियाः, सम्भवतः गहनजलस्रोतानां उपलब्धतायाः कारणात् (Nie et al. 2011) अथवा अन्यतन्त्राणि यथा तापीय-अभ्यस्तीकरणम्, उच्च-तापमानस्य ताप-गुणविशिष्ट क्षेत्र प्राधान्यं-प्राथमिकताम् सूचयन्ति ।

उच्चवार्षिकवृष्टिः अथवा अल्पशुष्ककालयुक्तेषु केषुचित् उष्णवलय वनेषु प्रतिरूपण-अध्ययनेन ज्ञायते यत् प्रकाशसंश्लेषण क्रियाकलापः (नित्यहरिद्वर्ण वृक्षाणि सहितम्) ऋतुअवलम्बिकता न्यूना भवति अथवा शुष्ककालेषु शिखरं प्राप्नोति (Uribe et al. 2021) । भारतस्य ब्राजीलस्य च केषुचित् किशोरवृक्ष-अध्ययनेषु शुष्ककालेषु प्रकाशसंश्लेषणस्य अधिकानि दराः ज्ञाताः (Ribeiro et al. 2009; Abhilash and Devakumar 2023) । प्रकाशसंश्लेषणस्य प्रतिनिधिरूपेण क्लोरोफिल् प्रतिदीप्तिम् उपयुज्यमानेन अन्येन अमेजन-अध्ययनेन (Green et al. 2020) । अमेजन-वने केषुचित् आर्द्रभागेषु शुष्ककालेषु प्रकाशसंश्लेषणस्य वृद्धिः अवलोकिता, अस्मिन् काले

कनिष्ठ/नवीनपत्राणां कारणं प्रवृत्तिः अस्ति तस्य विपरीतम् अस्माकं दत्तांशः ऋतुविविधतां, अधिकतमपत्रवयोः, पत्रस्वभाववर्गः इत्यादीन् संयोजनान् च न दर्शयति । यथा, शुष्क-अनुकूलितस्य नित्यहरिद्वर्ण जातेः *Memecylon umbellatum* इत्यस्य शुष्कऋतौ किञ्चित् कनिष्ठानि पत्राणि आसन्, अन्ययोः शुष्क-अनुकूलितजातयोः प्राचीनतराणि पत्राणि आसन् (पूरकचित्रम् ४) एतेन भिन्नता सूचयति यत् पत्रस्य आयुः (पत्रस्य ऋतुत्वं) शुष्कऋतुस्य प्रकाशसंश्लेषणस्य अधिकस्य दरस्य पूर्णतया व्याख्यां न करोति । एवं पत्रस्तरीय प्रकाशसंश्लेषणस्य शुष्कऋतुवृद्धौ सम्बद्धानां तन्त्राणां विषये अग्रे संशोधनस्य आवश्यकता वर्तते ।

अस्माकं अध्ययनं नित्यहरिद्वर्ण वृक्षेषु A_{opt} इत्यस्मिन् ऋतुविविधतां त्रयाणां प्रजातीनां कृते मापितं यत् सामान्यतया सानुषु अपेक्षाकृतशुष्कक्षेत्रेषु दृश्यते (Pascal 1988; Krishnadas et al. 2021)। एते भिन्नाः स्थलाकृतिकसम्बन्धाः एतेषु जातिषु जलअभिगमनकार्यनीतिषु, मूलगहनेषु च भेदं सूचयन्ति । अध्ययनैः प्रदर्शितं यत् सदापर्णा वृक्षाः वर्षपर्यन्तं प्रकाशसंश्लेषणं धारयितुं प्रायशः गभीरतरजलस्तरान् अधिगच्छन्ति (Hasselquist et al. 2010; Brinkmann et al. 2019)। शङ्क्यते यत् शिखरप्रदेशेषु, यत्र मृत्तिकायाः अल्पगम्भीरता प्रायः दृश्यते, जलस्य उपलब्धिः तुलनया न्यूना भवति, यः तलप्रदेशैः च उपत्यकाभिः च, यत्र मृत्तिकायाः अधिकगम्भीरता भवति, अपि तुलना क्रियते (Guha and Jain 2020), अधिकजलस्य उपलब्धतायाः सह । समस्तं संक्षिप्य वयं स्थलस्थे प्रौढ वृक्षेषु कृतपरिमाणानां प्रमाणानि योजयामः, यत् केचन शाश्वतपर्णिनः वृक्षाः शुष्कतर ऋतु परिस्थितिभिः सह सम्बद्धाः सन्तः उष्णशुष्ककाले, नवने काले अपि, ऊष्मसाम्ये उच्चतरं प्रकाशसंलेषणदरं प्राप्तवन्तः इति।

४.३. पत्रछिद्रवाहकतायां ऋतुविविधता

अस्माभिः ज्ञातं यत् अस्माभिः अध्ययनं कृतानां प्रजातीनां मध्ये तापमानस्य (प्रकाराः ऋतुः च) प्रति पत्रछिद्रवाहकता-प्रतिक्रियाः भिन्नाः सन्ति (चित्रम् ३) । एकं कारणं अस्ति यत् प्रत्येक जातीनां प्रदर्शनं भिन्नजलअभावस्थित्या प्रभावितं भवति (Schwartz et al. 2022), अस्माकं अध्ययने जलस्य उपलब्धतायां ऋतुकालान्तरं जलस्य उपलब्धतायां दीर्घकालीनभेदस्य प्रॉक्सीरूपेण कार्यं करोति सूक्ष्मवातावरणेषु अपि एतादृशाः भेदाः दृश्यन्ते (Chitra-Tarak et al. 2021; Ding et al. 2021) । जलप्रवेशस्य प्रकाशसंश्लेषण कार्यनीत्याः स्थलाकृतिकभेदाः ग्रीष्मकाले जलस्य अभावस्य प्रभावात् केषाञ्चन प्रजातीनां रक्षणं कर्तुं शक्नुवन्ति (Esteban et al. 2021)।

g_s प्रतिक्रियायां ऋतुकालिकं स्थलाकृतिकं च भेदं समर्थयन् अध्ययनं कृतानां वृक्षजातीनां मध्ये विविधाः तापमानप्रतिक्रियाः ऋतुविविधताश्च प्राप्नुमः । यथा अपेक्षितं, त्रयोऽपि पर्णपातीजातीयानां कृते, g_s सामान्यतया तापमानेन सह न्यूनीकृताः, सामान्यतया शुष्कऋतुस्य अपेक्षया आर्द्रऋतौ अधिकानि g_s प्राप्नुवन्ति, अमेजोनियादेशस्य ऋतुअवलम्बित शुष्कवनानां अध्ययनस्य सदृशं (Vourlitis et al. 2008; Sendall et al. 2009) तस्य विपरीतम् नित्यहरिद्वर्ण जातयः अधिकविविधप्रतिक्रियाः प्रदर्शितवन्तः । प्रथमं, नित्यहरिद्वर्ण जातीषु शुष्क-आर्द्र-ऋतुषु सामान्यतया g_s-प्रतिक्रिया समाना आसीत्, परन्तु एकः नित्यहरिद्वर्ण-जातिः, *Psydrax dicoccos*, ऋतुकाले भिन्नं प्रतिक्रियां दर्शयति स्म, शुष्कऋतौ च अधिकानि g_s अभिलेखयति स्म । अध्ययनं कृतानां अन्यजातीनां विपरीतम् यद्यपि शुष्कऋतौ *Memecylon umbellatum* g_s अपि वर्धते स्म तथापि तापीय अधिकतमे आर्द्रऋतुस्य g_s इत्यस्य तुलने सांख्यिकीयदृष्ट्या तुच्छः आसीत् अन्यः नित्यहरिद्वर्णः प्रजातिः, *Hopea ponga*, यद्यपि ऋतुत्वस्य प्रति उदासीनः, तथापि तापमानेन सह g_s इत्यस्य वृद्धिः अभिलेखिता, सम्भवतः वाष्पीकरणीयशीतलनद्वारा तापसंरक्षणतन्त्रस्य सूचकः (Urban et al. 2017) एषा प्रजातिः व्यापकं तापमानप्रतिक्रियावक्रं (चित्रम् १, पटलम् १) अभिलेखितवती यत्र अधिकतापमानेन सह A_{net} इत्यस्य कोऽपि न्यूनता नासीत् तथा च परितः प्रबलक्षेत्रेषु प्राप्ता, सम्भवतः अस्मिन् प्रजातौ विविधजलप्रयोगस्य पत्र-तापमान विनियमनकार्यनीतिं च सूचयति इदं ज्ञातव्यं यत् थाईलैण्ड्देशस्य अपेक्षाकृतं शुष्कं उष्णवलयवने मापितायाः *H. ferrea* इत्यस्य भिन्नप्रजातेः (Ishida et al. 2014) शुष्ककालस्य महत्त्वपूर्णं क्षयः अभवत्, यत्र अस्माभिः ज्ञातं।

४.४. पत्राणां ऋतुत्वं, प्रकाशस्य उपलब्धता, सूक्ष्मवातावरणविविधता च

अमेजनस्य अध्ययनेन प्रकाशसंश्लेषणस्य ऋतुप्रवृत्त्या सह पत्रस्य ऋतुत्वं सम्बद्धम् अस्ति (Wu et al. 2016; Chen et al. 2020) । तथापि अस्माकं दत्तांशः (पूरकचित्रम् ४) प्रकाशसंश्लेषण विविधतायाः व्याख्याने पत्रऋतुत्वस्य कृते केवलं दुर्बलभूमिकां सूचयति । उल्लेखनीयं यत्, ग्रीष्मकालीन (शुष्क) कालखण्डे पर्णपातीवृक्षाणां पत्राणि कनिष्ठानि आसन् (पूरकचित्रम् ४) । यद्यपि सामान्यतया प्रौढपत्राणाम् अपेक्षया युवानां पत्राणां उपग्रहणस्य दरः अधिकः भवति (उदा. Green et al., (2020)), अस्माकं अध्ययने पर्णपातीजातीनां A_{opt} मूल्यानि आर्द्रकाले अधिकानि आसन् यदा शुष्ककालस्य तुलने पत्राणि प्राचीनानि आसन्, येन एतेषां प्रजातीनां कृते पत्रवयोः अपेक्षया जलस्य उपलब्धतायाः सशक्तं नियन्त्रणं सूचितं भवति । पनामादेशे (Kitajima et al. 1997) एकस्मिन् अध्ययने शुष्ककालेषु प्रकाशसंश्लेषणस्य वृद्धिः (O_2 विकासदररूपेण मापिता) - जलअभावस्य अभावे अधिकप्रकाशस्य उपलब्धतायाः कारणम् - यदा अस्माकं निष्कर्षाः अस्य प्रतिरूपस्य विरोधं कुर्वन्ति अस्माकं अध्ययने मापनस्य समये बाह्य-IRGA-संवेदकेन अभिलेखितं पत्र-स्तरीयं प्रकाश मापनं (*PAR*), A_{opt}-विविधतायाः सह कोऽपि महत्त्वपूर्णः सम्बन्धः न दर्शितवान् । परीक्षितेषु पर्यावरणीय चरेषु (प्रकाशस्य उपलब्धता तथा पृष्ठीयमृदार्द्रता) केवलं पृष्ठीयमृदार्द्रता तापीयआदर्शतापमानयोः प्रकाशसंश्लेषणदराणां कृते सीमितव्याख्यात्मक शक्तिं प्रदर्शयति स्म ।

अग्रे परीक्षणार्थं यत् A_{opt} इत्यस्मिन् ऋतुविविधता मूलस्यगहनतायाः सह सम्बद्धा अस्ति वा, अस्माभिः अस्माकं अध्ययनक्षेत्रस्य (Gloor et al. 2023) स्वतन्त्रतया उपलब्धानां सञ्चिकानां उपयोगः कृतः यत् अपराह्णे प्रातःकाले च पर्णजलसंभाव्यदत्तांशस्य अन्तरस्य परीक्षणं कृतम् तथा च A_{opt} इत्यत्र

४.५. अनावृष्टिसंवेदनशीलतां अवगन्तुं निहितार्थाः

अस्माकं परिणामाः सहचरवृक्षजातीनां मध्ये विविधाः प्रकाशसंश्लेषणात्मकाः जलप्रयोगकार्यनीतयः च प्रकाशयन्ति, ये तेषां स्थलाकृतिकस्थित्या प्रभाविताः सन्ति । आर्द्र-सम्बद्ध-उपत्यक-नित्यहरिद्वर्ण वर्षपूर्ण उथल-भूजल-प्रवेशस्य लाभः भवितुं शक्यते, यदा तु पर्वत-शिखर-पर्णपात-जातयः शुष्क-ऋतु-जल-अभावस्य अधिकं दुर्बलाः भवितुम् अर्हन्ति शुष्क-सम्बद्धाः प्रवणनित्यहरिद्वर्ण पत्रस्वभाव वृक्षाः शुष्कऋतुप्रकाशसंश्लेषणस्य दरं अधिकं दर्शितवन्तः । सर्वाणि प्रजातयः पत्रछिद्रवाहकता-विनियमनं प्रदर्शयन्ति यत् जल-हानि-सापेक्षतया कार्बन-लाभस्य परिवर्तनशीलता स्वाभाविकतया करोति (Andriyas et al. 2021), यत्र अधिक-प्रभाविणः अनुकूलन-सक्षम-लक्षणाः सन्ति, ते जल-सीमित-स्थितौ अधिकं अनावृष्टि-सहिष्णुः भवन्ति परन्तु एतत् ज्ञातव्यं यत् अनावृष्टिसहिष्णुतायां पत्रछिद्रव्यवहारात् परं अन्यतन्त्राणि वा पारिस्थितिकी-अनुकूलनानि अपि सम्मिलिताः भवितुम् अर्हन्ति, विशेषतः चरम-वातावरणेषु अथवा समुदाय-स्तरस्य (Blonder et al. 2023) । सह्याद्री पर्वतश्रेणीषु पूर्वानुमानित-अतिरिक्तजलस्थितेः कोऽपि प्रभावः न ज्ञातः। यद्यपि पृष्ठीयमृदार्द्रतामापनेन किञ्चित् अन्वेषणं प्राप्यते तथापि ऋतुजलप्रवेशं पूर्णतया अवगन्तुं जातिषु प्रभावी मूलगहनतायाः भिन्नता तथा मूलगहनतायाः सह मृदायार्द्रता अत्यावश्यकी भवति ।

अन्तर्गतं (Sarkar and Maity 2022) प्रकाशसंश्लेषणलाभाः वर्धिताः जलसंरक्षणस्य अनुकूलाः भवितुम् अर्हन्ति ।

जलस्य उपलब्धतायाः, जातिविशिष्टलक्षणस्य, स्थलाकृतिकविविधतायाः च मध्ये अन्तरक्रियाणां अवगमनं दीर्घकालीन वातावरण परिवर्तनस्य वनप्रतिक्रियाणां पूर्वानुमानार्थं महत्त्वपूर्णम् अस्ति । उदाहरणार्थं जलप्रवेशस्य प्रकाशसंश्लेषण कार्यनीत्याः स्थलाकृतिक विविधता जलस्य अभावात् केषाञ्चन प्रजातीनां रक्षणं कर्तुं शक्नोति (Esteban et al. 2021; Kühnhammer et al. 2023), यत् सूचयति यत् अनावृष्टिः समुदायस्य अन्तः प्रजातयः भिन्नरूपेण प्रभावितं करिष्यति यद्यपि ऋतुविविधता भविष्यस्य तापनस्थितीनां पूर्णतया प्रतिकृतिं न कर्तुं शक्नोति तथापि ते प्रजातिप्रक्रियाणां लक्षणअनुकूलनस्य जलअभावस्य प्रति तेषां विविधसंवेदनशीलतायाः च अन्वेषणं प्रददति (Janssen et al. 2020)।

अस्माकं निष्कर्षाः उष्णवलय वनेषु वर्धमानेषु प्रौढवृक्षेषु ऋतुअवलम्बित अनावृष्टिसहिष्णुतायाः परिवर्तनशीलतायाः च तन्त्राणि अवगन्तुं योगदानं ददति, विशेषतः ये अल्पगहन जलस्तरेन सह सम्बद्धाः सन्ति ये महत्त्वपूर्णानि अनावृष्टि आश्रयस्थानं प्रदातुं शक्नुवन्ति (Costa et al. 2023)।

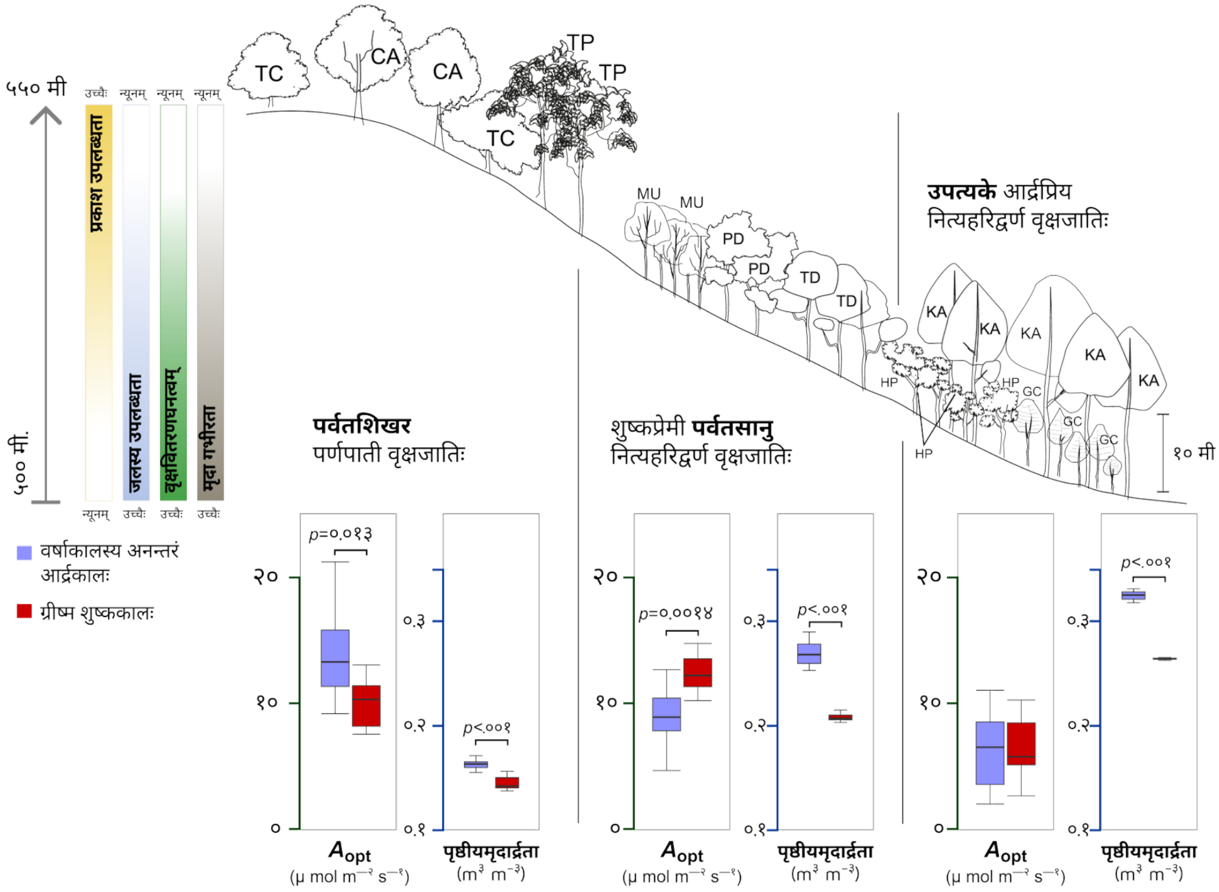

चित्र ४: भारतस्य सह्याद्री पर्वतश्रेणीषु अध्ययनं कृतानां वृक्षजातीनां वितरणं तथा आदर्शतापमानस्य (हरितक्षैतिजअक्षस्य) तथा पृष्ठीयमृदार्द्रतायां (नीलक्षैतिजअक्षस्य, m^3 m^{-3} मध्ये) प्रकाशसंश्लेषणस्य दरस्य भिन्नता आयतरूपेण चित्रणं कृता अस्ति । उपरि वामभागे आयताः वृक्षघनत्वे, विकिरणस्तरस्य, पर्वतशिखरात् उपत्यकाक्षेत्रपर्यन्तं जलस्य उपलब्धतायां च सापेक्षिकपरिवर्तनं सूचयन्ति । जातिसङ्केताः कोष्ठकेषु निम्नलिखितरूपेण सन्ति । *Terminalia paniculata* (TP), *Terminalia chebula* (TC), *Careya arborea* (CA), *Psydrax dicoccos* (PD), *Tetrapilus dioicus* (TD), *Memecylon umbellatum* (MU), *Hopea ponga* (HP), *Knema attenuata* (KA) and *Garcinia cambogioides* var. *cambogioides* (GC)

५. सारांशः

भारतस्य मध्य सह्याद्री पर्वतश्रेणीषु ऋतुशुष्कउष्णवल यवनस्थले शुष्कग्रीष्मकालस्य प्रारम्भिक वर्शऋतु उत्तरस्य आर्द्रकालस्य च कालखण्डे मापिते प्रकाशसंश्लेषणस्य तापमानप्रतिक्रियायां महत्त्वपूर्णाः अन्तरविशिष्टभेदाः ज्ञाताः वयं पर्वतशिखरेषु विभिन्नस्थानेषु दृश्यमानानां वृक्षजातीनां मध्ये आदर्शतापमानयोः CO_2-ग्रहण-दरयोः विशिष्टानि प्रतिमानाः अवलोकितवन्तः पर्णपातीजातयः आर्द्रऋतौ प्रकाशसंश्लेषणस्य दरं अधिकं प्रदर्शितवन्तः, येन तेषां विशिष्टऋतुरूपं प्रतिबिम्बितम् । तस्य विपरीतम् अस्माभिः नित्यहरिद्वर्ण जातीनां कृते द्वौ प्रतिमानौ प्राप्तौ : उपत्यकाजातीनां प्रकाशसंश्लेषणस्य दराः आर्द्रशुष्कऋतुषु समानाः आसन्, यदा तु सानुषु नित्यहरिद्वर्ण जातीनां आर्द्रऋतुस्य तुलने शुष्कऋतौ प्रकाशसंश्लेषणस्य दरं अप्रत्याशितरूपेण अधिकम् आसीत्, यत् सम्भवतः उष्णतरतापमानस्य प्राधान्यं सूचयति । अस्माकं परिणामाः दर्शयन्ति यत् नित्यहरिद्वर्ण वृक्षाणि सहितं सह-उपस्थिताः वृक्षजातयः स्वस्य ताप-आदर्श-तापमानस्य प्रकाशसंश्लेषणस्य विविध-ऋतुविविधतां प्रदर्शयन्ति ।

कृतज्ञता

अस्याः परियोजनायाः वित्तपोषणं UKRI अनुदानस्य NE/R००५०७९/१ इत्यस्य माध्यमेन सम्पन्नम्। DB IISER पुणे इत्यस्मै हार्दं धन्यवादं यः इन्द्रामुरल् निधिना तथा अन्येनापि समर्थनप्रदानेन सहायं कृतवान्। कोविड्–१९ महामारीकाले समग्रस्य विश्वस्वास्थ्यसेवाकर्मिणां समुदायस्य च निष्ठां प्रती अस्माकं कृतज्ञता। राकेश तिवारी उप्सालानगरे पोस्टडॉक्टोरल् वित्तपोषणार्थं वेनर्-ग्रेन् प्रतिष्ठानस्य हार्दं कृतज्ञतां निवेदयति। श्रृङ्गेर्याः श्री-शारदाचन्द्रमौलीश्वरयोः उभयोः च जगद्गुर्वोः कृते प्रदत्तमार्गदर्शनाय राकेशतिवारी विनयपूर्वकं धन्यवादं समर्पयति।

स्रोतः

Abhilash KP, Devakumar AS (2023) Seasonal photosynthesis variations of dominant tree species used in different urban landscapes. International Journal of Environment and Climate Change 13: 562–571

Andriyas T, Leksungnoen N, Tor-Ngern P (2021) Comparison of water-use characteristics of tropical tree saplings with implications for forest restoration. Sci Rep 11: 1745

Asargew MF, Masutomi Y, Kobayashi K, Aono M (2024) Water stress changes the relationship between photosynthesis and stomatal conductance. Sci Total Environ 907: 11

Ávila-Lovera E, Urich R, Coronel I, Tezara W (2019) Seasonal gas exchange and resource-use efficiency in evergreen versus deciduous species from a tropical dry forest. Tree Physiol 39: 1561–1571

Blonder BW, Aparecido LMT, Hultine KR, Lombardozzi D, Michaletz ST, Posch BC, Slot M, Winter K (2023) Plant water use theory should incorporate hypotheses about extreme environments, population ecology, and community ecology. New Phytol 238: 2271–2283

Borjigidai A, Hikosaka K, Hirose T, Hasegawa T, Okada M, Kobayashi K (2006) Seasonal changes in temperature dependence of photosynthetic rate in rice under a free-air CO2 enrichment. Ann Bot 97: 549–557

Brinkmann N, Eugster W, Buchmann N, Kahmen A (2019) Species-specific differences in water uptake depth of mature temperate trees vary with water availability in the soil. Plant Biol (Stuttg) 21: 71–81

Cai Z-Q, Schnitzer SA, Bongers F (2009) Seasonal differences in leaf-level physiology give lianas a competitive advantage over trees in a tropical seasonal forest. Oecologia 161: 25–33

Carvalho NS, Anderson LO, Nunes CA, PessÃ´a ACM, Silva Junior CH, Reis JBC, Shimabukuro YE, Berenguer E, Barlow J, AragÃ£o LEO (2021) Spatio-temporal variation in dry season determines the Amazonian fire calendar. Environ Res Lett 16: 125009

Cavanaugh JE, Neath AA (2019) The Akaike information criterion: Background, derivation, properties, application, interpretation, and refinements. Wiley Interdiscip Rev Comput Stat 11: e1460

Chen X, Maignan F, Viovy N, Bastos A, Goll D, Wu J, Liu L, Yue C, Peng S, Yuan W, Conceição AC, O'Sullivan M, Ciais P (2020) Novel representation of leaf phenology improves simulation of amazonian evergreen forest photosynthesis in a land surface model. J Adv Model Earth Syst 12:

Chitra-Tarak R, Xu C, Aguilar S, Anderson-Teixeira KJ, Chambers J, Detto M, Faybishenko B, Fisher RA, Knox RG, Koven CD, Kueppers LM, Kunert N, Kupers SJ, McDowell NG, Newman BD, Paton SR, Pérez R, Ruiz L, Sack L, Warren JM, Wolfe BT, Wright C, Wright SJ, Zailaa J, McMahon SM (2021) Hydraulically-vulnerable trees survive on deep-water access during droughts in a tropical forest. New Phytol 231: 1798–1813

Choury Z, Wujeska-Klause A, Bourne A, Bown NP, Tjoelker MG, Medlyn BE, Crous KY (2022) Tropical rainforest species have larger increases in temperature optima with warming than warm-temperate rainforest trees. New Phytol 234: 1220–1236

Comita LS, Engelbrecht BMJ (2009) Seasonal and spatial variation in water availability drive habitat associations in a tropical forest. Ecology 90: 2755–2765

Corredor-Londoño G-A, Beltrán J-W, Torres-González A-M, Sardi-Saavedra A (2020) Phenological synchrony and seasonality of eight tree species in a fragmented landscape in the Colombian Andes. Revista de Biología Tropical 68: 987–1000

Costa FRC, Schietti J, Stark SC, Smith MN (2023) The other side of tropical forest drought: do shallow water table regions of Amazonia act as large-scale hydrological refugia from drought? New Phytol 237: 714–733

Craven D, Dent D, Braden D, Ashton MS, Berlyn GP, Hall JS (2011) Seasonal variability of photosynthetic characteristics influences growth of eight tropical tree species at two sites with contrasting precipitation in Panama. For Ecol Manage 261: 1643–1653

Crous KY, Uddling J, De Kauwe MG (2022) Temperature responses of photosynthesis and respiration in evergreen trees from boreal to tropical latitudes. New Phytol 234: 353–374

Cunningham SC, Read J (2002) Comparison of Temperate and Tropical Rainforest Tree Species: Photosynthetic Responses to Growth Temperature. Oecologia 133: 112–119

Das A, Nagendra H, Anand M, Bunyan M (2015) Topographic and Bioclimatic Determinants of the Occurrence of Forest and Grassland in Tropical Montane Forest-Grassland Mosaics of the Western Ghats, India. PLoS One 10: e0130566

Devi NL, Brearley FQ, Tripathi SK (2023) Phenological diversity among sub-tropical moist forest trees of north-eastern India. J Trop Ecol 39: e29

Ding Y, Nie Y, Chen H, Wang K, Querejeta JI (2021) Water uptake depth is coordinated with leaf water potential, water-use efficiency and drought vulnerability in karst vegetation. New Phytol 229: 1339–1353

Docherty EM, Gloor E, Sponchiado D, Gilpin M, Pinto CAD, Junior HM, Coughlin I, Ferreira L, Junior JAS, da Costa ACL, Meir P, Galbraith D (2023) Long-term drought effects on the thermal sensitivity of Amazon forest trees. Plant Cell Environ 46: 185–198

Eamus D, Myers B, Duff G, Williams D (1999) Seasonal changes in photosynthesis of eight savanna tree species. Tree Physiol 19: 665–671

Esteban EJL, Castilho CV, Melgaço KL, Costa FRC (2021) The other side of droughts: wet extremes and topography as buffers of negative drought effects in an Amazonian forest. New Phytol 229: 1995–2006

Eze CE, Winter K, Slot M (2024) Vapor-pressure-deficit-controlled temperature response of photosynthesis in tropical trees. Photosynthetica 62: 318–325

Garen JC, Branch HA, Borrego I, Blonder B, Stinziano JR, Michaletz ST (2022) Gas exchange analysers exhibit large measurement error driven by internal thermal gradients. New Phytol 236: 369–384

Gjindali A, Herrmann HA, Schwartz J-M, Johnson GN, Calzadilla PI (2021) A holistic approach to study photosynthetic acclimation responses of plants to fluctuating light. Front Plant Sci 12: 668512

Gjindali A, Johnson GN (2023) Photosynthetic acclimation to changing environments. Biochem Soc Trans 51: 473–486

Gloor E, Barua D, Galbraith DR, Hegde B, Sunny R, Tiwari R (2023) Leaf water potential of tropical forest tree species, Sirsi, Western Ghats, India, 2020-2021. doi.org/10.5285/252b6a14-8a0e-4a6f-a879-99dff46fec71

Grace J, Okali DUU, Fasehun FE (1982) Stomatal conductance of two tropical trees during the wet season in Nigeria. J Appl Ecol 19: 659

Green JK, Berry J, Ciais P, Zhang Y, Gentine P (2020) Amazon rainforest photosynthesis increases in response to atmospheric dryness. Sci Adv 6:

Guha S, Jain V (2020) Role of inherent geological and climatic characteristics on landscape variability in the tectonically passive Western Ghat, India. Geomorphology (Amst) 350: 106840

Harris NL, Medina E (2013) Changes in leaf properties across an elevation gradient in the Luquillo Mountains, Puerto Rico. Ecol Bull 169–180

Hasselquist NJ, Allen MF, Santiago LS (2010) Water relations of evergreen and drought-deciduous trees along a seasonally dry tropical forest chronosequence. Oecologia 164: 881–890

Hernández GG, Winter K, Slot M (2020) Similar temperature dependence of photosynthetic parameters in sun and shade leaves of three tropical tree species. Tree Physiol 40: 637–651

Hikosaka K (2005) Nitrogen partitioning in the photosynthetic apparatus of Plantago asiatica leaves grown under different temperature and light conditions: similarities and differences between temperature and light acclimation. Plant Cell Physiol 46: 1283–1290

Ishida A, Diloksumpun S, Ladpala P, Staporn D, Panuthai S, Gamo M, Yazaki K, Ishizuka M, Puangchit L (2006) Contrasting seasonal leaf habits of canopy trees between tropical dry-deciduous and evergreen forests in Thailand. Tree Physiol 26: 643–656

Ishida A, Yamazaki J-Y, Harayama H, Yazaki K, Ladpala P, Nakano T, Adachi M, Yoshimura K, Panuthai S, Staporn D, Maeda T, Maruta E, Diloksumpun S, Puangchit L (2014) Photoprotection of evergreen and drought-deciduous tree leaves to overcome the dry season in monsoonal tropical dry forests in Thailand. Tree Physiol 34: 15–28

Janssen T, Fleischer K, Luyssaert S, Naudts K, Dolman H (2020) Drought resistance increases from the individual to the ecosystem level in highly diverse Neotropical rainforest: a meta-analysis of leaf, tree and ecosystem responses to drought. Biogeosciences 17: 2621–2645

Joshi RK, Mishra A, Gupta R, Garkoti SC (2024) Leaf and tree age-related changes in leaf ecophysiological traits, nutrient, and adaptive strategies of Alnus nepalensis in the central Himalaya. J Biosci 49: 1–14

June T, Evans JR, Farquhar GD (2004) A simple new equation for the reversible temperature dependence of photosynthetic electron transport: A study on soybean leaf. Funct Plant Biol 31:

Kailash BR, Charles B, Ravikanth G, Setty S, Kadirvelu K (2022) Identifying the potential global distribution and conservation areas for Terminalia chebula, an important medicinal tree species under changing climate scenario. Trop Ecol 63: 584–595

Kattge J, Knorr W (2007) Temperature acclimation in a biochemical model of photosynthesis: a reanalysis of data from 36 species. Plant Cell Environ 30: 1176–1190

Kitajima K, Mulkey SS, Wright SJ (1997) Seasonal leaf phenotypes in the canopy of a tropical dry forest: photosynthetic characteristics and associated traits. Oecologia 109: 490–498

Köpp Hollunder R, Garbin ML, Rubio Scarano F, Mariotte P (2022) Regional and local determinants of drought resilience in tropical forests. Ecol Evol 12: e8943

Kositsup B, Montpied P, Kasemsap P, Thaler P, Dreyer E (2008) Photosynthetic capacity and temperature responses of photosynthesis of rubber trees (Hevea brasiliensis Müll. Arg.) acclimate to changes in ambient temperatures. Trees 23:

Krishnadas M, Kumar A, Comita LS (2016) Environmental gradients structure tropical tree assemblages at the regional scale. J Veg Sci 27: 1117–1128

Krishnadas M, Sankaran M, Page N, Joshi J, Machado S, Nataraj N, Chengappa SK, Kumar V, Kumar A, Krishnamani R (2021) Seasonal drought regulates species distributions and assembly of tree communities across a tropical wet forest region. Glob Ecol Biogeogr 30: 1847–1862

Kühnhammer K, van Haren J, Kübert A, Bailey K, Dubbert M, Hu J, Ladd SN, Meredith LK, Werner C, Beyer M (2023) Deep roots mitigate drought impacts on tropical trees despite limited quantitative contribution to transpiration. Sci Total Environ 893: 164763

Liu J, Ryu Y, Luo X, Dechant B, Stocker B, Keenan T, Gentine P, Li X, Li B, Harrison S, Prentice I (2024) Evidence for widespread thermal acclimation of canopy photosynthesis. Research Square doi.org/10.21203/rs.3.rs-4013319/v1

Montagu KD, Woo KC (1999) Recovery of tree photosynthetic capacity from seasonal drought in the wet - dry tropics: the role of phyllode and canopy processes in Acacia auriculiformis. Funct Plant Biol 26: 135

Mott KA, Peak D (2011) Alternative perspective on the control of transpiration by radiation. Proceedings of the National Academy of Sciences 108: 19820–19823

Mujawamariya M, Wittemann M, Dusenge ME, Manishimwe A, Ntirugulirwa B, Zibera E, Nsabimana D, Wallin G, Uddling J (2023) Contrasting warming responses of photosynthesis in early- and late-successional tropical trees. Tree Physiol 43: 1104–1117

Muller O, Hirose T, Werger MJA, Hikosaka K (2011) Optimal use of leaf nitrogen explains seasonal changes in leaf nitrogen content of an understorey evergreen shrub. Ann Bot 108: 529–536

Naidu C, Swamy PM (1995) Seasonal pattern of photosynthetic rate and its relationship with chlorophyll content, ribulose-1,5-bisphosphate carboxylase activity and biomass production. Biol Plant 37: 349–354

Nie Y-P, Chen H-S, Wang K-L, Tan W, Deng P-Y, Yang J (2011) Seasonal water use patterns of woody species growing on the continuous dolostone outcrops and nearby thin soils in subtropical China. Plant Soil 341: 399–412

Pascal JP (1988) Wet evergreen forests of the Western Ghats of India. Institut francais de Pondichery, Pondichery

Peng J, Feng Y, Wang X, Li J, Xu G, Phonenasay S, Luo Q, Han Z, Lu W (2021) Effects of nitrogen application rate on the photosynthetic pigment, leaf fluorescence characteristics, and yield of indica hybrid rice and their interrelations. Sci Rep 11: 7485

Pinheiro J, Bates D, DebRoy S, And DS, R Core Team, (2018) nlme: Linear and nonlinear mixed effects models. In: R package

R Core Team, (2025) R: A language and environment for statistical computing

Rey-Sánchez AC, Slot M, Posada JM, Kitajima K (2016) Spatial and seasonal variation in leaf temperature within the canopy of a tropical forest. Clim Res 71: 75–89

Ribeiro RV, Machado EC, Santos MG, Oliveira RF (2009) Seasonal and diurnal changes in photosynthetic limitation of young sweet orange trees. Environ Exp Bot 66: 203–211

Santos VAHFD, Ferreira MJ, Rodrigues JVFC, Garcia MN, Ceron JVB, Nelson BW, Saleska SR (2018) Causes of reduced leaf-level photosynthesis during strong El Niño drought in a Central Amazon forest. Glob Chang Biol 24: 4266–4279

Sarkar S, Maity R (2022) Future Characteristics of Extreme Precipitation Indicate the Dominance of Frequency Over Intensity: A Multi-Model Assessment From CMIP6 Across

India. J Geophys Res D: Atmos 127:

Schmitt S, Trueba S, Coste S, Ducouret É, Tysklind N, Heuertz M, Bonal D, Burban B, Hérault B, Derroire G (2022) Seasonal variation of leaf thickness: An overlooked component of functional trait variability. Plant Biol 24: 458–463

Schwartz NB, Medvigy D, Tijerin J, Pérez-Aviles D, Rivera-Polanco D, Pereira D, Vargas G. G, Werden L, Du D, Arnold L, Powers JS (2022) Intra-annual variation in microclimatic conditions in relation to vegetation type and structure in two tropical dry forests undergoing secondary succession. For Ecol Manage 511: 120132

Sendall KM, Vourlitis GL, Lobo FA (2009) Seasonal variation in the maximum rate of leaf gas exchange of canopy and understory tree species in an Amazonian semi-deciduous forest. Braz J Plant Physiol 21: 65–74

Shi C, Sun G, Zhang H, Xiao B, Ze B, Zhang N, Wu N (2014) Effects of Warming on Chlorophyll Degradation and Carbohydrate Accumulation of Alpine Herbaceous Species during Plant Senescence on the Tibetan Plateau. PLoS One 9: e107874

Shigwan BK, Kulkarni A, Smrithy V, Datar MN (2024) An overview of tree ecology and forest studies in the Northern Western Ghats of India. IForest 17: 213–221

Slot M, Winter K (2017a) Photosynthetic acclimation to warming in tropical forest tree seedlings. J Exp Bot 68: 2275–2284

Slot M, Winter K (2017b) In situ temperature relationships of biochemical and stomatal controls of photosynthesis in four lowland tropical tree species. Plant Cell Environ 40: 3055–3068

Stahl C, Hérault B, Rossi V, Burban B, Bréchet C, Bonal D (2013) Depth of soil water uptake by tropical rainforest trees during dry periods: does tree dimension matter? Oecologia 173: 1191–1201

Still CJ, Sibley A, Page G, Meinzer FC, Sevanto S (2019) When a cuvette is not a canopy: A caution about measuring leaf temperature during gas exchange measurements. Agric For Meteorol 279: 107737

Tange T (1996) Seasonal changes in photosynthesis of young Cryptomeria japonica growing on ridges and foot-slopes. For Ecol Manage 89: 93–99

Urban J, Ingwers MW, McGuire MA, Teskey RO (2017) Increase in leaf temperature opens stomata and decouples net photosynthesis from stomatal conductance in *Pinus taeda* and *Populus deltoides x nigra*. J Exp Bot 68: 1757–1767

Uribe MR, Sierra CA, Dukes JS (2021) Seasonality of tropical photosynthesis: A pantropical map of correlations with precipitation and radiation and comparison to model outputs. J Geophys Res Biogeosci 126:

Vourlitis GL, de Souza Nogueira J, de Almeida Lobo F, Sendall KM, de Paulo SR, Dias CAA, Pinto OB, de Andrade NLR (2008) Energy balance and canopy conductance of a tropical semi-deciduous forest of the southern Amazon Basin. Water Resources Research 44:

Wada N, Kondo I, Tanaka R, Kishimoto J, Miyagi A, Kawai-Yamada M, Mizokami Y, Noguchi K (2023) Dynamic seasonal changes in photosynthesis systems in leaves of Asarum tamaense, an evergreen understorey herbaceous species. Ann Bot 131: 423–436

Way DA, Yamori W (2014) Thermal acclimation of photosynthesis: on the importance of adjusting our definitions and accounting for thermal acclimation of respiration. Photosynth Res 119: 89–100

Wild J, Kopecký M, Macek M, Šanda M, Jankovec J, Haase T (2019) Climate at ecologically relevant scales: A new temperature and soil moisture logger for long-term microclimate measurement. Agric For Meteorol 268: 40–47

Wittemann M, Andersson MX, Ntirugulirwa B, Tarvainen L, Wallin G, Uddling J (2022) Temperature acclimation of net photosynthesis and its underlying component processes in four tropical tree species. Tree Physiol 42: 1188–1202

Wu J, Albert LP, Lopes AP, Restrepo-Coupe N, Hayek M, Wiedemann KT, Guan K, Stark SC, Christoffersen B, Prohaska N, Tavares JV, Marostica S, Kobayashi H, Ferreira ML, Campos KS, da Silva R, Brando PM, Dye DG, Huxman TE, Huete AR, Nelson BW, Saleska SR (2016) Leaf development and demography explain photosynthetic seasonality in Amazon evergreen forests. Science 351: 972–976

Yamaguchi DP, Nakaji T, Hiura T, Hikosaka K (2016) Effects of seasonal change and experimental warming on the temperature dependence of photosynthesis in the canopy leaves of Quercus serrata. Tree Physiol 36: 1283–1295

Yamasaki T, Yamakawa T, Yamane Y, Koike H, Satoh K, Katoh S (2002) Temperature Acclimation of Photosynthesis and Related Changes in Photosystem II Electron Transport in Winter Wheat. Plant Physiol 128: 1087–1097

Yamori W, Hikosaka K, Way DA (2014) Temperature response of photosynthesis in C3, C4, and CAM plants: temperature acclimation and temperature adaptation. Photosynth Res 119: 101–117

Yamori W, Noguchi K, Hanba YT, Terashima I (2006) Effects of internal conductance on the temperature dependence of the photosynthetic rate in spinach leaves from contrasting growth temperatures. Plant Cell Physiol 47: 1069–1080

Yasumura Y, Hikosaka K, Hirose T (2006) Seasonal changes in photosynthesis, nitrogen content and nitrogen partitioning in Lindera umbellata leaves grown in high or low irradiance. Tree Physiol 26: 1315–1323

Zhang C, Su Y, Liu L, Wu J, Huang G, Li X, Bi C, Yan W, Lafortezza R (2023) Seasonal and long-term dynamics in forest microclimate effects: global pattern and mechanism. Npj Clim Atmos Sci 6: 1–12

Zhang J-L, Zhu J-J, Cao K-F (2007) Seasonal variation in photosynthesis in six woody species with different leaf phenology in a valley savanna in southwestern China. Trees 21: 631–643

Glossary of English and Sanskrit terms used

Air – वायुः, assimilation rate – उपग्रहण गतिः, brevideciduous – आंशिक पर्णपाति, buffer – रक्षणम् , bulk density – संहति सान्द्रता, canopy tree – पत्रावरणम्, climate – दीर्घ कालीन वातावरणम् , co-occurring – सहसम्भवः , coexisting – सहसम्भवः, data – दत्तांशः / तथ्यसमूहः, deciduous – पतनशीलः, deciduous leaf habit – पतनशीलपत्रस्वभावः, dry affinity – शुष्क प्राधान्यम् , evergreen leaf habit – नित्यहरिद्वर्णपत्रस्वभावः , mathametical model fit – गणतीय अनुरूपणम् , fluorescence – प्रभा प्रत्युत्सर्गः, in situ – स्वस्थानिकः, insulated thermo couple – विद्युन्निरोधित ताप सन्धिः, irreversible – अनावर्त्यः, juveniles – वृक्ष तरु / किशोरवृक्षाः, landscape – भूदृश्यं, leaf chamber – पत्रकोश, leaf habit – पत्रस्वभावः, leaf phenology – पत्रऋतुविकासः, light saturated – प्रकाश सम्पूर्णः, logger – लेखायन्त्रम् , measure/measurement – मापनम् , microclimate – सूक्ष्मवातावरणम् , niche preference – तापगुणविशिष्ट क्षेत्रप्राधान्यं, optimal – आदर्शः, optimisation – उत्तमीकरणम् , parameter – नियताङ्कः, petriplate – पेट्री पट्टिका, phenology – ऋतुपरिवर्तन विज्ञानम् , photoperiod – प्रकाशकालः, photoprotection – प्रकाश संरक्षणम् , photosynthesis – प्रकाश संश्लेषणम् , photosynthetic CO_2 assimilation – प्रकाशीय CO_2 उपग्रहणम् , photosystem – प्रकाश संहिता, physiology – क्रियाप्रक्रिया, plastic – परिवर्तन शीलता, plot – चित्रपटलम् , proxy – प्रतिनिधिः, quadratic /second order – द्वितीय घातः, reversible – आवर्तितः, season – ऋतु / ऋतुमानम् , seasonal – ऋतुपरिणामजन्यः, seasonal difference – ऋतु वैविध्यम् , ऋतु भेदः – seedling अङ्कुरः / नवांकुरः, species – जातिः / प्रजातिविशेषः, species specific – प्रजाति निर्दिष्टः, stomata – पत्रछिद्राणि, stomatal conductance – पत्रछिद्र वाहकता, subcanopy (tree) – उपावरणम् , thermal optima – आदर्श तापमानम् , thermal tolerance – तापसहिष्णुता, topography – स्थालाकृतिः, traits – लक्षणानि, transpiration – स्वेदस्रवणम् , tree species – वृक्षप्रजातिः, tropical – उष्णवलय, understorey tree – अधःवितान वृक्षः, undulating – उत्थानपतनात्मक, unit – मानम् / प्रमाणम् , warmer thermal niche preference – उष्णतर तापगुण क्षेत्र प्राधान्यम् , water availability – जलउपलब्धता, weather – वर्तमान वातावरणम् , wet – आर्द्रः, wind – वातः, y-axis – लंबाक्षः
